# Mind the gap: A review and recommendations for statistically evaluating Dual Systems models of adolescent risk behavior

**DOI:** 10.1016/j.dcn.2019.100681

**Published:** 2019-07-25

**Authors:** Samuel N. Meisel, Whitney D. Fosco, Larry W. Hawk, Craig R. Colder

**Affiliations:** a University at Buffalo, The State University of New York, United States; b Center for Children and Families, Florida International University, United States

**Keywords:** Dual systems models, Imbalance hypothesis, Sensation seeking, Self-Regulation, Latent difference scores, Growth mixture modeling

## Abstract

According to Dual Systems models (Casey et al., 2008; Luna and Wright, 2016; Steinberg, 2008), a rapidly-developing socioemotional system and gradually-developing cognitive control system characterize adolescent brain development. The imbalance hypothesis forwarded by Dual Systems models posits that the magnitude of the imbalance between these two developing systems should predict the propensity for engaging in a variety of risk behaviors. The current integrative review argues that the excitement generated by the imbalance hypothesis and its implications for explaining adolescent risk behaviors has not been meet with equal efforts to rigorously test this hypothesis. The goal of the current review is to help guide the field to consider appropriate and rigorous methods of testing the imbalance hypothesis. First, we review the analytic approaches that have been used to test the imbalance hypothesis and outline statistical and conceptual limitations of these approaches. Next, we discuss the utility of two longitudinal analytic approaches (Latent Difference Scores and Growth Mixture Modeling) for testing the imbalance hypothesis. We utilize data from a large community adolescent sample to illustrate each approach and argue that Latent Difference Scores and Growth Mixture Modeling approaches enhance the specificity and precision with which the imbalance hypothesis is evaluated.

## 1 Introduction

Adolescence is a developmental period marked by the emergence of a range of risk behaviors and mental health concerns that can persist into adulthood for some youth (Glied and Pine, 2002; IOM/NRC, 2011). These behaviors include the initiation and escalation of substance use (Colder et al., 2002; Miech et al., 2017), risky sexual activity and high rates of sexually transmitted diseases (Satterwhite et al., 2013), and fatal car crashes (Insurance Institute for Highway Safety, 2010). Moreover, rates of externalizing disorders, including oppositional defiant disorder, conduct disorder, and substance use disorders increase from early to late adolescence (Merikangas et al., 2010).

**Fig. 1:**
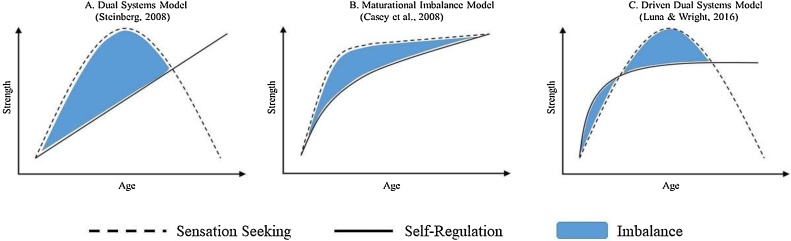
Adapted with permission from Shulman et al. (2016a). The figure depicts three Dual Systems models and the development of sensation seeking and self-regulation from late childhood to young adulthood according to each of these models. The blue portion in each model represents the *imbalance* between sensation seeking and self-regulation. The challenge when assessing the imbalance hypothesis is to use a data analytic technique that captures the difference between sensation seeking and self-regulation. Further, each of these Dual Systems model posit systematic changes in sensation seeking and self-regulation across time, therefore, data analytic techniques used to assess the imbalance hypothesis must also be able to capture the proposed developmental differences in sensation seeking and self-regulation from late childhood to young adulthood. A model that captures the dashed line (sensation seeking), either at a single time point or across time, is not assessing the imbalance. Similarly, a model that captures the solid line (self-regulation), either at a single time point or across time, is also not assessing the imbalance. Further, a model that simultaneously models the dashed line (sensation seeking) and solid line (self-regulation), at a singly time point, is not modeling the imbalance. We argue that only data analytic approaches that quantify the imbalance between the dashed and solid lines (the blue portion of each Dual Systems model) and account for developmental changes in imbalance can be rigorous tests of the imbalance hypothesis. (For interpretation of the references to colour in this figure legend, the reader is referred to the web version of this article).

The Dual Systems Model (Steinberg, 2008), Maturational Imbalance Model (Casey et al., 2008), and Driven Dual Systems Model (Luna and Wright, 2016), provide similar theoretical accounts for the increase in risk behaviors seen during adolescence. A common tenet of these leading developmental neuroscience models is that adolescent risk behaviors results from an imbalance between the development of a cognitive control and a socioemotional neural system (see Fig. 1). The prefrontal cortex primarily encompasses the cognitive control system, which includes executive functions and other higher-order self-regulatory processes necessary for top-down control of behavior (Velanova et al., 2009). Behaviorally, cognitive control involves the ability to adjust behavior in response to changing task demands and inhibit behavior that is no longer adaptive (Nigg, 2017). Cognitive control requires integrations of inhibitory control, conflict monitoring, working memory, and attentional control, though most work in this area focuses on tasks that assess inhibition (see Shulman et al., 2016a). Common paradigms include the Stop Signal Task (SST), Go/No-Go, and antisaccade tasks. Briefly, all three tasks require participants to withhold a prepotent response and instead execute a subdominant response.

The socioemotional system is mediated by subcortical dopaminergic regions, particularly the striatum, and is responsible for enhancing the motivational salience of rewarding stimuli (Braams et al., 2015). Indicators of the socioemotional system at a behavioral level include self-report measures such as the Sensation Seeking Scale (Zuckerman et al., 1978) and the BIS/BAS Questionnaire (Carver and White, 1994), as well as paradigms such as the Iowa Gambling Task (Bechara et al., 1994; Cauffman et al., 2010) and the Point Score Reaction Time Task for Children-Revised (Colder et al., 2011). Throughout the review, we refer to these systems at the level of the psychological, rather than the biological, construct. Accordingly, “sensation seeking” will refer to the socioemotional system, and “self-regulation” will refer to the cognitive control system (Smith et al., 2013).

We recognize that Dual Systems models diverge in a number of important ways, including the extent to which the development of sensation seeking and self-regulation should be orthogonal (Dual Systems Model) or interdependent (Maturation Imbalance) and whether the hypothesized trajectories of growth of each system are linear versus curvilinear (Shulman et al., 2016b, a). However, despite these differences, a cornerstone of all three models is the *imbalance hypothesis* – that gradual maturation of self-regulation is outpaced by rapid developments in sensation seeking, particularly during the first half of adolescence (Casey et al., 2008; Steinberg, 2008; Steinberg et al., 2008). This imbalance between sensation seeking and self-regulation is hypothesized to result in myriad adolescent risky behaviors (see Fig. 1 for a depiction of the imbalance forwarded by Dual Systems models). That is, motivation for risky behavior increases in adolescence, and the systems responsible for inhibiting those behaviors have not yet fully developed.

The Lifespan Wisdom Model (Romer et al., 2017) is related to dual systems models and the imbalance hypothesis, yet there are notable differences. For example, the Lifespan Wisdom Model asserts that the imbalance between sensation seeking and cognitive control is best represented by high levels of acting without thinking (poor impulse control), rather than separate measures of each system. Given this feature, it is unclear if the model falls under the rubric of a dual systems model. Furthermore, the model posits that imbalance (poor impulse control) characterizes only a subset of youth rather than being part of a normative developmental trajectory.

The centrality of the imbalance hypothesis to different theoretical approaches and models suggests the importance of using appropriate methods to test this hypothesis. As we argue below, we believe that data analytic methods used to date fail to appropriately capture the difference between sensation seeking and self-regulation and how that difference predicts risk behavior. The primary purpose of the current review is to discuss data analytic methods that provide a rigorous test of the imbalance hypothesis with longitudinal data. For the sake of simplicity, we refer to the Dual Systems Model (Steinberg, 2008), Maturational Imbalance Model (Casey et al., 2008), and Driven Dual Systems Model (Luna and Wright, 2016) collectively as Dual Systems models.

### 1.1 Critiques of dual systems models

Dual Systems models have spurred a large volume of research on adolescent brain development that has expanded our understanding of the neural underpinnings of adolescent risk behaviors (Shulman et al., 2016a). These models have become increasingly influential over the last decade, providing a theoretical framework for empirical investigations of substance use, delinquency, and myriad other risk behaviors, and also shaping public policy and juvenile justice decisions regarding adolescent delinquent behaviors (Casey et al., 2017; Rhyner et al., 2017; Scott et al., 2016; Steinberg, 2017).

Although these models have stimulated a productive and informative line of inquiry, researchers have identified several limitations. First, recent papers have noted a lack of specificity (e.g., according to Dual Systems models, how would one go about testing the imbalance and its proposed relationship to risk behavior?) and questionable falsifiability (e.g., how could one provide strong evidence that the imbalance is not implicated in risk behaviors?), suggesting that Dual Systems models of adolescent risk behavior are informative heuristics, rather than testable theories (van den Bos and Eppinger, 2016; van Duijvenvoorde et al., 2016; Pfeifer and Allen, 2016). In line with this critique, several recent papers have called for the need for greater specificity of Dual Systems models (Pfeifer and Allen, 2016; van den Bos and Eppinger, 2016).

A second critique of Dual Systems models is that there is limited evidence demonstrating that an imbalance between sensation seeking and self-regulation in adolescence is associated with real-world risk behavior (Johnson et al., 2009; Pfeifer and Allen, 2012; Best and Miller, 2010; Casey and Jones, 2010). As the field continues to accumulate neuroimaging data on the development of the socioemotional and cognitive control systems in adolescence, it is critical that the neuroimaging findings are complemented by rigorous behavioral evidence to inform the extent to which patterns of brain activation predict engagement in different types of risk behavior in adolescence (Pfeifer and Allen, 2016).

Although Dual Systems models are developmental in nature, most of the evidence in support of them is cross-sectional (Crone, van Duijenvoorde, & Peper, 2016; Shulman et al., 2016a). As shown in Fig. 1, the imbalance between the systems should grow from early to middle adolescence, peak in middle adolescence, and then diminish (Steinberg, 2008). Most previous work in this area has quantified the imbalance without regard to its developmental trajectory (e.g., Kim-Spoon et al., 2016; Rhodes et al., 2013; Vazsonyi and Ksinan, 2017). As more longitudinal studies are being undertaken to address this concern, it is important to consider analytic methods appropriate to describing and testing the complex developmental patterns hypothesized in these models (Lydon-Staley and Bassett, 2018).

### 1.2 Improving the specificity of the imbalance hypothesis

The current integrative review seeks to address these limitations. Our goals are to delineate the specificity issues and the lack of behavioral and longitudinal data in support of this hypothesis (Pfeifer and Allen, 2016) and address key conceptual and statistical considerations involved in operationalizing and testing the imbalance hypothesis with behavioral data in order to facilitate the generation of more precise predictions. We focus on behavioral data because of recent calls for empirical papers that assess Dual Systems models with behavioral data and the importance of demonstrating that the imbalance does in fact impact real-world risk behavior (Pfeifer and Allen, 2016). However, the data analytic methods discussed below could readily be applied to neuroimaging data.

We first review analytic approaches that have been used to date to quantify the imbalance between sensation seeking and self-regulation and describe both conceptual and statistical limitations of these approaches. To begin to address the aforementioned limitations on specificity, we also clearly delineate what information these analytic approaches provide and what questions each approach is able to answer. Next, we present several longitudinal analytic approaches that more appropriately test the imbalance hypothesis than previously-used methods in this area.

We hope the present paper leads the field to further consider the following questions: How do we test the imbalance with longitudinal data? What can the statistical analyses we use tell us about the imbalance and its development over time, as well as the relation of the imbalance to risk behaviors? Do current analytic approaches advance theory and move it in a direction towards falsifiability? Before delving into these issues, we take a short detour to discuss issues of measurement.

### 1.3 A brief word on measurement of the imbalance

Dual Systems models have repeatedly been criticized for their lack of specificity regarding how to measure the socioemotional and cognitive control systems (Duckworth and Steinberg, 2015; Harden et al., 2017; Pfeifer and Allen, 2016). This lack of precision has resulted in the same measures (e.g., delay discounting, acting without thinking) being used as indicators of both cognitive control (Shulman and Cauffman, 2014; Vazsonyi and Ksinan, 2017; van den Bos et al., 2015) and socioemotional systems (Cyders et al., 2009; Weigard et al., 2013). Considering prior reviews and commentaries have discussed these issues at length (Duckworth and Steinberg, 2015; Nigg, 2017; Pfeifer and Allen, 2016), we briefly note the implications of imprecise measurement of the socioemotional and cognitive control systems on testing the imbalance hypothesis.

The Dual Systems Model, Maturational Imbalance Model, and Driven Dual Systems Model all necessitate separate measures of the socioemotional system and cognitive control system to assess the imbalance (e.g., Duell et al., 2016). In contrast, the Lifespan Wisdom Model asserts that measuring poor impulse control (acting without thinking) captures the imbalance because of its positive association with reward sensitivity and negative association with cognitive control (Khurana et al., 2018). These different conceptualizations raise the question of how best to distinguish measures of the imbalance from measures/constructs that reflect correlates of the imbalance, an issue that has garnered little attention in the literature. Moreover, these varying conceptualizations of the imbalance have significant implications for the statistical methods needed to assess the imbalance hypothesis. Because the Dual Systems Model, Maturational Imbalance Model, and Driven Dual Systems Model all call for separate measures of the socioemotional and cognitive control systems, statistical techniques must capture the *difference* between two variables across time (see Fig. 1). In contrast, ascribing to the Lifespan Wisdom Model’s operationalization of measuring the imbalance only requires the modeling of a single variable across time.

The lack of a clear operationalization of these systems is of concern because it limits research progress for Dual Systems models and undermines efforts towards increased precision and risky tests of the imbalance hypothesis (Harden et al., 2017; Pfeifer and Allen, 2016). Our review of past analytic methods and presentation of alternative longitudinal data analytic approaches focuses on studies that conceptualize the imbalance as the difference between separate measures of sensation seeking and self-regulation as this seems to characterize most dual systems perspectives. However, where appropriate, we also highlight how our longitudinal methods can be applied to conceptualizations of a single indicator, poor impulse control, as reflecting the imbalance.

### 1.4 Review of methods used to assess the imbalance

Literature Search and Inclusion Criteria

We do not to provide an exhaustive review of all studies that have attempted to test the imbalance hypothesis (for a comprehensive review see Shulman et al., 2016a). Rather, our aim is to bring attention to the conceptual and statistical limitations of methods commonly used to test the imbalance hypothesis and to recommend alternative statistical methods that are better suited to test this tenet of Dual Systems models. Articles were identified through PubMed, Psych Info, and Google Scholar. Searches included keywords such as Dual Systems, Maturational Imbalance, socioemotional system, sensation seeking, reward sensitivity, cognitive control system, self-regulation, inhibitory control and risk behaviors (e.g., substance use, alcohol use, marijuana use, cigarette use, risky driving). Further articles were identified through searching reference sections of relevant articles. For inclusion, studies were required to have adolescent samples and behavioral measures assessing the socioemotional and self-regulation systems, as well as behavioral indicators of risk behavior. We focused primarily on studies that purported to directly test Dual Systems models or were cited as evidence of Dual Systems models. Table 1 provides a summary of the methods reviewed to test the imbalance hypothesis.

**Table 1:** Summary of Current Methods as well as Recommended Alternative Models to Testing the Imbalance Hypothesis.

Previous Approaches to Modeling the Imbalance	Description of Modeling Approach	Question that can be Answered	Limitations
The Regression Approach	• Indicators of self-regulation and sensation seeking are used to predict a risk outcome while controlling for the effects of the other.	• Does one system (either self-regulation or sensation seeking) predict risk outcomes uniquely, above and beyond the effect of the other system?	• Provides no information about whether the *imbalance* between sensation seeking and self-regulation is related to risk behaviors.
			• No information is provided about how developmental changes in the imbalance is related to risk behaviors.
The Moderation Approach	• The interaction of sensation seeking and self-regulation is used to predict risk outcomes.	• Does the association between sensation seeking and risk behavior vary depending on levels of self-regulation (or vice versa)?	• An interaction is not statistically equivalent to a difference score.
			• Individuals with the same imbalance will have different risk propensities when using the moderation approach.
			• No information is provided about how developmental changes in the imbalance is related to risk behaviors.
The Observed Difference Score Approach	• The difference between an indicator of sensation seeking and self-regulation (sensation seeking – self-regulation) is used to predict risk outcomes.	• Does the observed difference between indicators of sensation seeking and self-regulation predict risk outcomes?	• Observed difference scores often have poor reliability, especially in situations where the components that make up the difference score are correlated.
			• If the variances of sensation seeking and self-regulation indicators are not nearly equivalent in magnitude, the difference score will not reflect the difference between sensation seeking and self-regulation.
			• Indicators of sensation seeking and self-regulation must be on the same metric for the difference score to be meaningful.
			• No information is provided about how developmental changes in the imbalance is related to risk behaviors.
**Recommended Approaches to Modeling the Imbalance**			
Latent Difference Score Growth Model Approach	• Latent difference scores, which represent the imbalance between sensation seeking and self-regulation, after accounting for measurement error, can be used to predict risk outcomes. Further, a growth curve can be fit to the latent difference scores so changes in the imbalance across age (or waves) can be used to predict risk outcomes.	• Does the latent difference between indicators of sensation seeking and self-regulation (imbalance) predict risk outcomes?	• Indicators of sensation seeking and self-regulation must be on the same metric for the difference score to be meaningful (solution provided in text).
			• Based on the specification of these models, examination of whether the latent change score can account for unique variance in risk behaviors above and beyond sensation seeking or self-regulation is not possible.
		• How do the latent difference scores (imbalance) change across adolescence?	
		• Is growth in the imbalance related to risk outcomes?	
Growth Mixture Modeling Approach	• Identifies subgroups of adolescents based on their growth trajectories of sensation seeking and self-regulation.	• Are there distinct patterns of growth in sensation seeking and self-regulation across adolescence for subgroups of adolescents?	• The reliability and validity of subgroups identified in mixture modeling has been questioned.
			• Subgroups often do not replicate across samples.
		• Are distinct groups of adolescents, who are characterized by particular changes in sensation seeking and self-regulation, more prone to risk behaviors than other groups of adolescence?	

#### 1.4.1 Method 1: regression analyses demonstrating unique effects of sensation seeking and self-regulation

Across all methods discussed below, a key limitation is that they are not well suited to model the imbalance as a function of age. The cross-sectional designs used to date to test the imbalance hypothesis preclude examination of developmental changes in the imbalance, which is essential for testing Dual Systems models.

A simple method researchers have used to assess the imbalance hypothesis involves regression analyses, in which indicators of sensation seeking and self-regulation are used to predict a risk outcome, while controlling for the effects of the other process (e.g., Cyders et al., 2009; Shulman and Cauffman, 2014). Although finding unique effects of sensation seeking and self-regulation supports the tenet of Dual Systems models that sensation seeking and self-regulation are implicated in risk behavior in adolescence (Steinberg, 2008), studies demonstrating unique effects of sensation seeking and self-regulation have been cited as evidence that heightened sensation seeking in the context of low self-regulation is implicated in adolescent risk taking (Shulman et al., 2016a). Yet, a regression approach testing unique effects does not, in fact, take into account one’s standing on one variable relative to the other variable, and therefore provides no information about an imbalance between the two systems. Thus, because the regression approach does not characterize the imbalance between sensation seeking and self-regulation and it does not account for developmental changes, we argue that it is not an informative statistical test of the imbalance hypothesis.

#### 1.4.2 Method 2: moderation of sensation seeking x self-regulation

An extension of the regression approach is to include cross-product interaction terms (sensation seeking x self-regulation interaction terms) to predict risk behavior. To date, a number of studies have argued that assessing interactions of sensation seeking x self-regulation is a test of the imbalance hypothesis (Duell et al., 2016; Kim-Spoon et al., 2016, 2017; McCabe et al., 2015; Peeters et al., 2017; Rhodes et al., 2013; Wasserman et al., 2017), and we agree that the interaction can provide information regarding the imbalance. For example, Peeters et al. (2017) examined the interaction between sensation seeking assessed at age 16 and effortful control (a behavioral indicator of the self-regulation system) assessed at age 11 on adolescent drug use at age 16. The authors found support for an interaction, such that sensation seeking was related to alcohol and cannabis use for adolescents characterized by low effortful control. Conversely, there was no relationship between sensation seeking and alcohol or cannabis use for adolescents characterized by high effortful control. A similar analytic approach was used by Rhodes et al. (2013). These authors used behavioral tasks to assess sensation seeking and inhibitory control and found that high levels of sensation seeking prospectively predicted increases in delinquency, but only at low levels of inhibitory control.

There are several notable limitations of the moderation approach, although it does represent an improvement over the regression approaches that only examine unique effects of sensation seeking and self-regulation. First, as discussed above, assessing moderation using a standard regression approach does not provide any information about development of sensation seeking and self-regulation. Theory argues that the imbalance should be the largest in middle adolescence and decline in late adolescence and young adulthood (see Fig. 1). Testing this assertion, even with cross-sectional data, would require testing a three-way interaction term between sensation seeking, self-regulation, and age (see Duell et al., 2016).

A more serious concern regarding the moderation approach is that interaction terms do not uniquely and solely capture the imbalance between sensation seeking and self-regulation. Although moderation can demonstrate that self-regulation may modulate sensation seeking, arguing that moderation is a test of the imbalance hypothesis is statistically inaccurate (Edwards, 2001). We use hypothetical data from three adolescents to illustrate this point. Imagine three adolescents, Bill, Bob, and Barbara, who each completed measures of sensation seeking and self-regulation (higher scores on these hypothetical measures indicate stronger sensation seeking and self-regulation). Bill’s standardized score on the measure of sensation seeking was 1.50, and his standardized score on the measure of self-regulation was 1.20. Bob’s scores on sensation seeking and self-regulation were 0.20 and -0.10, respectively, and Barbara’s scores on sensation seeking and self-regulation were -0.60 and -0.90, respectively. The imbalance, or difference between the strength of sensation seeking and self-regulation, is the same for all three teens (0.30), and suggests that they should have the same risk level for engaging in risk behavior. Despite the imbalance being the same for all three adolescents, their estimated propensity for risk behavior varies considerably in a moderation approach. To illustrate this point, below is the overall regression equation for examining moderation of sensation seeking (SS) x self-regulation (SR) on a risk outcome, as well as the regression equations for Billy, Bobby, and Barbara predicting a risk behavior (Y).

$$Y=\beta_{0\;}+\;\beta_{1}SS+\;\beta_{2}SR+\;\beta_{3\;}SSxSR+\varepsilon$$

$$Y_{Bill}=\beta_{0\;}+\;\beta_{1}(1.50)+\;\beta_{2}(1.20)+\;\beta_{3\;}(1.80)$$

$$Y_{Bob}=\beta_{0\;}+\;\beta_{1}(0.20)+\;\beta_{2}(-0.10)+\;\beta_{3\;}(-0.02)$$

$$Y_{Barbara}=\beta_{0\;}+\;\beta_{1}(-0.60)+\;\beta_{2}(-0.90)+\;\beta_{3\;}(0.54)$$

If we plug in plausible regression coefficient values for *β_0_* (0.50), *β_1_* (0.30), *β_2_* (-0.15), *β_3_* (-0.05), the Y values for Bill (0.68), Bob (0.58), and Barbara (0.43) all differ, despite their equal imbalance between sensation seeking and self-regulation (0.30). This demonstrates that moderation does not solely capture the difference between sensation seeking and self-regulation because individuals with the same imbalance will have different predicted Y values on an outcome with different combinations of X values. For a more thorough explanation of how moderation does not accurately capture an imbalance, please see Edwards (2001). Thus, we urge researchers to be mindful in their interpretations of interactions between sensation seeking and self-regulation because these interaction terms do not solely reflect the imbalance. Considering this point, we do not recommend using the moderation approach when testing the imbalance hypothesis.

#### 1.4.3 Method 3: observed difference scores between sensation seeking and self-regulation

A useful alternative is to consider a difference score in a regression model. The difference between sensation seeking and self-regulation has the advantage of clear conceptual linkage to the imbalance hypothesis. A greater difference can be conceptualized as a larger imbalance. Below is the regression equation for the difference between sensation seeking and self-regulation as a predictor of risk behavior.

$$Y=\beta_{0\;}+\;\beta_{1}|\;SS-SR|$$

When using the same values for *β_0_* (0.50) and *β_1_* (0.30) as the previous example and plugging in the values on sensation seeking and self-regulation for Bill, Bob, and Barbara, the risk values are the same across all three adolescents (Y = 0.59), as they should be, given that their imbalance is equal.

The observed difference score approach may be advantageous over moderation because it uniquely informs whether the difference in the two systems accounts for variance in a risk outcome. In their cross-sectional study of individuals ages 12–28, Vazsonyi and Ksinan (2017) found that a difference score between their measures of sensation seeking and impulsivity predicted deviancy, such that individuals characterized by high levels of sensation seeking relative to levels of impulse control were more likely to engage in deviant behaviors.

However, there are conceptual and statistical concerns under certain circumstances that researchers should be mindful of when using observed difference scores. Several papers have outlined the primary issues with observed difference scores, including ambiguity, confounded effects, untested constraints, dimensional reduction, and, in particular, concerns about reliability (Cronbach and Furby, 1970; Edwards, 1994). We briefly touch on a few of these concerns most central to Dual Systems models but recommend Edwards (2001, 1994) and Johns (1981) for in-depth discussions on these issues. Concerns regarding ambiguity stem from collapsing two measures into a single variable, the variance of which is a function of the variances of the two components from whence it came (Edwards, 1994). The first issue related to ambiguity is determining how to create a meaningful difference score when the indicators of sensation seeking and self-regulation are on different metrics (Cronbach and Furby, 1970). In testing a difference score, Vazsonyi and Ksinan (2017) standardized their indices of sensations seeking and impulse control. Although they do not provide a rationale for why they standardized, the likely motivation was to create a meaningful difference score by placing the two components on the same standardized metric. In the subsequent section on modeling the imbalance with longitudinal data, we present a method of placing sensation seeking and self-regulation measures on the same metric using longitudinal data.

The second issue concerning ambiguity is whether it is accurate to state that a difference score between sensation seeking and self-regulation accurately reflects the difference (imbalance) between these two variables, as opposed to either reflecting sensation seeking or self-regulation (Edwards, 2001). The relative contribution of each component to a difference score depends heavily on the variance of each component (Edwards, 1994). For example, if a measure of sensation seeking is found to have much greater variance than a measure of self-regulation, then the difference score created by subtracting sensation seeking and self-regulation would primarily reflect sensation seeking and not self-regulation. The variance of measures of sensation seeking and self-regulation would have to be comparable in magnitude to attenuate this concern. Further, establishing a nomological network (Cronbach and Meehl, 1955) where the observed difference score is correlated with other constructs in a theoretically-consistent manner has been suggested as a method of attenuating these ambiguity concerns (Cronbach and Furby, 1970; Johns, 1981). Specifically, a researcher could correlate the indicator of sensation seeking and self-regulation, as well as the sensation seeking – self-regulation difference score, with a host of other variables (e.g., puberty, risk-taking, peer delinquency, temperament), and the difference score should demonstrate a pattern of correlations that is distinct from the correlation patterns of either sensation seeking or self-regulation alone.

An important assumption of using a difference score is that the components have equal regression weights of opposite signs when predicting the outcome of interest and that they account for roughly equivalent amounts of variance in an outcome. A strength of Vazsonyi and Ksinan (2017) is that they examined these constraints to determine if their data met this assumption. For example, the authors demonstrated that the effects of impulse control (*β*=-0.25) and sensation seeking (*β* = .23) were both significantly associated with deviance (their outcome of interest) and were opposite in sign and nearly equivalent in magnitude.

Lastly, reliability has been repeatedly cited as a concern with observed difference scores (Cronbach and Furby, 1970; Edwards, 1991). As the covariance between components increases, the reliability of observed difference scores decreases (Johns, 1981). This issue may be particularly problematic for tests of Dual Systems models because most studies find moderate correlations between their measures of sensation seeking and self-regulation (Steinberg et al., 2008; Vazsonyi and Ksinan, 2017).

Despite these concerns, we view the observed difference score method as the strongest method used to date to test the imbalance hypothesis when using separate measures of sensation seeking and self-regulation, as long as the aforementioned assumptions are met. In line with Vazsonyi and Ksinan (2017), we urge researchers to be mindful of the potential issues of using observed difference scores and test the appropriateness of using observed difference scores when feasible.

### 1.5 Summary of methods to date

To summarize, the primary methods used to date to assess the imbalance between sensation seeking and self-regulation with behavioral data include 1) regressions, in which a measure representing one system predicts the outcome, while controlling for levels of a measure representing the other system, 2) interactions of indicators of the two systems predicting risk outcomes, and 3) difference scores. Although the regression and moderation approaches are the most common, these statistical methods provide inadequate tests of the imbalance hypothesis. Observed difference scores provide the strongest test to date of the imbalance hypothesis, although researchers should be mindful of the assumptions and potential drawbacks with this approach.

Critically, all three analytic approaches have almost exclusively utilized cross-sectional data, which provides no information about developmental change. Although Peeters et al. (2017) and Rhodes et al. (2013) had longitudinal samples, sensation seeking and self-regulation were assessed once, precluding analysis of maturation of these processes. As seen in Fig. 1 and articulated in theoretical papers on the imbalance hypothesis (Steinberg et al., 2008; Casey and Jones, 2010), a central tenet of this hypothesis is that developmental changes in the imbalance between sensation seeking and self-regulation leads to changes in the probability of risk behaviors. Considering this argument in Dual Systems models, we view modeling the *development* of the imbalance as a critical component of conducting more precise tests of the imbalance hypothesis.

### 1.6 Longitudinal approaches to modeling the imbalance

Next, we discuss and illustrate two flexible data analytic strategies that provide more precise tests of the tenets of Dual Systems models that the imbalance between sensation seeking and self-regulation, and the development of the imbalance over the course of adolescence, should be associated with risk behaviors. After discussing the conceptual and statistical benefits of Latent Difference Score and Growth Mixture Modeling, we provide examples in which we use these methods to assess the relationship of the sensation seeking – self-regulation imbalance with risk behavior using a large longitudinal community sample. The worked examples focus on modeling the imbalance with separate measures of sensation seeking and self-regulation. For researchers taking the perspective that acting without thinking (impulse control) reflects a direct measure of the imbalance (Khurana et al., 2018), we note how Latent Difference Score and Growth Mixture Modeling approaches can be used with a single indicator of the imbalance. We hope to stimulate the field to reflect on the statistical complexity of modeling the imbalance and to explicitly discuss which data analytic strategies provide the best approach to modeling the imbalance and its relationship with risk behavior.

#### 1.6.1 A latent difference score approach

As noted above, difference scores provide a straightforward operationalization of the imbalance that can serve as the predictor of risk behavior (Vazsonyi and Ksinan, 2017), but have several statistical limitations. Latent Difference Score (LDS) models are an alternative approach to observed difference scores that maintain the theoretical appeal of assessing the imbalance with a difference score, while mitigating concerns about unreliability (Kievit et al., 2018; Ferrer and McArdle, 2010; McArdle, 2001, 2009). LDS models impose strict parameter constraints that permit modeling of latent changes that represent the difference of two latent variables (see de Haan et al., 2017). A significant benefit of LDS modeling is that a growth curve can be fit to the difference scores (McArdle, 2009), allowing researchers to examine the developmental changes in the imbalance (Kievit et al., 2018), as well as the association between growth in imbalance and risk behaviors. Thus, LDS models are particularly well-suited to test the imbalance hypothesis because they (1) explicitly model the imbalance using latent variables^1^, (2) allow for the examination of developmental changes in the imbalance, and (3) allow researchers to assess how growth in the imbalance is related to risk behaviors.

Despite these benefits, there are also several limitations of LDS models. First, it requires researchers to have three waves of data to estimate the growth portion of the model. Because the growth portion of the model is specified as a latent growth curve (see LDS example for greater detail), factors that typically influence performance of latent growth curve models (e.g., misspecification of the growth model, missing data patterns, and individual differences in measurement occasion time points) also impact performance of the LDS growth model (Curran et al., 2010; Miller and Ferrer, 2017; Muthén et al., 2011). The LDS approach also requires the use of large sample sizes (Grimm et al., 2012), which is not always feasible, particularly if constructs are being measured at the neurobiological level. Third, due to the specification of the latent difference scores, it is not possible to examine whether the imbalance accounts for unique variance in risk behaviors above and beyond either sensation seeking or self-regulation.

Finally, this modeling approach does not address how to create a meaningful difference across two variables on separate metrics (Cronbach and Furby, 1970). As noted earlier, this commonly occurs with behavioral indicators of self-regulation and sensation seeking. One solution is to standardize the indicators, as per Vazsonyi and Ksinan (2017); however, simple standardization will not work with longitudinal data because standardizing indicators at each assessment removes information about the means (means are 0 for standardized variables), which is crucial for understanding growth. An alternative is to collapse data across repeated measures and standardize (i.e., include all participants’ data from all waves of data collection onto the same distribution before standardizing; see Fosco et al. (2015) for an example of this standardization approach). This method puts each indicator on the same metric and retains information about growth. This method also provides a meaningful value for the intercept in a latent growth curve model when examining growth in the imbalance across time. That is, with standardized difference scores, the latent intercept represents agreement between sensation seeking and self-regulation (e.g., score below zero indicate an imbalance where inhibitory control is greater than sensitivity to reward, scores of zero indicate no imbalance between sensitivity to reward and inhibitory control, and scores greater than zero indicate an imbalance where sensitivity to reward is higher than inhibitory control) at the age where the intercept is specified. However, this approach does have limitations because the value of the difference score is relative and sample-dependent, making comparisons across samples difficult.

In our empirical example we use LDS modeling to demonstrate how this standardization approach can be used to model the relationship of the imbalance with risk behaviors, specifically the probability of alcohol and marijuana use, as well as levels or intensity of alcohol use and the frequency of marijuana use from early through late adolescence (ages 13–20). Our LDS model allowed us to assess the following questions:

1 Is there significant growth and individual variability in growth of the imbalance across ages 12–14?
2 Are initial levels of the imbalance or growth in the imbalance associated with alcohol and marijuana use?

#### 1.6.2 A growth mixture modeling approach

Growth Mixture Modeling (GMM) is a statistical technique that can be used to identify classes or groups of individuals that share similar patterns of growth in sensation seeking and self-regulation over time (for detailed descriptions of GMM as well as applied papers using GMM see Colder et al., 2001; Muthén and Muthén, 2000; Ram and Grimm, 2009). GMM is related to latent growth curve modeling, although it relaxes the assumption of a latent growth curve model that all individuals are drawn from a single population (Muthén and Muthén, 2000). Applied to Dual Systems models, one might expect a majority of adolescents to be characterized by rapidly-developing sensation seeking and slower-developing self-regulation (Fig. 1 Panel A), and this pattern would be expected to be associated with frequent engagement in risk behaviors (Steinberg, 2008). However, other patterns of growth in both sensation seeking and self-regulation for adolescents likely exist, and of interest is how different patterns of growth in sensation seeking and self-regulation and the imbalance they imply are related to risk behaviors (Crone et al., 2016; Khurana et al., 2018). For example, one might also expect a class that shows gradual increases in both sensation seeking and self-regulation, and hence a relatively modest imbalance and low levels of risk behavior.

Although no studies, to our knowledge, have used GMM to assess the imbalance hypothesis using separate measures of sensation seeking and self-regulation, Khurana et al. (2018) applied GMM to a measure of impulsivity, which they posit reflects the imbalance (acting without thinking). Invoking the Lifespan Wisdom Model (Romer et al., 2017), Khurana et al. (2018) hypothesized that only a subset of adolescents would exhibit an imbalance across adolescence and found evidence for two distinct subgroups of trajectories of acting without thinking across adolescence, a low stable trajectory and a high increasing trajectory. Adolescents in the high increasing trajectory had higher levels of Substance Use Disorder severity relative to adolescents in the low stable trajectory. This study highlights the appeal of GMM by demonstrating the ability of this modeling approach to identify distinct developmental patterns of the imbalance, which has been increasingly advocated for in studies of the imbalance (Crone et al., 2016; Romer et al., 2017).

It is important to differentiate GMM from parallel process growth models. GMM simultaneously estimates multiple growth curves, such as sensation seeking and self-regulation, and allows researchers to identify different groups or classes of individuals that share similar trajectories of both sensation seeking and self-regulation. Parallel process growth models allow one to model growth in multiple constructs, such as sensation seeking and self-regulation, and to estimate associations between the growth curves. For example, Shulman et al. (2016b) used a parallel process growth model to estimate trajectories of impulse control and sensation seeking, and found that both increased over time during adolescence, that increases in impulse control and sensation seeking were independent of each other. While parallel process models provide descriptive information regarding growth in sensation seeking and self-regulation, they do not provide much information about imbalance. A unique advantage of GMM is that it allows researchers to identify different patterns of growth in sensation seeking and self-regulation, and thereby different patterns of the imbalance (Khurana et al., 2018). This feature of GMM aligns with recent calls for research on Dual Systems models, and adolescent development more broadly, to account for the heterogeneity in developmental changes which may help identify adolescents at greatest risk to engage in risk behaviors (Crone et al., 2016; Khurana et al., 2018; Lanza and Cooper, 2016; Lydon-Staley and Bassett, 2018).

Yet, GMM also has limitations. The reliability and validity of classes obtained in GMM has been questioned (Bauer and Curran, 2003; Nylund, 2007). Researchers have called for the use of validity analyses, such as creating a nomological network, to provide support for obtained class structures (Nylund, 2007). Further, replication of class structure is important across multiple samples to provide evidence that growth patterns of sensation seeking and self-regulation and observed patterns of imbalance are not sample specific (Wright and Hallquist, 2014). In addition to concerns pertaining to reliability and validity, growth mixture models can be difficult to estimate and often result in underidentified or inadmissible solutions, especially as more parameters are allowed to vary across classes (Wang et al., 2005; Wright and Hallquist, 2014). Acknowledging the difficulties in estimating GMM, Wright and Hallquist (2014) recommend a sequential approach to GMM that balances accounting for within-class heterogeneity with the pragmatics of model estimation. This approach is used in our GMM example for the current integrative review. As with the LDS approach, another limitation of this method is that it requires large sample sizes (Nylund et al., 2007).

In our example using GMM, we demonstrate how this approach can be used to model trajectories of growth in sensation seeking and self-regulation and assess the relationship of the growth trajectories to the probability of alcohol and marijuana use and levels or intensity of alcohol and marijuana use from early through late adolescence. Importantly, as with the LDS approach, measures of sensation seeking and self-regulation will need to be on the same metric in order to make the imbalance between trajectories of sensation seeking and self-regulation interpretable. Once sensation seeking and self-regulation are on the same metric, GMM allowed us to assess the following questions:

1 How do sensation seeking and self-regulation develop across ages 12–14?
  a. What are the different prototypical patterns of growth for sensation seeking and self-regulation across ages 12–14?
2 Are different patterns of growth of sensation seeking and self-regulation associated with alcohol and marijuana use?

#### 1.6.3 Moderation

A particularly appealing feature of GMM is that it permits assessment of moderation. This is important, as most developmental models of risk behavior emphasize context as an important factor that interacts with individual differences (Chein et al., 2011; Crone and Dahl, 2012; Shulman et al., 2016a). Although moderation of dichotomous variables in univariate LDS models is possible, the use of continuous moderators is still being developed (see O’Rourke et al., 2019). The LDS growth model proposed in the current integrative review deviates from a typical LDS model in that the difference scores are a function of two variables and a latent growth curve is fit to the difference scores at each age. A benefit of this LDS growth model is that the specification of a latent growth curve permits using methods of assessing moderation of the association between latent slopes and intercepts and some outcome (see Preacher et al., 2006). For example, it is possible for researchers to examine how other variables (such as peer delinquency and parental monitoring) might moderate the association between growth in the imbalance, in an LDS model, as well as classes representing different patterns of imbalance, in a GMM, and risk behaviors.

## 2 Materials and methods

### 2.1 Participants

To illustrate these proposed data analytic approaches to assessing the imbalance hypothesis with longitudinal data, we used data from a community sample of 387 families (1 adolescent and 1 caregiver from western New York state) assessed annually for 9 years. The study examined risk and protective factors associated with the initiation and escalation of early adolescent substance use. The sample was evenly split on sex (55% female) and was predominantly non-Hispanic Caucasian (83.1%) and African American (9.1%). Median family income was $70,000 and ranged from $1500 to $500,000, and 6.2% of the families received public income assistance. The demographic characteristics of our community sample are similar to those from whence the sample came (for more complete details, see Trucco et al., 2014).

Participants had an average age of 12.1, 13.1, 14.1, 15.1, 16.1, 17.1, 18.4, 19.4, and 20.4 (*SD* range = 0.59 to 0.67) at waves (W) 1 to W9, respectively. The sample included 387, 373, 370, 368, 361, 349, 352, 349, and 350 adolescents at W1 to W9, respectively. Overall attrition across W1 through W9 was low (9.6%).

### 2.2 Procedures

For W1 to W3, adolescents and their parents were interviewed in university research offices. Informed consent and assent procedures were completed before the interviews began. Target families were compensated for their participation. W4 to W6 consisted of a brief telephone-based audio-computer-assisted self-interviewing (CASI) survey of substance use that took 10 to 15 min to complete. Parents provided consent over the phone and were given a phone number and PIN for their adolescent to use. Assent from the adolescent was obtained at the initiation of the audio-CASI survey. Procedures at W7 to W9 mirrored those of W1 to W3; however, adolescents and caregivers were provided with the option to complete the questionnaires online. Indicators of sensation seeking and self-regulation in our study were measured from W1 to W3, and substance use was assessed from W1 to W9^2^.

### 2.3 Measures

#### 2.3.1 Inhibitory control (W1-W3)

The current study assessed inhibitory control as the behavioral indicator of the self-regulation system (see Nigg, 2017). The Stop Signal Task (SST; Logan et al., 1984) is among the most commonly used task of inhibitory control; the SST assesses participants’ abilities to inhibit a dominant response through the use of two concurrent tasks – a go task and a stop task. The integration method was used to compute the stop signal reaction time (SSRT) for each test block and then averaged across blocks (Logan et al., 1984; Verbruggen et al., 2013). To make it easier to interpret this measure, SSRT values were multiplied by a constant (-1) so higher values reflected stronger response inhibition. Cronbach’s alpha for inhibitory control for the current sample computed using the SSRT from each of the three experimental blocks was acceptable (α range = 0.66-0.73). Further, in order for inhibitory control to be on the same metric as sensation seeking, SSRT was collapsed across waves and participants and then standardized. Previous work with this sample has shown that higher SSRT (i.e., worse inhibitory control), in the context of high reward sensitivity, prospectively predicts delinquent behavior in adolescence (Rhodes et al., 2013) and that initial levels of SSRT at age 11 prospectively predicts delinquent behavior in late adolescence (Fosco, 2017).

#### 2.3.2 Sensitivity to reward (W1-W3*)*

The Point Score Reaction Time Task for Children-Revised (PSRTT-CR; Colder et al., 2011) was used to assess sensitivity to reward as the behavioral indicator of the socioemotional system.^3^ This task starts with a practice block followed by four experimental blocks presented in a fixed order: *no reward*, *reward*, *punishment*, and *post-punishment*. Of interest in this task was the degree to which reaction times declined (i.e., got faster) during the *reward* compared to the *no reward* block (sensitivity to reward). Higher values on this task represented greater sensitivity to reward. In order for reward sensitivity to be on the same metric as inhibitory control, reward sensitivity was standardized across ages 12–14. Prior work with this sample has found faster growth in reward sensitivity, as measured on the PSRT, to be associated with more rapid escalation of substance use (Colder et al., 2013). Reward sensitivity measured using the PSRT was also found be associated with parent report of reward sensitivity and physiological reactivity to reward (Colder et al., 2011). Cronbach’s alpha for sensitivity to reward was adequate (α range = .73–.79) and computed by dividing the PSRT into three equal blocks at each age.

#### 2.3.3 Substance use (W1-W9)

Alcohol use was assessed across W1 to W9 with questions assessing past year frequency of alcohol use, as well as past year typical quantity of alcohol use during a drinking occasion without parental permission. Indicators of past year frequency and quantity of alcohol use were multiplied to get an index of number of drinks consumed in the past year. Past year marijuana frequency was assessed at W1 to W9.

## 3 Results

### 3.1 Latent difference score growth model approach

**Fig. 2:**
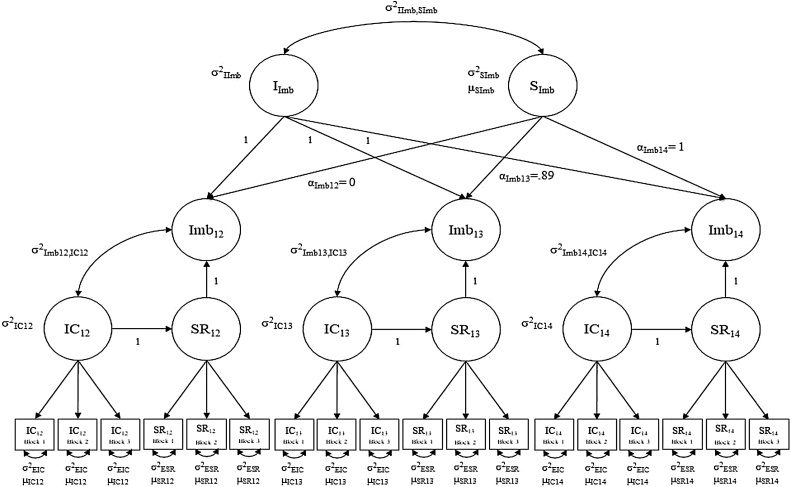
IC = inhibitory control, SR = sensitivity to reward, Imb = the difference (imbalance) between sensitivity to reward and inhibitory control, I = intercept, and S = slope.

A detailed account of the fitting of both the LDS growth model and our two-part growth models with random effects for alcohol use as well as Mplus output files can be found in the Supplemental Materials. As noted in the methods section, in order to create a meaningful difference, inhibitory control and sensitivity to reward were standardized to place them on the same metric (Edwards, 1994). Descriptive information regarding inhibitory control, sensitivity to reward, and alcohol and marijuana use can be found in Table 2. Analyses were conducted using Mplus version 8.2 using maximum likelihood estimation with robust standard errors and numerical integration to fit robust chi-square and standard error estimates (Muthén & Muthén, 1998–2017). Full information maximum likelihood estimation (FIML) was used in Mplus to handle missing data. Specification of our LDS model can be seen in Fig. 2.

**Table 2:** Mean values for inhibitory control, sensitivity to reward, and alcohol and marijuana use from ages 12 to 20.

	12	13	14	15	16	17	18	19	20
Inhibitory Control (raw)	162.96	133.99	127.04	–	–	–	–	–	–
Inhibitory Control (STD)	−0.17	0.23	0.32	–	–	–	–	–	–
Reward (raw)	683.32	589.87	545.76	–	–	–	–	–	–
No Reward (raw)	793.85	683.75	647.76	–	–	–	–	–	–
Sensitivity to Reward (STD)	0.62	0.53	0.58	–	–	–	–	–	–
Alcohol (% past year users)	7%	13%	22%	35%	43%	52%	64%	72%	76%
Alcohol (QxF)	0.06	0.61	2.00	5.97	12.08	48.61	163.16	184.95	212.86
Marijuana (% past year users)	1%	3%	9%	14%	19%	30%	48%	47%	50%
Marijuana (F)	0.03	0.12	0.71	4.38	6.69	21.81	46.48	54.48	64.81

*Note.* Raw = raw metric of the tasks (milliseconds), STD = standardized, QxF = quantity by frequency, F = frequency.

As outlined by de Haan et al. (2017), the first step when estimating an LDS model with separate constructs is to assess for measurement invariance. Longitudinal measurement invariance was tested separately for inhibitory control and sensitivity to reward using the three blocks of the stop signal task as indicators of latent inhibitory control at ages 12, 13, and 14 and trials of the PSRT were broken into three equal blocks and used as indicators of latent sensitivity to reward. Partial residual invariance was supported for both inhibitory control (*χ^2^* = 46.18(33), *p* = .06, CFI=.98, TLI=.98, RMSEA=.03, SRMR=.05) and sensitivity to reward (*χ^2^* = 36.73(35), *p* = .38, CFI=.99, TLI=.99, RMSEA=.01, SRMR=.04). After establishing partial longitudinal measurement invariance for inhibitory control and sensitivity to reward, latent difference scores were specified to represent the imbalance between inhibitory control and sensitivity to reward at ages 12, 13, and 14 (de Haan et al., 2017).

As seen in Fig. 2, Imb_12_ is a LDS that mathematically represents an adolescent’s latent score on sensitivity to reward at age 12 minus their latent score on inhibitory control at age 12 (the imbalance). Thus, Imb_12_, as well as Imb_13_ and Imb_14_, represent the imbalance, accounting for measurement error. The LDS model provides information regarding adolescents’ imbalance at a static point in time.

Because a central tenet of the imbalance hypothesis pertains to the development of the imbalance across adolescence, we extended the LDS model to include growth in the latent imbalance difference scores^4^, which we refer to as the LDS growth model. As seen in Fig. 2, a latent growth curve was fit to the latent imbalance difference scores at ages 12, 13, and 14. Through the specification of a latent intercept and slope, this LDS growth model provides information regarding adolescents’ initial levels of the imbalance and permits examination of whether there is significant variability in the imbalance at age 12 (σ^2^I_Imb_). The model also includes a latent slope (S_Imb_) representing growth in the imbalance across ages 12 to 14 and whether there is significant variability in growth of the imbalance from ages 12 to 14 (σ^2^S_Imb_). Through the estimation of a latent intercept and slope of the imbalance, researchers can then examine whether initial levels of the imbalance (in our example age 12) are associated with risk behaviors, and whether growth in the imbalance across adolescence (in our example growth from ages 12 to 14) is associated with risk behaviors.

The LDS growth model provided an adequate fit to the data (*χ^2^* = 266.22(148), *p* < .001, CFI=.92, TLI=.91, RMSEA=.04, SRMR=.12). Linear growth in the latent differences at ages 12, 13, and 14 was compared to a model where the loading of the growth curve at age 13 was freely estimated. Freeing the loading at age 13 led to a significant improvement in model fit. The slope mean of -0.47 (p<.001) indicated that the difference between sensitivity to reward and inhibitory control decreased with age. There was also evidence of significant variability in the intercept (σ^2^ = 0.25, p < .001) and slope (σ^2^ = 0.18, p < .001) suggesting that initial levels of the imbalance and growth in the imbalance varied significantly across individual adolescents. The covariance between the intercept and slope of the imbalance was also significant (σ^2^=-0.18, p < .001), indicating that higher levels of an imbalance at age 12 was associated with quicker decreases in the imbalance across ages 12–14. Mplus syntax output files for our LDS growth model can be found in Supplemental Materials 2.

**Fig. 3:**
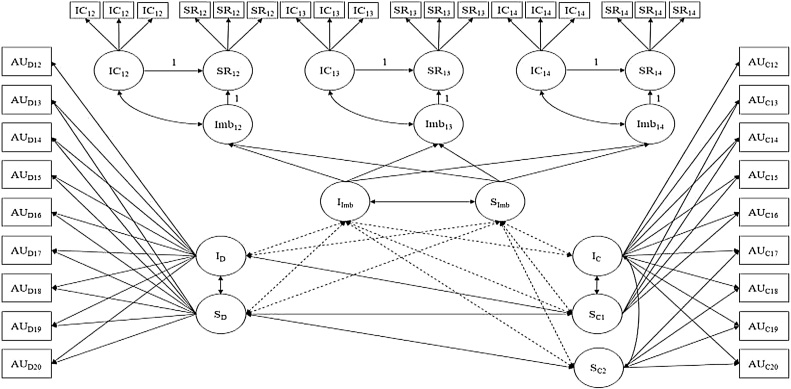
LDS approach to assessing the relationship between the imbalance and the probability of alcohol use and growth in alcohol use across ages 12–20. IC = inhibitory control, SR = sensitivity to reward, Imb = the latent difference (imbalance) between sensitivity to reward and inhibitory control, AU = alcohol use, D = dichotomous use (use vs. no use), C = continuous levels (quantity x frequency) of past year use, I = intercept, and S = slope. Solid two-headed arrows depict significant covariances. Dashed two-headed arrows depict non-significant estimated covariances between the imbalance and alcohol use.

Next, we assessed whether the intercept and slope terms from our LDS growth model covaried with the intercept and slope factors from a two-part growth model for both alcohol and marijuana use that spanned ages 13–20 (see Fig. 3). Two-part models simultaneously model dichotomous past year use (yes/no) as well as continuous levels of past year use (quantity x frequency of use for alcohol and frequency for marijuana). This modeling framework provides an analytic approach to account for the large proportion of zeros often observed when studying risk behaviors (Olsen and Schafer, 2001) and distinguishes probability of use from intensity or levels of use. Hence, this modeling approach allows for the examination of whether initial levels of the imbalance, as well as growth in the imbalance, were associated with (1) the initial probability of alcohol and marijuana use at age 13, (2) growth in the probability of alcohol use across ages 13 to 20, (3) initial levels of alcohol and marijuana use at age 13, and (4) growth in levels of alcohol and marijuana use across ages 12 to 20 and 13 to 20, respectively. For information regarding model specification and fitting of the two-part growth models with random-effects see Supplemental Materials 1 and 2.

For our alcohol use two-part growth curve model with random effects, the intercept for the probability of alcohol use, as well as levels of alcohol use, were set to age 13 due to the small number of users at age 12 (N = 15) (see Table 1). A piecewise model provided the best fit for modeling growth in the continuous portion of the model. The first slope represents change from ages 12 to 16 and the second slope represents change from ages 16 to 20. The means for the dichotomous and continuous slopes were all positive and significant indicating significant increases in the probability and levels of alcohol use from ages 12 to 20. The intercept for the dichotomous portion, intercept for the continuous portion of the model, and all slopes for the dichotomous and continuous portions of the model had statistically significant variability. This suggests individual variability in growth in alcohol use.

Non-linear slope factors provided the best fit for the dichotomous and continuous portions of the marijuana two-part growth model. The slope mean for both the dichotomous portion and continuous portions of the marijuana model were statistically significant and indicated significant increases in the probability of marijuana use and frequency of use from ages 13 to 20. Further, there was significant variability in the intercept of the dichotomous portion of the model and all slopes had significant variability, again suggesting individual variability in growth.

Next, the LDS growth model and two-part models for alcohol and marijuana use were combined to assess the relationship between the imbalance and substance use. Cohen’s (1992) power tables indicated that these models had sufficient power to detect a small to medium sized effect between the intercept and slope of the imbalance and growth in alcohol and marijuana use. For both the alcohol and marijuana use models, there were no significant covariances between the intercept and slope of the imbalance and the intercepts and slopes of alcohol and marijuana use. This suggests that the magnitude of the imbalance in early adolescence and growth in the imbalance across early-to-middle adolescence was unrelated to substance use in early-to-late adolescence.

### 3.2 Growth mixture modeling approach

GMM is an analytic technique that allowed us to simultaneously model growth in sensation seeking and self-regulation and to identify classes or groups that shared similar patterns of growth. The classes provide a description of different developmental patterns of imbalance. Detailed information regarding specification of our GMM and determination of class structure can be found in the Supplemental Materials. Analyses were conducted using Mplus version 8 (Muthén and Muthén, 1998–2017). The three standardized sensation seeking and inhibitory control blocks were averaged at ages 12, 13, and 14, respectively, and used for these analyses using the same standardization procedure discussed above. A sequential process was used to estimate our GMM, where univariate growth models were first estimated for inhibitory control and sensitivity to reward (Wright and Hallquist, 2014). Next, a series of growth mixture models were estimated: (1) GMM with unique means (also known as the Latent Class Growth Model) where means of the intercepts and slopes of inhibitory control and sensitivity to reward are allowed to vary freely across classes and variances and covariances of the growth factors are fixed to zero, (2) GMM with unique means and shared variance where means are once again allowed to vary across classes and variances and covariances are estimated for the growth factors but constrained to be equal across class solutions, and (3) and the GMM with unique means and variances where both means and variances are allowed to freely vary across classes.

A growth model where the slope loading for inhibitory control at age 13 was freely estimated provided the best fit to the data (χ^2^(0) = 0.00, p = 1.00, CFI = 1, TLI = 1., RMSEA = 0.00, SRMR = .01). The slope mean was significant (M = 0.52, p < .001) indicating significant growth in inhibitory control from ages 12 to 14. The variance of the intercept of inhibitory control at age 12 (σ^2^ = 0.37, p < .001) was statistically significant, indicating significant individual differences in initial levels of inhibitory control. The slope variance for inhibitory control was not statistically significant (σ^2^ = 0.18, p = .40) limited variability in change in inhibitory control from ages 12 to 14.

When modeling growth in sensitivity to reward, the residual variance for age 12 sensitivity to reward was initially estimated to be negative and was constrained to 0. A model where age 13 sensitivity to reward was freely estimated provided the best fit to the data (χ^2^(2) = 3.33, p = .18, CFI = .98, TLI = .98, RMSEA = 0.04, SRMR = .05). The slope mean was significant (M= -0.09, p = .01), indicating significant declines in sensitivity to reward from ages 12 to 14. The variance of the intercept of sensitivity to reward at age 12 (σ^2^ = 0.41, p < .001) was statistically significant, indicating significant individual differences in initial levels of sensitivity to reward. The variance of the slope for sensitivity to reward was also significant (σ^2^ = 0.34, p < .001), indicating significant individual differences in declines in sensitivity to reward from ages 12 to 14.

The Akaike information criterion (AIC), Bayesian information criterion (BIC), sample size adjusted BIC, entropy, class size, and bootstrapped likelihood ratio test (BLRT) were all used to determine the number of classes to extract (Nylund et al., 2007). Relative to a single class solution, a two class solution is supported by lower information criteria and a significant BLRT. These fit criteria were compared both within growth mixture modeling strategies (e.g., comparing fit criteria in the two and three class solutions for the GMM with unique means and shared variances) as well as across growth mixture modeling strategies (e.g., comparing fit criteria for the best class solution for the GMM with unique means to the best fitting class solution for the GMM with unique means and variances) to determine the final class solution.

The two class GMM with unique means and shared variances was selected as the final class solution. Information criteria and the BLRT suggested extracting more than 2 classes; however, we elected to retain a 2-class solution considering there was an extremely small class in the 3 class solution (N = 8, 2% of sample). The GMM with unique means and shared variances had a lower AIC, BIC, and aBIC than the best GMM with unique means solution and a higher entropy than the GMM with unique means and variances (see Supplemental Materials 1 for greater detail and Mplus output files). GMM with unique means and shared variances constrains variances and covariances to be equal across classes while allowing for unique mean patterns of growth across classes. The benefit of this GMM approach is that it allows for variability in growth parameters while being more stable than models with unique variance estimates in each class (Wright and Hallquist, 2014).

**Fig. 4:**
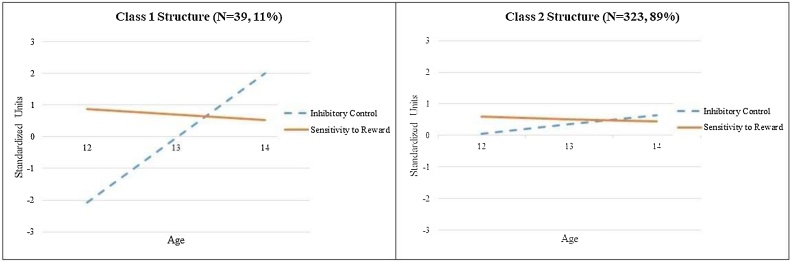
Final two class solution for the growth mixture model with unique means and shared variances.

Developmental patterns of the two classes can be found in Fig. 4. The intercept of inhibitory control (σ^2^ = 0.37, p < .001), slope of inhibitory control (σ^2^ = 0.33, p < .001), intercept of sensitivity to reward (σ^2^ = 0.40, p < .001), and growth in sensitivity to reward (σ^2^ = 0.34, p < .001) all had significant variability in the two class GMM with unique means and shared variances. Class 1, which consisted of 39 adolescents (11%), was characterized by a larger imbalance at ages 12 and 13 where sensitivity to reward was greater than inhibitory control and inhibitory control became greater than sensitivity to reward at age 14. Class 2 consisted of 323 adolescents (89%) and was characterized by a smaller imbalance, relative to Class 1, at ages 12–13 where sensitivity to reward was higher than inhibitory control, but then inhibitory control was greater than sensitivity to reward at age 14. The patterns of growth found in both Class 1 and Class 2 lend partial support to the imbalance hypothesis that adolescents’ sensitivity to reward was higher than their inhibitory control at ages 12 and 13. However, contrary to the tenet of imbalance hypothesis that argues that the imbalance should be largest in middle adolescence, both Class 1 and Class 2 found higher levels of inhibitory control relative to sensitivity to reward at age 14.

Next, we created a categorical variable representing class membership by assigning adolescents to their most likely class using class probabilities. Using the two-part growth models as described above, the probability of alcohol and marijuana use, as well as levels of alcohol and marijuana use, were regressed on the categorical class membership variable. Cohen’s (1992) power tables indicated that these models had sufficient power to detect a small to medium sized effect between class membership and growth in alcohol and marijuana use. No significant associations were found between class membership and either the probability of alcohol or marijuana use or levels of alcohol or marijuana use, suggesting that the different patterns of imbalance were not associated with growth in substance use.

## 4 Discussion

Dual Systems models are popular and influential theoretical accounts of adolescent risk behavior (Steinberg, 2017). As the field accumulates longitudinal data of the socioemotional and self-regulation systems at both neural and behavioral levels of analysis, there will be exciting opportunities to increase our understanding of the etiology and impact of risk behaviors, such as substance use. In line with recent calls to improve specificity of prediction models (Pfeifer and Allen, 2016), the current paper sought to highlight the need for more rigorous data analytic methods to test the imbalance hypothesis forwarded by Dual Systems models. We have argued that statistical techniques such as Latent Difference Score (LDS) and Growth Mixture Models (GMM) provide more precise and theoretically-consistent tests of the imbalance hypothesis than other approaches that have been used in the literature thus far, such as simple observed difference scores and regression approaches that test multiplicative interactions.

We reiterate that our goal of the current paper was not to provide support for or against Dual Systems Models. Our goal was to bring attention to data analytic methods that more appropriately quantify the imbalance than previously-used methods. Nevertheless, our empirical examples did not demonstrate expected relationships between the imbalance and risk behavior. Although our measures do not perfectly reflect the socioemotional and cognitive control systems (Harden et al., 2017), these findings are not due simply to measurement concerns. Indeed, using the same behavioral tasks from the current study, we have previously demonstrated that increases in reward sensitivity predict increases in substance use (Colder et al., 2013), and that poor self-regulation in the context of high sensitivity to reward predicts rule-breaking behavior (Rhodes et al., 2013). These two studies utilized data analytic methods most akin to the regression and moderation approaches discussed earlier. Taken together, our findings tentatively suggest that data analytic methods that do not adequately quantify the imbalance can be misconstrued as support for the imbalance hypothesis, whereas methods that are more appropriate for measuring the imbalance do not. As more large-scale, longitudinal studies collect data that will be suitable to the LDS and GMM approaches discussed herein, we look forward to seeing the extent to which this pattern is replicated with other samples.

### 4.1 Summary of LDS growth model approach

The LDS modeling approach allowed us to assess a number of important tenets of Dual Systems models. First, LDS modeling allowed for a more reliable estimate of the difference between our measures of sensation seeking and self-regulation than could be accomplished with observed difference scores. Second, it allowed for examining change in the imbalance across ages 12 to 14. Another advantage of this modeling approach was that it allowed for the examination of whether initial levels of the imbalance at age 12, as well as growth in the imbalance from ages 12 to 14, were associated with risk behaviors.

Of note, a potential conceptual limitation of the LDS growth model is that it does not provide information regarding what is leading to the imbalance. This is particularly problematic for studies interested in understanding the factors, such as substance use, that are thought to alter socioemotional and cognitive control systems (Brown et al., 2000; Koob and Le Moal, 2008; Squeglia et al., 2009). For example, growth in the imbalance across adolescence could be a function of increasing sensation seeking and stable self-regulation, rapidly increasing sensation seeking and slowly increasing self-regulation, or stable sensation seeking and decreasing self-regulation. The LDS growth model does not provide information that would distinguish these multiple possibilities for growth in the imbalance across adolescence.

### 4.2 Summary of GMM approach

GMM provides a flexible statistical approach to model patterns of development in sensation seeking and self-regulation. Similar to the LDS method, this approach allows for heterogeneity in growth patterns. Although the LDS growth model and GMM approaches both account for *within*-person change, GMM is a person-centered analytic technique that allows for the examination of different patterns or classes of growth in sensation seeking and self-regulation. The classes can provide a description of the different magnitudes and developmental patterns of imbalance, and how distinct growth patterns may be related to risk behavior. This feature of GMM is consistent with current conceptualizations of adolescent brain development that note that there is heterogeneity across adolescents in their patterns of growth in sensation seeking and self-regulation (e.g., Khurana et al., 2018).

### 4.3 Relevance to neuroimaging data

Although the focus of the current integrative review was on testing the imbalance hypothesis with behavioral data, we see no reason why the data analytic approaches we discussed could not be extended to certain types of neuroimaging data. LDS and GMM approaches could be applied to neuroimaging data that include indicators of both the socioemotional and cognitive control systems (e.g., striatum activation during a rewarding tasking and lPFC activation during an emotionally-salient task). The increased sophistication of the LDS and GMM approaches relative to past methods of assessing the imbalance, in conjunction with their ability to model both behavioral and neuroimaging data, may be helpful in the field’s efforts to more rigorously test the imbalance hypothesis and determine whether findings are consistent across methods.

Further, the ability of the LDS and GMM approaches to model neuroimaging data allows for the examination of competing views of how sensitivity to reward and cognitive control contribute to risk behavior during adolescence. Crone et al. (2016) have suggested that examining connectivity between the socioemotional and cognitive control systems may provide important insight into Dual Systems models over examining solely the imbalance hypothesis. Indeed, studies examining connectivity between the socioemotional and cognitive control systems have informed our understanding of how the socioemotional and cognitive control systems are connected, and how differences in their connectivity across adolescence is related to risk behavior (e.g. Peters et al., 2015; van Duijvenvoorde et al., 2016). Using the LDS approach or GMM approach to model the imbalance, future work could examine whether growth in the imbalance, as forwarded by the imbalance hypothesis, or growth in functional connectivity of the socioemotional and cognitive control systems across adolescence, is more strongly related to risk behavior. This example highlights how improving the specificity and precision of modeling the imbalance can help facilitate the generation of riskier prediction models and allow for testing competing models.

### 4.4 Conclusion

The maturational imbalance hypothesis is a cornerstone of Dual Systems models, which have shaped conceptualizations of adolescent risk behavior and had a substantial impact on public policy to address adolescent delinquency (Casey et al., 2017; Steinberg, 2017). Although the maturational imbalance hypothesis has been taken as having garnered a lot of support (Shulman et al., 2016a), we have argued that this tenet of Dual Systems models has not been rigorously tested. Commonly-used methods to date, such as the regression approach, moderation approach, and observed difference score approach each fail to adequately test this tenet of Dual Systems models. We proposed two promising techniques to assess the maturational imbalance hypothesis, a Latent Change Score approach and a Growth Mixture Modeling approach, which we argued provide a more rigorous evaluation of this hypothesis. We acknowledge that these are not the only longitudinal data analytic methods that can be used to assess the imbalance with longitudinal data (e.g., multilevel modeling). However, we view these methods as making significant improvements over techniques used to date. We hope this integrative review pushes the field to wrestle with the question of how best to assess the imbalance and its relation to risk behavior with longitudinal data. Doing so will help to refine and constrain theory and advance our understanding of adolescent development.

## Funding

This research was supported by a grant from the National Institute on Drug Abuse (R01DA019631) awarded to Craig R. Colder and a grant from the National Institute of Alcohol Abuse and Alcoholism (F31AA025521) awarded to Samuel N. Meisel.

## Declaration of Competing Interest

The authors declare that they have no known competing financial interests or personal relationships that could have appeared to influence the work reported in this paper.

## Footnotes

1 The LDS model presented in the current integrative review addresses how to longitudinally model the imbalance (difference) between separate measures of sensation seeking and self-regulation. A univariate LDS model, commonly referred to as a latent change score models (Kievit et al., 2018), is still a useful approach to measure the imbalance for researchers taking the perspective that acting without thinking reflects a direct measure of the imbalance (Khurana et al., 2018).LDS models of a single measure/construct reflecting the imbalance (e.g., acting without thinking) would allow for the modeling of growth in the imbalance across time, the examination of factors that contribute to changes in the imbalance, as well as assessing how growth in the imbalance is prospectively related to risk behaviors. See Kievit et al. (2018) as well as McArdle (2009) for detailed information regarding specifying and interpreting these LDS models.

2 A detailed codebook and description of our measures as well as E-Prime 3.0 software code (Psychology Software Tools, Pittsburgh, PA) for our measures of inhibitory control and sensitivity to reward will be publicly available at ICPSR. Raw substance use data will be publicly available through ICPSR and the raw data from the Stop Signal Task and Point Score Reaction Time Task for Children-Revised are not yet publicly available due to limited resources to prepare these data for public access.

3 Although “reward sensitivity” typically refers to the neurobiological construct, we use the term sensitivity to reward, as this is the term used by the task developers.

4 For alternative approaches to modeling growth in latent difference scores across two constructs see Cόrdova et al. (2016).
