# Supplementary material for: Mind the gap: A review and recommendations for statistically evaluating Dual Systems models of adolescent risk behavior
Source: Dev Cogn Neurosci. 2019 Jul 25;39:100681. doi: 10.1016/j.dcn.2019.100681 (PMC6969358; doi:10.1016/j.dcn.2019.100681)
Supplement: Supplementary file 1 [file mmc1.docx]

**Supplemental Materials: Detailed Results Section**

**Latent Difference Score Growth Modeling Approach**

**Longitudinal Measurement Invariance.** Latent Difference Score (LDS) models decompose continuous variables into two components, the continuous latent “true” score and a residual measurement error term. As outlined by de Haan and colleagues (2018), the first step when estimating an LDS model with separate constructs is to assess for measurement invariance. Testing longitudinal measurement invariance assesses whether the difference score has the same meaning across time (McArdle, 2009). Longitudinal measurement invariance was tested separately for inhibitory control and sensitivity to reward using the three blocks of the stop signal task as indicators of latent inhibitory control at ages 12, 13, and 14 and trials of the PSRT were broken into three equal blocks and used as indicators of latent sensitivity to reward. Constraining factor loadings for inhibitory control across time resulted in a non-significant decrement in fit (*χ^2^*=3.43(4), *p*=.48), and constraining item intercepts to be equal across time resulted in a significant decrement in model fit (*χ^2^*=89.40(6), *p*<.001). Partial scalar invariance was supported after freeing the intercepts for the first block of the stop task at ages 12, 13, and 14 (*χ^2^*=7.73(5), *p*=.05). Partial residual invariance was supported after freeing the residual variances for the first block of the stop task at age 12, second block of the stop task at age 14, and third block of the stop task at age 12 (*χ^2^*=3.71(3), *p*=.29). The final measurement model for inhibitory control provided a good fit to the data (*χ^2^*=46.18(33), *p*=.06, CFI=.98, TLI=.98, RMSEA=.03, SRMR=.05)

Constraining all factor loadings to be equal across time for the PSRT yielded a significant decrement in model fit (*χ^2^*=15.38(4), *p*=.003). Partial metric invariance was supported for the sensitivity to reward after freeing the factor loading for the third block of sensitivity to reward at age 12 (*χ^2^*=4.05(3), *p*=.25). Partial scalar invariance was supported once freeing the intercept for the first block at age 12 (*χ^2^*=3.88(5), *p*=.56). Constraining all residual variances across time resulted in a significant decrement in model fit (*χ^2^*=82.91(6), *p*<.001). Partial residual invariance was supported after freeing the residual variances for the first and third blocks of the PSRT at age 12 (*χ^2^*=1.53(6), *p*=.95). The final measurement model for sensitivity to reward provided a good fit to the data (*χ^2^*=36.73(35), *p*=.38, CFI=.99, TLI=.99, RMSEA=.01, SRMR=.04). Overall, support for partial residual invariance for both inhibitory control and sensitivity to reward indicates that these constructs retain the same meaning across age.

After establishing partial longitudinal measurement invariance for inhibitory control and sensitivity to reward, latent difference scores were specified to represent the imbalance between inhibitory control and sensitivity to reward at ages 12, 13, and 14 (de Hann et al., 2018). The regression pathways from within-age inhibitory control to sensitivity to reward as well as the pathway from the difference score (labeled Imb12, Imb13, and Imb14 in Figure 2) to sensitivity to reward were fixed to 1 in order to meet model identification requirements (McArdle, 2009; McArdle & Grimm, 2010). These parameter constraints allow for the modeling of latent differences that represent inhibitory control subtracted from sensitivity to reward (see McArdle, 2009). As noted in the discussion of difference scores in the introduction, sensitivity to reward and inhibitory control must be on the same metric in order to create a meaningful difference score. This was accomplished by collapsing repeated measures across age/time, separately for sensitivity to reward and inhibitory control, and then standardizing. This put the two variables on the same metric, but retained crucial information about change over time.

There are two components that influence the LDS variables in univariate models, proportional change paths and a growth factor (McArdle, 2009). The first component, autoproportions, or proportional change paths, are regression coefficients of the difference score regressed on its true score at the previous time point. When the model supports these paths, it suggests that standing on variable X at the prior wave influences the rate of change of variable X at the next time point. In the current model where we are examining the difference between two different variables over time, rather than examining changes in the same variable across time, the proportional change path takes on a different conceptual meaning. Here, a significant proportional change path would indicate that an adolescent’s standing on inhibitory control at a specific age predicted the differences between sensitivity to reward and inhibitory control at that age. Considering this path has limited conceptual appeal, proportional change paths were not included in our LDS model. When creating latent differences of two separate constructs, de Hann and colleagues (2018) recommend estimating a covariance, instead of a proportional change regression path, that conceptually represents whether levels of a construct are associated with the difference between that construct and a second construct. Including these covariances in our model was supported by nested model tests and statistically significant (σ^2^=-0.23, p<.001). The significant negative covariance between inhibitory control and the LDS indicate that higher levels of inhibitory control are associated with lower levels of an imbalance between inhibitory control and sensitivity to reward.

The second component, the growth parameter, reflects growth in change in a univariate LDS model. The difference score estimated in the current model is a function of two constructs and thus is parametrized differently than a univariate LDS model. The major difference is that difference scores are connected and influence each other over time through model constraints in a univariate LDS model, whereas the difference scores representing the imbalance in the current model are separate from one another. Because a central tenet of the imbalance hypothesis is that the imbalance between sensation seeking and cognitive control changes across adolescence, a latent growth curve model was fit to the latent difference scores estimated at ages 12, 13, and 14 (see Figure 2). Specifically, the latent difference scores at ages 12, 13, and 14 served as indicators of a latent growth curve model representing change in the imbalance across these ages. Considering the latent difference scores are a function of two constructs and our model specification differs from a typical univariate LDS model, we refer to our extension of LDS models as the LDS growth model.

The LDS growth model includes a slope growth factor with a shaping parameter, α_Gap_, depicted in Figure 1 as latent growth factor S_Imb_. The slope loadings can take many forms, such as a linear trend with loadings fixed to 0, 1, and 2 to represent increases in change scores over time. It is common to test other shaping parameters, including an intercept only model, loadings fixed to 0, 1, and 2, and freely estimating slope loadings. Importantly, the intercepts of the three blocks of inhibitory control were constrained to be equal within and across time and the intercepts of the three blocks of sensitivity to reward were also constrained to be equal within and across time in the LDS growth model for mean structure identification. Nested model tests supported freeing the loading for growth in the imbalance at age 13 (α_Imb12_=0, α_Imb13_=0.89, and α_Imb14_=1). The mean slope was estimated as -0.47 (*SE*=.07, p<.001) and the variance was estimated at .18 (*SE*=.04, p<.001). The significant mean and slope indicate that there was a significant decrease in the imbalance between sensitivity to reward and inhibitory control from ages 12 to 14 and that there was significant variability in growth. The latent intercept I_Imb_, representing individual differences in the imbalance at age 12, was also statistically significant (σ^2^=0.25, p<.001), suggesting that there was significant individual variability in the LDS (imbalance) between our indicators of sensitivity to reward and inhibitory control at age 12. The intercept and slope terms were significantly negatively correlated (*B*=-0.18, *SE*=.04, p<.001), suggesting that a greater imbalance at age 12 was associated with a more rapid decrease in the imbalance between ages 12 to 14.

Lastly, modification indices suggested allowing residual error variances for inhibitory control and sensitivity to reward to covary within and across time, respectively. Adding these covariances led to a significant improvement in model fit. The final LDS model provided an adequate fit to the data (*Χ^2^*=266.22(148), *p*<.001, CFI=.92, TLI=.91, RMSEA=.05, SRMR=.12). Mplus syntax for our LDS growth model can be found in Supplemental Materials 2.

**Growth Mixture Modeling Approach**

A growth mixture modeling approach was utilized to model different patterns of growth in sensitivity to reward and inhibitory control across ages 12 to 14 using Mplus 8.2 (Muthen & Muthen, 1998-2017). A sequential approach proposed by Wright and Hallquist (2014) was used to estimate our growth mixture models. First, latent growth curve models were estimated for sensitivity to reward and inhibitory control. Next, a series of growth mixture models were estimated: (1) GMM with unique means (also known as the Latent Class Growth Model) where means of the intercepts and slopes of inhibitory control and sensitivity to reward are allowed to vary freely across classes and variances and covariances of the growth factors are fixed to zero, (2) GMM with unique means and shared variance where means are once again allowed to vary across classes and variances and covariances are estimated for the growth factors but constrained to be equal across class solutions, and (3) and the GMM with unique means and variances where both means and variances are allowed to freely vary across classes.

A just-identified growth model where the slope loading for inhibitory at age 13 was freely estimated provided the best fit (χ^2^(0)=0.00, p=1.00, CFI=1, TLI=1, RMSEA=.00, SRMR=.00). The slope mean was significant (M=0.52, p<.001) indicating significant growth in inhibitory control from ages 12 to 14. The variance of the intercept of inhibitory control at age 12 (σ^2^=0.37, p<.0001) was statistically significant indicating significant individual differences in initial levels of inhibitory control. The slope variance for inhibitory control was non-significant (σ^2^=0.18, p=.40) indicating non-significant variability in increases in inhibitory control from ages 12 to 14. Initial levels of inhibitory control at age 12 were not significantly associated with growth in inhibitory control from ages 12 to 14 (σ^2^=-0.18, p=.32).

The initial growth model for sensitivity to reward initially produced a negative residual variance for sensitivity to reward at age 12. After constraining this residual variance to 0, the model estimated normally. A model where the slope loading for age 13 sensitivity to reward was freely estimated provided the best fit to the data (χ^2^(1)=3.33, p=.18, CFI=.98, TLI=.98, RMSEA=0.04, SRMR=.03). Sensitivity to reward significantly decreased from ages 12 to 14 (M=-0.09, p=.01). The variance of the intercept of sensitivity to reward at age 12 (σ^2^=0.41, p<.0001) was statistically significant indicating significant individual differences in initial levels of sensitivity to reward. The variance of the slope for sensitivity to reward was also significant (σ^2^=0.34, p<.0001) indicating significant individual differences in declines in sensitivity to reward from ages 12 to 14. Initial levels of sensitivity to reward at age 12 were significantly associated with growth in sensitivity to reward from ages 12 to 14 (σ^2^=-0.33, p<.0001), such that higher initial levels of sensitivity to reward were associated with quicker decreases in sensitivity reward across ages 12 to 14.

The Akaike information criterion (AIC), Bayesian information criterion (BIC), sample size adjusted BIC, entropy, class size, and bootstrapped likelihood ratio test (BLRT) were all used to determine the number of classes to extract (Muthén, 2004; Nylund, Asparouhov, & Muthén, 2007). Relative to a single-class solution, a two-class solution is supported by lower information criteria and a significant BLRT. These fit criteria were compared both within growth mixture modeling strategies (e.g., comparing fit criteria in the two and three class solutions for the GMM with unique means and shared variances) as well as across growth mixture modeling strategies (e.g., comparing fit criteria for the best class solution for the GMM with unique means to the best fitting class solution for the GMM with unique means and variances) to determine the final class solution. Supplemental Materials Table 1 depicts the extraction criteria for 1 to 4 class solutions for growth mixture model with unique means (GMM-UM), growth mixture model with unique means and shared variances (GMM-UMSV), and growth mixture model with unique means and variances (GMM-UMUV). To ensure that class solutions did not reflect local solutions, all class solutions were replicated using double the number of random starts (Hipp & Bauer, 2006).

Results of the GMM-UM indicated that the information criteria and the BLRT suggested extracting more than three classes. However, the four-class solution had a class with 8 individuals (2% of sample), which fell below the recommended 5% of the sample for the smallest class (Nylund et al., 2007). Thus, the three-class GMM-UM was retained as the best solution for this growth mixture model. Next, variances and covariances were freely estimated and constrained to be equal in the GMM-UMSV. The two-class GMM-UMSV produced an error message pertaining to a negative residual variance in inhibitory control at age 12. After constraining this residual variance to zero, all subsequent models estimated without issues. Similar to the GMM-UM, information criteria and the BLRT suggested extracting up to four classes. However, we elected to retain a two-class solution considering the extremely small class sizes in the three-and four-class solutions. Lastly, one through four class solutions were examined in the GMM-UMUV where variances and covariances were allowed to vary across classes. Of note, model estimation difficulties occurred while estimating the GMM-UMUV. When estimating the two class solution, the slope of inhibitory control had a negative variance in class one, the intercept and slope variances of inhibitory control as well as the slope of sensitivity to reward had negative variances in class two. All negative variances had to be constrained to zero in order for the model to estimate. Information criteria and the BLRT suggested extracting up to four classes, however, the fourth class was extremely small (N=6, 1.66%). This led to the three class GMM-UMUV being considered as a final class solution. All classes had poor entropy suggesting that individuals’ membership in the classes had poor certainty.

After comparing class solutions within GMM strategy, solutions were compared across GMM strategies. The two-class GMM-UMSV was retained as the final class solution because it had a lower AIC, BIC, and aBIC, and a higher entropy than the three-class GMM-UM. Relative to the GMM-UMUV, the two-class GMM-UMSV had a higher AIC, BIC, and aBIC than the three-class solution. Despite the improved fit in the GMM-UMUV, this class solution was not selected because entropy was low indicating there was poor certainty in which adolescents belonged to which class.

Developmental patterns of the two classes can be found in Figure 4. The intercept of inhibitory control (σ^2^=0.37, p<.001), slope of inhibitory control (σ^2^=0.33, p<.001), intercept of sensitivity to reward (σ^2^=0.40, p<.001), and growth in sensitivity to reward (σ^2^=0.34, p<.001) all had significant variability in the two class GMM with unique means and shared variances. Class 1, which consisted of 39 adolescents (11%), was characterized by a large imbalance at ages 12 and 13 where sensitivity to reward was greater than inhibitory control and inhibitory control became greater than sensitivity to reward at age 14. Class 2 consisted of 323 adolescents (89%) and was characterized by a smaller imbalance, relative to Class 1, at ages 12 to 13 where sensitivity to reward was higher than inhibitory control, but then inhibitory control was greater than sensitivity to reward at age 14. The patterns of growth found in both Class 1 and Class 2 lend partial support to the imbalance hypothesis such that adolescents’ sensitivity to reward was higher than their inhibitory control at ages 12 and 13. However, contrary to the tenet of imbalance hypothesis that argues that the imbalance should be largest in middle adolescence, both Classes 1 and 2 had higher levels of inhibitory control relative to sensitivity to reward at age 14.

**Substance Use Prediction Models**

*Two Part Growth Models with Random Effects*

Both alcohol and marijuana use were modeled using two-part growth models with random effects (Olsen & Schafer, 2001). All two-part models were estimated in Mplus 8.0 (Muthen & Muthen, 1998-2017) with maximum likelihood estimation with robust standard errors (MLR). Two-part models simultaneously model dichotomous past year use (yes/no) as well as continuous levels of past year use (quantity x frequency of use for alcohol and frequency for marijuana). Continuous use was set to missing for individuals who reported no use in a given year. Continuous levels of alcohol and marijuana use were log-transformed to adjust for non-normality. Dichotomous and continuous growth curves for both alcohol and marijuana were first estimated separately as outlined by Olsen & Schafer (2001). Considering the low rates of alcohol use at age 12 (15 total past year drinkers), the intercept for alcohol use was set at age 13. At age 12, there was only one marijuana user in the past year. This observation was deleted and the intercept for marijuana use was set to age 13. We assessed a variety of parameterizations of growth of alcohol and marijuana use, including polynomial (linear and quadratic trends), piecewise, and nonlinear proportional change (Bollen & Curran 2006). Nested model tests using the Satorra-Bentler method were used to build the most parsimonious and best fitting models for alcohol and marijuana use. After obtaining the best fitting models for the dichotomous and continuous growth curves for alcohol and marijuana use, the intercepts and growth factors for the dichotomous and continuous growth models were estimated to connect the alcohol and marijuana use models, respectively.

*Unconditional Two-Part Growth Models*

**Alcohol Use**. Non-linear slope factors provided the best fit for the dichotomous portion of the model. For the slope factors for the dichotomous portion of the model, the loadings for age 15 and 20 were freely estimated (-1, 0, 1, 2.32, 3, 4, 5, 6, 6.22 slope loadings for ages 12 to 20, respectively). The slope mean was positive and statistically significant (*M*=0.98, p<.001) suggesting an increasing trend in the probability of alcohol use. There was significant variability in the intercept (σ^2^=7.49, p<.001) and slope (σ^2^=0.31, p<.001), indicating significant variability in initial probability of use as well as growth in the probability of use. There was a negative covariance between the intercept and slope (covariance=-0.72, p<.001), suggesting that a high probability of use at age 13 was associated with slower than average increases in the probability of use.

A piecewise model provided the best fit for the continuous portion of the alcohol model. The first linear piece of continuous growth was from ages 12 to 16 and the second linear piece was from ages 16 to 20. The slope mean for ages 12 to 16 (*M*=0.41, p<.001) and 16 to 20 (*M*=1.04, p<.001) were both statistically significant, suggesting that there was an increasing trend in levels of alcohol use from ages 12 to 16 and 16 to 20, albeit more rapid increase in the second segment. There was significant variability in the intercept at age 13 (σ^2^=0.30, p=.006), the slope for ages 12 to 16 (σ^2^=0.04, p=.005) and 16 to 20 (σ^2^=0.36, p<.001). The covariance between the intercept and slope for ages 12 to 16 was statistically significant (covariance=0.08, p<.001), indicating that higher levels of use at age 13 were associated with faster than average increases in levels of use from ages 12 to 16. The covariance between the intercept and slope for ages 16 to 20 (covariance=-0.11, p=.11) was not statistically significant. The slopes for ages 12 to 16 and 16 to 20 were unrelated.

The final two-part growth model with random effects combined growth models for the dichotomous and continuous alcohol variables. The covariance between the intercept of the dichotomous portion of the model was marginally associated with the intercept of continuous levels of alcohol use (covariance = 0.83, p=.07) as well as the continuous slope between ages 12 and 16 (covariance=0.23, p=.06). The slope of the dichotomous portion of the model was also positively associated with the continuous slope of alcohol use from ages 16 to 20 (covariance=0.16, p<.001). Non-significant covariances were constrained to zero to reduce model complexity and assist with model convergence in subsequent models that included imbalance.

**Marijuana Use**. For the two-part marijuana use model, only one participant used marijuana in the past year at age 12. This observation was deleted, and the intercept for the dichotomous portion of the model and the intercept for the continuous portion of the model were both set to age 13. Non-linear slope factors provided the best fit for the dichotomous portion of the model. For the slope factors for the dichotomous portion of the model, the loadings for age 18 and 20 were freely (0, 1, 2, 3, 4, 5.74, 6, 5.98 slope loadings for ages 13 to 20, respectively). The slope mean was positive and statistically significant (*M*=0.86, p<.001) indicating an increase in the probability of marijuana use from ages 13 to 20. There was significant variability in the dichotomous intercept (σ^2^=7.06, p<.001), indicating significant variability in the initial probability of marijuana use. The slope variance was significant (σ^2^=0.35, p<.001), indicating there was significant variability in the growth of the probability of marijuana use across ages 13 to 20.

Non-linear slope factors provided the best fit for the continuous portion of the model. For the slope for the continuous portion, the loadings for ages 16, 18, and 20 were freely estimated (0, 1, 2, 2.43, 4, 5.38, 6, 6.27 slope loadings for ages 13 to 20, respectively). The mean of the intercept (*M*=0.58, p=.06) was marginally significant indicating that initial levels of marijuana use at age 13 were significantly different from 0. The slope mean was statistically significant (*M*=0.29, p<.001) suggesting that there was a significant increase in marijuana use from ages 13 to 20. The variance for the continuous intercept was non-significant (σ^2^=0.63, p=.25) indicating non-significant individual differences in continuous marijuana use at age 13. The slope of continuous marijuana use was statistically significant (σ^2^=0.10, p=.001), indicating significant variability in growth of continuous marijuana use across ages 13 to 20. The covariance between the intercept and slope was not statistically significant (covariance=0.02, p=.87).

The final model two-part growth model with random effects combined growth models for the dichotomous and continuous marijuana variables. The intercepts of the dichotomous and continuous portions of the model were statistically significant (covariance=1.37, p=.03) such that higher probability of age 13 marijuana use was associated with higher levels of marijuana use at age 13. The slope of the dichotomous portion and continuous portions of the model were also statistically significant (covariance=0.10, p=.008) indicating that greater growth in the probability of marijuana use from ages 13 to 20 was associated with greater growth in marijuana use frequency from ages 13 to 20.

*Latent Difference Score Growth Model Predicting Substance Use*

Next, the LDS growth model and two-part growth models for alcohol use and marijuana use were combined to assess the association between imbalance and drug use. Cohen’s (1992) power tables indicated that these models had sufficient power to detect a small to medium sized effect between the intercept and slope of the imbalance and growth in alcohol and marijuana use. All models controlled for gender as prior work has suggested that changes in sensation seeking and self-regulation may vary by gender (Rubia et al., 2013; Walker et al., 2017). Indeed, males were found to have a significantly greater imbalance between sensitivity to reward and inhibitory control at age 12 in both the alcohol (*β*=.23, p<.001) and marijuana use models (*β*=.23, p=.001).

*Alcohol Use.* Model results indicated that initial levels of the imbalance at age 12 did not significantly covary with the probability of alcohol use at age 13 (covariance=0.13, p=.25), growth in the probability of alcohol use (covariance=-0.01, p=.72), levels of alcohol use at age 12 (covariance=0.01, p=.87), growth in levels of alcohol use from ages 12 to 16 (covariance=0.03, p=.27), or growth in levels of alcohol use from ages 16 to 20 (covariance=-0.02, p=.62). Growth in the imbalance from ages 12 to 14 did not significantly covary with the probability of alcohol use at age 13 (covariance=-0.09, p=.48), growth in the probability of alcohol use (covariance=-0.002, p=.95), levels of alcohol use at age 13 (covariance=-0.01, p=.91), growth in levels of alcohol use from ages 12 to 16 (covariance=-0.03, p=.28), or growth in levels of alcohol use from ages 16 to 20 (covariance=0.04, p=.26).

*Marijuana Use.* Model results indicated that initial levels of the imbalance at age 12 did not significantly covary with the probability of marijuana use at age 13 (covariance=-0.04, p=.81), initial levels of marijuana use at age 13 (covariance=0.03, p=.73), growth in the probability of marijuana use (covariance=0.06, p=.13), or growth in frequency of marijuana use from ages 13 to 20 (covariance=-0.003, p=.87). Growth in the imbalance from ages 12 to 14 did not significantly covary with the probability of marijuana use at age 13 (covariance=-0.05, p=.73), initial levels of marijuana use at age 13 (covariance=-0.09, p=.52), growth in the probability of marijuana use (covariance=-0.02, p=.60), or growth in frequency of marijuana use from ages 13 to 20 (covariance=0.02, p=.40).

*GMM Predicting Substance Use*

A categorical variable representing class membership was created by assigning adolescents to their most likely class membership using class probabilities. Categorical class membership was then used to predict two-part models for both alcohol and marijuana use (See Supplemental Materials Figures 1 and 2). Models controlled for gender. Cohen’s (1992) power tables indicated that these models had sufficient power to detect a small to medium sized effect between class membership and growth in alcohol and marijuana use.

*Alcohol Use Prediction Model*: Class membership was not significantly associated with either the probability of use at age 13 (*β*=-.04, p=.51) or growth in the probability of use across ages 12 to 20 (*β*=.03, p=.73). Further, class membership was not associated with levels of alcohol use at age 13 (*β*=-.09, p=.62) or with growth in alcohol use between ages 12 to 16 (*β*=-.19, p=.40) or between 16 to 20 (*β*=.05, p=.50).

*Marijuana Use Prediction Model*: Class membership was not significantly associated with either the probability of marijuana use at age 13 (*β*=-.19, p=.11), growth in the probability of marijuana use from ages 13 to 20 (*β*=.15, p=.15), levels of marijuana use at age 13 (*β*=-.08, p=.66), or growth in the frequency of marijuana use from ages 13 to 20 (*β*=-.03, p=.76).

**References**

Bollen, K. A., & Curran, P. J. (2006). *Latent curve models: A structural equation perspective* (Vol. 467). John Wiley & Sons.

Hipp, J. R., & Bauer, D. J. (2006). Local solutions in the estimation of growth mixture models. *Psychological Methods*, *11*(1), 36-53. DOI: 10.1037/1082-989X.11.1.36

McArdle, J. J., & Grimm, K. J. (2010). Five steps in latent curve and latent change score modeling with longitudinal data. In *Longitudinal research with latent variables* (pp. 245-273). Springer, Berlin, Heidelberg.

Rubia, K., Lim, L., Ecker, C., Halari, R., Giampietro, V., Simmons, A., ... & Smith, A. (2013). Effects of age and gender on neural networks of motor response inhibition: from adolescence to mid-adulthood. *Neuroimage*, *83*, 690-703. <https://doi.org/10.1016/j.neuroimage.2013.06.078>

Walker, D. M., Bell, M. R., Flores, C., Gulley, J. M., Willing, J., & Paul, M. J. (2017). Adolescence and reward: making sense of neural and behavioral changes amid the chaos. *Journal of Neuroscience*, *37*(45), 10855-10866. DOI: https://doi.org/10.1523/JNEUROSCI

Supplemental Materials Table 1. Class Extraction Information.

| Model | LL | AIC | BIC | aBIC | Entropy | BLRT | BLRT *p* | Smallest Class |
| --- | --- | --- | --- | --- | --- | --- | --- | --- |
| GMM-UM (LCGM) | |  |  |  |  |  |  |  |
| Class 1 | -1644.07 | 3310.14 | 3352.94 | 3318.05 | 1.00 | - | - | N=362, 100% |
| Class 2 | -1563.62 | 3159.25 | 3221.51 | 3170.75 | 0.89 | 160.89 | .000 | N=34, 9.39% |
| Class 3 | -1507.61 | 3057.23 | 3138.95 | 3072.33 | 0.86 | 112.02 | .000 | N=32, 8.84% |
| Class 4 | -1479.49 | 3010.98 | 3112.17 | 3029.68 | 0.87 | 56.24 | .000 | N=8, 2.22% |
| GMM-UMSV | |  |  |  |  |  |  |  |
| Class 1 | -1539.66 | 3119.32 | 3197.16 | 3133.71 | 1.00 | - | - | N=362, 100% |
| Class 2 | -1497.35 | 3042.71 | 3136.11 | 3059.97 | 0.90 | 87.09 | .000 | N=39, 10.77% |
| Class 3 | -1474.09 | 3006.19 | 3119.04 | 3027.04 | 0.93 | 46.52 | .000 | N=8, 2.22% |
| Class 4 | -1456.92 | 2969.83 | 3078.80 | 2989.96 | .87 | 60.21 | .000 | N=8, 2.22% |
| GMM-UMUV | |  |  |  |  |  |  |  |
| Class 1 | -1539.66 | 3119.32 | 3197.16 | 3133.71 | 1.00 | - | - | N=362, 100% |
| Class 2 | -1500.89 | 3049.79 | 3143.19 | 3067.05 | 0.47 | 660.70 | .000 | N=148, 40.88% |
| Class 3 | -1473.45 | 3010.90 | 3135.43 | 3033.91 | 0.63 | 490.68 | .000 | N=20, 5.52% |
| Class 4 | -1464.93 | 3009.86 | 3165.53 | 3038.62 | 0.70 | 319.64 | .000 | N=6, 1.66% |

*Note*. LL=log-likelihood, BLRT=Bootstrapped Likelihood Ratio Test. GMM-UM=growth mixture model with unique means, LCGM=latent class growth model, GMM-UMSV=growth mixture model with unique means and shared variances, GMM-UMUV=growth mixture model with unique means and unique variances.


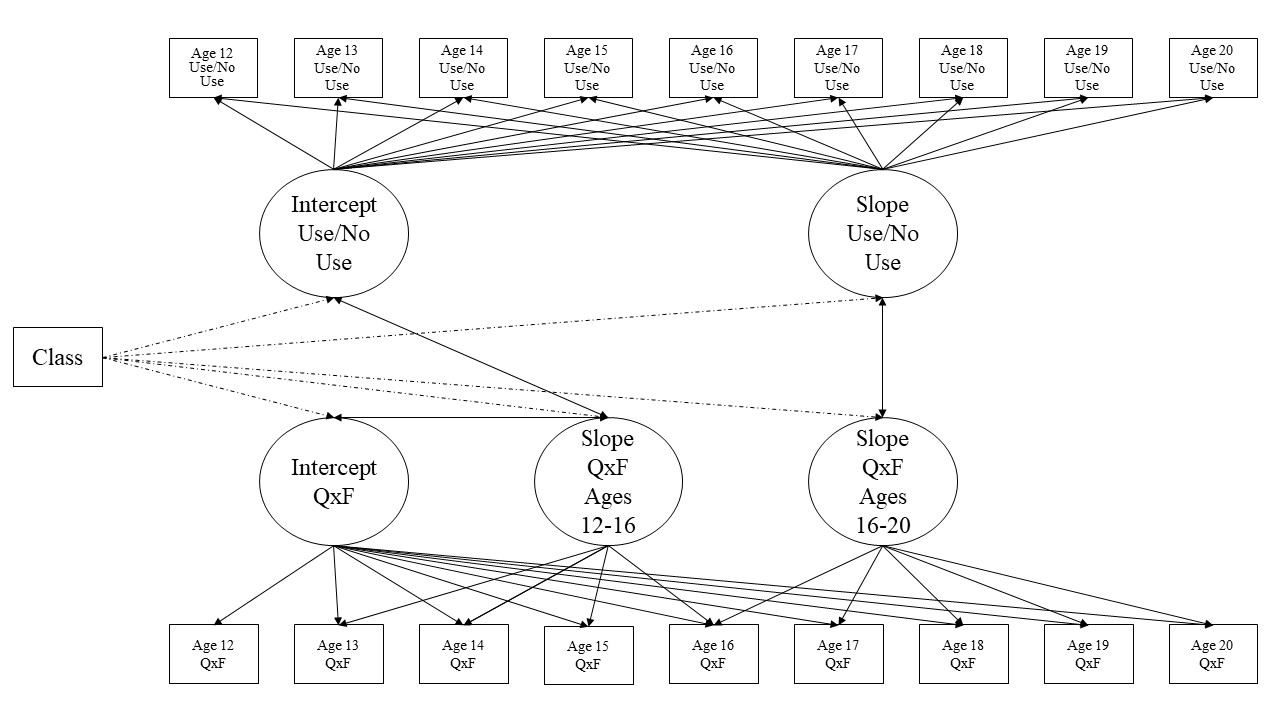


*Supplemental Materials Figure 1*. Class membership predicting the probability of alcohol use at age 13, growth in the probability of alcohol use from ages 12 to 20, initial levels of alcohol use at age 13, and growth in levels of alcohol use from ages 12 to 16 and 16 to 20. QxF=quantity by frequency, use/no use=probability of use. Dashed lines from class membership to slope factors indicate non-significant pathways.

*
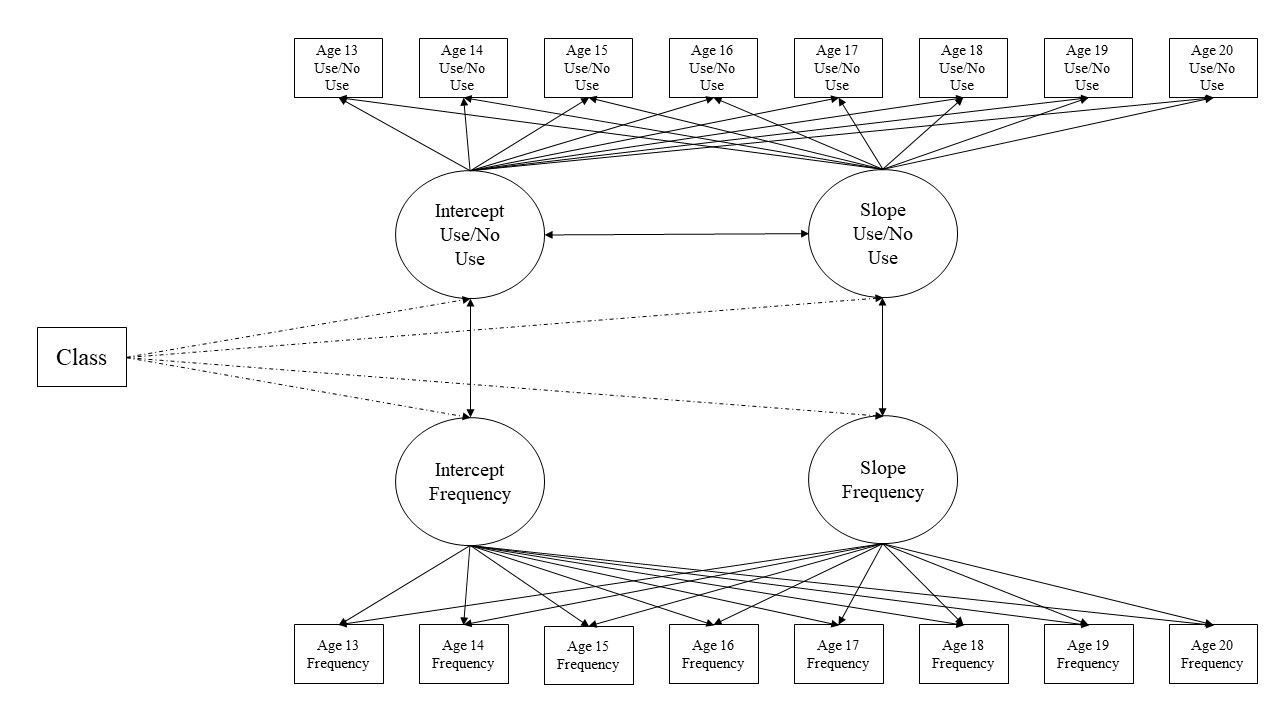
*

*Supplemental Materials Figure 2*. Class membership predicting the probability of marijuana use at age 13, growth in the probability of marijuana use from ages 13 to 20, initial levels of marijuana use at age 13, and growth in levels of marijuana use from ages 13 to 20. Use/no use=probability of use. Dashed lines from class membership to slope factors indicate non-significant pathways.
