# Supplementary material for: Mind the gap: A review and recommendations for statistically evaluating Dual Systems models of adolescent risk behavior
Source: Dev Cogn Neurosci. 2019 Jul 25;39:100681. doi: 10.1016/j.dcn.2019.100681 (PMC6969358; doi:10.1016/j.dcn.2019.100681)
Supplement: Supplementary file 2 [file mmc2.docx]

Mplus VERSION 8.2

MUTHEN & MUTHEN

04/05/2019 3:30 PM

INPUT INSTRUCTIONS

Title: Ages 12-14 LDS

Data: file is Dual Systems LDS R&R.dat;

Variable:

NAMES ARE

subject gender sr12r1 sr12r2 sr12r3 sr13r1 sr13r2 sr13r3

sr14r1 sr14r2 sr14r3 ic12r1 ic12r2 ic12r3 ic13r1 ic13r2

ic13r3 ic14r1 ic14r2 ic14r3;

!SR=sensitivity to reward, IC=inhibitory control

!12=age 12, 13=age 13, 14=age 14

!r1=response block 1, r2=response block 2, r3=response block 3

USEV

ic12r1-ic12r3 ic13r1-ic13r3 ic14r1-ic14r3

sr12r1-sr12r3 sr13r1-sr13r3 sr14r1-sr14r3;

missing are .;

Analysis:

estimator is ML;

model=nocovariances;

Model:

!PSRT Measurement Model

sr12 by sr12r1

sr12r2 (sr2)

sr12r3 ;

sr13 by sr13r1

sr13r2 (sr2)

sr13r3 (sr3);

sr14 by sr14r1

sr14r2 (sr2)

sr14r3 (sr3);

SR13R3 WITH SR13R1;

!Constraining Item Intercepts Within and Across Time for

!Mean Structure Identification

[sr12r1 sr13r1 sr14r1] (sri1);

[sr12r2 sr13r2 sr14r2] (sri1);

[sr12r3 sr13r3 sr14r3] (sri1);

!Constraining Across time Item Residual Variances

sr13r1 sr14r1 (srr1);

sr12r2 sr13r2 sr14r2 (srr2);

sr13r3 sr14r3 (srr3);

!Stop Signal Task Measurment Model

ic12 by ic12r1

ic12r2 (ic2)

ic12r3 (ic3);

ic13 by ic13r1

ic13r2 (ic2)

ic13r3 (ic3);

ic14 by ic14r1

ic14r2 (ic2)

ic14r3 (ic3);

IC14R3 WITH IC12R1;

!Constraining Item Intercepts Within and Across Time for

!Mean Structure Identification

[ic12r1 ic13r1 ic14r1] (ici1);

[ic12r2 ic13r2 ic14r2] (ici1);

[ic12r3 ic13r3 ic14r3] (ici1);

!Constraining Across time Item Residual Variances

ic13r1 ic14r1 (icr1);

ic12r2 ic13r2 (icr2);

ic13r3 ic14r3 (icr3);

!Specifying Latent Difference Score

sr12 on ic12@1;

sr13 on ic13@1;

sr14 on ic14@1;

dfim1 by sr12@1;

dfim2 by sr13@1;

dfim3 by sr14@1;

!Freely Estimated Variances for Latent Difference Score

dfim1 dfim2 dfim3 (dfimres);

!Constrained Difference Score Intercepts for Growth Model

[dfim1@0 dfim2@0 dfim3@0];

!Means and Variances of Sensitivity to Reward Constrained to 0

sr12-sr14@0;

[sr12-sr14@0];

[ic12-ic14];

!Covariance Between Difference Scores and Inhibitory Control

ic12 with dfim1 (icwdfim);

ic13 with dfim2 (icwdfim);

ic14 with dfim3 (icwdfim);

!Latent Growth of Difference Scores

idfim sdfim | dfim1@0 dfim2* dfim3@1;

idfim with sdfim;

!MIs

SR14R3 WITH SR14R2;

SR13R3 WITH SR13R2;

IC13 WITH IC12;

IC14R1 WITH IC12R1;

IC13R3 WITH IC13R1;

IC13R3 WITH IC12R2;

IC13R3 WITH IC12R3;

SR14R2 WITH SR13R1;

Output: res stdyx sampstat mod(5) svalues;

*** WARNING

Data set contains cases with missing on all variables.

These cases were not included in the analysis.

Number of cases with missing on all variables: 25

1 WARNING(S) FOUND IN THE INPUT INSTRUCTIONS

Ages 12-14 LDS

SUMMARY OF ANALYSIS

Number of groups 1

Number of observations 362

Number of dependent variables 18

Number of independent variables 0

Number of continuous latent variables 11

Observed dependent variables

Continuous

IC12R1 IC12R2 IC12R3 IC13R1 IC13R2 IC13R3

IC14R1 IC14R2 IC14R3 SR12R1 SR12R2 SR12R3

SR13R1 SR13R2 SR13R3 SR14R1 SR14R2 SR14R3

Continuous latent variables

SR12 SR13 SR14 IC12 IC13 IC14

DFIM1 DFIM2 DFIM3 IDFIM SDFIM

Estimator ML

Information matrix OBSERVED

Maximum number of iterations 1000

Convergence criterion 0.500D-04

Maximum number of steepest descent iterations 20

Maximum number of iterations for H1 2000

Convergence criterion for H1 0.100D-03

Input data file(s)

Dual Systems LDS R&R.dat

Input data format FREE

SUMMARY OF DATA

Number of missing data patterns 45

COVARIANCE COVERAGE OF DATA

Minimum covariance coverage value 0.100

PROPORTION OF DATA PRESENT

Covariance Coverage

IC12R1 IC12R2 IC12R3 IC13R1 IC13R2

________ ________ ________ ________ ________

IC12R1 0.936

IC12R2 0.906 0.931

IC12R3 0.903 0.903 0.928

IC13R1 0.831 0.831 0.820 0.887

IC13R2 0.834 0.831 0.826 0.859 0.890

IC13R3 0.834 0.834 0.829 0.859 0.870

IC14R1 0.489 0.483 0.483 0.475 0.481

IC14R2 0.497 0.492 0.492 0.483 0.483

IC14R3 0.494 0.489 0.492 0.481 0.481

SR12R1 0.931 0.925 0.923 0.848 0.854

SR12R2 0.931 0.925 0.923 0.848 0.854

SR12R3 0.931 0.925 0.923 0.848 0.854

SR13R1 0.870 0.865 0.862 0.884 0.887

SR13R2 0.870 0.865 0.862 0.884 0.887

SR13R3 0.870 0.865 0.862 0.884 0.887

SR14R1 0.517 0.511 0.511 0.503 0.503

SR14R2 0.517 0.511 0.511 0.503 0.503

SR14R3 0.517 0.511 0.511 0.503 0.503

Covariance Coverage

IC13R3 IC14R1 IC14R2 IC14R3 SR12R1

________ ________ ________ ________ ________

IC13R3 0.892

IC14R1 0.481 0.511

IC14R2 0.486 0.500 0.519

IC14R3 0.483 0.497 0.500 0.517

SR12R1 0.854 0.494 0.503 0.500 0.959

SR12R2 0.854 0.494 0.503 0.500 0.959

SR12R3 0.854 0.494 0.503 0.500 0.959

SR13R1 0.890 0.494 0.503 0.500 0.892

SR13R2 0.890 0.494 0.503 0.500 0.892

SR13R3 0.890 0.494 0.503 0.500 0.892

SR14R1 0.506 0.511 0.519 0.517 0.522

SR14R2 0.506 0.511 0.519 0.517 0.522

SR14R3 0.506 0.511 0.519 0.517 0.522

Covariance Coverage

SR12R2 SR12R3 SR13R1 SR13R2 SR13R3

________ ________ ________ ________ ________

SR12R2 0.959

SR12R3 0.959 0.959

SR13R1 0.892 0.892 0.931

SR13R2 0.892 0.892 0.931 0.931

SR13R3 0.892 0.892 0.931 0.931 0.931

SR14R1 0.522 0.522 0.522 0.522 0.522

SR14R2 0.522 0.522 0.522 0.522 0.522

SR14R3 0.522 0.522 0.522 0.522 0.522

Covariance Coverage

SR14R1 SR14R2 SR14R3

________ ________ ________

SR14R1 0.539

SR14R2 0.539 0.539

SR14R3 0.539 0.539 0.539

SAMPLE STATISTICS

ESTIMATED SAMPLE STATISTICS

Means

IC12R1 IC12R2 IC12R3 IC13R1 IC13R2

________ ________ ________ ________ ________

-0.023 -0.272 -0.223 0.328 0.192

Means

IC13R3 IC14R1 IC14R2 IC14R3 SR12R1

________ ________ ________ ________ ________

0.136 0.411 0.343 0.251 0.673

Means

SR12R2 SR12R3 SR13R1 SR13R2 SR13R3

________ ________ ________ ________ ________

0.578 0.627 0.484 0.509 0.601

Means

SR14R1 SR14R2 SR14R3

________ ________ ________

0.514 0.576 0.620

Covariances

IC12R1 IC12R2 IC12R3 IC13R1 IC13R2

________ ________ ________ ________ ________

IC12R1 1.080

IC12R2 0.525 1.126

IC12R3 0.519 0.676 1.256

IC13R1 0.148 0.187 0.244 0.494

IC13R2 0.143 0.194 0.251 0.241 0.749

IC13R3 0.190 0.298 0.319 0.216 0.394

IC14R1 0.215 0.226 0.222 0.131 0.216

IC14R2 0.136 0.211 0.164 0.160 0.244

IC14R3 0.229 0.165 0.191 0.103 0.232

SR12R1 -0.051 -0.088 -0.069 -0.046 -0.045

SR12R2 -0.060 -0.023 -0.078 -0.016 -0.046

SR12R3 -0.059 -0.012 -0.035 -0.019 -0.035

SR13R1 -0.007 0.015 0.016 -0.005 0.000

SR13R2 -0.008 -0.055 -0.013 -0.023 0.000

SR13R3 -0.036 -0.069 -0.074 -0.068 -0.024

SR14R1 -0.047 -0.032 -0.091 -0.050 -0.008

SR14R2 0.006 -0.026 -0.063 -0.012 -0.026

SR14R3 0.005 -0.021 -0.059 -0.022 -0.016

Covariances

IC13R3 IC14R1 IC14R2 IC14R3 SR12R1

________ ________ ________ ________ ________

IC13R3 0.779

IC14R1 0.131 0.533

IC14R2 0.220 0.242 0.464

IC14R3 0.191 0.237 0.292 0.588

SR12R1 -0.049 -0.126 -0.063 -0.069 0.627

SR12R2 -0.065 -0.127 -0.060 -0.073 0.353

SR12R3 -0.065 -0.081 -0.032 -0.049 0.299

SR13R1 -0.005 -0.035 -0.042 -0.010 0.103

SR13R2 -0.038 -0.061 -0.053 0.006 0.096

SR13R3 -0.072 -0.053 -0.091 -0.033 0.096

SR14R1 -0.059 -0.046 -0.020 -0.046 0.084

SR14R2 -0.006 -0.011 -0.035 -0.003 0.075

SR14R3 -0.043 -0.014 -0.037 -0.022 0.070

Covariances

SR12R2 SR12R3 SR13R1 SR13R2 SR13R3

________ ________ ________ ________ ________

SR12R2 0.574

SR12R3 0.326 0.535

SR13R1 0.115 0.090 0.292

SR13R2 0.136 0.101 0.149 0.334

SR13R3 0.149 0.126 0.102 0.212 0.354

SR14R1 0.063 0.088 0.081 0.072 0.096

SR14R2 0.092 0.061 0.050 0.079 0.077

SR14R3 0.083 0.046 0.079 0.088 0.102

Covariances

SR14R1 SR14R2 SR14R3

________ ________ ________

SR14R1 0.224

SR14R2 0.124 0.324

SR14R3 0.114 0.192 0.297

Correlations

IC12R1 IC12R2 IC12R3 IC13R1 IC13R2

________ ________ ________ ________ ________

IC12R1 1.000

IC12R2 0.477 1.000

IC12R3 0.446 0.569 1.000

IC13R1 0.203 0.251 0.310 1.000

IC13R2 0.159 0.211 0.259 0.396 1.000

IC13R3 0.208 0.318 0.323 0.348 0.516

IC14R1 0.284 0.292 0.271 0.256 0.342

IC14R2 0.192 0.292 0.215 0.335 0.413

IC14R3 0.288 0.203 0.223 0.190 0.350

SR12R1 -0.061 -0.105 -0.078 -0.083 -0.066

SR12R2 -0.076 -0.029 -0.092 -0.030 -0.071

SR12R3 -0.078 -0.015 -0.042 -0.036 -0.055

SR13R1 -0.013 0.026 0.027 -0.013 0.001

SR13R2 -0.014 -0.090 -0.020 -0.056 0.000

SR13R3 -0.059 -0.109 -0.110 -0.162 -0.047

SR14R1 -0.096 -0.063 -0.171 -0.150 -0.020

SR14R2 0.009 -0.044 -0.098 -0.031 -0.053

SR14R3 0.009 -0.037 -0.096 -0.058 -0.034

Correlations

IC13R3 IC14R1 IC14R2 IC14R3 SR12R1

________ ________ ________ ________ ________

IC13R3 1.000

IC14R1 0.203 1.000

IC14R2 0.365 0.487 1.000

IC14R3 0.282 0.424 0.559 1.000

SR12R1 -0.070 -0.217 -0.117 -0.113 1.000

SR12R2 -0.098 -0.229 -0.115 -0.127 0.589

SR12R3 -0.101 -0.151 -0.064 -0.087 0.517

SR13R1 -0.010 -0.088 -0.113 -0.025 0.240

SR13R2 -0.075 -0.145 -0.134 0.014 0.209

SR13R3 -0.138 -0.122 -0.225 -0.072 0.204

SR14R1 -0.142 -0.132 -0.061 -0.128 0.224

SR14R2 -0.012 -0.027 -0.090 -0.006 0.167

SR14R3 -0.090 -0.036 -0.099 -0.052 0.162

Correlations

SR12R2 SR12R3 SR13R1 SR13R2 SR13R3

________ ________ ________ ________ ________

SR12R2 1.000

SR12R3 0.589 1.000

SR13R1 0.281 0.228 1.000

SR13R2 0.311 0.239 0.477 1.000

SR13R3 0.331 0.290 0.317 0.616 1.000

SR14R1 0.176 0.254 0.317 0.262 0.342

SR14R2 0.213 0.147 0.163 0.241 0.226

SR14R3 0.202 0.116 0.267 0.278 0.316

Correlations

SR14R1 SR14R2 SR14R3

________ ________ ________

SR14R1 1.000

SR14R2 0.459 1.000

SR14R3 0.440 0.619 1.000

MAXIMUM LOG-LIKELIHOOD VALUE FOR THE UNRESTRICTED (H1) MODEL IS -5012.754

UNIVARIATE SAMPLE STATISTICS

UNIVARIATE HIGHER-ORDER MOMENT DESCRIPTIVE STATISTICS

Variable/ Mean/ Skewness/ Minimum/ % with Percentiles

Sample Size Variance Kurtosis Maximum Min/Max 20%/60% 40%/80% Median

IC12R1 -0.031 -0.868 -3.200 1.77% -0.707 -0.139 0.033

339.000 1.075 1.081 2.044 0.29% 0.280 0.813

IC12R2 -0.289 -1.156 -3.608 2.37% -0.893 -0.301 -0.146

337.000 1.117 1.608 1.869 0.30% 0.064 0.493

IC12R3 -0.217 -0.780 -3.661 0.89% -0.958 -0.280 -0.093

336.000 1.217 0.773 2.062 0.30% 0.163 0.579

IC13R1 0.325 -0.243 -2.001 0.31% -0.232 0.170 0.304

321.000 0.488 0.381 2.041 0.31% 0.473 0.906

IC13R2 0.184 -0.651 -2.845 0.93% -0.449 0.077 0.272

322.000 0.736 1.178 1.986 0.31% 0.411 0.844

IC13R3 0.117 -0.854 -2.763 2.17% -0.452 -0.005 0.211

323.000 0.767 1.336 1.835 0.31% 0.383 0.789

IC14R1 0.369 -0.628 -1.986 1.08% -0.129 0.229 0.473

185.000 0.527 1.211 2.041 0.54% 0.610 0.875

IC14R2 0.308 -0.615 -2.243 1.06% -0.201 0.177 0.299

188.000 0.452 1.503 1.728 0.53% 0.442 0.910

IC14R3 0.215 -0.497 -2.340 0.53% -0.373 0.070 0.246

187.000 0.586 1.149 2.065 0.53% 0.390 0.889

SR12R1 0.670 1.169 -1.982 0.29% 0.127 0.430 0.532

347.000 0.627 4.247 4.720 0.29% 0.707 1.148

SR12R2 0.574 0.663 -1.727 0.29% 0.033 0.341 0.469

347.000 0.572 1.746 3.573 0.29% 0.645 1.110

SR12R3 0.624 0.370 -1.837 0.29% 0.107 0.433 0.534

347.000 0.532 1.939 4.103 0.29% 0.699 1.104

SR13R1 0.482 0.836 -1.012 0.30% 0.115 0.300 0.406

337.000 0.292 1.992 2.863 0.30% 0.512 0.837

SR13R2 0.506 0.832 -1.550 0.30% 0.109 0.310 0.420

337.000 0.335 1.781 2.906 0.30% 0.503 0.907

SR13R3 0.597 1.181 -0.879 0.30% 0.160 0.379 0.479

337.000 0.355 2.585 3.236 0.30% 0.643 0.964

SR14R1 0.526 0.815 -0.715 0.51% 0.185 0.346 0.468

195.000 0.221 1.999 2.509 0.51% 0.581 0.854

SR14R2 0.574 0.953 -0.657 0.51% 0.131 0.366 0.488

195.000 0.325 1.837 2.885 0.51% 0.648 0.975

SR14R3 0.623 0.766 -0.823 0.51% 0.189 0.410 0.581

195.000 0.300 1.074 2.729 0.51% 0.687 0.994

THE MODEL ESTIMATION TERMINATED NORMALLY

MODEL FIT INFORMATION

Number of Free Parameters 41

Loglikelihood

H0 Value -5145.866

H1 Value -5012.754

Information Criteria

Akaike (AIC) 10373.733

Bayesian (BIC) 10533.290

Sample-Size Adjusted BIC 10403.216

(n* = (n + 2) / 24)

Chi-Square Test of Model Fit

Value 266.224

Degrees of Freedom 148

P-Value 0.0000

RMSEA (Root Mean Square Error Of Approximation)

Estimate 0.047

90 Percent C.I. 0.038 0.056

Probability RMSEA <= .05 0.700

CFI/TLI

CFI 0.915

TLI 0.913

Chi-Square Test of Model Fit for the Baseline Model

Value 1551.368

Degrees of Freedom 153

P-Value 0.0000

SRMR (Standardized Root Mean Square Residual)

Value 0.122

MODEL RESULTS

Two-Tailed

Estimate S.E. Est./S.E. P-Value

IDFIM |

DFIM1 1.000 0.000 999.000 999.000

DFIM2 1.000 0.000 999.000 999.000

DFIM3 1.000 0.000 999.000 999.000

SDFIM |

DFIM1 0.000 0.000 999.000 999.000

DFIM2 0.892 0.092 9.745 0.000

DFIM3 1.000 0.000 999.000 999.000

SR12 BY

SR12R1 1.000 0.000 999.000 999.000

SR12R2 1.009 0.025 40.939 0.000

SR12R3 0.953 0.042 22.685 0.000

SR13 BY

SR13R1 1.000 0.000 999.000 999.000

SR13R2 1.009 0.025 40.939 0.000

SR13R3 0.918 0.032 28.644 0.000

SR14 BY

SR14R1 1.000 0.000 999.000 999.000

SR14R2 1.009 0.025 40.939 0.000

SR14R3 0.918 0.032 28.644 0.000

IC12 BY

IC12R1 1.000 0.000 999.000 999.000

IC12R2 1.341 0.105 12.814 0.000

IC12R3 1.378 0.104 13.281 0.000

IC13 BY

IC13R1 1.000 0.000 999.000 999.000

IC13R2 1.341 0.105 12.814 0.000

IC13R3 1.378 0.104 13.281 0.000

IC14 BY

IC14R1 1.000 0.000 999.000 999.000

IC14R2 1.341 0.105 12.814 0.000

IC14R3 1.378 0.104 13.281 0.000

DFIM1 BY

SR12 1.000 0.000 999.000 999.000

DFIM2 BY

SR13 1.000 0.000 999.000 999.000

DFIM3 BY

SR14 1.000 0.000 999.000 999.000

SR12 ON

IC12 1.000 0.000 999.000 999.000

SR13 ON

IC13 1.000 0.000 999.000 999.000

SR14 ON

IC14 1.000 0.000 999.000 999.000

IC12 WITH

DFIM1 -0.234 0.036 -6.588 0.000

IC13 0.024 0.010 2.519 0.012

IC13 WITH

DFIM2 -0.234 0.036 -6.588 0.000

IC14 WITH

DFIM3 -0.234 0.036 -6.588 0.000

IDFIM WITH

SDFIM -0.175 0.039 -4.507 0.000

SR13R3 WITH

SR13R1 -0.046 0.013 -3.670 0.000

SR13R2 0.067 0.015 4.317 0.000

IC14R3 WITH

IC12R1 0.137 0.041 3.363 0.001

SR14R3 WITH

SR14R2 0.080 0.016 4.994 0.000

IC14R1 WITH

IC12R1 0.084 0.036 2.348 0.019

IC13R3 WITH

IC13R1 -0.098 0.032 -3.014 0.003

IC12R2 0.111 0.034 3.224 0.001

IC12R3 0.099 0.040 2.490 0.013

SR14R2 WITH

SR13R1 -0.029 0.014 -2.156 0.031

Means

IC12 -0.730 0.170 -4.290 0.000

IC13 -0.419 0.153 -2.741 0.006

IC14 -0.330 0.150 -2.206 0.027

IDFIM 0.000 0.000 999.000 999.000

SDFIM -0.471 0.067 -7.003 0.000

Intercepts

IC12R1 0.736 0.164 4.496 0.000

IC12R2 0.736 0.164 4.496 0.000

IC12R3 0.736 0.164 4.496 0.000

IC13R1 0.736 0.164 4.496 0.000

IC13R2 0.736 0.164 4.496 0.000

IC13R3 0.736 0.164 4.496 0.000

IC14R1 0.736 0.164 4.496 0.000

IC14R2 0.736 0.164 4.496 0.000

IC14R3 0.736 0.164 4.496 0.000

SR12R1 1.343 0.174 7.733 0.000

SR12R2 1.343 0.174 7.733 0.000

SR12R3 1.343 0.174 7.733 0.000

SR13R1 1.343 0.174 7.733 0.000

SR13R2 1.343 0.174 7.733 0.000

SR13R3 1.343 0.174 7.733 0.000

SR14R1 1.343 0.174 7.733 0.000

SR14R2 1.343 0.174 7.733 0.000

SR14R3 1.343 0.174 7.733 0.000

SR12 0.000 0.000 999.000 999.000

SR13 0.000 0.000 999.000 999.000

SR14 0.000 0.000 999.000 999.000

DFIM1 0.000 0.000 999.000 999.000

DFIM2 0.000 0.000 999.000 999.000

DFIM3 0.000 0.000 999.000 999.000

Variances

IC12 0.262 0.035 7.506 0.000

IC13 0.227 0.034 6.754 0.000

IC14 0.202 0.032 6.231 0.000

IDFIM 0.252 0.040 6.258 0.000

SDFIM 0.182 0.048 3.803 0.000

Residual Variances

IC12R1 0.717 0.065 10.999 0.000

IC12R2 0.448 0.039 11.379 0.000

IC12R3 0.605 0.065 9.270 0.000

IC13R1 0.313 0.029 10.992 0.000

IC13R2 0.448 0.039 11.379 0.000

IC13R3 0.318 0.035 9.016 0.000

IC14R1 0.313 0.029 10.992 0.000

IC14R2 0.170 0.033 5.077 0.000

IC14R3 0.318 0.035 9.016 0.000

SR12R1 0.309 0.030 10.324 0.000

SR12R2 0.196 0.014 14.337 0.000

SR12R3 0.247 0.026 9.667 0.000

SR13R1 0.127 0.013 9.586 0.000

SR13R2 0.196 0.014 14.337 0.000

SR13R3 0.203 0.018 11.072 0.000

SR14R1 0.127 0.013 9.586 0.000

SR14R2 0.196 0.014 14.337 0.000

SR14R3 0.203 0.018 11.072 0.000

SR12 0.000 0.000 999.000 999.000

SR13 0.000 0.000 999.000 999.000

SR14 0.000 0.000 999.000 999.000

DFIM1 0.311 0.044 7.033 0.000

DFIM2 0.311 0.044 7.033 0.000

DFIM3 0.311 0.044 7.033 0.000

STANDARDIZED MODEL RESULTS

STDYX Standardization

Two-Tailed

Estimate S.E. Est./S.E. P-Value

IDFIM |

DFIM1 0.669 0.045 14.900 0.000

DFIM2 0.797 0.084 9.511 0.000

DFIM3 0.798 0.085 9.419 0.000

SDFIM |

DFIM1 0.000 0.000 999.000 999.000

DFIM2 0.604 0.096 6.325 0.000

DFIM3 0.678 0.100 6.746 0.000

SR12 BY

SR12R1 0.732 0.025 29.272 0.000

SR12R2 0.806 0.019 42.000 0.000

SR12R3 0.753 0.027 28.187 0.000

SR13 BY

SR13R1 0.742 0.029 25.954 0.000

SR13R2 0.668 0.025 27.207 0.000

SR13R3 0.626 0.032 19.437 0.000

SR14 BY

SR14R1 0.711 0.033 21.605 0.000

SR14R2 0.634 0.030 21.159 0.000

SR14R3 0.591 0.036 16.441 0.000

IC12 BY

IC12R1 0.517 0.034 15.244 0.000

IC12R2 0.716 0.027 26.862 0.000

IC12R3 0.672 0.030 22.658 0.000

IC13 BY

IC13R1 0.648 0.038 16.949 0.000

IC13R2 0.690 0.025 27.141 0.000

IC13R3 0.759 0.027 28.274 0.000

IC14 BY

IC14R1 0.626 0.041 15.351 0.000

IC14R2 0.825 0.031 26.642 0.000

IC14R3 0.740 0.028 26.360 0.000

DFIM1 BY

SR12 1.256 0.061 20.519 0.000

DFIM2 BY

SR13 1.598 0.099 16.186 0.000

DFIM3 BY

SR14 1.747 0.127 13.753 0.000

SR12 ON

IC12 0.857 0.074 11.634 0.000

SR13 ON

IC13 1.210 0.107 11.327 0.000

SR14 ON

IC14 1.249 0.129 9.679 0.000

IC12 WITH

DFIM1 -0.819 0.042 -19.317 0.000

IC13 0.100 0.041 2.428 0.015

IC13 WITH

DFIM2 -0.880 0.027 -32.093 0.000

IC14 WITH

DFIM3 -0.933 0.027 -33.963 0.000

IDFIM WITH

SDFIM -0.816 0.042 -19.488 0.000

SR13R3 WITH

SR13R1 -0.289 0.087 -3.330 0.001

SR13R2 0.334 0.063 5.315 0.000

IC14R3 WITH

IC12R1 0.288 0.082 3.496 0.000

SR14R3 WITH

SR14R2 0.399 0.066 6.092 0.000

IC14R1 WITH

IC12R1 0.176 0.074 2.385 0.017

IC13R3 WITH

IC13R1 -0.310 0.114 -2.730 0.006

IC12R2 0.293 0.089 3.312 0.001

IC12R3 0.227 0.090 2.523 0.012

SR14R2 WITH

SR13R1 -0.185 0.085 -2.181 0.029

Means

IC12 -1.426 0.275 -5.189 0.000

IC13 -0.879 0.279 -3.154 0.002

IC14 -0.734 0.295 -2.493 0.013

IDFIM 0.000 0.000 999.000 999.000

SDFIM -1.104 0.205 -5.396 0.000

Intercepts

IC12R1 0.744 0.164 4.539 0.000

IC12R2 0.767 0.175 4.387 0.000

IC12R3 0.701 0.160 4.380 0.000

IC13R1 1.001 0.213 4.702 0.000

IC13R2 0.795 0.178 4.472 0.000

IC13R3 0.850 0.195 4.354 0.000

IC14R1 1.025 0.219 4.688 0.000

IC14R2 1.008 0.228 4.424 0.000

IC14R3 0.879 0.200 4.388 0.000

SR12R1 1.646 0.227 7.260 0.000

SR12R2 1.796 0.248 7.243 0.000

SR12R3 1.777 0.233 7.633 0.000

SR13R1 2.529 0.337 7.501 0.000

SR13R2 2.258 0.297 7.590 0.000

SR13R3 2.325 0.304 7.652 0.000

SR14R1 2.652 0.362 7.328 0.000

SR14R2 2.346 0.313 7.487 0.000

SR14R3 2.404 0.321 7.497 0.000

SR12 0.000 0.000 999.000 999.000

SR13 0.000 0.000 999.000 999.000

SR14 0.000 0.000 999.000 999.000

DFIM1 0.000 0.000 999.000 999.000

DFIM2 0.000 0.000 999.000 999.000

DFIM3 0.000 0.000 999.000 999.000

Variances

IC12 1.000 0.000 999.000 999.000

IC13 1.000 0.000 999.000 999.000

IC14 1.000 0.000 999.000 999.000

IDFIM 1.000 0.000 999.000 999.000

SDFIM 1.000 0.000 999.000 999.000

Residual Variances

IC12R1 0.732 0.035 20.846 0.000

IC12R2 0.488 0.038 12.778 0.000

IC12R3 0.549 0.040 13.783 0.000

IC13R1 0.580 0.050 11.697 0.000

IC13R2 0.523 0.035 14.904 0.000

IC13R3 0.424 0.041 10.417 0.000

IC14R1 0.608 0.051 11.908 0.000

IC14R2 0.319 0.051 6.234 0.000

IC14R3 0.453 0.042 10.916 0.000

SR12R1 0.464 0.037 12.682 0.000

SR12R2 0.350 0.031 11.329 0.000

SR12R3 0.433 0.040 10.758 0.000

SR13R1 0.450 0.042 10.619 0.000

SR13R2 0.554 0.033 16.888 0.000

SR13R3 0.608 0.040 15.078 0.000

SR14R1 0.495 0.047 10.588 0.000

SR14R2 0.598 0.038 15.732 0.000

SR14R3 0.650 0.043 15.277 0.000

SR12 0.000 999.000 999.000 999.000

SR13 0.000 999.000 999.000 999.000

SR14 0.000 999.000 999.000 999.000

DFIM1 0.553 0.060 9.217 0.000

DFIM2 0.786 0.040 19.459 0.000

DFIM3 0.787 0.043 18.364 0.000

R-SQUARE

Observed Two-Tailed

Variable Estimate S.E. Est./S.E. P-Value

IC12R1 0.268 0.035 7.622 0.000

IC12R2 0.512 0.038 13.431 0.000

IC12R3 0.451 0.040 11.329 0.000

IC13R1 0.420 0.050 8.474 0.000

IC13R2 0.477 0.035 13.570 0.000

IC13R3 0.576 0.041 14.137 0.000

IC14R1 0.392 0.051 7.675 0.000

IC14R2 0.681 0.051 13.321 0.000

IC14R3 0.547 0.042 13.180 0.000

SR12R1 0.536 0.037 14.636 0.000

SR12R2 0.650 0.031 21.000 0.000

SR12R3 0.567 0.040 14.094 0.000

SR13R1 0.550 0.042 12.977 0.000

SR13R2 0.446 0.033 13.603 0.000

SR13R3 0.392 0.040 9.718 0.000

SR14R1 0.505 0.047 10.802 0.000

SR14R2 0.402 0.038 10.579 0.000

SR14R3 0.350 0.043 8.220 0.000

Latent Two-Tailed

Variable Estimate S.E. Est./S.E. P-Value

SR12 1.000 999.000 999.000 999.000

SR13 1.000 999.000 999.000 999.000

SR14 1.000 999.000 999.000 999.000

DFIM1 0.447 0.060 7.450 0.000

DFIM2 0.214 0.040 5.300 0.000

DFIM3 0.213 0.043 4.969 0.000

QUALITY OF NUMERICAL RESULTS

Condition Number for the Information Matrix 0.586E-04

(ratio of smallest to largest eigenvalue)

MODEL COMMAND WITH FINAL ESTIMATES USED AS STARTING VALUES

idfim sdfim | dfim1@0 dfim2* dfim3@1;

sr12 BY sr12r1@1;

sr12 BY sr12r2*1.00898 (sr2);

sr12 BY sr12r3*0.95291;

sr13 BY sr13r1@1;

sr13 BY sr13r2*1.00898 (sr2);

sr13 BY sr13r3*0.91820 (sr3);

sr14 BY sr14r1@1;

sr14 BY sr14r2*1.00898 (sr2);

sr14 BY sr14r3*0.91820 (sr3);

ic12 BY ic12r1@1;

ic12 BY ic12r2*1.34083 (ic2);

ic12 BY ic12r3*1.37764 (ic3);

ic13 BY ic13r1@1;

ic13 BY ic13r2*1.34083 (ic2);

ic13 BY ic13r3*1.37764 (ic3);

ic14 BY ic14r1@1;

ic14 BY ic14r2*1.34083 (ic2);

ic14 BY ic14r3*1.37764 (ic3);

dfim1 BY sr12@1;

dfim2 BY sr13@1;

dfim3 BY sr14@1;

sdfim BY dfim2*0.89209;

sr12 ON ic12@1;

sr13 ON ic13@1;

sr14 ON ic14@1;

sr13r3 WITH sr13r1*-0.04635;

sr13r3 WITH sr13r2*0.06667;

ic14r3 WITH ic12r1*0.13729;

sr14r3 WITH sr14r2*0.07967;

ic14r1 WITH ic12r1*0.08356;

ic13r3 WITH ic13r1*-0.09790;

ic13r3 WITH ic12r2*0.11064;

ic13r3 WITH ic12r3*0.09948;

sr14r2 WITH sr13r1*-0.02914;

ic12 WITH dfim1*-0.23401 (icwdfim);

ic12 WITH ic13*0.02441;

ic13 WITH dfim2*-0.23401 (icwdfim);

ic14 WITH dfim3*-0.23401 (icwdfim);

idfim WITH sdfim*-0.17456;

[ ic12r1*0.73570 ] (ici1);

[ ic12r2*0.73570 ] (ici1);

[ ic12r3*0.73570 ] (ici1);

[ ic13r1*0.73570 ] (ici1);

[ ic13r2*0.73570 ] (ici1);

[ ic13r3*0.73570 ] (ici1);

[ ic14r1*0.73570 ] (ici1);

[ ic14r2*0.73570 ] (ici1);

[ ic14r3*0.73570 ] (ici1);

[ sr12r1*1.34328 ] (sri1);

[ sr12r2*1.34328 ] (sri1);

[ sr12r3*1.34328 ] (sri1);

[ sr13r1*1.34328 ] (sri1);

[ sr13r2*1.34328 ] (sri1);

[ sr13r3*1.34328 ] (sri1);

[ sr14r1*1.34328 ] (sri1);

[ sr14r2*1.34328 ] (sri1);

[ sr14r3*1.34328 ] (sri1);

[ sr12@0 ];

[ sr13@0 ];

[ sr14@0 ];

[ ic12*-0.73000 ];

[ ic13*-0.41863 ];

[ ic14*-0.33008 ];

[ dfim1@0 ];

[ dfim2@0 ];

[ dfim3@0 ];

[ idfim@0 ];

[ sdfim*-0.47072 ];

ic12r1*0.71691;

ic12r2*0.44835 (icr2);

ic12r3*0.60524;

ic13r1*0.31343 (icr1);

ic13r2*0.44835 (icr2);

ic13r3*0.31756 (icr3);

ic14r1*0.31343 (icr1);

ic14r2*0.16996;

ic14r3*0.31756 (icr3);

sr12r1*0.30935;

sr12r2*0.19608 (srr2);

sr12r3*0.24746;

sr13r1*0.12696 (srr1);

sr13r2*0.19608 (srr2);

sr13r3*0.20295 (srr3);

sr14r1*0.12696 (srr1);

sr14r2*0.19608 (srr2);

sr14r3*0.20295 (srr3);

sr12@0;

sr13@0;

sr14@0;

ic12*0.26212;

ic13*0.22707;

ic14*0.20202;

dfim1*0.31130 (dfimres);

dfim2*0.31130 (dfimres);

dfim3*0.31130 (dfimres);

idfim*0.25163;

sdfim*0.18172;

RESIDUAL OUTPUT

ESTIMATED MODEL AND RESIDUALS (OBSERVED - ESTIMATED)

Model Estimated Means

IC12R1 IC12R2 IC12R3 IC13R1 IC13R2

________ ________ ________ ________ ________

0.006 -0.243 -0.270 0.317 0.174

Model Estimated Means

IC13R3 IC14R1 IC14R2 IC14R3 SR12R1

________ ________ ________ ________ ________

0.159 0.406 0.293 0.281 0.613

Model Estimated Means

SR12R2 SR12R3 SR13R1 SR13R2 SR13R3

________ ________ ________ ________ ________

0.607 0.648 0.505 0.497 0.573

Model Estimated Means

SR14R1 SR14R2 SR14R3

________ ________ ________

0.542 0.535 0.608

Residuals for Means

IC12R1 IC12R2 IC12R3 IC13R1 IC13R2

________ ________ ________ ________ ________

-0.029 -0.029 0.047 0.011 0.018

Residuals for Means

IC13R3 IC14R1 IC14R2 IC14R3 SR12R1

________ ________ ________ ________ ________

-0.023 0.006 0.050 -0.030 0.059

Residuals for Means

SR12R2 SR12R3 SR13R1 SR13R2 SR13R3

________ ________ ________ ________ ________

-0.029 -0.020 -0.021 0.011 0.028

Residuals for Means

SR14R1 SR14R2 SR14R3

________ ________ ________

-0.028 0.041 0.012

Standardized Residuals (z-scores) for Means

IC12R1 IC12R2 IC12R3 IC13R1 IC13R2

________ ________ ________ ________ ________

-0.781 -0.901 1.491 0.552 0.687

Standardized Residuals (z-scores) for Means

IC13R3 IC14R1 IC14R2 IC14R3 SR12R1

________ ________ ________ ________ ________

-0.891 0.175 2.869 -1.083 3.082

Standardized Residuals (z-scores) for Means

SR12R2 SR12R3 SR13R1 SR13R2 SR13R3

________ ________ ________ ________ ________

-1.924 -3.070 -1.638 0.791 1.622

Standardized Residuals (z-scores) for Means

SR14R1 SR14R2 SR14R3

________ ________ ________

-2.338 1.607 0.554

Normalized Residuals for Means

IC12R1 IC12R2 IC12R3 IC13R1 IC13R2

________ ________ ________ ________ ________

-0.510 -0.507 0.770 0.283 0.378

Normalized Residuals for Means

IC13R3 IC14R1 IC14R2 IC14R3 SR12R1

________ ________ ________ ________ ________

-0.477 0.113 1.050 -0.545 1.396

Normalized Residuals for Means

SR12R2 SR12R3 SR13R1 SR13R2 SR13R3

________ ________ ________ ________ ________

-0.720 -0.522 -0.708 0.366 0.863

Normalized Residuals for Means

SR14R1 SR14R2 SR14R3

________ ________ ________

-0.847 0.995 0.317

Model Estimated Covariances

IC12R1 IC12R2 IC12R3 IC13R1 IC13R2

________ ________ ________ ________ ________

IC12R1 0.979

IC12R2 0.351 0.920

IC12R3 0.361 0.484 1.103

IC13R1 0.024 0.033 0.034 0.541

IC13R2 0.033 0.044 0.045 0.304 0.857

IC13R3 0.034 0.156 0.146 0.215 0.419

IC14R1 0.084 0.000 0.000 0.000 0.000

IC14R2 0.000 0.000 0.000 0.000 0.000

IC14R3 0.137 0.000 0.000 0.000 0.000

SR12R1 0.028 0.038 0.039 0.024 0.033

SR12R2 0.028 0.038 0.039 0.025 0.033

SR12R3 0.027 0.036 0.037 0.023 0.031

SR13R1 0.024 0.033 0.034 -0.007 -0.009

SR13R2 0.025 0.033 0.034 -0.007 -0.009

SR13R3 0.022 0.030 0.031 -0.006 -0.009

SR14R1 0.000 0.000 0.000 0.000 0.000

SR14R2 0.000 0.000 0.000 0.000 0.000

SR14R3 0.000 0.000 0.000 0.000 0.000

Model Estimated Covariances

IC13R3 IC14R1 IC14R2 IC14R3 SR12R1

________ ________ ________ ________ ________

IC13R3 0.749

IC14R1 0.000 0.515

IC14R2 0.000 0.271 0.533

IC14R3 0.000 0.278 0.373 0.701

SR12R1 0.034 0.000 0.000 0.000 0.666

SR12R2 0.034 0.000 0.000 0.000 0.360

SR12R3 0.032 0.000 0.000 0.000 0.340

SR13R1 -0.010 0.000 0.000 0.000 0.120

SR13R2 -0.010 0.000 0.000 0.000 0.121

SR13R3 -0.009 0.000 0.000 0.000 0.110

SR14R1 0.000 -0.032 -0.043 -0.044 0.077

SR14R2 0.000 -0.032 -0.043 -0.044 0.078

SR14R3 0.000 -0.029 -0.039 -0.040 0.071

Model Estimated Covariances

SR12R2 SR12R3 SR13R1 SR13R2 SR13R3

________ ________ ________ ________ ________

SR12R2 0.560

SR12R3 0.343 0.572

SR13R1 0.121 0.115 0.282

SR13R2 0.122 0.116 0.157 0.354

SR13R3 0.111 0.105 0.096 0.210 0.334

SR14R1 0.078 0.073 0.083 0.084 0.077

SR14R2 0.078 0.074 0.055 0.085 0.077

SR14R3 0.071 0.067 0.077 0.077 0.070

Model Estimated Covariances

SR14R1 SR14R2 SR14R3

________ ________ ________

SR14R1 0.256

SR14R2 0.131 0.328

SR14R3 0.119 0.200 0.312

Model Estimated Correlations

IC12R1 IC12R2 IC12R3 IC13R1 IC13R2

________ ________ ________ ________ ________

IC12R1 1.000

IC12R2 0.370 1.000

IC12R3 0.348 0.481 1.000

IC13R1 0.034 0.046 0.044 1.000

IC13R2 0.036 0.049 0.046 0.447 1.000

IC13R3 0.039 0.188 0.160 0.338 0.524

IC14R1 0.118 0.000 0.000 0.000 0.000

IC14R2 0.000 0.000 0.000 0.000 0.000

IC14R3 0.166 0.000 0.000 0.000 0.000

SR12R1 0.035 0.048 0.045 0.041 0.043

SR12R2 0.038 0.053 0.050 0.045 0.048

SR12R3 0.036 0.050 0.046 0.042 0.045

SR13R1 0.046 0.064 0.060 -0.018 -0.019

SR13R2 0.042 0.058 0.054 -0.016 -0.017

SR13R3 0.039 0.054 0.051 -0.015 -0.016

SR14R1 0.000 0.000 0.000 0.000 0.000

SR14R2 0.000 0.000 0.000 0.000 0.000

SR14R3 0.000 0.000 0.000 0.000 0.000

Model Estimated Correlations

IC13R3 IC14R1 IC14R2 IC14R3 SR12R1

________ ________ ________ ________ ________

IC13R3 1.000

IC14R1 0.000 1.000

IC14R2 0.000 0.517 1.000

IC14R3 0.000 0.463 0.610 1.000

SR12R1 0.048 0.000 0.000 0.000 1.000

SR12R2 0.052 0.000 0.000 0.000 0.590

SR12R3 0.049 0.000 0.000 0.000 0.551

SR13R1 -0.021 0.000 0.000 0.000 0.277

SR13R2 -0.019 0.000 0.000 0.000 0.250

SR13R3 -0.018 0.000 0.000 0.000 0.234

SR14R1 0.000 -0.088 -0.116 -0.104 0.186

SR14R2 0.000 -0.079 -0.103 -0.093 0.166

SR14R3 0.000 -0.073 -0.097 -0.087 0.155

Model Estimated Correlations

SR12R2 SR12R3 SR13R1 SR13R2 SR13R3

________ ________ ________ ________ ________

SR12R2 1.000

SR12R3 0.607 1.000

SR13R1 0.306 0.285 1.000

SR13R2 0.275 0.257 0.495 1.000

SR13R3 0.258 0.241 0.313 0.612 1.000

SR14R1 0.205 0.192 0.310 0.279 0.262

SR14R2 0.183 0.171 0.181 0.249 0.234

SR14R3 0.171 0.160 0.258 0.233 0.218

Model Estimated Correlations

SR14R1 SR14R2 SR14R3

________ ________ ________

SR14R1 1.000

SR14R2 0.451 1.000

SR14R3 0.420 0.624 1.000

Residuals for Covariances

IC12R1 IC12R2 IC12R3 IC13R1 IC13R2

________ ________ ________ ________ ________

IC12R1 0.101

IC12R2 0.174 0.207

IC12R3 0.158 0.192 0.153

IC13R1 0.124 0.154 0.211 -0.047

IC13R2 0.111 0.150 0.206 -0.064 -0.108

IC13R3 0.157 0.142 0.173 0.001 -0.025

IC14R1 0.132 0.226 0.222 0.131 0.216

IC14R2 0.136 0.211 0.164 0.160 0.244

IC14R3 0.092 0.165 0.191 0.103 0.232

SR12R1 -0.079 -0.126 -0.108 -0.071 -0.078

SR12R2 -0.088 -0.061 -0.117 -0.040 -0.079

SR12R3 -0.086 -0.048 -0.072 -0.042 -0.066

SR13R1 -0.032 -0.018 -0.017 0.002 0.010

SR13R2 -0.033 -0.088 -0.047 -0.016 0.009

SR13R3 -0.059 -0.099 -0.104 -0.061 -0.015

SR14R1 -0.047 -0.032 -0.091 -0.050 -0.008

SR14R2 0.006 -0.026 -0.063 -0.012 -0.026

SR14R3 0.005 -0.021 -0.059 -0.022 -0.016

Residuals for Covariances

IC13R3 IC14R1 IC14R2 IC14R3 SR12R1

________ ________ ________ ________ ________

IC13R3 0.030

IC14R1 0.131 0.018

IC14R2 0.220 -0.028 -0.069

IC14R3 0.191 -0.041 -0.081 -0.113

SR12R1 -0.083 -0.126 -0.063 -0.069 -0.039

SR12R2 -0.099 -0.127 -0.060 -0.073 -0.007

SR12R3 -0.097 -0.081 -0.032 -0.049 -0.041

SR13R1 0.005 -0.035 -0.042 -0.010 -0.018

SR13R2 -0.029 -0.061 -0.053 0.006 -0.026

SR13R3 -0.063 -0.053 -0.091 -0.033 -0.015

SR14R1 -0.059 -0.014 0.023 -0.002 0.007

SR14R2 -0.006 0.021 0.008 0.042 -0.002

SR14R3 -0.043 0.015 0.003 0.019 -0.001

Residuals for Covariances

SR12R2 SR12R3 SR13R1 SR13R2 SR13R3

________ ________ ________ ________ ________

SR12R2 0.014

SR12R3 -0.017 -0.037

SR13R1 -0.006 -0.024 0.010

SR13R2 0.014 -0.014 -0.007 -0.020

SR13R3 0.038 0.021 0.006 0.002 0.020

SR14R1 -0.014 0.014 -0.002 -0.012 0.020

SR14R2 0.014 -0.013 -0.005 -0.006 -0.001

SR14R3 0.012 -0.021 0.002 0.010 0.032

Residuals for Covariances

SR14R1 SR14R2 SR14R3

________ ________ ________

SR14R1 -0.032

SR14R2 -0.007 -0.004

SR14R3 -0.005 -0.008 -0.015

Residuals for Correlations

IC12R1 IC12R2 IC12R3 IC13R1 IC13R2

________ ________ ________ ________ ________

IC12R1 0.000

IC12R2 0.106 0.000

IC12R3 0.098 0.088 0.000

IC13R1 0.169 0.204 0.267 0.000

IC13R2 0.124 0.162 0.212 -0.052 0.000

IC13R3 0.168 0.131 0.162 0.010 -0.008

IC14R1 0.166 0.292 0.271 0.256 0.342

IC14R2 0.192 0.292 0.215 0.335 0.413

IC14R3 0.122 0.203 0.223 0.190 0.350

SR12R1 -0.096 -0.153 -0.123 -0.124 -0.109

SR12R2 -0.115 -0.082 -0.142 -0.075 -0.119

SR12R3 -0.114 -0.065 -0.089 -0.078 -0.099

SR13R1 -0.059 -0.039 -0.034 0.005 0.019

SR13R2 -0.056 -0.148 -0.074 -0.040 0.017

SR13R3 -0.098 -0.163 -0.161 -0.147 -0.031

SR14R1 -0.096 -0.063 -0.171 -0.150 -0.020

SR14R2 0.009 -0.044 -0.098 -0.031 -0.053

SR14R3 0.009 -0.037 -0.096 -0.058 -0.034

Residuals for Correlations

IC13R3 IC14R1 IC14R2 IC14R3 SR12R1

________ ________ ________ ________ ________

IC13R3 0.000

IC14R1 0.203 0.000

IC14R2 0.365 -0.029 0.000

IC14R3 0.282 -0.039 -0.052 0.000

SR12R1 -0.118 -0.217 -0.117 -0.113 0.000

SR12R2 -0.150 -0.229 -0.115 -0.127 -0.001

SR12R3 -0.150 -0.151 -0.064 -0.087 -0.035

SR13R1 0.011 -0.088 -0.113 -0.025 -0.038

SR13R2 -0.056 -0.145 -0.134 0.014 -0.041

SR13R3 -0.120 -0.122 -0.225 -0.072 -0.031

SR14R1 -0.142 -0.044 0.055 -0.024 0.038

SR14R2 -0.012 0.051 0.013 0.086 0.001

SR14R3 -0.090 0.037 -0.002 0.034 0.007

Residuals for Correlations

SR12R2 SR12R3 SR13R1 SR13R2 SR13R3

________ ________ ________ ________ ________

SR12R2 0.000

SR12R3 -0.018 0.000

SR13R1 -0.025 -0.057 0.000

SR13R2 0.036 -0.018 -0.018 0.000

SR13R3 0.073 0.049 0.004 0.004 0.000

SR14R1 -0.029 0.062 0.007 -0.017 0.080

SR14R2 0.030 -0.024 -0.018 -0.008 -0.008

SR14R3 0.031 -0.044 0.009 0.046 0.098

Residuals for Correlations

SR14R1 SR14R2 SR14R3

________ ________ ________

SR14R1 0.000

SR14R2 0.008 0.000

SR14R3 0.019 -0.005 0.000

Standardized Residuals (z-scores) for Covariances

IC12R1 IC12R2 IC12R3 IC13R1 IC13R2

________ ________ ________ ________ ________

IC12R1 1.980

IC12R2 2.912 2.869

IC12R3 2.495 3.055 2.265

IC13R1 3.037 3.694 4.680 -2.342

IC13R2 2.221 3.020 3.801 -2.513 -2.905

IC13R3 3.141 3.445 4.266 0.046 -0.750

IC14R1 3.566 4.182 3.893 3.505 4.481

IC14R2 2.885 4.174 3.049 4.786 5.497

IC14R3 2.545 2.907 3.182 2.649 4.580

SR12R1 -1.958 -3.335 -2.691 -2.309 -2.152

SR12R2 -2.295 -1.757 -3.087 -1.464 -2.329

SR12R3 -2.309 -1.400 -1.958 -1.560 -2.005

SR13R1 -1.075 -0.632 -0.573 0.112 0.468

SR13R2 -1.020 -2.794 -1.386 -0.804 0.408

SR13R3 -1.729 -2.957 -2.922 -2.961 -0.618

SR14R1 -1.401 -0.905 -2.422 -2.083 -0.262

SR14R2 0.140 -0.627 -1.419 -0.431 -0.700

SR14R3 0.143 -0.542 -1.411 -0.833 -0.459

Standardized Residuals (z-scores) for Covariances

IC13R3 IC14R1 IC14R2 IC14R3 SR12R1

________ ________ ________ ________ ________

IC13R3 0.692

IC14R1 2.733 0.405

IC14R2 4.947 -0.993 -3.221

IC14R3 3.845 -1.261 -2.699 -2.705

SR12R1 -2.235 -2.863 -1.538 -1.475 -7.076

SR12R2 -2.862 -3.191 -1.587 -1.747 -1.415

SR12R3 -2.909 -2.236 -0.937 -1.266 -5.034

SR13R1 0.244 -1.247 -1.614 -0.344 -1.117

SR13R2 -1.252 -2.102 -1.960 0.199 -1.368

SR13R3 -2.565 -1.737 -3.191 -1.030 -0.685

SR14R1 -1.922 -0.632 1.374 -0.117 0.310

SR14R2 -0.164 0.773 0.361 1.598 -0.083

SR14R3 -1.247 0.577 0.119 0.728 -0.025

Standardized Residuals (z-scores) for Covariances

SR12R2 SR12R3 SR13R1 SR13R2 SR13R3

________ ________ ________ ________ ________

SR12R2 0.698

SR12R3 -3.506 999.000

SR13R1 -0.466 -1.781 0.690

SR13R2 0.828 -0.878 -0.748 -1.230

SR13R3 1.922 1.091 0.601 0.129 1.078

SR14R1 -0.795 0.881 -0.197 -0.988 1.292

SR14R2 0.533 -0.562 -0.392 -0.328 -0.038

SR14R3 0.484 -0.952 0.127 0.601 1.667

Standardized Residuals (z-scores) for Covariances

SR14R1 SR14R2 SR14R3

________ ________ ________

SR14R1 -2.445

SR14R2 -0.542 -0.168

SR14R3 -0.422 -0.468 -0.691

Normalized Residuals for Covariances

IC12R1 IC12R2 IC12R3 IC13R1 IC13R2

________ ________ ________ ________ ________

IC12R1 1.210

IC12R2 2.594 2.379

IC12R3 2.223 2.515 1.548

IC13R1 2.955 3.521 4.479 -1.191

IC13R2 2.146 2.824 3.581 -1.664 -1.813

IC13R3 3.030 2.586 2.960 0.018 -0.524

IC14R1 2.569 4.182 3.893 3.505 4.481

IC14R2 2.885 4.174 3.049 4.786 5.497

IC14R3 1.686 2.907 3.182 2.649 4.580

SR12R1 -1.775 -2.763 -2.254 -2.202 -2.020

SR12R2 -2.061 -1.411 -2.536 -1.381 -2.168

SR12R3 -2.083 -1.143 -1.621 -1.475 -1.872

SR13R1 -1.021 -0.573 -0.522 0.092 0.369

SR13R2 -0.977 -2.573 -1.284 -0.685 0.334

SR13R3 -1.673 -2.782 -2.760 -2.618 -0.534

SR14R1 -1.401 -0.905 -2.422 -2.083 -0.262

SR14R2 0.140 -0.627 -1.419 -0.431 -0.700

SR14R3 0.143 -0.542 -1.411 -0.833 -0.459

Normalized Residuals for Covariances

IC13R3 IC14R1 IC14R2 IC14R3 SR12R1

________ ________ ________ ________ ________

IC13R3 0.489

IC14R1 2.733 0.324

IC14R2 4.947 -0.701 -1.442

IC14R3 3.845 -0.918 -1.818 -1.869

SR12R1 -2.097 -2.863 -1.538 -1.475 -0.819

SR12R2 -2.661 -3.191 -1.587 -1.747 -0.181

SR12R3 -2.712 -2.236 -0.937 -1.266 -1.162

SR13R1 0.191 -1.247 -1.614 -0.344 -0.728

SR13R2 -1.015 -2.102 -1.960 0.199 -0.975

SR13R3 -2.193 -1.737 -3.191 -1.030 -0.536

SR14R1 -1.922 -0.547 0.983 -0.090 0.235

SR14R2 -0.164 0.701 0.291 1.335 -0.070

SR14R3 -1.247 0.527 0.098 0.620 -0.021

Normalized Residuals for Covariances

SR12R2 SR12R3 SR13R1 SR13R2 SR13R3

________ ________ ________ ________ ________

SR12R2 0.332

SR12R3 -0.484 -0.896

SR13R1 -0.279 -1.100 0.443

SR13R2 0.547 -0.599 -0.395 -0.766

SR13R3 1.447 0.832 0.317 0.073 0.750

SR14R1 -0.542 0.583 -0.122 -0.632 0.962

SR14R2 0.420 -0.434 -0.216 -0.246 -0.031

SR14R3 0.393 -0.755 0.097 0.466 1.377

Normalized Residuals for Covariances

SR14R1 SR14R2 SR14R3

________ ________ ________

SR14R1 -1.397

SR14R2 -0.330 -0.131

SR14R3 -0.269 -0.301 -0.512

MODEL MODIFICATION INDICES

NOTE: Modification indices for direct effects of observed dependent variables

regressed on covariates may not be included. To include these, request

MODINDICES (ALL).

Minimum M.I. value for printing the modification index 5.000

M.I. E.P.C. Std E.P.C. StdYX E.P.C.

BY Statements

SR12 BY IC12R3 5.893 -0.171 -0.102 -0.097

SR12 BY SR12R1 10.616 -0.167 -0.100 -0.123

SR12 BY SR12R2 8.456 0.103 0.062 0.082

SR13 BY IC12R3 5.368 -0.177 -0.070 -0.067

SR13 BY SR12R1 11.570 -0.162 -0.064 -0.078

SR13 BY SR12R2 6.820 0.122 0.048 0.064

SR14 BY IC12R3 7.190 -0.221 -0.080 -0.076

SR14 BY SR12R1 8.660 -0.149 -0.053 -0.066

SR14 BY SR12R2 5.145 0.111 0.040 0.054

IC12 BY IC12R2 11.162 0.098 0.050 0.052

IC12 BY SR12R1 13.459 -0.172 -0.088 -0.108

IC12 BY SR13R3 6.317 -0.104 -0.053 -0.092

IC13 BY SR12R1 5.856 -0.156 -0.074 -0.091

IC13 BY SR13R3 7.040 -0.136 -0.065 -0.112

IC14 BY IC12R2 6.941 0.318 0.143 0.149

IC14 BY SR12R1 7.032 -0.223 -0.100 -0.123

DFIM1 BY IC12R2 5.116 -0.164 -0.123 -0.128

DFIM1 BY IC13R1 6.369 -0.145 -0.109 -0.148

DFIM1 BY IC13R3 6.143 -0.181 -0.136 -0.157

DFIM1 BY IC14R1 16.286 -0.280 -0.210 -0.292

DFIM1 BY SR13R3 7.891 0.113 0.085 0.147

DFIM2 BY IC12R3 14.055 -0.321 -0.202 -0.192

DFIM2 BY IC14R2 14.277 -0.227 -0.143 -0.196

DFIM2 BY SR12R2 7.007 0.135 0.085 0.114

DFIM3 BY IC12R3 8.961 -0.277 -0.174 -0.166

DFIM3 BY SR12R2 6.407 0.144 0.090 0.121

IDFIM BY IC14R1 6.030 -0.279 -0.140 -0.195

ON/BY Statements

SR12 ON SR12 /

SR12 BY SR12 7.185 -1.061 -1.061 -1.061

SR12 ON IC12 /

IC12 BY SR12 23.720 -0.733 -0.628 -0.628

SR12 ON IC13 /

IC13 BY SR12 11.246 -0.312 -0.249 -0.249

SR12 ON IC14 /

IC14 BY SR12 5.238 -0.251 -0.189 -0.189

SR12 ON DFIM1 /

DFIM1 BY SR12 17.508 0.684 0.859 0.859

SR12 ON DFIM2 /

DFIM2 BY SR12 8.753 0.258 0.271 0.271

SR12 ON DFIM3 /

DFIM3 BY SR12 5.081 0.209 0.220 0.220

IC12 ON SR12 /

SR12 BY IC12 24.384 -0.516 -0.602 -0.602

IC12 ON SR13 /

SR13 BY IC12 12.127 -0.410 -0.316 -0.316

IC12 ON SR14 /

SR14 BY IC12 6.397 -0.322 -0.226 -0.226

IC12 ON IC12 /

IC12 BY IC12 19.490 0.750 0.750 0.750

IC12 ON IC13 /

IC13 BY IC12 5.855 0.200 0.186 0.186

IC12 ON DFIM1 /

DFIM1 BY IC12 24.725 -0.338 -0.495 -0.495

IC12 ON DFIM2 /

DFIM2 BY IC12 9.115 -0.155 -0.190 -0.190

IC12 ON DFIM3 /

DFIM3 BY IC12 5.347 -0.139 -0.170 -0.170

IC12 ON IDFIM /

IDFIM BY IC12 22.871 -0.591 -0.579 -0.579

IC12 ON SDFIM /

SDFIM BY IC12 22.784 1.081 0.900 0.900

IC13 ON SR12 /

SR12 BY IC13 5.511 -0.132 -0.166 -0.166

IC13 ON IC12 /

IC12 BY IC13 9.038 0.244 0.262 0.262

IC13 ON DFIM1 /

DFIM1 BY IC13 7.152 -0.091 -0.143 -0.143

IC13 ON SDFIM /

SDFIM BY IC13 8.147 0.309 0.276 0.276

IC14 ON IC12 /

IC12 BY IC14 8.600 0.148 0.168 0.168

IC14 ON IC13 /

IC13 BY IC14 9.609 0.159 0.169 0.169

IC14 ON DFIM1 /

DFIM1 BY IC14 12.854 -0.137 -0.229 -0.229

IC14 ON DFIM2 /

DFIM2 BY IC14 12.465 -0.151 -0.212 -0.212

IC14 ON IDFIM /

IDFIM BY IC14 6.106 -0.178 -0.199 -0.199

DFIM1 ON SR12 /

SR12 BY DFIM1 7.185 -1.061 -0.845 -0.845

DFIM1 ON IC12 /

IC12 BY DFIM1 23.720 -0.733 -0.500 -0.500

DFIM1 ON IC13 /

IC13 BY DFIM1 11.246 -0.312 -0.198 -0.198

DFIM1 ON IC14 /

IC14 BY DFIM1 5.238 -0.251 -0.151 -0.151

DFIM1 ON DFIM1 /

DFIM1 BY DFIM1 17.508 0.684 0.684 0.684

DFIM1 ON DFIM2 /

DFIM2 BY DFIM1 8.753 0.258 0.216 0.216

DFIM1 ON DFIM3 /

DFIM3 BY DFIM1 5.081 0.209 0.175 0.175

IDFIM ON SR12 /

SR12 BY IDFIM 8.582 -1.167 -1.390 -1.390

IDFIM ON IC12 /

IC12 BY IDFIM 10.371 -0.185 -0.189 -0.189

IDFIM ON DFIM1 /

DFIM1 BY IDFIM 9.382 0.193 0.289 0.289

WITH Statements

SR12R1 WITH IC12R2 6.559 -0.071 -0.071 -0.191

SR13R3 WITH IC13R1 5.735 -0.040 -0.040 -0.157

IC12 WITH SR12 19.491 -0.175 999.000 999.000

IC12 WITH SR13 5.855 -0.047 999.000 999.000

IC13 WITH SR12 9.038 -0.057 999.000 999.000

DFIM1 WITH IC12 19.491 -0.133 -0.466 -0.466

DFIM1 WITH IC13 9.038 -0.057 -0.215 -0.215

DFIM2 WITH IC12 5.855 -0.047 -0.164 -0.164

IDFIM WITH IC12 10.094 -0.046 -0.179 -0.179

Means/Intercepts/Thresholds

[ SR12R1 ] 8.428 0.078 0.078 0.096

DIAGRAM INFORMATION

Use View Diagram under the Diagram menu in the Mplus Editor to view the diagram.

If running Mplus from the Mplus Diagrammer, the diagram opens automatically.

Diagram output

u:\windows\dcn r&r\r&r analyses\latent change score inspired\sr - ic lcs with multiple item indicators item intercep

Beginning Time: 15:30:50

Ending Time: 15:30:53

Elapsed Time: 00:00:03

MUTHEN & MUTHEN

3463 Stoner Ave.

Los Angeles, CA 90066

Tel: (310) 391-9971

Fax: (310) 391-8971

Web: www.StatModel.com

Support: Support@StatModel.com

Copyright (c) 1998-2018 Muthen & Muthen
