# Supplementary material for: Mind the gap: A review and recommendations for statistically evaluating Dual Systems models of adolescent risk behavior
Source: Dev Cogn Neurosci. 2019 Jul 25;39:100681. doi: 10.1016/j.dcn.2019.100681 (PMC6969358; doi:10.1016/j.dcn.2019.100681)
Supplement: Supplementary file 3 [file mmc3.docx]

Mplus VERSION 8.2

MUTHEN & MUTHEN

04/05/2019 3:37 PM

INPUT INSTRUCTIONS

Title: LDS Growth Model and Alcohol Two-Part Model

Data: file is predictR&R.dat;

Variable:

NAMES ARE

subject gender sr12r1 sr12r2 sr12r3 sr13r1 sr13r2 sr13r3

sr14r1 sr14r2 sr14r3 ic12r1 ic12r2 ic12r3 ic13r1 ic13r2

ic13r3 ic14r1 ic14r2 ic14r3 alcd12-alcd20 alcc12-alcc20;

!SR=sensitivity to reward, IC=inhibitory control

!12=age 12, 13=age 13, 14=age 14

!r1=response block 1, r2=response block 2, r3=response block 3

USEV

ic12r1-ic12r3 ic13r1-ic13r3 ic14r1-ic14r3

sr12r1-sr12r3 sr13r1-sr13r3 sr14r1-sr14r3

alcd12-alcd20 alcc12-alcc20 gender;

missing are .;

CATEGORICAL ARE alcd12-alcd20;

Define:

!Log-Transformed Continuous Alcohol Use

alcc12=log(alcc12+1);

alcc13=log(alcc13+1);

alcc14=log(alcc14+1);

alcc15=log(alcc15+1);

alcc16=log(alcc16+1);

alcc17=log(alcc17+1);

alcc18=log(alcc18+1);

alcc19=log(alcc19+1);

alcc20=log(alcc20+1);

Analysis:

estimator is MLR;

model=nocovariances;

mconvergence=.01;

Model:

!Sensitivity to Reward (PSRT) Measurement Model

sr12 by sr12r1

sr12r2 (sr2)

sr12r3 ;

sr13 by sr13r1

sr13r2 (sr2)

sr13r3 (sr3);

sr14 by sr14r1

sr14r2 (sr2)

sr14r3 (sr3);

!Constraining Item Intercepts Within and Across Time for

!Mean Structure Identification

[sr12r1 sr13r1 sr14r1] (sri1);

[sr12r2 sr13r2 sr14r2] (sri1);

[sr12r3 sr13r3 sr14r3] (sri1);

!Constraining Across time Item Residual Variances

sr13r1 sr14r1 (srr1);

sr12r2 sr13r2 sr14r2 (srr2);

sr13r3 sr14r3 (srr3);

!Stop Signal Task Measurment Model

ic12 by ic12r1

ic12r2 (ic2)

ic12r3 (ic3);

ic13 by ic13r1

ic13r2 (ic2)

ic13r3 (ic3);

ic14 by ic14r1

ic14r2 (ic2)

ic14r3 (ic3);

!Constraining Item Intercepts Within and Across Time for

!Mean Structure Identification

[ic12r1 ic13r1 ic14r1] (ici1);

[ic12r2 ic13r2 ic14r2] (ici1);

[ic12r3 ic13r3 ic14r3] (ici1);

!Constraining Across time Item Residual Variances

ic13r1 ic14r1 (icr1);

ic12r2 ic13r2 (icr2);

ic13r3 ic14r3 (icr3);

!Specifying Latent Difference Score

sr12 on ic12@1;

sr13 on ic13@1;

sr14 on ic14@1;

dfim1 by sr12@1;

dfim2 by sr13@1;

dfim3 by sr14@1;

!Means and Variances of Sensitivity to Reward Constrained to 0

!for Model Identification

sr12-sr14@0;

[sr12-sr14@0];

[ic12-ic14];

!Latent Difference Score Variances

dfim1 dfim2 dfim3 (dfimres);

!Constrained Change Score Intercepts for Growth Model

[dfim1@0 dfim2@0 dfim3@0];

!Covariance Between Difference Scores and Inhibitory Control

ic12 with dfim1 (icwdfim);

ic13 with dfim2 (icwdfim);

ic14 with dfim3 (icwdfim);

!Latent Growth of Difference Scores

idfim sdfim | dfim1@0 dfim2* dfim3@1;

idfim with sdfim;

!Covariances added based on Modification Indices

SR13R3 WITH SR13R1;

SR14R3 WITH SR14R2;

SR13R3 WITH SR13R2;

SR14R2 WITH SR13R1;

IC13 WITH IC12;

IC14R1 WITH IC12R1;

IC13R3 WITH IC13R1;

IC13R3 WITH IC12R2;

IC13R3 WITH IC12R3;

IC14R3 WITH IC12R1;

!Alchol Two-Part Growth Model With Random Effects

!Dichotomous Growth Model ages 12-20

id sd | alcd12@-1 alcd13@0 alcd14@1 alcd15* alcd16@3 alcd17@4

alcd18@5 alcd19@6 alcd20*;

sd BY alcd15*2.32557;

sd BY alcd20*6.22476;

!Continuous Growth Model ages 12-16

ic sc | alcc12@-1 alcc13@0 alcc14@1 alcc15@2 alcc16@3 alcc17@3

alcc18@3 alcc19@3 alcc20@3;

!Continuous Growth Model ages 16-20

ic sc2 | alcc12@0 alcc13@0 alcc14@0 alcc15@0 alcc16@0 alcc17@1

alcc18@2 alcc19* alcc20*;

sc2 BY alcc19*2.32512;

sc2 BY alcc20*2.39166;

!Covariances Between Growth Factors

id WITH sd*-0.72279;

id WITH ic*0.83417;

id WITH sc*0.25907;

id WITH sc2@0;

ic WITH sc*0.08127;

ic WITH sc2*-0.10838;

sc WITH sc2@0;

sd WITH ic@0;

sd WITH sc@0;

sd WITH sc2*0.16021;

!Continuous Alcohl Use Intercepts Constrained to 0 for Model

!Identification

[ alcc12@0 ];

[ alcc13@0 ];

[ alcc14@0 ];

[ alcc15@0 ];

[ alcc16@0 ];

[ alcc17@0 ];

[ alcc18@0 ];

[ alcc19@0 ];

[ alcc20@0 ];

[ id@0 ];

!Growth Factor Means Start Values

[ sd*0.97615 ];

[ ic*0.45873 ];

[ sc*0.41418 ];

[ sc2*1.03536 ];

!Dichotomous Alcohol Use Thresholds Start Values

[ alcd12$1*3.67237 ] (28);

[ alcd13$1*3.67237 ] (28);

[ alcd14$1*3.67237 ] (28);

[ alcd15$1*3.67237 ] (28);

[ alcd16$1*3.67237 ] (28);

[ alcd17$1*3.67237 ] (28);

[ alcd18$1*3.67237 ] (28);

[ alcd19$1*3.67237 ] (28);

[ alcd20$1*3.67237 ] (28);

!Variance of Continuous Alcohol Use Constrained to 0 Due

!to a Negative Residual Variance

alcc12@0;

!Start Values for Variances of Continous Alcohol Use and

!Alcohol Use Growth Factors

alcc13*0.43376;

alcc14*0.60803;

alcc15*0.93064;

alcc16*0.73305;

alcc17*1.55435;

alcc18*1.50926;

alcc19*0.81892;

alcc20*0.74486;

id*7.48873;

sd*0.30789;

ic*0.30434;

sc*0.04254;

sc2*0.35544;

!LDS Growth Model Covariances with Alcohol Use

id sd ic sc sc2 with ichim schim;

!Controlling for Gender

id sd ic sc sc2 ichim schim on gender;

Output: res stdyx tech4 sampstat svalues;

*** WARNING

Data set contains cases with missing on all variables except

x-variables. These cases were not included in the analysis.

Number of cases with missing on all variables except x-variables: 4

1 WARNING(S) FOUND IN THE INPUT INSTRUCTIONS

Revised Classes Predicting Alcohol 2-Part DCN R&R

SUMMARY OF ANALYSIS

Number of groups 1

Number of observations 383

Number of dependent variables 36

Number of independent variables 1

Number of continuous latent variables 16

Observed dependent variables

Continuous

ALCC12 ALCC13 ALCC14 ALCC15 ALCC16 ALCC17

ALCC18 ALCC19 ALCC20 IC12R1 IC12R2 IC12R3

IC13R1 IC13R2 IC13R3 IC14R1 IC14R2 IC14R3

SR12R1 SR12R2 SR12R3 SR13R1 SR13R2 SR13R3

SR14R1 SR14R2 SR14R3

Binary and ordered categorical (ordinal)

ALCD12 ALCD13 ALCD14 ALCD15 ALCD16 ALCD17

ALCD18 ALCD19 ALCD20

Observed independent variables

GENDER

Continuous latent variables

SR12 SR13 SR14 IC12 IC13 IC14

DFIM1 DFIM2 DFIM3 SD SC2 IDFIM

SDFIM ID IC SC

Estimator MLR

Information matrix OBSERVED

Optimization Specifications for the Quasi-Newton Algorithm for

Continuous Outcomes

Maximum number of iterations 100

Convergence criterion 0.100D-05

Optimization Specifications for the EM Algorithm

Maximum number of iterations 500

Convergence criteria

Loglikelihood change 0.100D-02

Relative loglikelihood change 0.100D-05

Derivative 0.100D-02

Optimization Specifications for the M step of the EM Algorithm for

Categorical Latent variables

Number of M step iterations 1

M step convergence criterion 0.100D-02

Basis for M step termination ITERATION

Optimization Specifications for the M step of the EM Algorithm for

Censored, Binary or Ordered Categorical (Ordinal), Unordered

Categorical (Nominal) and Count Outcomes

Number of M step iterations 1

M step convergence criterion 0.100D-02

Basis for M step termination ITERATION

Maximum value for logit thresholds 15

Minimum value for logit thresholds -15

Minimum expected cell size for chi-square 0.100D-01

Maximum number of iterations for H1 2000

Convergence criterion for H1 0.100D-03

Optimization algorithm EMA

Integration Specifications

Type STANDARD

Number of integration points 15

Dimensions of numerical integration 2

Adaptive quadrature ON

Link LOGIT

Cholesky OFF

Input data file(s)

predictR&R.dat

Input data format FREE

SUMMARY OF DATA

Number of missing data patterns 269

Number of y missing data patterns 223

Number of u missing data patterns 64

COVARIANCE COVERAGE OF DATA

Minimum covariance coverage value 0.100

PROPORTION OF DATA PRESENT

Covariance Coverage

ALCD12 ALCD13 ALCD14 ALCD15 ALCD16

________ ________ ________ ________ ________

ALCD12 0.935

ALCD13 0.872 0.932

ALCD14 0.898 0.909 0.958

ALCD15 0.849 0.867 0.883 0.903

ALCD16 0.820 0.833 0.849 0.822 0.875

ALCD17 0.757 0.762 0.775 0.749 0.744

ALCD18 0.726 0.728 0.742 0.721 0.697

ALCD19 0.773 0.786 0.809 0.778 0.762

ALCD20 0.590 0.603 0.616 0.598 0.587

ALCC12 0.039 0.031 0.037 0.034 0.029

ALCC13 0.102 0.117 0.115 0.112 0.102

ALCC14 0.193 0.198 0.209 0.204 0.180

ALCC15 0.295 0.300 0.311 0.319 0.287

ALCC16 0.347 0.355 0.360 0.360 0.376

ALCC17 0.384 0.399 0.397 0.402 0.392

ALCC18 0.460 0.467 0.473 0.465 0.452

ALCC19 0.564 0.567 0.587 0.567 0.556

ALCC20 0.446 0.457 0.465 0.454 0.454

IC12R1 0.885 0.830 0.854 0.807 0.773

IC12R2 0.880 0.825 0.851 0.799 0.768

IC12R3 0.877 0.822 0.846 0.796 0.765

IC13R1 0.815 0.833 0.828 0.781 0.749

IC13R2 0.820 0.836 0.825 0.783 0.755

IC13R3 0.820 0.838 0.828 0.783 0.757

IC14R1 0.473 0.470 0.480 0.457 0.428

IC14R2 0.480 0.478 0.488 0.465 0.433

IC14R3 0.478 0.475 0.486 0.460 0.431

SR12R1 0.906 0.851 0.875 0.822 0.794

SR12R2 0.906 0.851 0.875 0.822 0.794

SR12R3 0.906 0.851 0.875 0.822 0.794

SR13R1 0.856 0.875 0.864 0.820 0.789

SR13R2 0.856 0.875 0.864 0.820 0.789

SR13R3 0.856 0.875 0.864 0.820 0.789

SR14R1 0.499 0.496 0.507 0.480 0.452

SR14R2 0.499 0.496 0.507 0.480 0.452

SR14R3 0.499 0.496 0.507 0.480 0.452

GENDER 0.935 0.932 0.958 0.903 0.875

Covariance Coverage

ALCD17 ALCD18 ALCD19 ALCD20 ALCC12

________ ________ ________ ________ ________

ALCD17 0.802

ALCD18 0.637 0.765

ALCD19 0.700 0.666 0.822

ALCD20 0.504 0.480 0.551 0.634

ALCC12 0.034 0.029 0.031 0.018 0.039

ALCC13 0.078 0.089 0.091 0.076 0.023

ALCC14 0.151 0.151 0.175 0.154 0.031

ALCC15 0.238 0.240 0.266 0.227 0.029

ALCC16 0.300 0.295 0.332 0.272 0.029

ALCC17 0.415 0.326 0.363 0.272 0.018

ALCC18 0.415 0.488 0.420 0.313 0.026

ALCC19 0.504 0.470 0.593 0.405 0.023

ALCC20 0.384 0.360 0.420 0.480 0.016

IC12R1 0.715 0.687 0.731 0.567 0.034

IC12R2 0.708 0.676 0.726 0.556 0.037

IC12R3 0.708 0.679 0.723 0.559 0.034

IC13R1 0.687 0.668 0.713 0.538 0.031

IC13R2 0.687 0.674 0.713 0.543 0.034

IC13R3 0.692 0.676 0.715 0.546 0.034

IC14R1 0.423 0.355 0.402 0.436 0.016

IC14R2 0.428 0.358 0.410 0.441 0.018

IC14R3 0.426 0.350 0.407 0.439 0.016

SR12R1 0.731 0.702 0.749 0.574 0.037

SR12R2 0.731 0.702 0.749 0.574 0.037

SR12R3 0.731 0.702 0.749 0.574 0.037

SR13R1 0.718 0.700 0.747 0.569 0.034

SR13R2 0.718 0.700 0.747 0.569 0.034

SR13R3 0.718 0.700 0.747 0.569 0.034

SR14R1 0.444 0.371 0.428 0.460 0.018

SR14R2 0.444 0.371 0.428 0.460 0.018

SR14R3 0.444 0.371 0.428 0.460 0.018

GENDER 0.802 0.765 0.822 0.634 0.039

Covariance Coverage

ALCC13 ALCC14 ALCC15 ALCC16 ALCC17

________ ________ ________ ________ ________

ALCC13 0.117

ALCC14 0.073 0.209

ALCC15 0.089 0.154 0.319

ALCC16 0.076 0.133 0.238 0.376

ALCC17 0.055 0.117 0.201 0.248 0.415

ALCC18 0.073 0.123 0.196 0.243 0.282

ALCC19 0.073 0.144 0.230 0.285 0.319

ALCC20 0.068 0.128 0.201 0.238 0.243

IC12R1 0.097 0.185 0.290 0.324 0.366

IC12R2 0.097 0.185 0.285 0.329 0.360

IC12R3 0.097 0.180 0.272 0.313 0.350

IC13R1 0.102 0.180 0.272 0.319 0.360

IC13R2 0.107 0.180 0.274 0.324 0.358

IC13R3 0.104 0.183 0.264 0.319 0.355

IC14R1 0.052 0.117 0.172 0.185 0.230

IC14R2 0.057 0.123 0.180 0.183 0.232

IC14R3 0.055 0.120 0.175 0.183 0.235

SR12R1 0.097 0.191 0.287 0.337 0.368

SR12R2 0.097 0.191 0.287 0.337 0.368

SR12R3 0.097 0.191 0.287 0.337 0.368

SR13R1 0.110 0.188 0.282 0.332 0.366

SR13R2 0.110 0.188 0.282 0.332 0.366

SR13R3 0.110 0.188 0.282 0.332 0.366

SR14R1 0.057 0.125 0.183 0.193 0.240

SR14R2 0.057 0.125 0.183 0.193 0.240

SR14R3 0.057 0.125 0.183 0.193 0.240

GENDER 0.117 0.209 0.319 0.376 0.415

Covariance Coverage

ALCC18 ALCC19 ALCC20 IC12R1 IC12R2

________ ________ ________ ________ ________

ALCC18 0.488

ALCC19 0.379 0.593

ALCC20 0.292 0.355 0.480

IC12R1 0.433 0.533 0.423 0.885

IC12R2 0.433 0.533 0.418 0.856 0.880

IC12R3 0.426 0.522 0.418 0.854 0.854

IC13R1 0.433 0.527 0.410 0.786 0.786

IC13R2 0.439 0.527 0.420 0.789 0.786

IC13R3 0.439 0.525 0.418 0.789 0.789

IC14R1 0.240 0.303 0.332 0.462 0.457

IC14R2 0.245 0.313 0.337 0.470 0.465

IC14R3 0.235 0.303 0.326 0.467 0.462

SR12R1 0.441 0.546 0.431 0.880 0.875

SR12R2 0.441 0.546 0.431 0.880 0.875

SR12R3 0.441 0.546 0.431 0.880 0.875

SR13R1 0.446 0.546 0.431 0.822 0.817

SR13R2 0.446 0.546 0.431 0.822 0.817

SR13R3 0.446 0.546 0.431 0.822 0.817

SR14R1 0.253 0.324 0.345 0.488 0.483

SR14R2 0.253 0.324 0.345 0.488 0.483

SR14R3 0.253 0.324 0.345 0.488 0.483

GENDER 0.488 0.593 0.480 0.885 0.880

Covariance Coverage

IC12R3 IC13R1 IC13R2 IC13R3 IC14R1

________ ________ ________ ________ ________

IC12R3 0.877

IC13R1 0.775 0.838

IC13R2 0.781 0.812 0.841

IC13R3 0.783 0.812 0.822 0.843

IC14R1 0.457 0.449 0.454 0.454 0.483

IC14R2 0.465 0.457 0.457 0.460 0.473

IC14R3 0.465 0.454 0.454 0.457 0.470

SR12R1 0.872 0.802 0.807 0.807 0.467

SR12R2 0.872 0.802 0.807 0.807 0.467

SR12R3 0.872 0.802 0.807 0.807 0.467

SR13R1 0.815 0.836 0.838 0.841 0.467

SR13R2 0.815 0.836 0.838 0.841 0.467

SR13R3 0.815 0.836 0.838 0.841 0.467

SR14R1 0.483 0.475 0.475 0.478 0.483

SR14R2 0.483 0.475 0.475 0.478 0.483

SR14R3 0.483 0.475 0.475 0.478 0.483

GENDER 0.877 0.838 0.841 0.843 0.483

Covariance Coverage

IC14R2 IC14R3 SR12R1 SR12R2 SR12R3

________ ________ ________ ________ ________

IC14R2 0.491

IC14R3 0.473 0.488

SR12R1 0.475 0.473 0.906

SR12R2 0.475 0.473 0.906 0.906

SR12R3 0.475 0.473 0.906 0.906 0.906

SR13R1 0.475 0.473 0.843 0.843 0.843

SR13R2 0.475 0.473 0.843 0.843 0.843

SR13R3 0.475 0.473 0.843 0.843 0.843

SR14R1 0.491 0.488 0.493 0.493 0.493

SR14R2 0.491 0.488 0.493 0.493 0.493

SR14R3 0.491 0.488 0.493 0.493 0.493

GENDER 0.491 0.488 0.906 0.906 0.906

Covariance Coverage

SR13R1 SR13R2 SR13R3 SR14R1 SR14R2

________ ________ ________ ________ ________

SR13R1 0.880

SR13R2 0.880 0.880

SR13R3 0.880 0.880 0.880

SR14R1 0.493 0.493 0.493 0.509

SR14R2 0.493 0.493 0.493 0.509 0.509

SR14R3 0.493 0.493 0.493 0.509 0.509

GENDER 0.880 0.880 0.880 0.509 0.509

Covariance Coverage

SR14R3 GENDER

________ ________

SR14R3 0.509

GENDER 0.509 1.000

WARNING: THE COVARIANCE COVERAGE FALLS BELOW THE SPECIFIED LIMIT.

PROPORTION OF DATA PRESENT FOR U

Covariance Coverage

ALCD12 ALCD13 ALCD14 ALCD15 ALCD16

________ ________ ________ ________ ________

ALCD12 0.935

ALCD13 0.872 0.932

ALCD14 0.898 0.909 0.958

ALCD15 0.849 0.867 0.883 0.903

ALCD16 0.820 0.833 0.849 0.822 0.875

ALCD17 0.757 0.762 0.775 0.749 0.744

ALCD18 0.726 0.728 0.742 0.721 0.697

ALCD19 0.773 0.786 0.809 0.778 0.762

ALCD20 0.590 0.603 0.616 0.598 0.587

Covariance Coverage

ALCD17 ALCD18 ALCD19 ALCD20

________ ________ ________ ________

ALCD17 0.802

ALCD18 0.637 0.765

ALCD19 0.700 0.666 0.822

ALCD20 0.504 0.480 0.551 0.634

PROPORTION OF DATA PRESENT FOR Y

Covariance Coverage

ALCC12 ALCC13 ALCC14 ALCC15 ALCC16

________ ________ ________ ________ ________

ALCC12 0.039

ALCC13 0.023 0.117

ALCC14 0.031 0.073 0.209

ALCC15 0.029 0.089 0.154 0.319

ALCC16 0.029 0.076 0.133 0.238 0.376

ALCC17 0.018 0.055 0.117 0.201 0.248

ALCC18 0.026 0.073 0.123 0.196 0.243

ALCC19 0.023 0.073 0.144 0.230 0.285

ALCC20 0.016 0.068 0.128 0.201 0.238

IC12R1 0.034 0.097 0.185 0.290 0.324

IC12R2 0.037 0.097 0.185 0.285 0.329

IC12R3 0.034 0.097 0.180 0.272 0.313

IC13R1 0.031 0.102 0.180 0.272 0.319

IC13R2 0.034 0.107 0.180 0.274 0.324

IC13R3 0.034 0.104 0.183 0.264 0.319

IC14R1 0.016 0.052 0.117 0.172 0.185

IC14R2 0.018 0.057 0.123 0.180 0.183

IC14R3 0.016 0.055 0.120 0.175 0.183

SR12R1 0.037 0.097 0.191 0.287 0.337

SR12R2 0.037 0.097 0.191 0.287 0.337

SR12R3 0.037 0.097 0.191 0.287 0.337

SR13R1 0.034 0.110 0.188 0.282 0.332

SR13R2 0.034 0.110 0.188 0.282 0.332

SR13R3 0.034 0.110 0.188 0.282 0.332

SR14R1 0.018 0.057 0.125 0.183 0.193

SR14R2 0.018 0.057 0.125 0.183 0.193

SR14R3 0.018 0.057 0.125 0.183 0.193

GENDER 0.039 0.117 0.209 0.319 0.376

Covariance Coverage

ALCC17 ALCC18 ALCC19 ALCC20 IC12R1

________ ________ ________ ________ ________

ALCC17 0.415

ALCC18 0.282 0.488

ALCC19 0.319 0.379 0.593

ALCC20 0.243 0.292 0.355 0.480

IC12R1 0.366 0.433 0.533 0.423 0.885

IC12R2 0.360 0.433 0.533 0.418 0.856

IC12R3 0.350 0.426 0.522 0.418 0.854

IC13R1 0.360 0.433 0.527 0.410 0.786

IC13R2 0.358 0.439 0.527 0.420 0.789

IC13R3 0.355 0.439 0.525 0.418 0.789

IC14R1 0.230 0.240 0.303 0.332 0.462

IC14R2 0.232 0.245 0.313 0.337 0.470

IC14R3 0.235 0.235 0.303 0.326 0.467

SR12R1 0.368 0.441 0.546 0.431 0.880

SR12R2 0.368 0.441 0.546 0.431 0.880

SR12R3 0.368 0.441 0.546 0.431 0.880

SR13R1 0.366 0.446 0.546 0.431 0.822

SR13R2 0.366 0.446 0.546 0.431 0.822

SR13R3 0.366 0.446 0.546 0.431 0.822

SR14R1 0.240 0.253 0.324 0.345 0.488

SR14R2 0.240 0.253 0.324 0.345 0.488

SR14R3 0.240 0.253 0.324 0.345 0.488

GENDER 0.415 0.488 0.593 0.480 0.885

Covariance Coverage

IC12R2 IC12R3 IC13R1 IC13R2 IC13R3

________ ________ ________ ________ ________

IC12R2 0.880

IC12R3 0.854 0.877

IC13R1 0.786 0.775 0.838

IC13R2 0.786 0.781 0.812 0.841

IC13R3 0.789 0.783 0.812 0.822 0.843

IC14R1 0.457 0.457 0.449 0.454 0.454

IC14R2 0.465 0.465 0.457 0.457 0.460

IC14R3 0.462 0.465 0.454 0.454 0.457

SR12R1 0.875 0.872 0.802 0.807 0.807

SR12R2 0.875 0.872 0.802 0.807 0.807

SR12R3 0.875 0.872 0.802 0.807 0.807

SR13R1 0.817 0.815 0.836 0.838 0.841

SR13R2 0.817 0.815 0.836 0.838 0.841

SR13R3 0.817 0.815 0.836 0.838 0.841

SR14R1 0.483 0.483 0.475 0.475 0.478

SR14R2 0.483 0.483 0.475 0.475 0.478

SR14R3 0.483 0.483 0.475 0.475 0.478

GENDER 0.880 0.877 0.838 0.841 0.843

Covariance Coverage

IC14R1 IC14R2 IC14R3 SR12R1 SR12R2

________ ________ ________ ________ ________

IC14R1 0.483

IC14R2 0.473 0.491

IC14R3 0.470 0.473 0.488

SR12R1 0.467 0.475 0.473 0.906

SR12R2 0.467 0.475 0.473 0.906 0.906

SR12R3 0.467 0.475 0.473 0.906 0.906

SR13R1 0.467 0.475 0.473 0.843 0.843

SR13R2 0.467 0.475 0.473 0.843 0.843

SR13R3 0.467 0.475 0.473 0.843 0.843

SR14R1 0.483 0.491 0.488 0.493 0.493

SR14R2 0.483 0.491 0.488 0.493 0.493

SR14R3 0.483 0.491 0.488 0.493 0.493

GENDER 0.483 0.491 0.488 0.906 0.906

Covariance Coverage

SR12R3 SR13R1 SR13R2 SR13R3 SR14R1

________ ________ ________ ________ ________

SR12R3 0.906

SR13R1 0.843 0.880

SR13R2 0.843 0.880 0.880

SR13R3 0.843 0.880 0.880 0.880

SR14R1 0.493 0.493 0.493 0.493 0.509

SR14R2 0.493 0.493 0.493 0.493 0.509

SR14R3 0.493 0.493 0.493 0.493 0.509

GENDER 0.906 0.880 0.880 0.880 0.509

Covariance Coverage

SR14R2 SR14R3 GENDER

________ ________ ________

SR14R2 0.509

SR14R3 0.509 0.509

GENDER 0.509 0.509 1.000

THE COVARIANCE COVERAGE FALLS BELOW THE SPECIFIED LIMIT. THE MISSING

DATA EM ALGORITHM WILL NOT BE INITIATED. CHECK YOUR DATA OR LOWER THE

COVARIANCE COVERAGE LIMIT.

UNIVARIATE PROPORTIONS AND COUNTS FOR CATEGORICAL VARIABLES

ALCD12

Category 1 0.958 343.000

Category 2 0.042 15.000

ALCD13

Category 1 0.874 312.000

Category 2 0.126 45.000

ALCD14

Category 1 0.782 287.000

Category 2 0.218 80.000

ALCD15

Category 1 0.647 224.000

Category 2 0.353 122.000

ALCD16

Category 1 0.570 191.000

Category 2 0.430 144.000

ALCD17

Category 1 0.482 148.000

Category 2 0.518 159.000

ALCD18

Category 1 0.362 106.000

Category 2 0.638 187.000

ALCD19

Category 1 0.279 88.000

Category 2 0.721 227.000

ALCD20

Category 1 0.243 59.000

Category 2 0.757 184.000

SAMPLE STATISTICS

UNIVARIATE SAMPLE STATISTICS

UNIVARIATE HIGHER-ORDER MOMENT DESCRIPTIVE STATISTICS

Variable/ Mean/ Skewness/ Minimum/ % with Percentiles

Sample Size Variance Kurtosis Maximum Min/Max 20%/60% 40%/80% Median

ALCC12 0.557 1.534 0.223 40.00% 0.223 0.223 0.405

15.000 0.205 1.299 1.792 6.67% 0.405 0.693

ALCC13 0.812 1.805 0.010 2.22% 0.223 0.405 0.560

45.000 0.625 2.757 3.453 2.22% 0.693 1.099

ALCC14 1.383 1.212 0.223 16.25% 0.405 0.916 1.099

80.000 1.224 1.144 4.732 1.25% 1.609 2.140

ALCC15 1.981 0.625 0.405 11.48% 0.693 1.386 1.748

122.000 1.586 -0.498 5.347 0.82% 2.197 3.045

ALCC16 2.242 0.528 0.405 11.81% 0.693 1.609 2.197

144.000 2.036 -0.584 5.707 0.69% 2.565 3.434

ALCC17 3.238 0.261 0.405 3.14% 1.792 2.565 3.045

159.000 2.611 -0.580 7.463 0.63% 3.584 4.691

ALCC18 4.431 -0.273 0.693 0.53% 2.944 4.111 4.654

187.000 2.801 -0.535 7.463 1.60% 5.017 5.666

ALCC19 4.540 -0.410 1.099 6.17% 3.497 4.290 4.691

227.000 2.685 -0.490 7.353 0.88% 5.056 6.033

ALCC20 4.684 -0.388 1.099 4.89% 3.611 4.290 4.702

184.000 2.479 -0.356 7.354 0.54% 5.198 6.151

IC12R1 -0.031 -0.868 -3.200 1.77% -0.707 -0.139 0.033

339.000 1.075 1.081 2.044 0.29% 0.280 0.813

IC12R2 -0.289 -1.156 -3.608 2.37% -0.893 -0.301 -0.146

337.000 1.117 1.608 1.869 0.30% 0.064 0.493

IC12R3 -0.217 -0.780 -3.661 0.89% -0.958 -0.280 -0.093

336.000 1.217 0.773 2.062 0.30% 0.163 0.579

IC13R1 0.325 -0.243 -2.001 0.31% -0.232 0.170 0.304

321.000 0.488 0.381 2.041 0.31% 0.473 0.906

IC13R2 0.184 -0.651 -2.845 0.93% -0.449 0.077 0.272

322.000 0.736 1.178 1.986 0.31% 0.411 0.844

IC13R3 0.117 -0.854 -2.763 2.17% -0.452 -0.005 0.211

323.000 0.767 1.336 1.835 0.31% 0.383 0.789

IC14R1 0.369 -0.628 -1.986 1.08% -0.129 0.229 0.473

185.000 0.527 1.211 2.041 0.54% 0.610 0.875

IC14R2 0.308 -0.615 -2.243 1.06% -0.201 0.177 0.299

188.000 0.452 1.503 1.728 0.53% 0.442 0.910

IC14R3 0.215 -0.497 -2.340 0.53% -0.373 0.070 0.246

187.000 0.586 1.149 2.065 0.53% 0.390 0.889

SR12R1 0.670 1.169 -1.982 0.29% 0.127 0.430 0.532

347.000 0.627 4.247 4.720 0.29% 0.707 1.148

SR12R2 0.574 0.663 -1.727 0.29% 0.033 0.341 0.469

347.000 0.572 1.746 3.573 0.29% 0.645 1.110

SR12R3 0.624 0.370 -1.837 0.29% 0.107 0.433 0.534

347.000 0.532 1.939 4.103 0.29% 0.699 1.104

SR13R1 0.482 0.836 -1.012 0.30% 0.115 0.300 0.406

337.000 0.292 1.992 2.863 0.30% 0.512 0.837

SR13R2 0.506 0.832 -1.550 0.30% 0.109 0.310 0.420

337.000 0.335 1.781 2.906 0.30% 0.503 0.907

SR13R3 0.597 1.181 -0.879 0.30% 0.160 0.379 0.479

337.000 0.355 2.585 3.236 0.30% 0.643 0.964

SR14R1 0.526 0.815 -0.715 0.51% 0.185 0.346 0.468

195.000 0.221 1.999 2.509 0.51% 0.581 0.854

SR14R2 0.574 0.953 -0.657 0.51% 0.131 0.366 0.488

195.000 0.325 1.837 2.885 0.51% 0.648 0.975

SR14R3 0.623 0.766 -0.823 0.51% 0.189 0.410 0.581

195.000 0.300 1.074 2.729 0.51% 0.687 0.994

GENDER 0.446 0.215 0.000 55.35% 0.000 0.000 0.000

383.000 0.247 -1.954 1.000 44.65% 1.000 1.000

THE MODEL ESTIMATION TERMINATED NORMALLY

MODEL FIT INFORMATION

Number of Free Parameters 86

Loglikelihood

H0 Value -8320.479

H0 Scaling Correction Factor 1.2168

for MLR

Information Criteria

Akaike (AIC) 16812.958

Bayesian (BIC) 17152.489

Sample-Size Adjusted BIC 16879.625

(n* = (n + 2) / 24)

MODEL RESULTS

Two-Tailed

Estimate S.E. Est./S.E. P-Value

ID |

ALCD12 1.000 0.000 999.000 999.000

ALCD13 1.000 0.000 999.000 999.000

ALCD14 1.000 0.000 999.000 999.000

ALCD15 1.000 0.000 999.000 999.000

ALCD16 1.000 0.000 999.000 999.000

ALCD17 1.000 0.000 999.000 999.000

ALCD18 1.000 0.000 999.000 999.000

ALCD19 1.000 0.000 999.000 999.000

ALCD20 1.000 0.000 999.000 999.000

SD |

ALCD12 -1.000 0.000 999.000 999.000

ALCD13 0.000 0.000 999.000 999.000

ALCD14 1.000 0.000 999.000 999.000

ALCD15 2.349 0.145 16.210 0.000

ALCD16 3.000 0.000 999.000 999.000

ALCD17 4.000 0.000 999.000 999.000

ALCD18 5.000 0.000 999.000 999.000

ALCD19 6.000 0.000 999.000 999.000

ALCD20 6.219 0.372 16.703 0.000

IC |

ALCC12 1.000 0.000 999.000 999.000

ALCC13 1.000 0.000 999.000 999.000

ALCC14 1.000 0.000 999.000 999.000

ALCC15 1.000 0.000 999.000 999.000

ALCC16 1.000 0.000 999.000 999.000

ALCC17 1.000 0.000 999.000 999.000

ALCC18 1.000 0.000 999.000 999.000

ALCC19 1.000 0.000 999.000 999.000

ALCC20 1.000 0.000 999.000 999.000

SC |

ALCC12 -1.000 0.000 999.000 999.000

ALCC13 0.000 0.000 999.000 999.000

ALCC14 1.000 0.000 999.000 999.000

ALCC15 2.000 0.000 999.000 999.000

ALCC16 3.000 0.000 999.000 999.000

ALCC17 3.000 0.000 999.000 999.000

ALCC18 3.000 0.000 999.000 999.000

ALCC19 3.000 0.000 999.000 999.000

ALCC20 3.000 0.000 999.000 999.000

SC2 |

ALCC12 0.000 0.000 999.000 999.000

ALCC13 0.000 0.000 999.000 999.000

ALCC14 0.000 0.000 999.000 999.000

ALCC15 0.000 0.000 999.000 999.000

ALCC16 0.000 0.000 999.000 999.000

ALCC17 1.000 0.000 999.000 999.000

ALCC18 2.000 0.000 999.000 999.000

ALCC19 2.315 0.100 23.061 0.000

ALCC20 2.439 0.111 21.913 0.000

IDFIM |

DFIM1 1.000 0.000 999.000 999.000

DFIM2 1.000 0.000 999.000 999.000

DFIM3 1.000 0.000 999.000 999.000

SDFIM |

DFIM1 0.000 0.000 999.000 999.000

DFIM2 0.904 0.121 7.440 0.000

DFIM3 1.000 0.000 999.000 999.000

SR12 BY

SR12R1 1.000 0.000 999.000 999.000

SR12R2 1.014 0.029 34.882 0.000

SR12R3 0.948 0.053 17.909 0.000

SR13 BY

SR13R1 1.000 0.000 999.000 999.000

SR13R2 1.014 0.029 34.882 0.000

SR13R3 0.916 0.036 25.481 0.000

SR14 BY

SR14R1 1.000 0.000 999.000 999.000

SR14R2 1.014 0.029 34.882 0.000

SR14R3 0.916 0.036 25.481 0.000

IC12 BY

IC12R1 1.000 0.000 999.000 999.000

IC12R2 1.332 0.148 9.016 0.000

IC12R3 1.369 0.141 9.705 0.000

IC13 BY

IC13R1 1.000 0.000 999.000 999.000

IC13R2 1.332 0.148 9.016 0.000

IC13R3 1.369 0.141 9.705 0.000

IC14 BY

IC14R1 1.000 0.000 999.000 999.000

IC14R2 1.332 0.148 9.016 0.000

IC14R3 1.369 0.141 9.705 0.000

DFIM1 BY

SR12 1.000 0.000 999.000 999.000

DFIM2 BY

SR13 1.000 0.000 999.000 999.000

DFIM3 BY

SR14 1.000 0.000 999.000 999.000

SR12 ON

IC12 1.000 0.000 999.000 999.000

SR13 ON

IC13 1.000 0.000 999.000 999.000

SR14 ON

IC14 1.000 0.000 999.000 999.000

ID ON

GENDER -0.360 0.380 -0.947 0.344

SD ON

GENDER 0.037 0.090 0.408 0.683

IC ON

GENDER -0.037 0.134 -0.276 0.783

SC ON

GENDER 0.026 0.063 0.417 0.677

SC2 ON

GENDER 0.251 0.103 2.433 0.015

IDFIM ON

GENDER 0.227 0.071 3.185 0.001

SDFIM ON

GENDER -0.047 0.074 -0.633 0.527

IC12 WITH

DFIM1 -0.236 0.052 -4.506 0.000

IC13 0.025 0.010 2.501 0.012

IC13 WITH

DFIM2 -0.236 0.052 -4.506 0.000

IC14 WITH

DFIM3 -0.236 0.052 -4.506 0.000

IDFIM WITH

SDFIM -0.175 0.041 -4.215 0.000

ID 0.133 0.118 1.128 0.259

SD -0.012 0.033 -0.351 0.726

IC 0.008 0.049 0.163 0.871

SC 0.028 0.025 1.102 0.270

SC2 -0.016 0.033 -0.494 0.621

ID WITH

SD -0.558 0.267 -2.095 0.036

IC 0.702 0.421 1.669 0.095

SC 0.324 0.155 2.093 0.036

SC2 0.000 0.000 999.000 999.000

SDFIM -0.089 0.126 -0.705 0.481

IC WITH

SC 0.083 0.013 6.478 0.000

SC2 -0.119 0.059 -2.035 0.042

SD 0.000 0.000 999.000 999.000

SDFIM -0.005 0.052 -0.101 0.919

SC WITH

SC2 0.000 0.000 999.000 999.000

SD 0.000 0.000 999.000 999.000

SDFIM -0.026 0.024 -1.080 0.280

SD WITH

SC2 0.133 0.038 3.509 0.000

SDFIM -0.002 0.034 -0.061 0.951

SC2 WITH

SDFIM 0.039 0.034 1.127 0.260

SR13R3 WITH

SR13R1 -0.045 0.018 -2.465 0.014

SR13R2 0.064 0.024 2.732 0.006

IC14R3 WITH

IC12R1 0.137 0.040 3.444 0.001

SR14R3 WITH

SR14R2 0.077 0.020 3.951 0.000

IC14R1 WITH

IC12R1 0.083 0.032 2.603 0.009

IC13R3 WITH

IC13R1 -0.099 0.042 -2.353 0.019

IC12R2 0.110 0.047 2.319 0.020

IC12R3 0.098 0.042 2.345 0.019

SR14R2 WITH

SR13R1 -0.029 0.013 -2.151 0.031

Means

IC12 -0.747 0.236 -3.171 0.002

IC13 -0.433 0.208 -2.081 0.037

IC14 -0.345 0.204 -1.688 0.091

Intercepts

ALCC12 0.000 0.000 999.000 999.000

ALCC13 0.000 0.000 999.000 999.000

ALCC14 0.000 0.000 999.000 999.000

ALCC15 0.000 0.000 999.000 999.000

ALCC16 0.000 0.000 999.000 999.000

ALCC17 0.000 0.000 999.000 999.000

ALCC18 0.000 0.000 999.000 999.000

ALCC19 0.000 0.000 999.000 999.000

ALCC20 0.000 0.000 999.000 999.000

IC12R1 0.751 0.216 3.486 0.000

IC12R2 0.751 0.216 3.486 0.000

IC12R3 0.751 0.216 3.486 0.000

IC13R1 0.751 0.216 3.486 0.000

IC13R2 0.751 0.216 3.486 0.000

IC13R3 0.751 0.216 3.486 0.000

IC14R1 0.751 0.216 3.486 0.000

IC14R2 0.751 0.216 3.486 0.000

IC14R3 0.751 0.216 3.486 0.000

SR12R1 1.263 0.237 5.319 0.000

SR12R2 1.263 0.237 5.319 0.000

SR12R3 1.263 0.237 5.319 0.000

SR13R1 1.263 0.237 5.319 0.000

SR13R2 1.263 0.237 5.319 0.000

SR13R3 1.263 0.237 5.319 0.000

SR14R1 1.263 0.237 5.319 0.000

SR14R2 1.263 0.237 5.319 0.000

SR14R3 1.263 0.237 5.319 0.000

SR12 0.000 0.000 999.000 999.000

SR13 0.000 0.000 999.000 999.000

SR14 0.000 0.000 999.000 999.000

DFIM1 0.000 0.000 999.000 999.000

DFIM2 0.000 0.000 999.000 999.000

DFIM3 0.000 0.000 999.000 999.000

SD 0.968 0.083 11.697 0.000

SC2 0.952 0.077 12.376 0.000

IDFIM 0.000 0.000 999.000 999.000

SDFIM -0.447 0.075 -5.934 0.000

ID 0.000 0.000 999.000 999.000

IC 0.555 0.219 2.531 0.011

SC 0.382 0.089 4.297 0.000

Thresholds

ALCD12$1 3.490 0.335 10.406 0.000

ALCD13$1 3.490 0.335 10.406 0.000

ALCD14$1 3.490 0.335 10.406 0.000

ALCD15$1 3.490 0.335 10.406 0.000

ALCD16$1 3.490 0.335 10.406 0.000

ALCD17$1 3.490 0.335 10.406 0.000

ALCD18$1 3.490 0.335 10.406 0.000

ALCD19$1 3.490 0.335 10.406 0.000

ALCD20$1 3.490 0.335 10.406 0.000

Variances

IC12 0.263 0.049 5.378 0.000

IC13 0.230 0.050 4.572 0.000

IC14 0.206 0.047 4.418 0.000

Residual Variances

ALCC12 0.000 0.000 999.000 999.000

ALCC13 0.425 0.111 3.815 0.000

ALCC14 0.729 0.179 4.073 0.000

ALCC15 0.894 0.154 5.803 0.000

ALCC16 0.815 0.134 6.068 0.000

ALCC17 1.482 0.170 8.739 0.000

ALCC18 1.467 0.193 7.580 0.000

ALCC19 0.945 0.236 4.004 0.000

ALCC20 0.807 0.168 4.800 0.000

IC12R1 0.715 0.067 10.717 0.000

IC12R2 0.450 0.056 8.058 0.000

IC12R3 0.607 0.079 7.662 0.000

IC13R1 0.312 0.035 8.960 0.000

IC13R2 0.450 0.056 8.058 0.000

IC13R3 0.317 0.039 8.046 0.000

IC14R1 0.312 0.035 8.960 0.000

IC14R2 0.171 0.040 4.290 0.000

IC14R3 0.317 0.039 8.046 0.000

SR12R1 0.309 0.046 6.778 0.000

SR12R2 0.193 0.021 9.384 0.000

SR12R3 0.248 0.037 6.775 0.000

SR13R1 0.131 0.017 7.555 0.000

SR13R2 0.193 0.021 9.384 0.000

SR13R3 0.202 0.027 7.488 0.000

SR14R1 0.131 0.017 7.555 0.000

SR14R2 0.193 0.021 9.384 0.000

SR14R3 0.202 0.027 7.488 0.000

SR12 0.000 0.000 999.000 999.000

SR13 0.000 0.000 999.000 999.000

SR14 0.000 0.000 999.000 999.000

DFIM1 0.311 0.063 4.913 0.000

DFIM2 0.311 0.063 4.913 0.000

DFIM3 0.311 0.063 4.913 0.000

SD 0.280 0.063 4.471 0.000

SC2 0.296 0.052 5.704 0.000

IDFIM 0.241 0.043 5.550 0.000

SDFIM 0.183 0.050 3.632 0.000

ID 6.684 1.410 4.740 0.000

IC 0.284 0.096 2.971 0.003

SC 0.048 0.017 2.853 0.004

STANDARDIZED MODEL RESULTS

STDYX Standardization

Two-Tailed

Estimate S.E. Est./S.E. P-Value

ID |

ALCD12 0.767 0.019 41.452 0.000

ALCD13 0.819 0.028 28.751 0.000

ALCD14 0.856 0.046 18.784 0.000

ALCD15 0.868 0.070 12.419 0.000

ALCD16 0.856 0.079 10.801 0.000

ALCD17 0.820 0.087 9.391 0.000

ALCD18 0.768 0.088 8.693 0.000

ALCD19 0.709 0.085 8.391 0.000

ALCD20 0.696 0.091 7.655 0.000

SD |

ALCD12 -0.157 0.012 -13.395 0.000

ALCD13 0.000 0.000 999.000 999.000

ALCD14 0.175 0.015 11.516 0.000

ALCD15 0.417 0.049 8.502 0.000

ALCD16 0.525 0.053 9.981 0.000

ALCD17 0.670 0.069 9.712 0.000

ALCD18 0.784 0.080 9.800 0.000

ALCD19 0.869 0.085 10.182 0.000

ALCD20 0.884 0.084 10.551 0.000

IC |

ALCC12 1.307 0.110 11.891 0.000

ALCC13 0.633 0.075 8.417 0.000

ALCC14 0.481 0.078 6.207 0.000

ALCC15 0.409 0.067 6.058 0.000

ALCC16 0.375 0.063 5.938 0.000

ALCC17 0.320 0.055 5.772 0.000

ALCC18 0.287 0.048 6.021 0.000

ALCC19 0.294 0.048 6.162 0.000

ALCC20 0.293 0.048 6.093 0.000

SC |

ALCC12 -0.537 0.202 -2.655 0.008

ALCC13 0.000 0.000 999.000 999.000

ALCC14 0.198 0.038 5.257 0.000

ALCC15 0.336 0.058 5.780 0.000

ALCC16 0.462 0.075 6.118 0.000

ALCC17 0.395 0.065 6.073 0.000

ALCC18 0.353 0.062 5.668 0.000

ALCC19 0.362 0.066 5.531 0.000

ALCC20 0.361 0.066 5.489 0.000

SC2 |

ALCC12 0.000 0.000 999.000 999.000

ALCC13 0.000 0.000 999.000 999.000

ALCC14 0.000 0.000 999.000 999.000

ALCC15 0.000 0.000 999.000 999.000

ALCC16 0.000 0.000 999.000 999.000

ALCC17 0.335 0.031 10.954 0.000

ALCC18 0.600 0.048 12.620 0.000

ALCC19 0.712 0.063 11.375 0.000

ALCC20 0.749 0.052 14.343 0.000

IDFIM |

DFIM1 0.670 0.053 12.623 0.000

DFIM2 0.802 0.100 8.001 0.000

DFIM3 0.803 0.101 7.909 0.000

SDFIM |

DFIM1 0.000 0.000 999.000 999.000

DFIM2 0.617 0.115 5.386 0.000

DFIM3 0.683 0.119 5.735 0.000

SR12 BY

SR12R1 0.732 0.029 25.140 0.000

SR12R2 0.809 0.028 28.663 0.000

SR12R3 0.751 0.034 22.288 0.000

SR13 BY

SR13R1 0.734 0.035 20.832 0.000

SR13R2 0.670 0.033 20.442 0.000

SR13R3 0.624 0.044 14.024 0.000

SR14 BY

SR14R1 0.703 0.038 18.560 0.000

SR14R2 0.637 0.036 17.485 0.000

SR14R3 0.589 0.044 13.380 0.000

IC12 BY

IC12R1 0.518 0.041 12.608 0.000

IC12R2 0.713 0.037 19.465 0.000

IC12R3 0.669 0.033 20.035 0.000

IC13 BY

IC13R1 0.652 0.055 11.864 0.000

IC13R2 0.690 0.036 19.326 0.000

IC13R3 0.759 0.031 24.769 0.000

IC14 BY

IC14R1 0.630 0.057 10.999 0.000

IC14R2 0.825 0.038 21.816 0.000

IC14R3 0.741 0.031 24.043 0.000

DFIM1 BY

SR12 1.260 0.085 14.883 0.000

DFIM2 BY

SR13 1.606 0.113 14.212 0.000

DFIM3 BY

SR14 1.755 0.150 11.712 0.000

SR12 ON

IC12 0.859 0.100 8.553 0.000

SR13 ON

IC13 1.227 0.132 9.327 0.000

SR14 ON

IC14 1.268 0.157 8.067 0.000

ID ON

GENDER -0.069 0.072 -0.959 0.338

SD ON

GENDER 0.034 0.084 0.410 0.682

IC ON

GENDER -0.034 0.122 -0.280 0.779

SC ON

GENDER 0.060 0.147 0.408 0.683

SC2 ON

GENDER 0.224 0.088 2.544 0.011

IDFIM ON

GENDER 0.224 0.065 3.423 0.001

SDFIM ON

GENDER -0.054 0.084 -0.642 0.521

IC12 WITH

DFIM1 -0.825 0.051 -16.107 0.000

IC13 0.103 0.044 2.327 0.020

IC13 WITH

DFIM2 -0.881 0.029 -30.915 0.000

IC14 WITH

DFIM3 -0.933 0.032 -29.334 0.000

IDFIM WITH

SDFIM -0.831 0.044 -18.848 0.000

ID 0.105 0.091 1.149 0.251

SD -0.044 0.126 -0.353 0.724

IC 0.031 0.188 0.163 0.871

SC 0.258 0.219 1.178 0.239

SC2 -0.061 0.122 -0.495 0.621

ID WITH

SD -0.408 0.130 -3.152 0.002

IC 0.509 0.267 1.910 0.056

SC 0.572 0.167 3.424 0.001

SC2 0.000 0.000 999.000 999.000

SDFIM -0.080 0.113 -0.713 0.476

IC WITH

SC 0.714 0.088 8.074 0.000

SC2 -0.410 0.203 -2.020 0.043

SD 0.000 0.000 999.000 999.000

SDFIM -0.023 0.226 -0.102 0.919

SC WITH

SC2 0.000 0.000 999.000 999.000

SD 0.000 0.000 999.000 999.000

SDFIM -0.277 0.241 -1.149 0.250

SD WITH

SC2 0.460 0.133 3.466 0.001

SDFIM -0.009 0.148 -0.061 0.951

SC2 WITH

SDFIM 0.166 0.148 1.117 0.264

SR13R3 WITH

SR13R1 -0.278 0.123 -2.263 0.024

SR13R2 0.327 0.095 3.430 0.001

IC14R3 WITH

IC12R1 0.288 0.079 3.660 0.000

SR14R3 WITH

SR14R2 0.393 0.075 5.249 0.000

IC14R1 WITH

IC12R1 0.177 0.068 2.582 0.010

IC13R3 WITH

IC13R1 -0.315 0.148 -2.133 0.033

IC12R2 0.291 0.114 2.558 0.011

IC12R3 0.224 0.093 2.423 0.015

SR14R2 WITH

SR13R1 -0.180 0.084 -2.151 0.031

Means

IC12 -1.457 0.359 -4.061 0.000

IC13 -0.901 0.361 -2.497 0.013

IC14 -0.761 0.385 -1.976 0.048

Intercepts

ALCC12 0.000 0.000 999.000 999.000

ALCC13 0.000 0.000 999.000 999.000

ALCC14 0.000 0.000 999.000 999.000

ALCC15 0.000 0.000 999.000 999.000

ALCC16 0.000 0.000 999.000 999.000

ALCC17 0.000 0.000 999.000 999.000

ALCC18 0.000 0.000 999.000 999.000

ALCC19 0.000 0.000 999.000 999.000

ALCC20 0.000 0.000 999.000 999.000

IC12R1 0.760 0.211 3.600 0.000

IC12R2 0.785 0.230 3.419 0.001

IC12R3 0.716 0.210 3.411 0.001

IC13R1 1.020 0.279 3.656 0.000

IC13R2 0.811 0.231 3.511 0.000

IC13R3 0.868 0.257 3.383 0.001

IC14R1 1.044 0.287 3.637 0.000

IC14R2 1.026 0.294 3.486 0.000

IC14R3 0.896 0.263 3.406 0.001

SR12R1 1.549 0.316 4.895 0.000

SR12R2 1.688 0.341 4.949 0.000

SR12R3 1.675 0.310 5.397 0.000

SR13R1 2.368 0.445 5.325 0.000

SR13R2 2.133 0.385 5.536 0.000

SR13R3 2.198 0.410 5.367 0.000

SR14R1 2.480 0.478 5.184 0.000

SR14R2 2.216 0.407 5.444 0.000

SR14R3 2.272 0.435 5.224 0.000

SR12 0.000 0.000 999.000 999.000

SR13 0.000 0.000 999.000 999.000

SR14 0.000 0.000 999.000 999.000

DFIM1 0.000 0.000 999.000 999.000

DFIM2 0.000 0.000 999.000 999.000

DFIM3 0.000 0.000 999.000 999.000

SD 1.828 0.185 9.889 0.000

SC2 1.705 0.221 7.730 0.000

IDFIM 0.000 0.000 999.000 999.000

SDFIM -1.042 0.226 -4.613 0.000

ID 0.000 0.000 999.000 999.000

IC 1.039 0.555 1.874 0.061

SC 1.742 0.653 2.667 0.008

Thresholds

ALCD12$1 1.033 0.083 12.436 0.000

ALCD13$1 1.103 0.084 13.188 0.000

ALCD14$1 1.153 0.087 13.198 0.000

ALCD15$1 1.169 0.099 11.821 0.000

ALCD16$1 1.153 0.105 10.958 0.000

ALCD17$1 1.104 0.111 9.947 0.000

ALCD18$1 1.034 0.111 9.316 0.000

ALCD19$1 0.955 0.106 8.972 0.000

ALCD20$1 0.937 0.115 8.153 0.000

Variances

IC12 1.000 0.000 999.000 999.000

IC13 1.000 0.000 999.000 999.000

IC14 1.000 0.000 999.000 999.000

Residual Variances

ALCC12 0.000 999.000 999.000 999.000

ALCC13 0.599 0.095 6.285 0.000

ALCC14 0.594 0.078 7.643 0.000

ALCC15 0.525 0.058 9.050 0.000

ALCC16 0.401 0.059 6.834 0.000

ALCC17 0.534 0.043 12.507 0.000

ALCC18 0.423 0.049 8.666 0.000

ALCC19 0.287 0.066 4.349 0.000

ALCC20 0.244 0.050 4.898 0.000

IC12R1 0.731 0.043 17.160 0.000

IC12R2 0.491 0.052 9.393 0.000

IC12R3 0.552 0.045 12.341 0.000

IC13R1 0.575 0.072 8.036 0.000

IC13R2 0.524 0.049 10.638 0.000

IC13R3 0.423 0.047 9.090 0.000

IC14R1 0.603 0.072 8.340 0.000

IC14R2 0.319 0.062 5.099 0.000

IC14R3 0.451 0.046 9.877 0.000

SR12R1 0.464 0.043 10.898 0.000

SR12R2 0.345 0.046 7.560 0.000

SR12R3 0.437 0.051 8.640 0.000

SR13R1 0.462 0.052 8.937 0.000

SR13R2 0.551 0.044 12.542 0.000

SR13R3 0.611 0.055 11.016 0.000

SR14R1 0.506 0.053 9.521 0.000

SR14R2 0.595 0.046 12.828 0.000

SR14R3 0.653 0.052 12.567 0.000

SR12 0.000 999.000 999.000 999.000

SR13 0.000 999.000 999.000 999.000

SR14 0.000 999.000 999.000 999.000

DFIM1 0.551 0.071 7.736 0.000

DFIM2 0.789 0.045 17.503 0.000

DFIM3 0.790 0.046 17.109 0.000

SD 0.999 0.006 172.999 0.000

SC2 0.950 0.039 24.165 0.000

IDFIM 0.950 0.029 32.434 0.000

SDFIM 0.997 0.009 109.016 0.000

ID 0.995 0.010 100.138 0.000

IC 0.999 0.008 118.938 0.000

SC 0.996 0.018 56.480 0.000

R-SQUARE

Observed Two-Tailed

Variable Estimate S.E. Est./S.E. P-Value

ALCD12 0.712 0.049 14.397 0.000

ALCD13 0.671 0.047 14.376 0.000

ALCD14 0.641 0.040 15.994 0.000

ALCD15 0.631 0.033 19.276 0.000

ALCD16 0.641 0.033 19.703 0.000

ALCD17 0.671 0.034 19.593 0.000

ALCD18 0.711 0.035 20.531 0.000

ALCD19 0.754 0.033 22.917 0.000

ALCD20 0.763 0.037 20.398 0.000

ALCC12 1.000 999.000 999.000 999.000

ALCC13 0.401 0.095 4.208 0.000

ALCC14 0.406 0.078 5.228 0.000

ALCC15 0.475 0.058 8.194 0.000

ALCC16 0.599 0.059 10.199 0.000

ALCC17 0.466 0.043 10.926 0.000

ALCC18 0.577 0.049 11.804 0.000

ALCC19 0.713 0.066 10.811 0.000

ALCC20 0.756 0.050 15.186 0.000

IC12R1 0.269 0.043 6.304 0.000

IC12R2 0.509 0.052 9.733 0.000

IC12R3 0.448 0.045 10.017 0.000

IC13R1 0.425 0.072 5.932 0.000

IC13R2 0.476 0.049 9.663 0.000

IC13R3 0.577 0.047 12.385 0.000

IC14R1 0.397 0.072 5.500 0.000

IC14R2 0.681 0.062 10.908 0.000

IC14R3 0.549 0.046 12.022 0.000

SR12R1 0.536 0.043 12.570 0.000

SR12R2 0.655 0.046 14.332 0.000

SR12R3 0.563 0.051 11.144 0.000

SR13R1 0.538 0.052 10.416 0.000

SR13R2 0.449 0.044 10.221 0.000

SR13R3 0.389 0.055 7.012 0.000

SR14R1 0.494 0.053 9.280 0.000

SR14R2 0.405 0.046 8.743 0.000

SR14R3 0.347 0.052 6.690 0.000

Latent Two-Tailed

Variable Estimate S.E. Est./S.E. P-Value

SR12 1.000 999.000 999.000 999.000

SR13 1.000 999.000 999.000 999.000

SR14 1.000 999.000 999.000 999.000

DFIM1 0.449 0.071 6.312 0.000

DFIM2 0.211 0.045 4.692 0.000

DFIM3 0.210 0.046 4.560 0.000

SD 0.001 0.006 0.205 0.838

SC2 0.050 0.039 1.272 0.203

IDFIM 0.050 0.029 1.712 0.087

SDFIM 0.003 0.009 0.321 0.748

ID 0.005 0.010 0.479 0.632

IC 0.001 0.008 0.140 0.889

SC 0.004 0.018 0.204 0.838

QUALITY OF NUMERICAL RESULTS

Condition Number for the Information Matrix 0.988E-06

(ratio of smallest to largest eigenvalue)

MODEL COMMAND WITH FINAL ESTIMATES USED AS STARTING VALUES

idfim sdfim | dfim1@0 dfim2* dfim3@1;

id sd | alcd12@-1 alcd13@0 alcd14@1 alcd15* alcd16@3 alcd17@4

alcd18@5 alcd19@6 alcd20*;

ic sc | alcc12@-1 alcc13@0 alcc14@1 alcc15@2 alcc16@3 alcc17@3

alcc18@3 alcc19@3 alcc20@3;

ic sc2 | alcc12@0 alcc13@0 alcc14@0 alcc15@0 alcc16@0 alcc17@1

alcc18@2 alcc19* alcc20*;

sr12 BY sr12r1@1;

sr12 BY sr12r2*1.01438 (sr2);

sr12 BY sr12r3*0.94832;

sr13 BY sr13r1@1;

sr13 BY sr13r2*1.01438 (sr2);

sr13 BY sr13r3*0.91585 (sr3);

sr14 BY sr14r1@1;

sr14 BY sr14r2*1.01438 (sr2);

sr14 BY sr14r3*0.91585 (sr3);

ic12 BY ic12r1@1;

ic12 BY ic12r2*1.33170 (ic2);

ic12 BY ic12r3*1.36905 (ic3);

ic13 BY ic13r1@1;

ic13 BY ic13r2*1.33170 (ic2);

ic13 BY ic13r3*1.36905 (ic3);

ic14 BY ic14r1@1;

ic14 BY ic14r2*1.33170 (ic2);

ic14 BY ic14r3*1.36905 (ic3);

sd BY alcd15*2.34924;

sd BY alcd20*6.21855;

sc2 BY alcc19*2.31494;

sc2 BY alcc20*2.43884;

dfim1 BY sr12@1;

dfim2 BY sr13@1;

dfim3 BY sr14@1;

sdfim BY dfim2*0.90397;

id ON gender*-0.35985;

sd ON gender*0.03664;

ic ON gender*-0.03683;

sc ON gender*0.02647;

sc2 ON gender*0.25123;

idfim ON gender*0.22691;

sdfim ON gender*-0.04671;

sr12 ON ic12@1;

sr13 ON ic13@1;

sr14 ON ic14@1;

sr13r3 WITH sr13r1*-0.04523;

sr13r3 WITH sr13r2*0.06443;

ic14r3 WITH ic12r1*0.13709;

sr14r3 WITH sr14r2*0.07746;

ic14r1 WITH ic12r1*0.08345;

ic13r3 WITH ic13r1*-0.09922;

ic13r3 WITH ic12r2*0.10975;

ic13r3 WITH ic12r3*0.09839;

sr14r2 WITH sr13r1*-0.02866;

ic12 WITH dfim1*-0.23597 (icwdfim);

ic12 WITH ic13*0.02529;

ic13 WITH dfim2*-0.23597 (icwdfim);

ic14 WITH dfim3*-0.23597 (icwdfim);

idfim WITH sdfim*-0.17472;

idfim WITH id*0.13327;

idfim WITH sd*-0.01152;

idfim WITH ic*0.00802;

idfim WITH sc*0.02772;

idfim WITH sc2*-0.01618;

id WITH sd*-0.55835;

id WITH ic*0.70245;

id WITH sc*0.32369;

id WITH sc2@0;

id WITH sdfim*-0.08902;

ic WITH sc*0.08332;

ic WITH sc2*-0.11906;

ic WITH sd@0;

ic WITH sdfim*-0.00524;

sc WITH sc2@0;

sc WITH sd@0;

sc WITH sdfim*-0.02592;

sd WITH sc2*0.13251;

sd WITH sdfim*-0.00206;

sc2 WITH sdfim*0.03859;

[ alcc12@0 ];

[ alcc13@0 ];

[ alcc14@0 ];

[ alcc15@0 ];

[ alcc16@0 ];

[ alcc17@0 ];

[ alcc18@0 ];

[ alcc19@0 ];

[ alcc20@0 ];

[ ic12r1*0.75122 ] (ici1);

[ ic12r2*0.75122 ] (ici1);

[ ic12r3*0.75122 ] (ici1);

[ ic13r1*0.75122 ] (ici1);

[ ic13r2*0.75122 ] (ici1);

[ ic13r3*0.75122 ] (ici1);

[ ic14r1*0.75122 ] (ici1);

[ ic14r2*0.75122 ] (ici1);

[ ic14r3*0.75122 ] (ici1);

[ sr12r1*1.26271 ] (sri1);

[ sr12r2*1.26271 ] (sri1);

[ sr12r3*1.26271 ] (sri1);

[ sr13r1*1.26271 ] (sri1);

[ sr13r2*1.26271 ] (sri1);

[ sr13r3*1.26271 ] (sri1);

[ sr14r1*1.26271 ] (sri1);

[ sr14r2*1.26271 ] (sri1);

[ sr14r3*1.26271 ] (sri1);

[ sr12@0 ];

[ sr13@0 ];

[ sr14@0 ];

[ ic12*-0.74696 ];

[ ic13*-0.43258 ];

[ ic14*-0.34503 ];

[ dfim1@0 ];

[ dfim2@0 ];

[ dfim3@0 ];

[ sd*0.96779 ];

[ sc2*0.95223 ];

[ idfim@0 ];

[ sdfim*-0.44681 ];

[ id@0 ];

[ ic*0.55455 ];

[ sc*0.38179 ];

[ alcd12$1*3.48957 ] (86);

[ alcd13$1*3.48957 ] (86);

[ alcd14$1*3.48957 ] (86);

[ alcd15$1*3.48957 ] (86);

[ alcd16$1*3.48957 ] (86);

[ alcd17$1*3.48957 ] (86);

[ alcd18$1*3.48957 ] (86);

[ alcd19$1*3.48957 ] (86);

[ alcd20$1*3.48957 ] (86);

alcc12@0;

alcc13*0.42522;

alcc14*0.72938;

alcc15*0.89381;

alcc16*0.81456;

alcc17*1.48205;

alcc18*1.46657;

alcc19*0.94459;

alcc20*0.80734;

ic12r1*0.71545;

ic12r2*0.44983 (icr2);

ic12r3*0.60686;

ic13r1*0.31211 (icr1);

ic13r2*0.44983 (icr2);

ic13r3*0.31694 (icr3);

ic14r1*0.31211 (icr1);

ic14r2*0.17062;

ic14r3*0.31694 (icr3);

sr12r1*0.30852;

sr12r2*0.19316 (srr2);

sr12r3*0.24812;

sr13r1*0.13126 (srr1);

sr13r2*0.19316 (srr2);

sr13r3*0.20159 (srr3);

sr14r1*0.13126 (srr1);

sr14r2*0.19316 (srr2);

sr14r3*0.20159 (srr3);

sr12@0;

sr13@0;

sr14@0;

ic12*0.26282;

ic13*0.23040;

ic14*0.20582;

dfim1*0.31113 (dfimres);

dfim2*0.31113 (dfimres);

dfim3*0.31113 (dfimres);

sd*0.27996;

sc2*0.29632;

idfim*0.24114;

sdfim*0.18320;

id*6.68421;

ic*0.28440;

sc*0.04788;

RESIDUAL OUTPUT

ESTIMATED MODEL AND RESIDUALS (OBSERVED - ESTIMATED)

Model Estimated Means

ALCC12 ALCC13 ALCC14 ALCC15 ALCC16

________ ________ ________ ________ ________

0.144 0.538 0.932 1.325 1.719

Model Estimated Means

ALCC17 ALCC18 ALCC19 ALCC20 IC12R1

________ ________ ________ ________ ________

2.783 3.848 4.183 4.315 0.004

Model Estimated Means

IC12R2 IC12R3 IC13R1 IC13R2 IC13R3

________ ________ ________ ________ ________

-0.244 -0.271 0.319 0.175 0.159

Model Estimated Means

IC14R1 IC14R2 IC14R3 SR12R1 SR12R2

________ ________ ________ ________ ________

0.406 0.292 0.279 0.617 0.608

Model Estimated Means

SR12R3 SR13R1 SR13R2 SR13R3 SR14R1

________ ________ ________ ________ ________

0.650 0.509 0.498 0.572 0.551

Model Estimated Means

SR14R2 SR14R3

________ ________

0.541 0.611

Residuals for Means

ALCC12 ALCC13 ALCC14 ALCC15 ALCC16

________ ________ ________ ________ ________

0.413 0.274 0.451 0.656 0.523

Residuals for Means

ALCC17 ALCC18 ALCC19 ALCC20 IC12R1

________ ________ ________ ________ ________

0.455 0.583 0.357 0.369 -0.035

Residuals for Means

IC12R2 IC12R3 IC13R1 IC13R2 IC13R3

________ ________ ________ ________ ________

-0.045 0.055 0.006 0.008 -0.042

Residuals for Means

IC14R1 IC14R2 IC14R3 SR12R1 SR12R2

________ ________ ________ ________ ________

-0.037 0.016 -0.064 0.053 -0.034

Residuals for Means

SR12R3 SR13R1 SR13R2 SR13R3 SR14R1

________ ________ ________ ________ ________

-0.027 -0.027 0.008 0.025 -0.025

Residuals for Means

SR14R2 SR14R3

________ ________

0.033 0.012

Model Estimated Covariances

ALCC12 ALCC13 ALCC14 ALCC15 ALCC16

________ ________ ________ ________ ________

ALCC12 0.167

ALCC13 0.202 0.710

ALCC14 0.237 0.368 1.228

ALCC15 0.272 0.451 0.630 1.703

ALCC16 0.307 0.534 0.761 0.988 2.030

ALCC17 0.184 0.413 0.641 0.870 1.099

ALCC18 0.061 0.291 0.522 0.752 0.983

ALCC19 0.022 0.253 0.484 0.715 0.946

ALCC20 0.007 0.238 0.469 0.700 0.932

IC12R1 0.000 0.000 0.000 0.000 0.000

IC12R2 0.000 0.000 0.000 0.000 0.000

IC12R3 0.000 0.000 0.000 0.000 0.000

IC13R1 0.000 0.000 0.000 0.000 0.000

IC13R2 0.000 0.000 0.000 0.000 0.000

IC13R3 0.000 0.000 0.000 0.000 0.000

IC14R1 0.000 0.000 0.000 0.000 0.000

IC14R2 0.000 0.000 0.000 0.000 0.000

IC14R3 0.000 0.000 0.000 0.000 0.000

SR12R1 -0.023 0.006 0.035 0.064 0.094

SR12R2 -0.024 0.006 0.036 0.065 0.095

SR12R3 -0.022 0.006 0.033 0.061 0.089

SR13R1 -0.004 0.002 0.007 0.013 0.018

SR13R2 -0.004 0.002 0.007 0.013 0.018

SR13R3 -0.004 0.001 0.007 0.012 0.017

SR14R1 -0.002 0.001 0.004 0.007 0.010

SR14R2 -0.002 0.001 0.004 0.007 0.010

SR14R3 -0.002 0.001 0.004 0.006 0.009

Model Estimated Covariances

ALCC17 ALCC18 ALCC19 ALCC20 IC12R1

________ ________ ________ ________ ________

ALCC17 2.777

ALCC18 1.490 3.464

ALCC19 1.552 2.157 3.293

ALCC20 1.576 2.220 2.423 3.310

IC12R1 0.000 0.000 0.000 0.000 0.978

IC12R2 0.000 0.000 0.000 0.000 0.350

IC12R3 0.000 0.000 0.000 0.000 0.360

IC13R1 0.000 0.000 0.000 0.000 0.025

IC13R2 0.000 0.000 0.000 0.000 0.034

IC13R3 0.000 0.000 0.000 0.000 0.035

IC14R1 0.000 0.000 0.000 0.000 0.083

IC14R2 0.000 0.000 0.000 0.000 0.000

IC14R3 0.000 0.000 0.000 0.000 0.137

SR12R1 0.091 0.089 0.089 0.088 0.027

SR12R2 0.093 0.091 0.090 0.090 0.027

SR12R3 0.087 0.085 0.084 0.084 0.025

SR13R1 0.048 0.078 0.088 0.092 0.025

SR13R2 0.049 0.080 0.089 0.093 0.026

SR13R3 0.044 0.072 0.081 0.084 0.023

SR14R1 0.044 0.077 0.088 0.092 0.000

SR14R2 0.044 0.078 0.089 0.093 0.000

SR14R3 0.040 0.071 0.080 0.084 0.000

Model Estimated Covariances

IC12R2 IC12R3 IC13R1 IC13R2 IC13R3

________ ________ ________ ________ ________

IC12R2 0.916

IC12R3 0.479 1.099

IC13R1 0.034 0.035 0.543

IC13R2 0.045 0.046 0.307 0.858

IC13R3 0.156 0.146 0.216 0.420 0.749

IC14R1 0.000 0.000 0.000 0.000 0.000

IC14R2 0.000 0.000 0.000 0.000 0.000

IC14R3 0.000 0.000 0.000 0.000 0.000

SR12R1 0.036 0.037 0.025 0.034 0.035

SR12R2 0.036 0.037 0.026 0.034 0.035

SR12R3 0.034 0.035 0.024 0.032 0.033

SR13R1 0.034 0.035 -0.006 -0.007 -0.008

SR13R2 0.034 0.035 -0.006 -0.008 -0.008

SR13R3 0.031 0.032 -0.005 -0.007 -0.007

SR14R1 0.000 0.000 0.000 0.000 0.000

SR14R2 0.000 0.000 0.000 0.000 0.000

SR14R3 0.000 0.000 0.000 0.000 0.000

Model Estimated Covariances

IC14R1 IC14R2 IC14R3 SR12R1 SR12R2

________ ________ ________ ________ ________

IC14R1 0.518

IC14R2 0.274 0.536

IC14R3 0.282 0.375 0.703

SR12R1 0.000 0.000 0.000 0.664

SR12R2 0.000 0.000 0.000 0.361 0.559

SR12R3 0.000 0.000 0.000 0.337 0.342

SR13R1 0.000 0.000 0.000 0.119 0.121

SR13R2 0.000 0.000 0.000 0.121 0.122

SR13R3 0.000 0.000 0.000 0.109 0.110

SR14R1 -0.030 -0.040 -0.041 0.077 0.078

SR14R2 -0.031 -0.041 -0.042 0.078 0.079

SR14R3 -0.028 -0.037 -0.038 0.070 0.071

Model Estimated Covariances

SR12R3 SR13R1 SR13R2 SR13R3 SR14R1

________ ________ ________ ________ ________

SR12R3 0.568

SR13R1 0.113 0.284

SR13R2 0.114 0.155 0.351

SR13R3 0.103 0.095 0.207 0.330

SR14R1 0.073 0.082 0.084 0.075 0.259

SR14R2 0.074 0.055 0.085 0.076 0.130

SR14R3 0.066 0.075 0.076 0.069 0.117

Model Estimated Covariances

SR14R2 SR14R3

________ ________

SR14R2 0.325

SR14R3 0.196 0.309

Residuals for Covariances

ALCC12 ALCC13 ALCC14 ALCC15 ALCC16

________ ________ ________ ________ ________

ALCC12 0.039

ALCC13 0.312 -0.085

ALCC14 0.816 0.938 -0.004

ALCC15 0.900 0.197 0.971 -0.117

ALCC16 0.476 0.374 1.230 1.022 0.005

ALCC17 0.420 1.598 0.703 0.272 1.503

ALCC18 0.278 0.528 0.273 0.713 1.027

ALCC19 -0.368 0.104 -0.858 0.277 0.808

ALCC20 -1.121 -0.696 -0.645 -0.016 0.169

IC12R1 0.194 -0.214 0.238 0.164 -0.011

IC12R2 0.249 0.102 0.540 0.144 0.277

IC12R3 -0.017 -0.042 0.042 0.100 0.257

IC13R1 0.200 -0.010 0.139 0.060 0.195

IC13R2 -0.155 0.050 0.060 -0.092 -0.182

IC13R3 -0.074 0.092 -0.022 -0.058 0.030

IC14R1 -0.340 -0.079 -0.067 -0.068 0.078

IC14R2 -0.381 -0.098 0.005 0.075 0.235

IC14R3 -0.194 0.050 0.062 -0.057 -0.369

SR12R1 0.049 -0.035 -0.070 0.056 0.215

SR12R2 -0.045 -0.193 -0.105 -0.028 0.143

SR12R3 0.066 -0.086 0.028 0.021 0.004

SR13R1 0.071 -0.081 -0.093 -0.039 0.030

SR13R2 0.007 -0.091 -0.162 -0.058 -0.212

SR13R3 0.146 0.005 -0.102 -0.111 -0.199

SR14R1 0.014 0.090 -0.231 0.006 0.072

SR14R2 -0.095 0.114 -0.194 -0.031 0.191

SR14R3 -0.046 0.020 -0.200 0.051 0.196

Residuals for Covariances

ALCC17 ALCC18 ALCC19 ALCC20 IC12R1

________ ________ ________ ________ ________

ALCC17 -0.166

ALCC18 1.181 -0.663

ALCC19 0.526 0.814 -0.607

ALCC20 -1.662 -0.121 0.992 -0.831

IC12R1 0.136 0.054 -0.077 -0.214 0.097

IC12R2 0.309 0.317 -0.098 -0.569 0.161

IC12R3 0.488 0.713 0.244 -0.170 0.103

IC13R1 -0.095 0.008 -0.095 -0.132 0.098

IC13R2 0.039 -0.064 0.027 -0.020 0.095

IC13R3 0.201 0.085 0.039 0.072 0.141

IC14R1 -0.149 0.310 0.229 0.251 0.077

IC14R2 0.080 0.224 0.393 0.311 0.081

IC14R3 -0.157 -0.081 -0.262 -0.122 0.092

SR12R1 0.360 -0.033 -0.285 -0.424 -0.077

SR12R2 0.067 0.016 -0.315 -0.257 -0.085

SR12R3 -0.068 0.102 -0.212 -0.332 -0.082

SR13R1 0.066 -0.037 -0.134 -0.419 -0.027

SR13R2 -0.094 -0.340 -0.231 -0.301 -0.032

SR13R3 0.050 -0.123 -0.276 -0.145 -0.054

SR14R1 -0.254 -0.033 0.064 0.026 -0.100

SR14R2 -0.205 0.136 -0.034 0.198 -0.062

SR14R3 -0.263 0.040 0.031 0.097 -0.064

Residuals for Covariances

IC12R2 IC12R3 IC13R1 IC13R2 IC13R3

________ ________ ________ ________ ________

IC12R2 0.201

IC12R3 0.133 0.117

IC13R1 0.143 0.187 -0.054

IC13R2 0.120 0.195 -0.103 -0.122

IC13R3 0.141 0.128 -0.032 -0.062 0.018

IC14R1 0.152 0.179 0.091 0.187 0.119

IC14R2 0.157 0.101 0.159 0.193 0.198

IC14R3 0.117 0.146 0.092 0.214 0.186

SR12R1 -0.125 -0.110 -0.068 -0.071 -0.094

SR12R2 -0.060 -0.111 -0.041 -0.079 -0.111

SR12R3 -0.046 -0.073 -0.040 -0.065 -0.109

SR13R1 -0.011 -0.009 0.004 0.007 0.006

SR13R2 -0.080 -0.062 -0.020 0.010 -0.025

SR13R3 -0.093 -0.105 -0.068 -0.015 -0.059

SR14R1 -0.082 -0.115 -0.046 -0.023 -0.038

SR14R2 -0.093 -0.117 -0.009 -0.038 0.018

SR14R3 -0.095 -0.109 -0.014 -0.012 -0.020

Residuals for Covariances

IC14R1 IC14R2 IC14R3 SR12R1 SR12R2

________ ________ ________ ________ ________

IC14R1 0.009

IC14R2 -0.057 -0.083

IC14R3 -0.062 -0.124 -0.116

SR12R1 -0.124 -0.065 -0.074 -0.038

SR12R2 -0.152 -0.094 -0.102 -0.008 0.013

SR12R3 -0.103 -0.030 -0.061 -0.040 -0.018

SR13R1 -0.005 -0.026 0.014 -0.014 -0.006

SR13R2 -0.050 -0.072 0.018 -0.034 0.002

SR13R3 -0.031 -0.087 -0.005 -0.020 0.029

SR14R1 -0.013 0.018 -0.006 -0.049 -0.082

SR14R2 0.031 0.002 0.040 -0.057 -0.056

SR14R3 0.023 -0.007 0.014 -0.051 -0.054

Residuals for Covariances

SR12R3 SR13R1 SR13R2 SR13R3 SR14R1

________ ________ ________ ________ ________

SR12R3 -0.036

SR13R1 -0.023 0.008

SR13R2 -0.024 -0.006 -0.015

SR13R3 0.015 0.007 0.006 0.025

SR14R1 -0.036 -0.010 -0.016 0.028 -0.038

SR14R2 -0.062 -0.002 -0.004 0.023 -0.007

SR14R3 -0.068 0.017 0.025 0.069 -0.005

Residuals for Covariances

SR14R2 SR14R3

________ ________

SR14R2 0.000

SR14R3 -0.003 -0.009

UNIVARIATE DISTRIBUTION FIT

Variable Observed Estimated Residual (Obs.-Est.) Stand. Residual

ALCD12

Category 1 0.958 0.916 0.042 2.975

Category 2 0.042 0.084 -0.042 -2.975

ALCD13

Category 1 0.874 0.878 -0.004 -0.220

Category 2 0.126 0.122 0.004 0.220

ALCD14

Category 1 0.782 0.814 -0.032 -1.592

Category 2 0.218 0.186 0.032 1.592

ALCD15

Category 1 0.647 0.676 -0.028 -1.177

Category 2 0.353 0.324 0.028 1.177

ALCD16

Category 1 0.570 0.593 -0.022 -0.894

Category 2 0.430 0.407 0.022 0.894

ALCD17

Category 1 0.482 0.463 0.019 0.732

Category 2 0.518 0.537 -0.019 -0.732

ALCD18

Category 1 0.362 0.352 0.010 0.404

Category 2 0.638 0.648 -0.010 -0.404

ALCD19

Category 1 0.279 0.267 0.012 0.536

Category 2 0.721 0.733 -0.012 -0.536

ALCD20

Category 1 0.243 0.252 -0.009 -0.424

Category 2 0.757 0.748 0.009 0.424

BIVARIATE DISTRIBUTIONS FIT

Variable Variable Observed Estimated Residual (Obs.-Est.) Stand. Residual

ALCD12 ALCD13

Category 1 Category 1 0.874 0.839 0.035 1.853

Category 1 Category 2 0.090 0.076 0.013 0.987

Category 2 Category 1 0.009 0.038 -0.029 -2.979

Category 2 Category 2 0.027 0.046 -0.019 -1.776

ALCD12 ALCD14

Category 1 Category 1 0.779 0.783 -0.004 -0.169

Category 1 Category 2 0.180 0.133 0.047 2.703

Category 2 Category 1 0.006 0.031 -0.025 -2.847

Category 2 Category 2 0.035 0.053 -0.018 -1.585

ALCD12 ALCD15

Category 1 Category 1 0.646 0.652 -0.006 -0.249

Category 1 Category 2 0.314 0.264 0.050 2.227

Category 2 Category 1 0.006 0.023 -0.017 -2.228

Category 2 Category 2 0.034 0.061 -0.027 -2.203

ALCD12 ALCD16

Category 1 Category 1 0.576 0.572 0.004 0.177

Category 1 Category 2 0.389 0.344 0.045 1.837

Category 2 Category 1 0.000 0.021 -0.021 -2.840

Category 2 Category 2 0.035 0.063 -0.028 -2.282

ALCD12 ALCD17

Category 1 Category 1 0.472 0.446 0.027 1.044

Category 1 Category 2 0.483 0.470 0.013 0.499

Category 2 Category 1 0.021 0.018 0.003 0.468

Category 2 Category 2 0.024 0.067 -0.042 -3.329

ALCD12 ALCD18

Category 1 Category 1 0.363 0.336 0.027 1.113

Category 1 Category 2 0.597 0.579 0.018 0.699

Category 2 Category 1 0.004 0.015 -0.012 -1.884

Category 2 Category 2 0.036 0.069 -0.033 -2.526

ALCD12 ALCD19

Category 1 Category 1 0.260 0.253 0.007 0.315

Category 1 Category 2 0.699 0.663 0.037 1.513

Category 2 Category 1 0.010 0.014 -0.004 -0.659

Category 2 Category 2 0.030 0.070 -0.040 -3.036

ALCD12 ALCD20

Category 1 Category 1 0.239 0.238 0.001 0.028

Category 1 Category 2 0.730 0.678 0.053 2.199

Category 2 Category 1 0.004 0.014 -0.009 -1.581

Category 2 Category 2 0.027 0.070 -0.044 -3.344

ALCD13 ALCD14

Category 1 Category 1 0.736 0.764 -0.028 -1.291

Category 1 Category 2 0.138 0.114 0.024 1.476

Category 2 Category 1 0.046 0.050 -0.004 -0.362

Category 2 Category 2 0.080 0.072 0.008 0.613

ALCD13 ALCD15

Category 1 Category 1 0.627 0.642 -0.015 -0.618

Category 1 Category 2 0.244 0.236 0.008 0.368

Category 2 Category 1 0.027 0.034 -0.007 -0.736

Category 2 Category 2 0.102 0.088 0.014 0.962

ALCD13 ALCD16

Category 1 Category 1 0.542 0.564 -0.022 -0.869

Category 1 Category 2 0.335 0.313 0.022 0.933

Category 2 Category 1 0.031 0.028 0.003 0.365

Category 2 Category 2 0.091 0.094 -0.003 -0.214

ALCD13 ALCD17

Category 1 Category 1 0.445 0.441 0.004 0.151

Category 1 Category 2 0.452 0.436 0.016 0.623

Category 2 Category 1 0.031 0.022 0.009 1.167

Category 2 Category 2 0.072 0.100 -0.028 -1.849

ALCD13 ALCD18

Category 1 Category 1 0.337 0.334 0.003 0.129

Category 1 Category 2 0.541 0.544 -0.003 -0.103

Category 2 Category 1 0.022 0.018 0.003 0.498

Category 2 Category 2 0.100 0.104 -0.004 -0.249

ALCD13 ALCD19

Category 1 Category 1 0.256 0.252 0.004 0.188

Category 1 Category 2 0.628 0.626 0.002 0.077

Category 2 Category 1 0.023 0.016 0.008 1.209

Category 2 Category 2 0.093 0.107 -0.014 -0.871

ALCD13 ALCD20

Category 1 Category 1 0.229 0.237 -0.008 -0.349

Category 1 Category 2 0.645 0.641 0.004 0.180

Category 2 Category 1 0.013 0.015 -0.002 -0.352

Category 2 Category 2 0.113 0.107 0.005 0.340

ALCD14 ALCD15

Category 1 Category 1 0.592 0.620 -0.028 -1.131

Category 1 Category 2 0.178 0.194 -0.016 -0.812

Category 2 Category 1 0.056 0.056 0.000 0.035

Category 2 Category 2 0.175 0.131 0.044 2.558

ALCD14 ALCD16

Category 1 Category 1 0.520 0.549 -0.029 -1.136

Category 1 Category 2 0.268 0.265 0.003 0.129

Category 2 Category 1 0.055 0.044 0.012 1.120

Category 2 Category 2 0.157 0.143 0.014 0.800

ALCD14 ALCD17

Category 1 Category 1 0.444 0.433 0.012 0.466

Category 1 Category 2 0.360 0.381 -0.021 -0.837

Category 2 Category 1 0.044 0.031 0.013 1.472

Category 2 Category 2 0.152 0.156 -0.004 -0.217

ALCD14 ALCD18

Category 1 Category 1 0.324 0.329 -0.005 -0.207

Category 1 Category 2 0.472 0.485 -0.013 -0.507

Category 2 Category 1 0.039 0.023 0.016 2.051

Category 2 Category 2 0.165 0.163 0.002 0.116

ALCD14 ALCD19

Category 1 Category 1 0.235 0.249 -0.013 -0.606

Category 1 Category 2 0.548 0.565 -0.016 -0.648

Category 2 Category 1 0.039 0.018 0.020 2.964

Category 2 Category 2 0.177 0.168 0.009 0.496

ALCD14 ALCD20

Category 1 Category 1 0.203 0.235 -0.031 -1.440

Category 1 Category 2 0.547 0.579 -0.032 -1.288

Category 2 Category 1 0.042 0.018 0.025 3.680

Category 2 Category 2 0.208 0.169 0.039 2.035

ALCD15 ALCD16

Category 1 Category 1 0.502 0.502 0.000 0.000

Category 1 Category 2 0.149 0.174 -0.025 -1.278

Category 2 Category 1 0.060 0.091 -0.031 -2.087

Category 2 Category 2 0.289 0.233 0.055 2.564

ALCD15 ALCD17

Category 1 Category 1 0.415 0.407 0.008 0.310

Category 1 Category 2 0.268 0.269 0.000 -0.019

Category 2 Category 1 0.049 0.057 -0.008 -0.662

Category 2 Category 2 0.268 0.268 0.000 0.020

ALCD15 ALCD18

Category 1 Category 1 0.293 0.315 -0.022 -0.915

Category 1 Category 2 0.373 0.360 0.013 0.523

Category 2 Category 1 0.062 0.037 0.025 2.587

Category 2 Category 2 0.272 0.288 -0.016 -0.690

ALCD15 ALCD19

Category 1 Category 1 0.225 0.241 -0.017 -0.760

Category 1 Category 2 0.433 0.434 -0.001 -0.049

Category 2 Category 1 0.047 0.026 0.021 2.614

Category 2 Category 2 0.295 0.299 -0.003 -0.143

ALCD15 ALCD20

Category 1 Category 1 0.197 0.228 -0.032 -1.472

Category 1 Category 2 0.424 0.448 -0.024 -0.942

Category 2 Category 1 0.044 0.024 0.020 2.488

Category 2 Category 2 0.336 0.300 0.036 1.535

ALCD16 ALCD17

Category 1 Category 1 0.404 0.385 0.019 0.756

Category 1 Category 2 0.193 0.208 -0.015 -0.718

Category 2 Category 1 0.070 0.079 -0.009 -0.621

Category 2 Category 2 0.333 0.329 0.005 0.193

ALCD16 ALCD18

Category 1 Category 1 0.277 0.304 -0.026 -1.128

Category 1 Category 2 0.300 0.289 0.011 0.462

Category 2 Category 1 0.075 0.048 0.027 2.432

Category 2 Category 2 0.348 0.359 -0.011 -0.442

ALCD16 ALCD19

Category 1 Category 1 0.209 0.235 -0.026 -1.222

Category 1 Category 2 0.356 0.357 -0.001 -0.042

Category 2 Category 1 0.062 0.032 0.030 3.320

Category 2 Category 2 0.373 0.376 -0.002 -0.092

ALCD16 ALCD20

Category 1 Category 1 0.169 0.223 -0.054 -2.534

Category 1 Category 2 0.369 0.370 -0.001 -0.037

Category 2 Category 1 0.058 0.029 0.028 3.283

Category 2 Category 2 0.404 0.378 0.026 1.068

ALCD17 ALCD18

Category 1 Category 1 0.279 0.277 0.002 0.066

Category 1 Category 2 0.209 0.186 0.023 1.144

Category 2 Category 1 0.070 0.075 -0.005 -0.377

Category 2 Category 2 0.443 0.462 -0.019 -0.754

ALCD17 ALCD19

Category 1 Category 1 0.216 0.222 -0.005 -0.241

Category 1 Category 2 0.265 0.242 0.023 1.052

Category 2 Category 1 0.063 0.046 0.018 1.660

Category 2 Category 2 0.455 0.491 -0.036 -1.395

ALCD17 ALCD20

Category 1 Category 1 0.181 0.211 -0.029 -1.409

Category 1 Category 2 0.280 0.253 0.027 1.219

Category 2 Category 1 0.057 0.041 0.016 1.522

Category 2 Category 2 0.482 0.495 -0.013 -0.517

ALCD18 ALCD19

Category 1 Category 1 0.231 0.201 0.030 1.461

Category 1 Category 2 0.137 0.150 -0.013 -0.724

Category 2 Category 1 0.063 0.066 -0.003 -0.242

Category 2 Category 2 0.569 0.582 -0.014 -0.541

ALCD18 ALCD20

Category 1 Category 1 0.207 0.193 0.013 0.664

Category 1 Category 2 0.141 0.159 -0.017 -0.936

Category 2 Category 1 0.043 0.059 -0.016 -1.294

Category 2 Category 2 0.609 0.589 0.020 0.783

ALCD19 ALCD20

Category 1 Category 1 0.147 0.171 -0.024 -1.260

Category 1 Category 2 0.118 0.096 0.022 1.488

Category 2 Category 1 0.090 0.081 0.009 0.646

Category 2 Category 2 0.645 0.652 -0.007 -0.294

TECHNICAL 4 OUTPUT

ESTIMATES DERIVED FROM THE MODEL

ESTIMATED MEANS FOR THE LATENT VARIABLES

SR12 SR13 SR14 IC12 IC13

________ ________ ________ ________ ________

-0.646 -0.754 -0.711 -0.747 -0.433

ESTIMATED MEANS FOR THE LATENT VARIABLES

IC14 DFIM1 DFIM2 DFIM3 SD

________ ________ ________ ________ ________

-0.345 0.101 -0.321 -0.366 0.984

ESTIMATED MEANS FOR THE LATENT VARIABLES

SC2 IDFIM SDFIM ID IC

________ ________ ________ ________ ________

1.064 0.101 -0.468 -0.161 0.538

ESTIMATED MEANS FOR THE LATENT VARIABLES

SC GENDER

________ ________

0.394 0.446

S.E. FOR ESTIMATED MEANS FOR THE LATENT VARIABLES

SR12 SR13 SR14 IC12 IC13

________ ________ ________ ________ ________

0.234 0.238 0.240 0.236 0.208

S.E. FOR ESTIMATED MEANS FOR THE LATENT VARIABLES

IC14 DFIM1 DFIM2 DFIM3 SD

________ ________ ________ ________ ________

0.204 0.032 0.066 0.075 0.074

S.E. FOR ESTIMATED MEANS FOR THE LATENT VARIABLES

SC2 IDFIM SDFIM ID IC

________ ________ ________ ________ ________

0.072 0.032 0.075 0.170 0.237

S.E. FOR ESTIMATED MEANS FOR THE LATENT VARIABLES

SC GENDER

________ ________

0.093 0.025

EST./S.E. FOR ESTIMATED MEANS FOR THE LATENT VARIABLES

SR12 SR13 SR14 IC12 IC13

________ ________ ________ ________ ________

-2.755 -3.166 -2.965 -3.171 -2.081

EST./S.E. FOR ESTIMATED MEANS FOR THE LATENT VARIABLES

IC14 DFIM1 DFIM2 DFIM3 SD

________ ________ ________ ________ ________

-1.688 3.134 -4.860 -4.865 13.317

EST./S.E. FOR ESTIMATED MEANS FOR THE LATENT VARIABLES

SC2 IDFIM SDFIM ID IC

________ ________ ________ ________ ________

14.729 3.134 -6.199 -0.945 2.270

EST./S.E. FOR ESTIMATED MEANS FOR THE LATENT VARIABLES

SC GENDER

________ ________

4.213 17.576

TWO-TAILED P-VALUE FOR ESTIMATED MEANS FOR THE LATENT VARIABLES

SR12 SR13 SR14 IC12 IC13

________ ________ ________ ________ ________

0.006 0.002 0.003 0.002 0.037

TWO-TAILED P-VALUE FOR ESTIMATED MEANS FOR THE LATENT VARIABLES

IC14 DFIM1 DFIM2 DFIM3 SD

________ ________ ________ ________ ________

0.091 0.002 0.000 0.000 0.000

TWO-TAILED P-VALUE FOR ESTIMATED MEANS FOR THE LATENT VARIABLES

SC2 IDFIM SDFIM ID IC

________ ________ ________ ________ ________

0.000 0.002 0.000 0.345 0.023

TWO-TAILED P-VALUE FOR ESTIMATED MEANS FOR THE LATENT VARIABLES

SC GENDER

________ ________

0.000 0.000

ESTIMATED COVARIANCE MATRIX FOR THE LATENT VARIABLES

SR12 SR13 SR14 IC12 IC13

________ ________ ________ ________ ________

SR12 0.356

SR13 0.119 0.153

SR14 0.077 0.082 0.128

IC12 0.027 0.025 0.000 0.263

IC13 0.025 -0.006 0.000 0.025 0.230

IC14 0.000 0.000 -0.030 0.000 0.000

DFIM1 0.329 0.094 0.077 -0.236 0.000

DFIM2 0.094 0.159 0.082 0.000 -0.236

DFIM3 0.077 0.082 0.158 0.000 0.000

SD -0.009 -0.012 -0.012 0.000 0.000

SC2 -0.002 0.030 0.034 0.000 0.000

IDFIM 0.254 0.094 0.077 0.000 0.000

SDFIM -0.177 -0.011 0.006 0.000 0.000

ID 0.113 0.036 0.028 0.000 0.000

IC 0.006 0.002 0.001 0.000 0.000

SC 0.029 0.005 0.003 0.000 0.000

GENDER 0.056 0.046 0.045 0.000 0.000

ESTIMATED COVARIANCE MATRIX FOR THE LATENT VARIABLES

IC14 DFIM1 DFIM2 DFIM3 SD

________ ________ ________ ________ ________

IC14 0.206

DFIM1 0.000 0.565

DFIM2 0.000 0.094 0.395

DFIM3 -0.236 0.077 0.082 0.394

SD 0.000 -0.009 -0.012 -0.012 0.280

SC2 0.000 -0.002 0.030 0.034 0.135

IDFIM 0.000 0.254 0.094 0.077 -0.009

SDFIM 0.000 -0.177 -0.011 0.006 -0.002

ID 0.000 0.113 0.036 0.028 -0.562

IC 0.000 0.006 0.002 0.001 0.000

SC 0.000 0.029 0.005 0.003 0.000

GENDER 0.000 0.056 0.046 0.045 0.009

ESTIMATED COVARIANCE MATRIX FOR THE LATENT VARIABLES

SC2 IDFIM SDFIM ID IC

________ ________ ________ ________ ________

SC2 0.312

IDFIM -0.002 0.254

SDFIM 0.036 -0.177 0.184

ID -0.022 0.113 -0.085 6.716

IC -0.121 0.006 -0.005 0.706 0.285

SC 0.002 0.029 -0.026 0.321 0.083

GENDER 0.062 0.056 -0.012 -0.089 -0.009

ESTIMATED COVARIANCE MATRIX FOR THE LATENT VARIABLES

SC GENDER

________ ________

SC 0.048

GENDER 0.007 0.247

S.E. FOR ESTIMATED COVARIANCE MATRIX FOR THE LATENT VARIABLES

SR12 SR13 SR14 IC12 IC13

________ ________ ________ ________ ________

SR12 0.053

SR13 0.026 0.021

SR14 0.025 0.016 0.019

IC12 0.021 0.010 0.000 0.049

IC13 0.010 0.012 0.000 0.010 0.050

IC14 0.000 0.000 0.014 0.000 0.000

DFIM1 0.050 0.028 0.025 0.052 0.000

DFIM2 0.028 0.024 0.016 0.000 0.052

DFIM3 0.025 0.016 0.024 0.000 0.000

SD 0.033 0.020 0.021 0.000 0.000

SC2 0.033 0.021 0.022 0.000 0.000

IDFIM 0.046 0.028 0.025 0.000 0.000

SDFIM 0.043 0.026 0.022 0.000 0.000

ID 0.119 0.078 0.083 0.000 0.000

IC 0.049 0.024 0.025 0.000 0.000

SC 0.024 0.011 0.011 0.000 0.000

GENDER 0.018 0.011 0.011 0.000 0.000

S.E. FOR ESTIMATED COVARIANCE MATRIX FOR THE LATENT VARIABLES

IC14 DFIM1 DFIM2 DFIM3 SD

________ ________ ________ ________ ________

IC14 0.047

DFIM1 0.000 0.074

DFIM2 0.000 0.028 0.066

DFIM3 0.052 0.025 0.016 0.066

SD 0.000 0.033 0.020 0.021 0.063

SC2 0.000 0.033 0.021 0.022 0.038

IDFIM 0.000 0.046 0.028 0.025 0.033

SDFIM 0.000 0.043 0.026 0.022 0.034

ID 0.000 0.119 0.078 0.083 0.268

IC 0.000 0.049 0.024 0.025 0.002

SC 0.000 0.024 0.011 0.011 0.001

GENDER 0.000 0.018 0.011 0.011 0.022

S.E. FOR ESTIMATED COVARIANCE MATRIX FOR THE LATENT VARIABLES

SC2 IDFIM SDFIM ID IC

________ ________ ________ ________ ________

SC2 0.055

IDFIM 0.033 0.046

SDFIM 0.035 0.043 0.051

ID 0.027 0.119 0.126 1.421

IC 0.058 0.049 0.052 0.425 0.097

SC 0.004 0.024 0.024 0.156 0.013

GENDER 0.026 0.018 0.018 0.094 0.033

S.E. FOR ESTIMATED COVARIANCE MATRIX FOR THE LATENT VARIABLES

SC GENDER

________ ________

SC 0.017

GENDER 0.016 0.018

EST./S.E. FOR ESTIMATED COVARIANCE MATRIX FOR THE LATENT VARIABLES

SR12 SR13 SR14 IC12 IC13

________ ________ ________ ________ ________

SR12 6.736

SR13 4.593 7.386

SR14 3.122 5.080 6.835

IC12 1.257 2.501 0.000 5.378

IC13 2.501 -0.478 0.000 2.501 4.572

IC14 0.000 0.000 -2.185 0.000 0.000

DFIM1 6.601 3.308 3.122 -4.506 0.000

DFIM2 3.308 6.525 5.080 0.000 -4.506

DFIM3 3.122 5.080 6.691 0.000 0.000

SD -0.284 -0.590 -0.579 0.000 0.000

SC2 -0.063 1.451 1.518 0.000 0.000

IDFIM 5.518 3.308 3.122 0.000 0.000

SDFIM -4.170 -0.435 0.291 0.000 0.000

ID 0.949 0.468 0.341 0.000 0.000

IC 0.121 0.068 0.046 0.000 0.000

SC 1.200 0.520 0.271 0.000 0.000

GENDER 3.104 4.134 3.911 0.000 0.000

EST./S.E. FOR ESTIMATED COVARIANCE MATRIX FOR THE LATENT VARIABLES

IC14 DFIM1 DFIM2 DFIM3 SD

________ ________ ________ ________ ________

IC14 4.418

DFIM1 0.000 7.650

DFIM2 0.000 3.308 5.942

DFIM3 -4.506 3.122 5.080 5.958

SD 0.000 -0.284 -0.590 -0.579 4.461

SC2 0.000 -0.063 1.451 1.518 3.512

IDFIM 0.000 5.518 3.308 3.122 -0.284

SDFIM 0.000 -4.170 -0.435 0.291 -0.074

ID 0.000 0.949 0.468 0.341 -2.096

IC 0.000 0.121 0.068 0.046 -0.209

SC 0.000 1.200 0.520 0.271 0.291

GENDER 0.000 3.104 4.134 3.911 0.408

EST./S.E. FOR ESTIMATED COVARIANCE MATRIX FOR THE LATENT VARIABLES

SC2 IDFIM SDFIM ID IC

________ ________ ________ ________ ________

SC2 5.706

IDFIM -0.063 5.518

SDFIM 1.031 -4.170 3.620

ID -0.820 0.949 -0.674 4.727

IC -2.076 0.121 -0.093 1.659 2.948

SC 0.451 1.200 -1.104 2.056 6.467

GENDER 2.396 3.104 -0.632 -0.944 -0.276

EST./S.E. FOR ESTIMATED COVARIANCE MATRIX FOR THE LATENT VARIABLES

SC GENDER

________ ________

SC 2.904

GENDER 0.417 13.838

TWO-TAILED P-VALUE FOR ESTIMATED COVARIANCE MATRIX FOR THE LATENT VARIABLES

SR12 SR13 SR14 IC12 IC13

________ ________ ________ ________ ________

SR12 0.000

SR13 0.000 0.000

SR14 0.002 0.000 0.000

IC12 0.209 0.012 1.000 0.000

IC13 0.012 0.632 1.000 0.012 0.000

IC14 1.000 1.000 0.029 1.000 1.000

DFIM1 0.000 0.001 0.002 0.000 1.000

DFIM2 0.001 0.000 0.000 1.000 0.000

DFIM3 0.002 0.000 0.000 1.000 1.000

SD 0.777 0.555 0.562 1.000 1.000

SC2 0.949 0.147 0.129 1.000 1.000

IDFIM 0.000 0.001 0.002 1.000 1.000

SDFIM 0.000 0.664 0.771 1.000 1.000

ID 0.343 0.640 0.733 1.000 1.000

IC 0.904 0.946 0.963 1.000 1.000

SC 0.230 0.603 0.787 1.000 1.000

GENDER 0.002 0.000 0.000 1.000 1.000

TWO-TAILED P-VALUE FOR ESTIMATED COVARIANCE MATRIX FOR THE LATENT VARIABLES

IC14 DFIM1 DFIM2 DFIM3 SD

________ ________ ________ ________ ________

IC14 0.000

DFIM1 1.000 0.000

DFIM2 1.000 0.001 0.000

DFIM3 0.000 0.002 0.000 0.000

SD 1.000 0.777 0.555 0.562 0.000

SC2 1.000 0.949 0.147 0.129 0.000

IDFIM 1.000 0.000 0.001 0.002 0.777

SDFIM 1.000 0.000 0.664 0.771 0.941

ID 1.000 0.343 0.640 0.733 0.036

IC 1.000 0.904 0.946 0.963 0.834

SC 1.000 0.230 0.603 0.787 0.771

GENDER 1.000 0.002 0.000 0.000 0.684

TWO-TAILED P-VALUE FOR ESTIMATED COVARIANCE MATRIX FOR THE LATENT VARIABLES

SC2 IDFIM SDFIM ID IC

________ ________ ________ ________ ________

SC2 0.000

IDFIM 0.949 0.000

SDFIM 0.303 0.000 0.000

ID 0.412 0.343 0.500 0.000

IC 0.038 0.904 0.926 0.097 0.003

SC 0.652 0.230 0.269 0.040 0.000

GENDER 0.017 0.002 0.527 0.345 0.783

TWO-TAILED P-VALUE FOR ESTIMATED COVARIANCE MATRIX FOR THE LATENT VARIABLES

SC GENDER

________ ________

SC 0.004

GENDER 0.677 0.000

ESTIMATED CORRELATION MATRIX FOR THE LATENT VARIABLES

SR12 SR13 SR14 IC12 IC13

________ ________ ________ ________ ________

SR12 1.000

SR13 0.509 1.000

SR14 0.359 0.588 1.000

IC12 0.088 0.126 0.000 1.000

IC13 0.088 -0.030 0.000 0.103 1.000

IC14 0.000 0.000 -0.186 0.000 0.000

DFIM1 0.734 0.318 0.285 -0.612 0.000

DFIM2 0.250 0.645 0.366 0.000 -0.783

DFIM3 0.204 0.335 0.704 0.000 0.000

SD -0.030 -0.057 -0.063 0.000 0.000

SC2 -0.006 0.138 0.168 0.000 0.000

IDFIM 0.845 0.475 0.425 0.000 0.000

SDFIM -0.694 -0.067 0.042 0.000 0.000

ID 0.073 0.036 0.030 0.000 0.000

IC 0.019 0.008 0.006 0.000 0.000

SC 0.223 0.064 0.038 0.000 0.000

GENDER 0.189 0.235 0.250 0.000 0.000

ESTIMATED CORRELATION MATRIX FOR THE LATENT VARIABLES

IC14 DFIM1 DFIM2 DFIM3 SD

________ ________ ________ ________ ________

IC14 1.000

DFIM1 0.000 1.000

DFIM2 0.000 0.198 1.000

DFIM3 -0.829 0.162 0.209 1.000

SD 0.000 -0.024 -0.035 -0.036 1.000

SC2 0.000 -0.005 0.086 0.096 0.456

IDFIM 0.000 0.670 0.296 0.242 -0.035

SDFIM 0.000 -0.550 -0.042 0.024 -0.011

ID 0.000 0.058 0.022 0.017 -0.409

IC 0.000 0.015 0.005 0.003 -0.001

SC 0.000 0.177 0.040 0.022 0.002

GENDER 0.000 0.150 0.146 0.143 0.034

ESTIMATED CORRELATION MATRIX FOR THE LATENT VARIABLES

SC2 IDFIM SDFIM ID IC

________ ________ ________ ________ ________

SC2 1.000

IDFIM -0.007 1.000

SDFIM 0.149 -0.821 1.000

ID -0.015 0.087 -0.076 1.000

IC -0.407 0.022 -0.021 0.510 1.000

SC 0.013 0.264 -0.279 0.566 0.710

GENDER 0.224 0.224 -0.054 -0.069 -0.034

ESTIMATED CORRELATION MATRIX FOR THE LATENT VARIABLES

SC GENDER

________ ________

SC 1.000

GENDER 0.060 1.000

S.E. FOR ESTIMATED CORRELATION MATRIX FOR THE LATENT VARIABLES

SR12 SR13 SR14 IC12 IC13

________ ________ ________ ________ ________

SR12 0.000

SR13 0.079 0.000

SR14 0.102 0.094 0.000

IC12 0.068 0.052 0.000 0.000

IC13 0.036 0.062 0.000 0.044 0.000

IC14 0.000 0.000 0.083 0.000 0.000

DFIM1 0.043 0.081 0.080 0.064 0.000

DFIM2 0.066 0.048 0.064 0.000 0.035

DFIM3 0.060 0.060 0.047 0.000 0.000

SD 0.105 0.096 0.109 0.000 0.000

SC2 0.099 0.097 0.112 0.000 0.000

IDFIM 0.039 0.109 0.116 0.000 0.000

SDFIM 0.054 0.153 0.141 0.000 0.000

ID 0.076 0.077 0.089 0.000 0.000

IC 0.155 0.114 0.131 0.000 0.000

SC 0.176 0.125 0.142 0.000 0.000

GENDER 0.057 0.052 0.059 0.000 0.000

S.E. FOR ESTIMATED CORRELATION MATRIX FOR THE LATENT VARIABLES

IC14 DFIM1 DFIM2 DFIM3 SD

________ ________ ________ ________ ________

IC14 0.000

DFIM1 0.000 0.000

DFIM2 0.000 0.057 0.000

DFIM3 0.036 0.050 0.045 0.000

SD 0.000 0.084 0.060 0.063 0.000

SC2 0.000 0.078 0.061 0.065 0.130

IDFIM 0.000 0.053 0.074 0.069 0.125

SDFIM 0.000 0.063 0.096 0.080 0.148

ID 0.000 0.061 0.048 0.051 0.130

IC 0.000 0.123 0.071 0.074 0.006

SC 0.000 0.139 0.078 0.081 0.007

GENDER 0.000 0.045 0.034 0.035 0.084

S.E. FOR ESTIMATED CORRELATION MATRIX FOR THE LATENT VARIABLES

SC2 IDFIM SDFIM ID IC

________ ________ ________ ________ ________

SC2 0.000

IDFIM 0.117 0.000

SDFIM 0.146 0.045 0.000

ID 0.018 0.090 0.112 0.000

IC 0.194 0.183 0.225 0.268 0.000

SC 0.031 0.207 0.238 0.172 0.088

GENDER 0.088 0.065 0.084 0.072 0.122

S.E. FOR ESTIMATED CORRELATION MATRIX FOR THE LATENT VARIABLES

SC GENDER

________ ________

SC 0.000

GENDER 0.147 0.000

EST./S.E. FOR ESTIMATED CORRELATION MATRIX FOR THE LATENT VARIABLES

SR12 SR13 SR14 IC12 IC13

________ ________ ________ ________ ________

SR12 999.000

SR13 6.417 999.000

SR14 3.524 6.248 999.000

IC12 1.291 2.438 0.000 999.000

IC13 2.435 -0.479 0.000 2.327 999.000

IC14 0.000 0.000 -2.251 0.000 0.000

DFIM1 17.046 3.930 3.543 -9.509 0.000

DFIM2 3.794 13.351 5.703 0.000 -22.226

DFIM3 3.398 5.562 14.906 0.000 0.000

SD -0.284 -0.586 -0.579 0.000 0.000

SC2 -0.063 1.431 1.505 0.000 0.000

IDFIM 21.737 4.351 3.667 0.000 0.000

SDFIM -12.954 -0.439 0.297 0.000 0.000

ID 0.959 0.466 0.340 0.000 0.000

IC 0.121 0.068 0.046 0.000 0.000

SC 1.272 0.513 0.267 0.000 0.000

GENDER 3.342 4.554 4.223 0.000 0.000

EST./S.E. FOR ESTIMATED CORRELATION MATRIX FOR THE LATENT VARIABLES

IC14 DFIM1 DFIM2 DFIM3 SD

________ ________ ________ ________ ________

IC14 999.000

DFIM1 0.000 999.000

DFIM2 0.000 3.467 999.000

DFIM3 -23.198 3.256 4.603 999.000

SD 0.000 -0.284 -0.583 -0.574 999.000

SC2 0.000 -0.063 1.403 1.468 3.498

IDFIM 0.000 12.623 3.974 3.523 -0.285

SDFIM 0.000 -8.714 -0.436 0.297 -0.074

ID 0.000 0.949 0.465 0.340 -3.161

IC 0.000 0.121 0.068 0.046 -0.211

SC 0.000 1.278 0.512 0.268 0.289

GENDER 0.000 3.347 4.296 4.024 0.410

EST./S.E. FOR ESTIMATED CORRELATION MATRIX FOR THE LATENT VARIABLES

SC2 IDFIM SDFIM ID IC

________ ________ ________ ________ ________

SC2 999.000

IDFIM -0.063 999.000

SDFIM 1.021 -18.285 999.000

ID -0.840 0.960 -0.680 999.000

IC -2.104 0.121 -0.093 1.904 999.000

SC 0.439 1.275 -1.172 3.292 8.092

GENDER 2.544 3.423 -0.642 -0.959 -0.280

EST./S.E. FOR ESTIMATED CORRELATION MATRIX FOR THE LATENT VARIABLES

SC GENDER

________ ________

SC 999.000

GENDER 0.408 999.000

TWO-TAILED P-VALUE FOR ESTIMATED CORRELATION MATRIX FOR THE LATENT VARIABLES

SR12 SR13 SR14 IC12 IC13

________ ________ ________ ________ ________

SR12 0.000

SR13 0.000 0.000

SR14 0.000 0.000 0.000

IC12 0.197 0.015 1.000 0.000

IC13 0.015 0.632 1.000 0.020 0.000

IC14 1.000 1.000 0.024 1.000 1.000

DFIM1 0.000 0.000 0.000 0.000 1.000

DFIM2 0.000 0.000 0.000 1.000 0.000

DFIM3 0.001 0.000 0.000 1.000 1.000

SD 0.776 0.558 0.563 1.000 1.000

SC2 0.949 0.152 0.132 1.000 1.000

IDFIM 0.000 0.000 0.000 1.000 1.000

SDFIM 0.000 0.661 0.767 1.000 1.000

ID 0.338 0.641 0.734 1.000 1.000

IC 0.904 0.946 0.963 1.000 1.000

SC 0.203 0.608 0.789 1.000 1.000

GENDER 0.001 0.000 0.000 1.000 1.000

TWO-TAILED P-VALUE FOR ESTIMATED CORRELATION MATRIX FOR THE LATENT VARIABLES

IC14 DFIM1 DFIM2 DFIM3 SD

________ ________ ________ ________ ________

IC14 0.000

DFIM1 1.000 0.000

DFIM2 1.000 0.001 0.000

DFIM3 0.000 0.001 0.000 0.000

SD 1.000 0.777 0.560 0.566 0.000

SC2 1.000 0.949 0.160 0.142 0.000

IDFIM 1.000 0.000 0.000 0.000 0.776

SDFIM 1.000 0.000 0.663 0.766 0.941

ID 1.000 0.343 0.642 0.734 0.002

IC 1.000 0.904 0.946 0.963 0.833

SC 1.000 0.201 0.609 0.789 0.773

GENDER 1.000 0.001 0.000 0.000 0.682

TWO-TAILED P-VALUE FOR ESTIMATED CORRELATION MATRIX FOR THE LATENT VARIABLES

SC2 IDFIM SDFIM ID IC

________ ________ ________ ________ ________

SC2 0.000

IDFIM 0.949 0.000

SDFIM 0.307 0.000 0.000

ID 0.401 0.337 0.496 0.000

IC 0.035 0.904 0.926 0.057 0.000

SC 0.661 0.202 0.241 0.001 0.000

GENDER 0.011 0.001 0.521 0.338 0.779

TWO-TAILED P-VALUE FOR ESTIMATED CORRELATION MATRIX FOR THE LATENT VARIABLES

SC GENDER

________ ________

SC 0.000

GENDER 0.683 0.000

DIAGRAM INFORMATION

Use View Diagram under the Diagram menu in the Mplus Editor to view the diagram.

If running Mplus from the Mplus Diagrammer, the diagram opens automatically.

Diagram output

u:\windows\dcn r&r\r&r analyses\prediction model\latent difference growth model predicting au two-part.dgm

Beginning Time: 15:37:30

Ending Time: 15:37:46

Elapsed Time: 00:00:16

MUTHEN & MUTHEN

3463 Stoner Ave.

Los Angeles, CA 90066

Tel: (310) 391-9971

Fax: (310) 391-8971

Web: www.StatModel.com

Support: Support@StatModel.com

Copyright (c) 1998-2018 Muthen & Muthen
