# Supplementary material for: Mind the gap: A review and recommendations for statistically evaluating Dual Systems models of adolescent risk behavior
Source: Dev Cogn Neurosci. 2019 Jul 25;39:100681. doi: 10.1016/j.dcn.2019.100681 (PMC6969358; doi:10.1016/j.dcn.2019.100681)
Supplement: Supplementary file 4 [file mmc4.docx]

Mplus VERSION 8.2

MUTHEN & MUTHEN

04/05/2019 3:44 PM

INPUT INSTRUCTIONS

Title: LDS Growth Model and Alcohol Two-Part Model

Data: file is predictR&R.dat;

Variable:

NAMES ARE

subject gender sr12r1 sr12r2 sr12r3 sr13r1 sr13r2 sr13r3

sr14r1 sr14r2 sr14r3 ic12r1 ic12r2 ic12r3 ic13r1 ic13r2

ic13r3 ic14r1 ic14r2 ic14r3 mard12-mard20 marc12-marc20;

!SR=sensitivity to reward, IC=inhibitory control

!12=age 12, 13=age 13, 14=age 14

!r1=response block 1, r2=response block 2, r3=response block 3

USEV

ic12r1-ic12r3 ic13r1-ic13r3 ic14r1-ic14r3

sr12r1-sr12r3 sr13r1-sr13r3 sr14r1-sr14r3

mard12-mard20 marc12-marc20 gender;

missing are .;

CATEGORICAL ARE mard13-mard20;

Define:

!Log-Transformed Continuous Marijuana Use

marc13=log(marc13+1);

marc14=log(marc14+1);

marc15=log(marc15+1);

marc16=log(marc16+1);

marc17=log(marc17+1);

marc18=log(marc18+1);

marc19=log(marc19+1);

marc20=log(marc20+1);

Analysis:

estimator is MLR;

model=nocovariances;

mconvergence=.01;

Model:

!Sensitivity to Reward (PSRT) Measurement Model

sr12 by sr12r1

sr12r2 (sr2)

sr12r3 ;

sr13 by sr13r1

sr13r2 (sr2)

sr13r3 (sr3);

sr14 by sr14r1

sr14r2 (sr2)

sr14r3 (sr3);

!Constraining Item Intercepts Within and Across Time for

!Mean Structure Identification

[sr12r1 sr13r1 sr14r1] (sri1);

[sr12r2 sr13r2 sr14r2] (sri1);

[sr12r3 sr13r3 sr14r3] (sri1);

!Constraining Across time Item Residual Variances

sr13r1 sr14r1 (srr1);

sr12r2 sr13r2 sr14r2 (srr2);

sr13r3 sr14r3 (srr3);

!Stop Signal Task Measurment Model

ic12 by ic12r1

ic12r2 (ic2)

ic12r3 (ic3);

ic13 by ic13r1

ic13r2 (ic2)

ic13r3 (ic3);

ic14 by ic14r1

ic14r2 (ic2)

ic14r3 (ic3);

!Constraining Item Intercepts Within and Across Time for

!Mean Structure Identification

[ic12r1 ic13r1 ic14r1] (ici1);

[ic12r2 ic13r2 ic14r2] (ici1);

[ic12r3 ic13r3 ic14r3] (ici1);

!Constraining Across time Item Residual Variances

ic13r1 ic14r1 (icr1);

ic12r2 ic13r2 (icr2);

ic13r3 ic14r3 (icr3);

!Specifying Latent Difference Score

sr12 on ic12@1;

sr13 on ic13@1;

sr14 on ic14@1;

dfim1 by sr12@1;

dfim2 by sr13@1;

dfim3 by sr14@1;

!Means and Variances of Sensitivity to Reward Constrained to 0

!for Model Identification

sr12-sr14@0;

[sr12-sr14@0];

[ic12-ic14];

!Latent Difference Score Variances

dfim1 dfim2 dfim3 (dfimres);

!Constrained Change Score Intercepts for Growth Model

[dfim1@0 dfim2@0 dfim3@0];

!Covariance Between Difference Scores and Inhibitory Control

ic12 with dfim1 (icwdfim);

ic13 with dfim2 (icwdfim);

ic14 with dfim3 (icwdfim);

!Latent Growth of Difference Scores

idfim sdfim | dfim1@0 dfim2* dfim3@1;

idfim with sdfim;

!Covariances added based on Modification Indices

SR13R3 WITH SR13R1;

SR14R3 WITH SR14R2;

SR13R3 WITH SR13R2;

SR14R2 WITH SR13R1;

IC13 WITH IC12;

IC14R1 WITH IC12R1;

IC13R3 WITH IC13R1;

IC13R3 WITH IC12R2;

IC13R3 WITH IC12R3;

IC14R3 WITH IC12R1;

!Marijuana Two-Part Growth Model With Random Effects

!Dichotomous Growth Model ages 13-20

id sd | mard13@0 mard14@1 mard15@2 mard16@3 mard17@4 mard18*

mard19@6 mard20*;

sd BY mard18*5.74545;

sd BY mard20*5.98383;

!Continuous Growth Model ages 13-20

ic sc | marc13@0 marc14@1 marc15@2 marc16* marc17@4

marc18* marc19@6 marc20*;

sc BY marc16*2.42648;

sc BY marc18*5.38491;

sc BY marc20*6.28684;

!Covariances Between Growth Factors

id WITH sd*-0.87695;

id WITH ic*1.93801;

id WITH sc@0;

ic WITH sc@0;

sd WITH ic*-0.22456;

sd WITH sc*0.12190;

!Continuous Marijuana Use Intercepts Constrained to 0 for Model

!Identification

[ marc13@0 ];

[ marc14@0 ];

[ marc15@0 ];

[ marc16@0 ];

[ marc17@0 ];

[ marc18@0 ];

[ marc19@0 ];

[ marc20@0 ];

[ id@0 ];

!Growth Factor Means Start Values

[ sd*0.86006 ];

[ ic*0.31980 ];

[ sc*0.33860 ];

!Dichotomous Marijuana Use Thresholds Start Values

[ mard13$1*5.18694 ] (25);

[ mard14$1*5.18694 ] (25);

[ mard15$1*5.18694 ] (25);

[ mard16$1*5.18694 ] (25);

[ mard17$1*5.18694 ] (25);

[ mard18$1*5.18694 ] (25);

[ mard19$1*5.18694 ] (25);

[ mard20$1*5.18694 ] (25);

!Start Values for Variances of Continous Marijuana Use and

!Marijuana Use Growth Factors

marc13*0.43001;

marc14*0.33564;

marc15*0.94733;

marc16*0.98942;

marc17*1.45900;

marc18*1.11107;

marc19*0.94213;

marc20*1.31857;

sc*0.09352;

id*7.00211;

sd*0.38070;

ic*0.82317;

!LDS Growth Model Covariances with Marijuana Use

id sd ic sc with idfim sdfim;

!Controlling for Gender

id sd ic sc idfim sdfim on gender;

Output: res stdyx tech4 sampstat svalues;

*** WARNING

Data set contains cases with missing on all variables except

x-variables. These cases were not included in the analysis.

Number of cases with missing on all variables except x-variables: 4

2 WARNING(S) FOUND IN THE INPUT INSTRUCTIONS

Revised Classes Predicting Alcohol 2-Part DCN R&R

SUMMARY OF ANALYSIS

Number of groups 1

Number of observations 383

Number of dependent variables 34

Number of independent variables 1

Number of continuous latent variables 15

Observed dependent variables

Continuous

MARC13 MARC14 MARC15 MARC16 MARC17 MARC18

MARC19 MARC20 IC12R1 IC12R2 IC12R3 IC13R1

IC13R2 IC13R3 IC14R1 IC14R2 IC14R3 SR12R1

SR12R2 SR12R3 SR13R1 SR13R2 SR13R3 SR14R1

SR14R2 SR14R3

Binary and ordered categorical (ordinal)

MARD13 MARD14 MARD15 MARD16 MARD17 MARD18

MARD19 MARD20

Observed independent variables

GENDER

Continuous latent variables

SR12 SR13 SR14 IC12 IC13 IC14

DFIM1 DFIM2 DFIM3 SD SC IDFIM

SDFIM ID IC

Estimator MLR

Information matrix OBSERVED

Optimization Specifications for the Quasi-Newton Algorithm for

Continuous Outcomes

Maximum number of iterations 100

Convergence criterion 0.100D-05

Optimization Specifications for the EM Algorithm

Maximum number of iterations 500

Convergence criteria

Loglikelihood change 0.100D-02

Relative loglikelihood change 0.100D-05

Derivative 0.100D-02

Optimization Specifications for the M step of the EM Algorithm for

Categorical Latent variables

Number of M step iterations 1

M step convergence criterion 0.100D-02

Basis for M step termination ITERATION

Optimization Specifications for the M step of the EM Algorithm for

Censored, Binary or Ordered Categorical (Ordinal), Unordered

Categorical (Nominal) and Count Outcomes

Number of M step iterations 1

M step convergence criterion 0.100D-02

Basis for M step termination ITERATION

Maximum value for logit thresholds 15

Minimum value for logit thresholds -15

Minimum expected cell size for chi-square 0.100D-01

Maximum number of iterations for H1 2000

Convergence criterion for H1 0.100D-03

Optimization algorithm EMA

Integration Specifications

Type STANDARD

Number of integration points 15

Dimensions of numerical integration 2

Adaptive quadrature ON

Link LOGIT

Cholesky OFF

Input data file(s)

predictR&R.dat

Input data format FREE

SUMMARY OF DATA

Number of missing data patterns 250

Number of y missing data patterns 177

Number of u missing data patterns 60

COVARIANCE COVERAGE OF DATA

Minimum covariance coverage value 0.100

PROPORTION OF DATA PRESENT

Covariance Coverage

MARD13 MARD14 MARD15 MARD16 MARD17

________ ________ ________ ________ ________

MARD13 0.937

MARD14 0.914 0.961

MARD15 0.862 0.875 0.893

MARD16 0.836 0.849 0.815 0.872

MARD17 0.762 0.775 0.739 0.728 0.796

MARD18 0.739 0.752 0.721 0.705 0.640

MARD19 0.781 0.799 0.768 0.755 0.692

MARD20 0.598 0.614 0.590 0.580 0.499

MARC13 0.023 0.023 0.016 0.018 0.016

MARC14 0.081 0.089 0.081 0.068 0.052

MARC15 0.117 0.125 0.125 0.102 0.089

MARC16 0.151 0.151 0.146 0.159 0.120

MARC17 0.232 0.232 0.232 0.219 0.240

MARC18 0.358 0.360 0.355 0.339 0.292

MARC19 0.368 0.376 0.366 0.350 0.324

MARC20 0.305 0.305 0.295 0.290 0.251

IC12R1 0.836 0.856 0.796 0.770 0.713

IC12R2 0.830 0.854 0.789 0.765 0.705

IC12R3 0.828 0.849 0.786 0.762 0.705

IC13R1 0.838 0.828 0.770 0.749 0.684

IC13R2 0.841 0.825 0.773 0.755 0.684

IC13R3 0.843 0.828 0.773 0.757 0.689

IC14R1 0.473 0.483 0.452 0.426 0.423

IC14R2 0.480 0.491 0.460 0.431 0.428

IC14R3 0.478 0.488 0.454 0.428 0.426

SR12R1 0.856 0.877 0.812 0.791 0.728

SR12R2 0.856 0.877 0.812 0.791 0.728

SR12R3 0.856 0.877 0.812 0.791 0.728

SR13R1 0.880 0.864 0.809 0.789 0.715

SR13R2 0.880 0.864 0.809 0.789 0.715

SR13R3 0.880 0.864 0.809 0.789 0.715

SR14R1 0.499 0.509 0.475 0.449 0.444

SR14R2 0.499 0.509 0.475 0.449 0.444

SR14R3 0.499 0.509 0.475 0.449 0.444

GENDER 0.937 0.961 0.893 0.872 0.796

Covariance Coverage

MARD18 MARD19 MARD20 MARC13 MARC14

________ ________ ________ ________ ________

MARD18 0.773

MARD19 0.668 0.812

MARD20 0.480 0.543 0.632

MARC13 0.023 0.018 0.010 0.023

MARC14 0.063 0.060 0.063 0.013 0.089

MARC15 0.094 0.099 0.084 0.010 0.055

MARC16 0.131 0.141 0.112 0.013 0.037

MARC17 0.193 0.214 0.138 0.010 0.031

MARC18 0.371 0.321 0.245 0.021 0.047

MARC19 0.313 0.384 0.279 0.016 0.039

MARC20 0.251 0.274 0.316 0.010 0.039

IC12R1 0.689 0.721 0.564 0.023 0.076

IC12R2 0.681 0.715 0.554 0.023 0.076

IC12R3 0.684 0.713 0.556 0.023 0.070

IC13R1 0.674 0.705 0.533 0.021 0.073

IC13R2 0.679 0.708 0.538 0.023 0.070

IC13R3 0.681 0.708 0.540 0.023 0.070

IC14R1 0.358 0.397 0.431 0.010 0.044

IC14R2 0.360 0.402 0.436 0.010 0.050

IC14R3 0.352 0.399 0.433 0.010 0.047

SR12R1 0.708 0.739 0.572 0.023 0.076

SR12R2 0.708 0.739 0.572 0.023 0.076

SR12R3 0.708 0.739 0.572 0.023 0.076

SR13R1 0.705 0.739 0.564 0.023 0.073

SR13R2 0.705 0.739 0.564 0.023 0.073

SR13R3 0.705 0.739 0.564 0.023 0.073

SR14R1 0.373 0.420 0.454 0.010 0.050

SR14R2 0.373 0.420 0.454 0.010 0.050

SR14R3 0.373 0.420 0.454 0.010 0.050

GENDER 0.773 0.812 0.632 0.023 0.089

Covariance Coverage

MARC15 MARC16 MARC17 MARC18 MARC19

________ ________ ________ ________ ________

MARC15 0.125

MARC16 0.060 0.159

MARC17 0.060 0.091 0.240

MARC18 0.081 0.110 0.151 0.371

MARC19 0.073 0.115 0.154 0.258 0.384

MARC20 0.055 0.084 0.094 0.185 0.217

IC12R1 0.099 0.131 0.209 0.334 0.350

IC12R2 0.099 0.128 0.209 0.329 0.347

IC12R3 0.094 0.123 0.201 0.321 0.337

IC13R1 0.097 0.123 0.201 0.324 0.324

IC13R2 0.099 0.136 0.198 0.329 0.332

IC13R3 0.099 0.133 0.198 0.324 0.326

IC14R1 0.055 0.068 0.112 0.170 0.204

IC14R2 0.060 0.068 0.112 0.172 0.211

IC14R3 0.057 0.065 0.110 0.162 0.204

SR12R1 0.099 0.133 0.217 0.337 0.352

SR12R2 0.099 0.133 0.217 0.337 0.352

SR12R3 0.099 0.133 0.217 0.337 0.352

SR13R1 0.099 0.136 0.206 0.334 0.342

SR13R2 0.099 0.136 0.206 0.334 0.342

SR13R3 0.099 0.136 0.206 0.334 0.342

SR14R1 0.060 0.070 0.115 0.178 0.219

SR14R2 0.060 0.070 0.115 0.178 0.219

SR14R3 0.060 0.070 0.115 0.178 0.219

GENDER 0.125 0.159 0.240 0.371 0.384

Covariance Coverage

MARC20 IC12R1 IC12R2 IC12R3 IC13R1

________ ________ ________ ________ ________

MARC20 0.316

IC12R1 0.290 0.885

IC12R2 0.285 0.856 0.880

IC12R3 0.279 0.854 0.854 0.877

IC13R1 0.272 0.786 0.786 0.775 0.838

IC13R2 0.282 0.789 0.786 0.781 0.812

IC13R3 0.277 0.789 0.789 0.783 0.812

IC14R1 0.222 0.462 0.457 0.457 0.449

IC14R2 0.227 0.470 0.465 0.465 0.457

IC14R3 0.219 0.467 0.462 0.465 0.454

SR12R1 0.287 0.880 0.875 0.872 0.802

SR12R2 0.287 0.880 0.875 0.872 0.802

SR12R3 0.287 0.880 0.875 0.872 0.802

SR13R1 0.287 0.822 0.817 0.815 0.836

SR13R2 0.287 0.822 0.817 0.815 0.836

SR13R3 0.287 0.822 0.817 0.815 0.836

SR14R1 0.235 0.488 0.483 0.483 0.475

SR14R2 0.235 0.488 0.483 0.483 0.475

SR14R3 0.235 0.488 0.483 0.483 0.475

GENDER 0.316 0.885 0.880 0.877 0.838

Covariance Coverage

IC13R2 IC13R3 IC14R1 IC14R2 IC14R3

________ ________ ________ ________ ________

IC13R2 0.841

IC13R3 0.822 0.843

IC14R1 0.454 0.454 0.483

IC14R2 0.457 0.460 0.473 0.491

IC14R3 0.454 0.457 0.470 0.473 0.488

SR12R1 0.807 0.807 0.467 0.475 0.473

SR12R2 0.807 0.807 0.467 0.475 0.473

SR12R3 0.807 0.807 0.467 0.475 0.473

SR13R1 0.838 0.841 0.467 0.475 0.473

SR13R2 0.838 0.841 0.467 0.475 0.473

SR13R3 0.838 0.841 0.467 0.475 0.473

SR14R1 0.475 0.478 0.483 0.491 0.488

SR14R2 0.475 0.478 0.483 0.491 0.488

SR14R3 0.475 0.478 0.483 0.491 0.488

GENDER 0.841 0.843 0.483 0.491 0.488

Covariance Coverage

SR12R1 SR12R2 SR12R3 SR13R1 SR13R2

________ ________ ________ ________ ________

SR12R1 0.906

SR12R2 0.906 0.906

SR12R3 0.906 0.906 0.906

SR13R1 0.843 0.843 0.843 0.880

SR13R2 0.843 0.843 0.843 0.880 0.880

SR13R3 0.843 0.843 0.843 0.880 0.880

SR14R1 0.493 0.493 0.493 0.493 0.493

SR14R2 0.493 0.493 0.493 0.493 0.493

SR14R3 0.493 0.493 0.493 0.493 0.493

GENDER 0.906 0.906 0.906 0.880 0.880

Covariance Coverage

SR13R3 SR14R1 SR14R2 SR14R3 GENDER

________ ________ ________ ________ ________

SR13R3 0.880

SR14R1 0.493 0.509

SR14R2 0.493 0.509 0.509

SR14R3 0.493 0.509 0.509 0.509

GENDER 0.880 0.509 0.509 0.509 1.000

WARNING: THE COVARIANCE COVERAGE FALLS BELOW THE SPECIFIED LIMIT.

PROPORTION OF DATA PRESENT FOR U

Covariance Coverage

MARD13 MARD14 MARD15 MARD16 MARD17

________ ________ ________ ________ ________

MARD13 0.937

MARD14 0.914 0.961

MARD15 0.862 0.875 0.893

MARD16 0.836 0.849 0.815 0.872

MARD17 0.762 0.775 0.739 0.728 0.796

MARD18 0.739 0.752 0.721 0.705 0.640

MARD19 0.781 0.799 0.768 0.755 0.692

MARD20 0.598 0.614 0.590 0.580 0.499

Covariance Coverage

MARD18 MARD19 MARD20

________ ________ ________

MARD18 0.773

MARD19 0.668 0.812

MARD20 0.480 0.543 0.632

PROPORTION OF DATA PRESENT FOR Y

Covariance Coverage

MARC13 MARC14 MARC15 MARC16 MARC17

________ ________ ________ ________ ________

MARC13 0.023

MARC14 0.013 0.089

MARC15 0.010 0.055 0.125

MARC16 0.013 0.037 0.060 0.159

MARC17 0.010 0.031 0.060 0.091 0.240

MARC18 0.021 0.047 0.081 0.110 0.151

MARC19 0.016 0.039 0.073 0.115 0.154

MARC20 0.010 0.039 0.055 0.084 0.094

IC12R1 0.023 0.076 0.099 0.131 0.209

IC12R2 0.023 0.076 0.099 0.128 0.209

IC12R3 0.023 0.070 0.094 0.123 0.201

IC13R1 0.021 0.073 0.097 0.123 0.201

IC13R2 0.023 0.070 0.099 0.136 0.198

IC13R3 0.023 0.070 0.099 0.133 0.198

IC14R1 0.010 0.044 0.055 0.068 0.112

IC14R2 0.010 0.050 0.060 0.068 0.112

IC14R3 0.010 0.047 0.057 0.065 0.110

SR12R1 0.023 0.076 0.099 0.133 0.217

SR12R2 0.023 0.076 0.099 0.133 0.217

SR12R3 0.023 0.076 0.099 0.133 0.217

SR13R1 0.023 0.073 0.099 0.136 0.206

SR13R2 0.023 0.073 0.099 0.136 0.206

SR13R3 0.023 0.073 0.099 0.136 0.206

SR14R1 0.010 0.050 0.060 0.070 0.115

SR14R2 0.010 0.050 0.060 0.070 0.115

SR14R3 0.010 0.050 0.060 0.070 0.115

GENDER 0.023 0.089 0.125 0.159 0.240

Covariance Coverage

MARC18 MARC19 MARC20 IC12R1 IC12R2

________ ________ ________ ________ ________

MARC18 0.371

MARC19 0.258 0.384

MARC20 0.185 0.217 0.316

IC12R1 0.334 0.350 0.290 0.885

IC12R2 0.329 0.347 0.285 0.856 0.880

IC12R3 0.321 0.337 0.279 0.854 0.854

IC13R1 0.324 0.324 0.272 0.786 0.786

IC13R2 0.329 0.332 0.282 0.789 0.786

IC13R3 0.324 0.326 0.277 0.789 0.789

IC14R1 0.170 0.204 0.222 0.462 0.457

IC14R2 0.172 0.211 0.227 0.470 0.465

IC14R3 0.162 0.204 0.219 0.467 0.462

SR12R1 0.337 0.352 0.287 0.880 0.875

SR12R2 0.337 0.352 0.287 0.880 0.875

SR12R3 0.337 0.352 0.287 0.880 0.875

SR13R1 0.334 0.342 0.287 0.822 0.817

SR13R2 0.334 0.342 0.287 0.822 0.817

SR13R3 0.334 0.342 0.287 0.822 0.817

SR14R1 0.178 0.219 0.235 0.488 0.483

SR14R2 0.178 0.219 0.235 0.488 0.483

SR14R3 0.178 0.219 0.235 0.488 0.483

GENDER 0.371 0.384 0.316 0.885 0.880

Covariance Coverage

IC12R3 IC13R1 IC13R2 IC13R3 IC14R1

________ ________ ________ ________ ________

IC12R3 0.877

IC13R1 0.775 0.838

IC13R2 0.781 0.812 0.841

IC13R3 0.783 0.812 0.822 0.843

IC14R1 0.457 0.449 0.454 0.454 0.483

IC14R2 0.465 0.457 0.457 0.460 0.473

IC14R3 0.465 0.454 0.454 0.457 0.470

SR12R1 0.872 0.802 0.807 0.807 0.467

SR12R2 0.872 0.802 0.807 0.807 0.467

SR12R3 0.872 0.802 0.807 0.807 0.467

SR13R1 0.815 0.836 0.838 0.841 0.467

SR13R2 0.815 0.836 0.838 0.841 0.467

SR13R3 0.815 0.836 0.838 0.841 0.467

SR14R1 0.483 0.475 0.475 0.478 0.483

SR14R2 0.483 0.475 0.475 0.478 0.483

SR14R3 0.483 0.475 0.475 0.478 0.483

GENDER 0.877 0.838 0.841 0.843 0.483

Covariance Coverage

IC14R2 IC14R3 SR12R1 SR12R2 SR12R3

________ ________ ________ ________ ________

IC14R2 0.491

IC14R3 0.473 0.488

SR12R1 0.475 0.473 0.906

SR12R2 0.475 0.473 0.906 0.906

SR12R3 0.475 0.473 0.906 0.906 0.906

SR13R1 0.475 0.473 0.843 0.843 0.843

SR13R2 0.475 0.473 0.843 0.843 0.843

SR13R3 0.475 0.473 0.843 0.843 0.843

SR14R1 0.491 0.488 0.493 0.493 0.493

SR14R2 0.491 0.488 0.493 0.493 0.493

SR14R3 0.491 0.488 0.493 0.493 0.493

GENDER 0.491 0.488 0.906 0.906 0.906

Covariance Coverage

SR13R1 SR13R2 SR13R3 SR14R1 SR14R2

________ ________ ________ ________ ________

SR13R1 0.880

SR13R2 0.880 0.880

SR13R3 0.880 0.880 0.880

SR14R1 0.493 0.493 0.493 0.509

SR14R2 0.493 0.493 0.493 0.509 0.509

SR14R3 0.493 0.493 0.493 0.509 0.509

GENDER 0.880 0.880 0.880 0.509 0.509

Covariance Coverage

SR14R3 GENDER

________ ________

SR14R3 0.509

GENDER 0.509 1.000

THE COVARIANCE COVERAGE FALLS BELOW THE SPECIFIED LIMIT. THE MISSING

DATA EM ALGORITHM WILL NOT BE INITIATED. CHECK YOUR DATA OR LOWER THE

COVARIANCE COVERAGE LIMIT.

UNIVARIATE PROPORTIONS AND COUNTS FOR CATEGORICAL VARIABLES

MARD13

Category 1 0.975 350.000

Category 2 0.025 9.000

MARD14

Category 1 0.908 334.000

Category 2 0.092 34.000

MARD15

Category 1 0.860 294.000

Category 2 0.140 48.000

MARD16

Category 1 0.817 273.000

Category 2 0.183 61.000

MARD17

Category 1 0.698 213.000

Category 2 0.302 92.000

MARD18

Category 1 0.520 154.000

Category 2 0.480 142.000

MARD19

Category 1 0.527 164.000

Category 2 0.473 147.000

MARD20

Category 1 0.500 121.000

Category 2 0.500 121.000

SAMPLE STATISTICS

UNIVARIATE SAMPLE STATISTICS

UNIVARIATE HIGHER-ORDER MOMENT DESCRIPTIVE STATISTICS

Variable/ Mean/ Skewness/ Minimum/ % with Percentiles

Sample Size Variance Kurtosis Maximum Min/Max 20%/60% 40%/80% Median

MARC13 1.533 -0.214 0.693 22.22% 0.693 1.386 1.609

9.000 0.298 -0.906 2.398 11.11% 1.609 1.946

MARC14 1.743 0.413 0.693 23.53% 0.693 1.099 1.386

34.000 0.828 -1.221 3.434 5.88% 1.792 2.773

MARC15 2.319 0.687 0.693 20.83% 0.693 1.609 2.197

48.000 2.097 -0.637 5.707 2.08% 2.303 3.434

MARC16 2.509 0.734 0.693 11.48% 1.386 2.079 2.303

61.000 1.917 -0.282 5.903 1.64% 2.398 3.932

MARC17 2.793 0.454 0.693 10.87% 0.916 1.792 2.398

92.000 3.329 -1.250 5.903 1.09% 2.944 4.875

MARC18 3.220 -0.025 0.693 1.41% 0.916 2.565 3.434

142.000 3.787 -1.585 5.889 12.68% 3.970 5.460

MARC19 3.383 -0.119 0.916 34.69% 0.916 2.565 3.434

147.000 4.102 -1.655 5.889 17.69% 4.875 5.460

MARC20 3.605 -0.266 0.916 28.93% 0.916 3.434 3.970

121.000 3.970 -1.547 5.889 20.66% 4.875 5.889

IC12R1 -0.031 -0.868 -3.200 1.77% -0.707 -0.139 0.033

339.000 1.075 1.081 2.044 0.29% 0.280 0.813

IC12R2 -0.289 -1.156 -3.608 2.37% -0.893 -0.301 -0.146

337.000 1.117 1.608 1.869 0.30% 0.064 0.493

IC12R3 -0.217 -0.780 -3.661 0.89% -0.958 -0.280 -0.093

336.000 1.217 0.773 2.062 0.30% 0.163 0.579

IC13R1 0.325 -0.243 -2.001 0.31% -0.232 0.170 0.304

321.000 0.488 0.381 2.041 0.31% 0.473 0.906

IC13R2 0.184 -0.651 -2.845 0.93% -0.449 0.077 0.272

322.000 0.736 1.178 1.986 0.31% 0.411 0.844

IC13R3 0.117 -0.854 -2.763 2.17% -0.452 -0.005 0.211

323.000 0.767 1.336 1.835 0.31% 0.383 0.789

IC14R1 0.369 -0.628 -1.986 1.08% -0.129 0.229 0.473

185.000 0.527 1.211 2.041 0.54% 0.610 0.875

IC14R2 0.308 -0.615 -2.243 1.06% -0.201 0.177 0.299

188.000 0.452 1.503 1.728 0.53% 0.442 0.910

IC14R3 0.215 -0.497 -2.340 0.53% -0.373 0.070 0.246

187.000 0.586 1.149 2.065 0.53% 0.390 0.889

SR12R1 0.670 1.169 -1.982 0.29% 0.127 0.430 0.532

347.000 0.627 4.247 4.720 0.29% 0.707 1.148

SR12R2 0.574 0.663 -1.727 0.29% 0.033 0.341 0.469

347.000 0.572 1.746 3.573 0.29% 0.645 1.110

SR12R3 0.624 0.370 -1.837 0.29% 0.107 0.433 0.534

347.000 0.532 1.939 4.103 0.29% 0.699 1.104

SR13R1 0.482 0.836 -1.012 0.30% 0.115 0.300 0.406

337.000 0.292 1.992 2.863 0.30% 0.512 0.837

SR13R2 0.506 0.832 -1.550 0.30% 0.109 0.310 0.420

337.000 0.335 1.781 2.906 0.30% 0.503 0.907

SR13R3 0.597 1.181 -0.879 0.30% 0.160 0.379 0.479

337.000 0.355 2.585 3.236 0.30% 0.643 0.964

SR14R1 0.526 0.815 -0.715 0.51% 0.185 0.346 0.468

195.000 0.221 1.999 2.509 0.51% 0.581 0.854

SR14R2 0.574 0.953 -0.657 0.51% 0.131 0.366 0.488

195.000 0.325 1.837 2.885 0.51% 0.648 0.975

SR14R3 0.623 0.766 -0.823 0.51% 0.189 0.410 0.581

195.000 0.300 1.074 2.729 0.51% 0.687 0.994

GENDER 0.446 0.215 0.000 55.35% 0.000 0.000 0.000

383.000 0.247 -1.954 1.000 44.65% 1.000 1.000

THE MODEL ESTIMATION TERMINATED NORMALLY

MODEL FIT INFORMATION

Number of Free Parameters 80

Loglikelihood

H0 Value -7282.463

H0 Scaling Correction Factor 1.2199

for MLR

Information Criteria

Akaike (AIC) 14724.926

Bayesian (BIC) 15040.769

Sample-Size Adjusted BIC 14786.941

(n* = (n + 2) / 24)

MODEL RESULTS

Two-Tailed

Estimate S.E. Est./S.E. P-Value

ID |

MARD13 1.000 0.000 999.000 999.000

MARD14 1.000 0.000 999.000 999.000

MARD15 1.000 0.000 999.000 999.000

MARD16 1.000 0.000 999.000 999.000

MARD17 1.000 0.000 999.000 999.000

MARD18 1.000 0.000 999.000 999.000

MARD19 1.000 0.000 999.000 999.000

MARD20 1.000 0.000 999.000 999.000

SD |

MARD13 0.000 0.000 999.000 999.000

MARD14 1.000 0.000 999.000 999.000

MARD15 2.000 0.000 999.000 999.000

MARD16 3.000 0.000 999.000 999.000

MARD17 4.000 0.000 999.000 999.000

MARD18 5.734 0.223 25.727 0.000

MARD19 6.000 0.000 999.000 999.000

MARD20 5.924 0.288 20.603 0.000

IC |

MARC13 1.000 0.000 999.000 999.000

MARC14 1.000 0.000 999.000 999.000

MARC15 1.000 0.000 999.000 999.000

MARC16 1.000 0.000 999.000 999.000

MARC17 1.000 0.000 999.000 999.000

MARC18 1.000 0.000 999.000 999.000

MARC19 1.000 0.000 999.000 999.000

MARC20 1.000 0.000 999.000 999.000

SC |

MARC13 0.000 0.000 999.000 999.000

MARC14 1.000 0.000 999.000 999.000

MARC15 2.000 0.000 999.000 999.000

MARC16 2.653 0.279 9.511 0.000

MARC17 4.000 0.000 999.000 999.000

MARC18 5.364 0.189 28.344 0.000

MARC19 6.000 0.000 999.000 999.000

MARC20 6.270 0.217 28.933 0.000

IDFIM |

DFIM1 1.000 0.000 999.000 999.000

DFIM2 1.000 0.000 999.000 999.000

DFIM3 1.000 0.000 999.000 999.000

SDFIM |

DFIM1 0.000 0.000 999.000 999.000

DFIM2 0.889 0.110 8.107 0.000

DFIM3 1.000 0.000 999.000 999.000

SR12 BY

SR12R1 1.000 0.000 999.000 999.000

SR12R2 1.014 0.029 34.777 0.000

SR12R3 0.949 0.053 17.968 0.000

SR13 BY

SR13R1 1.000 0.000 999.000 999.000

SR13R2 1.014 0.029 34.777 0.000

SR13R3 0.916 0.036 25.574 0.000

SR14 BY

SR14R1 1.000 0.000 999.000 999.000

SR14R2 1.014 0.029 34.777 0.000

SR14R3 0.916 0.036 25.574 0.000

IC12 BY

IC12R1 1.000 0.000 999.000 999.000

IC12R2 1.333 0.147 9.040 0.000

IC12R3 1.370 0.141 9.744 0.000

IC13 BY

IC13R1 1.000 0.000 999.000 999.000

IC13R2 1.333 0.147 9.040 0.000

IC13R3 1.370 0.141 9.744 0.000

IC14 BY

IC14R1 1.000 0.000 999.000 999.000

IC14R2 1.333 0.147 9.040 0.000

IC14R3 1.370 0.141 9.744 0.000

DFIM1 BY

SR12 1.000 0.000 999.000 999.000

DFIM2 BY

SR13 1.000 0.000 999.000 999.000

DFIM3 BY

SR14 1.000 0.000 999.000 999.000

SR12 ON

IC12 1.000 0.000 999.000 999.000

SR13 ON

IC13 1.000 0.000 999.000 999.000

SR14 ON

IC14 1.000 0.000 999.000 999.000

ID ON

GENDER 0.279 0.464 0.600 0.548

SD ON

GENDER 0.128 0.104 1.226 0.220

IC ON

GENDER -0.351 0.285 -1.232 0.218

SC ON

GENDER 0.266 0.064 4.127 0.000

IDFIM ON

GENDER 0.227 0.071 3.188 0.001

SDFIM ON

GENDER -0.043 0.075 -0.571 0.568

IC12 WITH

DFIM1 -0.235 0.052 -4.503 0.000

IC13 0.026 0.010 2.586 0.010

IC13 WITH

DFIM2 -0.235 0.052 -4.503 0.000

IC14 WITH

DFIM3 -0.235 0.052 -4.503 0.000

IDFIM WITH

SDFIM -0.173 0.042 -4.079 0.000

ID -0.040 0.168 -0.241 0.810

SD 0.057 0.039 1.483 0.138

IC 0.031 0.092 0.333 0.739

SC -0.003 0.022 -0.153 0.878

ID WITH

SD -0.779 0.265 -2.942 0.003

IC 1.986 0.504 3.942 0.000

SC 0.000 0.000 999.000 999.000

SDFIM -0.054 0.156 -0.343 0.731

IC WITH

SC 0.000 0.000 999.000 999.000

SD -0.182 0.106 -1.727 0.084

SDFIM -0.087 0.136 -0.641 0.521

SD WITH

SC 0.103 0.027 3.830 0.000

SDFIM -0.019 0.036 -0.524 0.600

SC WITH

SDFIM 0.023 0.028 0.831 0.406

SR13R3 WITH

SR13R1 -0.045 0.019 -2.444 0.015

SR13R2 0.064 0.024 2.737 0.006

IC14R3 WITH

IC12R1 0.137 0.040 3.451 0.001

SR14R3 WITH

SR14R2 0.078 0.019 3.998 0.000

IC14R1 WITH

IC12R1 0.084 0.032 2.602 0.009

IC13R3 WITH

IC13R1 -0.099 0.042 -2.342 0.019

IC12R2 0.110 0.047 2.321 0.020

IC12R3 0.099 0.042 2.361 0.018

SR14R2 WITH

SR13R1 -0.028 0.013 -2.083 0.037

Means

IC12 -0.746 0.234 -3.193 0.001

IC13 -0.434 0.206 -2.103 0.036

IC14 -0.342 0.203 -1.689 0.091

Intercepts

MARC13 0.000 0.000 999.000 999.000

MARC14 0.000 0.000 999.000 999.000

MARC15 0.000 0.000 999.000 999.000

MARC16 0.000 0.000 999.000 999.000

MARC17 0.000 0.000 999.000 999.000

MARC18 0.000 0.000 999.000 999.000

MARC19 0.000 0.000 999.000 999.000

MARC20 0.000 0.000 999.000 999.000

IC12R1 0.751 0.214 3.511 0.000

IC12R2 0.751 0.214 3.511 0.000

IC12R3 0.751 0.214 3.511 0.000

IC13R1 0.751 0.214 3.511 0.000

IC13R2 0.751 0.214 3.511 0.000

IC13R3 0.751 0.214 3.511 0.000

IC14R1 0.751 0.214 3.511 0.000

IC14R2 0.751 0.214 3.511 0.000

IC14R3 0.751 0.214 3.511 0.000

SR12R1 1.261 0.235 5.357 0.000

SR12R2 1.261 0.235 5.357 0.000

SR12R3 1.261 0.235 5.357 0.000

SR13R1 1.261 0.235 5.357 0.000

SR13R2 1.261 0.235 5.357 0.000

SR13R3 1.261 0.235 5.357 0.000

SR14R1 1.261 0.235 5.357 0.000

SR14R2 1.261 0.235 5.357 0.000

SR14R3 1.261 0.235 5.357 0.000

SR12 0.000 0.000 999.000 999.000

SR13 0.000 0.000 999.000 999.000

SR14 0.000 0.000 999.000 999.000

DFIM1 0.000 0.000 999.000 999.000

DFIM2 0.000 0.000 999.000 999.000

DFIM3 0.000 0.000 999.000 999.000

SD 0.790 0.098 8.093 0.000

SC 0.232 0.060 3.891 0.000

IDFIM 0.000 0.000 999.000 999.000

SDFIM -0.455 0.075 -6.090 0.000

ID 0.000 0.000 999.000 999.000

IC 0.462 0.266 1.737 0.082

Thresholds

MARD13$1 5.179 0.482 10.751 0.000

MARD14$1 5.179 0.482 10.751 0.000

MARD15$1 5.179 0.482 10.751 0.000

MARD16$1 5.179 0.482 10.751 0.000

MARD17$1 5.179 0.482 10.751 0.000

MARD18$1 5.179 0.482 10.751 0.000

MARD19$1 5.179 0.482 10.751 0.000

MARD20$1 5.179 0.482 10.751 0.000

Variances

IC12 0.265 0.049 5.402 0.000

IC13 0.230 0.050 4.582 0.000

IC14 0.203 0.046 4.395 0.000

Residual Variances

MARC13 0.421 0.379 1.111 0.267

MARC14 0.408 0.176 2.321 0.020

MARC15 1.058 0.283 3.743 0.000

MARC16 1.135 0.270 4.211 0.000

MARC17 1.540 0.238 6.477 0.000

MARC18 1.162 0.236 4.918 0.000

MARC19 0.931 0.216 4.315 0.000

MARC20 1.227 0.298 4.122 0.000

IC12R1 0.715 0.067 10.724 0.000

IC12R2 0.449 0.056 8.051 0.000

IC12R3 0.606 0.079 7.674 0.000

IC13R1 0.312 0.035 9.004 0.000

IC13R2 0.449 0.056 8.051 0.000

IC13R3 0.317 0.039 8.061 0.000

IC14R1 0.312 0.035 9.004 0.000

IC14R2 0.171 0.040 4.312 0.000

IC14R3 0.317 0.039 8.061 0.000

SR12R1 0.311 0.046 6.832 0.000

SR12R2 0.193 0.020 9.480 0.000

SR12R3 0.247 0.037 6.734 0.000

SR13R1 0.131 0.017 7.482 0.000

SR13R2 0.193 0.020 9.480 0.000

SR13R3 0.201 0.027 7.500 0.000

SR14R1 0.131 0.017 7.482 0.000

SR14R2 0.193 0.020 9.480 0.000

SR14R3 0.201 0.027 7.500 0.000

SR12 0.000 0.000 999.000 999.000

SR13 0.000 0.000 999.000 999.000

SR14 0.000 0.000 999.000 999.000

DFIM1 0.312 0.063 4.935 0.000

DFIM2 0.312 0.063 4.935 0.000

DFIM3 0.312 0.063 4.935 0.000

SD 0.350 0.078 4.490 0.000

SC 0.081 0.015 5.319 0.000

IDFIM 0.238 0.044 5.401 0.000

SDFIM 0.181 0.051 3.531 0.000

ID 6.775 1.615 4.195 0.000

IC 0.822 0.347 2.367 0.018

STANDARDIZED MODEL RESULTS

STDYX Standardization

Two-Tailed

Estimate S.E. Est./S.E. P-Value

ID |

MARD13 0.821 0.032 25.599 0.000

MARD14 0.874 0.046 18.967 0.000

MARD15 0.898 0.064 14.076 0.000

MARD16 0.886 0.079 11.185 0.000

MARD17 0.842 0.088 9.535 0.000

MARD18 0.726 0.092 7.863 0.000

MARD19 0.707 0.088 8.068 0.000

MARD20 0.713 0.088 8.083 0.000

SD |

MARD13 0.000 0.000 999.000 999.000

MARD14 0.200 0.021 9.501 0.000

MARD15 0.410 0.044 9.369 0.000

MARD16 0.607 0.063 9.663 0.000

MARD17 0.769 0.074 10.348 0.000

MARD18 0.950 0.076 12.483 0.000

MARD19 0.969 0.077 12.602 0.000

MARD20 0.964 0.077 12.503 0.000

IC |

MARC13 0.818 0.111 7.373 0.000

MARC14 0.806 0.102 7.921 0.000

MARC15 0.621 0.108 5.770 0.000

MARC16 0.577 0.104 5.560 0.000

MARC17 0.475 0.091 5.238 0.000

MARC18 0.431 0.088 4.894 0.000

MARC19 0.411 0.086 4.798 0.000

MARC20 0.388 0.082 4.745 0.000

SC |

MARC13 0.000 0.000 999.000 999.000

MARC14 0.274 0.048 5.702 0.000

MARC15 0.422 0.051 8.316 0.000

MARC16 0.520 0.068 7.653 0.000

MARC17 0.645 0.058 11.178 0.000

MARC18 0.785 0.061 12.826 0.000

MARC19 0.837 0.059 14.235 0.000

MARC20 0.827 0.055 15.160 0.000

IDFIM |

DFIM1 0.667 0.054 12.393 0.000

DFIM2 0.796 0.101 7.890 0.000

DFIM3 0.797 0.102 7.777 0.000

SDFIM |

DFIM1 0.000 0.000 999.000 999.000

DFIM2 0.602 0.111 5.424 0.000

DFIM3 0.678 0.120 5.651 0.000

SR12 BY

SR12R1 0.732 0.029 25.189 0.000

SR12R2 0.810 0.028 29.012 0.000

SR12R3 0.752 0.034 22.297 0.000

SR13 BY

SR13R1 0.736 0.035 20.951 0.000

SR13R2 0.672 0.032 20.867 0.000

SR13R3 0.626 0.044 14.191 0.000

SR14 BY

SR14R1 0.702 0.039 18.189 0.000

SR14R2 0.636 0.036 17.416 0.000

SR14R3 0.589 0.044 13.231 0.000

IC12 BY

IC12R1 0.520 0.041 12.692 0.000

IC12R2 0.716 0.036 19.623 0.000

IC12R3 0.671 0.033 20.236 0.000

IC13 BY

IC13R1 0.651 0.055 11.879 0.000

IC13R2 0.690 0.036 19.307 0.000

IC13R3 0.759 0.031 24.820 0.000

IC14 BY

IC14R1 0.627 0.057 10.913 0.000

IC14R2 0.824 0.038 21.718 0.000

IC14R3 0.739 0.031 23.784 0.000

DFIM1 BY

SR12 1.254 0.084 15.003 0.000

DFIM2 BY

SR13 1.598 0.112 14.329 0.000

DFIM3 BY

SR14 1.761 0.151 11.647 0.000

SR12 ON

IC12 0.860 0.100 8.597 0.000

SR13 ON

IC13 1.218 0.130 9.381 0.000

SR14 ON

IC14 1.263 0.157 8.037 0.000

ID ON

GENDER 0.053 0.087 0.608 0.543

SD ON

GENDER 0.107 0.086 1.245 0.213

IC ON

GENDER -0.189 0.149 -1.267 0.205

SC ON

GENDER 0.421 0.092 4.565 0.000

IDFIM ON

GENDER 0.226 0.066 3.434 0.001

SDFIM ON

GENDER -0.050 0.086 -0.579 0.563

IC12 WITH

DFIM1 -0.816 0.051 -15.936 0.000

IC13 0.106 0.044 2.414 0.016

IC13 WITH

DFIM2 -0.878 0.029 -30.136 0.000

IC14 WITH

DFIM3 -0.933 0.032 -29.310 0.000

IDFIM WITH

SDFIM -0.832 0.046 -18.206 0.000

ID -0.032 0.133 -0.240 0.810

SD 0.198 0.137 1.443 0.149

IC 0.070 0.205 0.339 0.734

SC -0.024 0.156 -0.153 0.879

ID WITH

SD -0.506 0.089 -5.717 0.000

IC 0.841 0.110 7.652 0.000

SC 0.000 0.000 999.000 999.000

SDFIM -0.048 0.140 -0.346 0.729

IC WITH

SC 0.000 0.000 999.000 999.000

SD -0.340 0.203 -1.677 0.094

SDFIM -0.226 0.342 -0.660 0.509

SD WITH

SC 0.613 0.118 5.204 0.000

SDFIM -0.075 0.145 -0.515 0.606

SC WITH

SDFIM 0.192 0.233 0.823 0.410

SR13R3 WITH

SR13R1 -0.279 0.125 -2.241 0.025

SR13R2 0.326 0.095 3.429 0.001

IC14R3 WITH

IC12R1 0.288 0.079 3.668 0.000

SR14R3 WITH

SR14R2 0.394 0.074 5.311 0.000

IC14R1 WITH

IC12R1 0.177 0.068 2.582 0.010

IC13R3 WITH

IC13R1 -0.314 0.148 -2.124 0.034

IC12R2 0.291 0.114 2.558 0.011

IC12R3 0.225 0.092 2.439 0.015

SR14R2 WITH

SR13R1 -0.175 0.084 -2.081 0.037

Means

IC12 -1.448 0.354 -4.087 0.000

IC13 -0.905 0.358 -2.529 0.011

IC14 -0.760 0.384 -1.980 0.048

Intercepts

MARC13 0.000 0.000 999.000 999.000

MARC14 0.000 0.000 999.000 999.000

MARC15 0.000 0.000 999.000 999.000

MARC16 0.000 0.000 999.000 999.000

MARC17 0.000 0.000 999.000 999.000

MARC18 0.000 0.000 999.000 999.000

MARC19 0.000 0.000 999.000 999.000

MARC20 0.000 0.000 999.000 999.000

IC12R1 0.758 0.209 3.628 0.000

IC12R2 0.783 0.228 3.441 0.001

IC12R3 0.715 0.208 3.431 0.001

IC13R1 1.020 0.277 3.684 0.000

IC13R2 0.811 0.229 3.540 0.000

IC13R3 0.868 0.255 3.409 0.001

IC14R1 1.046 0.285 3.664 0.000

IC14R2 1.030 0.293 3.519 0.000

IC14R3 0.899 0.262 3.436 0.001

SR12R1 1.541 0.313 4.931 0.000

SR12R2 1.683 0.337 4.986 0.000

SR12R3 1.671 0.308 5.434 0.000

SR13R1 2.361 0.440 5.371 0.000

SR13R2 2.124 0.381 5.573 0.000

SR13R3 2.192 0.405 5.408 0.000

SR14R1 2.483 0.475 5.229 0.000

SR14R2 2.215 0.404 5.479 0.000

SR14R3 2.272 0.432 5.261 0.000

SR12 0.000 0.000 999.000 999.000

SR13 0.000 0.000 999.000 999.000

SR14 0.000 0.000 999.000 999.000

DFIM1 0.000 0.000 999.000 999.000

DFIM2 0.000 0.000 999.000 999.000

DFIM3 0.000 0.000 999.000 999.000

SD 1.327 0.186 7.150 0.000

SC 0.741 0.236 3.143 0.002

IDFIM 0.000 0.000 999.000 999.000

SDFIM -1.068 0.231 -4.620 0.000

ID 0.000 0.000 999.000 999.000

IC 0.501 0.346 1.448 0.148

Thresholds

MARD13$1 1.631 0.094 17.412 0.000

MARD14$1 1.736 0.099 17.565 0.000

MARD15$1 1.785 0.111 16.039 0.000

MARD16$1 1.761 0.128 13.801 0.000

MARD17$1 1.673 0.141 11.904 0.000

MARD18$1 1.442 0.153 9.440 0.000

MARD19$1 1.405 0.144 9.762 0.000

MARD20$1 1.416 0.147 9.646 0.000

Variances

IC12 1.000 0.000 999.000 999.000

IC13 1.000 0.000 999.000 999.000

IC14 1.000 0.000 999.000 999.000

Residual Variances

MARC13 0.331 0.182 1.821 0.069

MARC14 0.311 0.146 2.132 0.033

MARC15 0.478 0.111 4.306 0.000

MARC16 0.444 0.080 5.573 0.000

MARC17 0.407 0.051 7.935 0.000

MARC18 0.253 0.052 4.834 0.000

MARC19 0.184 0.045 4.133 0.000

MARC20 0.217 0.050 4.346 0.000

IC12R1 0.730 0.043 17.117 0.000

IC12R2 0.488 0.052 9.338 0.000

IC12R3 0.549 0.045 12.322 0.000

IC13R1 0.576 0.071 8.085 0.000

IC13R2 0.524 0.049 10.615 0.000

IC13R3 0.424 0.046 9.139 0.000

IC14R1 0.606 0.072 8.402 0.000

IC14R2 0.321 0.062 5.141 0.000

IC14R3 0.454 0.046 9.907 0.000

SR12R1 0.465 0.042 10.937 0.000

SR12R2 0.344 0.045 7.606 0.000

SR12R3 0.434 0.051 8.543 0.000

SR13R1 0.458 0.052 8.847 0.000

SR13R2 0.548 0.043 12.668 0.000

SR13R3 0.608 0.055 11.012 0.000

SR14R1 0.507 0.054 9.338 0.000

SR14R2 0.596 0.046 12.854 0.000

SR14R3 0.654 0.052 12.481 0.000

SR12 0.000 999.000 999.000 999.000

SR13 0.000 999.000 999.000 999.000

SR14 0.000 999.000 999.000 999.000

DFIM1 0.555 0.072 7.727 0.000

DFIM2 0.791 0.045 17.587 0.000

DFIM3 0.792 0.046 17.168 0.000

SD 0.989 0.018 53.789 0.000

SC 0.823 0.078 10.589 0.000

IDFIM 0.949 0.030 32.001 0.000

SDFIM 0.998 0.009 116.113 0.000

ID 0.997 0.009 107.293 0.000

IC 0.964 0.057 17.059 0.000

R-SQUARE

Observed Two-Tailed

Variable Estimate S.E. Est./S.E. P-Value

MARD13 0.674 0.053 12.799 0.000

MARD14 0.630 0.052 12.128 0.000

MARD15 0.609 0.047 13.069 0.000

MARD16 0.620 0.040 15.409 0.000

MARD17 0.657 0.037 17.921 0.000

MARD18 0.745 0.035 21.378 0.000

MARD19 0.758 0.033 23.105 0.000

MARD20 0.754 0.035 21.729 0.000

MARC13 0.669 0.182 3.686 0.000

MARC14 0.689 0.146 4.727 0.000

MARC15 0.522 0.111 4.694 0.000

MARC16 0.556 0.080 6.983 0.000

MARC17 0.593 0.051 11.550 0.000

MARC18 0.747 0.052 14.282 0.000

MARC19 0.816 0.045 18.266 0.000

MARC20 0.783 0.050 15.686 0.000

IC12R1 0.270 0.043 6.346 0.000

IC12R2 0.512 0.052 9.811 0.000

IC12R3 0.451 0.045 10.118 0.000

IC13R1 0.424 0.071 5.940 0.000

IC13R2 0.476 0.049 9.654 0.000

IC13R3 0.576 0.046 12.410 0.000

IC14R1 0.394 0.072 5.456 0.000

IC14R2 0.679 0.062 10.859 0.000

IC14R3 0.546 0.046 11.892 0.000

SR12R1 0.535 0.042 12.594 0.000

SR12R2 0.656 0.045 14.506 0.000

SR12R3 0.566 0.051 11.149 0.000

SR13R1 0.542 0.052 10.476 0.000

SR13R2 0.452 0.043 10.434 0.000

SR13R3 0.392 0.055 7.096 0.000

SR14R1 0.493 0.054 9.095 0.000

SR14R2 0.404 0.046 8.708 0.000

SR14R3 0.346 0.052 6.615 0.000

Latent Two-Tailed

Variable Estimate S.E. Est./S.E. P-Value

SR12 1.000 999.000 999.000 999.000

SR13 1.000 999.000 999.000 999.000

SR14 1.000 999.000 999.000 999.000

DFIM1 0.445 0.072 6.196 0.000

DFIM2 0.209 0.045 4.657 0.000

DFIM3 0.208 0.046 4.501 0.000

SD 0.011 0.018 0.622 0.534

SC 0.177 0.078 2.283 0.022

IDFIM 0.051 0.030 1.717 0.086

SDFIM 0.002 0.009 0.289 0.772

ID 0.003 0.009 0.304 0.761

IC 0.036 0.057 0.633 0.527

QUALITY OF NUMERICAL RESULTS

Condition Number for the Information Matrix 0.476E-05

(ratio of smallest to largest eigenvalue)

MODEL COMMAND WITH FINAL ESTIMATES USED AS STARTING VALUES

idfim sdfim | dfim1@0 dfim2* dfim3@1;

id sd | mard13@0 mard14@1 mard15@2 mard16@3 mard17@4 mard18*

mard19@6 mard20*;

ic sc | marc13@0 marc14@1 marc15@2 marc16* marc17@4

marc18* marc19@6 marc20*;

sr12 BY sr12r1@1;

sr12 BY sr12r2*1.01424 (sr2);

sr12 BY sr12r3*0.94877;

sr13 BY sr13r1@1;

sr13 BY sr13r2*1.01424 (sr2);

sr13 BY sr13r3*0.91582 (sr3);

sr14 BY sr14r1@1;

sr14 BY sr14r2*1.01424 (sr2);

sr14 BY sr14r3*0.91582 (sr3);

ic12 BY ic12r1@1;

ic12 BY ic12r2*1.33320 (ic2);

ic12 BY ic12r3*1.37006 (ic3);

ic13 BY ic13r1@1;

ic13 BY ic13r2*1.33320 (ic2);

ic13 BY ic13r3*1.37006 (ic3);

ic14 BY ic14r1@1;

ic14 BY ic14r2*1.33320 (ic2);

ic14 BY ic14r3*1.37006 (ic3);

sd BY mard18*5.73372;

sd BY mard20*5.92395;

sc BY marc16*2.65336;

sc BY marc18*5.36370;

sc BY marc20*6.27038;

dfim1 BY sr12@1;

dfim2 BY sr13@1;

dfim3 BY sr14@1;

sdfim BY dfim2*0.88882;

id ON gender*0.27860;

sd ON gender*0.12804;

ic ON gender*-0.35142;

sc ON gender*0.26563;

idfim ON gender*0.22724;

sdfim ON gender*-0.04273;

sr12 ON ic12@1;

sr13 ON ic13@1;

sr14 ON ic14@1;

sr13r3 WITH sr13r1*-0.04528;

sr13r3 WITH sr13r2*0.06431;

ic14r3 WITH ic12r1*0.13736;

sr14r3 WITH sr14r2*0.07765;

ic14r1 WITH ic12r1*0.08352;

ic13r3 WITH ic13r1*-0.09877;

ic13r3 WITH ic12r2*0.10984;

ic13r3 WITH ic12r3*0.09890;

sr14r2 WITH sr13r1*-0.02779;

ic12 WITH dfim1*-0.23505 (icwdfim);

ic12 WITH ic13*0.02613;

ic13 WITH dfim2*-0.23505 (icwdfim);

ic14 WITH dfim3*-0.23505 (icwdfim);

idfim WITH sdfim*-0.17269;

idfim WITH id*-0.04044;

idfim WITH sd*0.05725;

idfim WITH ic*0.03078;

idfim WITH sc*-0.00331;

id WITH sd*-0.77938;

id WITH ic*1.98567;

id WITH sc@0;

id WITH sdfim*-0.05355;

ic WITH sc@0;

ic WITH sd*-0.18247;

ic WITH sdfim*-0.08717;

sd WITH sc*0.10321;

sd WITH sdfim*-0.01886;

sc WITH sdfim*0.02318;

[ marc13@0 ];

[ marc14@0 ];

[ marc15@0 ];

[ marc16@0 ];

[ marc17@0 ];

[ marc18@0 ];

[ marc19@0 ];

[ marc20@0 ];

[ ic12r1*0.75096 ] (ici1);

[ ic12r2*0.75096 ] (ici1);

[ ic12r3*0.75096 ] (ici1);

[ ic13r1*0.75096 ] (ici1);

[ ic13r2*0.75096 ] (ici1);

[ ic13r3*0.75096 ] (ici1);

[ ic14r1*0.75096 ] (ici1);

[ ic14r2*0.75096 ] (ici1);

[ ic14r3*0.75096 ] (ici1);

[ sr12r1*1.26108 ] (sri1);

[ sr12r2*1.26108 ] (sri1);

[ sr12r3*1.26108 ] (sri1);

[ sr13r1*1.26108 ] (sri1);

[ sr13r2*1.26108 ] (sri1);

[ sr13r3*1.26108 ] (sri1);

[ sr14r1*1.26108 ] (sri1);

[ sr14r2*1.26108 ] (sri1);

[ sr14r3*1.26108 ] (sri1);

[ sr12@0 ];

[ sr13@0 ];

[ sr14@0 ];

[ ic12*-0.74598 ];

[ ic13*-0.43355 ];

[ ic14*-0.34231 ];

[ dfim1@0 ];

[ dfim2@0 ];

[ dfim3@0 ];

[ sd*0.78986 ];

[ sc*0.23243 ];

[ idfim@0 ];

[ sdfim*-0.45489 ];

[ id@0 ];

[ ic*0.46222 ];

[ mard13$1*5.17870 ] (80);

[ mard14$1*5.17870 ] (80);

[ mard15$1*5.17870 ] (80);

[ mard16$1*5.17870 ] (80);

[ mard17$1*5.17870 ] (80);

[ mard18$1*5.17870 ] (80);

[ mard19$1*5.17870 ] (80);

[ mard20$1*5.17870 ] (80);

marc13*0.42124;

marc14*0.40810;

marc15*1.05834;

marc16*1.13511;

marc17*1.53988;

marc18*1.16242;

marc19*0.93112;

marc20*1.22704;

ic12r1*0.71539;

ic12r2*0.44868 (icr2);

ic12r3*0.60629;

ic13r1*0.31250 (icr1);

ic13r2*0.44868 (icr2);

ic13r3*0.31732 (icr3);

ic14r1*0.31250 (icr1);

ic14r2*0.17077;

ic14r3*0.31732 (icr3);

sr12r1*0.31107;

sr12r2*0.19322 (srr2);

sr12r3*0.24709;

sr13r1*0.13065 (srr1);

sr13r2*0.19322 (srr2);

sr13r3*0.20135 (srr3);

sr14r1*0.13065 (srr1);

sr14r2*0.19322 (srr2);

sr14r3*0.20135 (srr3);

sr12@0;

sr13@0;

sr14@0;

ic12*0.26523;

ic13*0.22956;

ic14*0.20293;

dfim1*0.31249 (dfimres);

dfim2*0.31249 (dfimres);

dfim3*0.31249 (dfimres);

sd*0.35010;

sc*0.08089;

idfim*0.23782;

sdfim*0.18108;

id*6.77491;

ic*0.82213;

RESIDUAL OUTPUT

ESTIMATED MODEL AND RESIDUALS (OBSERVED - ESTIMATED)

Model Estimated Means

MARC13 MARC14 MARC15 MARC16 MARC17

________ ________ ________ ________ ________

0.305 0.656 1.007 1.237 1.709

Model Estimated Means

MARC18 MARC19 MARC20 IC12R1 IC12R2

________ ________ ________ ________ ________

2.188 2.411 2.506 0.005 -0.244

Model Estimated Means

IC12R3 IC13R1 IC13R2 IC13R3 IC14R1

________ ________ ________ ________ ________

-0.271 0.317 0.173 0.157 0.409

Model Estimated Means

IC14R2 IC14R3 SR12R1 SR12R2 SR12R3

________ ________ ________ ________ ________

0.295 0.282 0.617 0.607 0.650

Model Estimated Means

SR13R1 SR13R2 SR13R3 SR14R1 SR14R2

________ ________ ________ ________ ________

0.508 0.497 0.571 0.546 0.536

Model Estimated Means

SR14R3

________

0.606

Residuals for Means

MARC13 MARC14 MARC15 MARC16 MARC17

________ ________ ________ ________ ________

1.228 1.087 1.312 1.272 1.083

Residuals for Means

MARC18 MARC19 MARC20 IC12R1 IC12R2

________ ________ ________ ________ ________

1.032 0.972 1.099 -0.036 -0.045

Residuals for Means

IC12R3 IC13R1 IC13R2 IC13R3 IC14R1

________ ________ ________ ________ ________

0.054 0.008 0.011 -0.040 -0.039

Residuals for Means

IC14R2 IC14R3 SR12R1 SR12R2 SR12R3

________ ________ ________ ________ ________

0.013 -0.067 0.053 -0.033 -0.026

Residuals for Means

SR13R1 SR13R2 SR13R3 SR14R1 SR14R2

________ ________ ________ ________ ________

-0.026 0.009 0.026 -0.020 0.038

Residuals for Means

SR14R3

________

0.017

Model Estimated Covariances

MARC13 MARC14 MARC15 MARC16 MARC17

________ ________ ________ ________ ________

MARC13 1.274

MARC14 0.830 1.313

MARC15 0.807 0.980 2.212

MARC16 0.791 1.029 1.267 2.558

MARC17 0.760 1.131 1.501 1.743 3.781

MARC18 0.729 1.233 1.738 2.067 2.746

MARC19 0.714 1.281 1.848 2.218 2.982

MARC20 0.708 1.301 1.895 2.283 3.082

IC12R1 0.000 0.000 0.000 0.000 0.000

IC12R2 0.000 0.000 0.000 0.000 0.000

IC12R3 0.000 0.000 0.000 0.000 0.000

IC13R1 0.000 0.000 0.000 0.000 0.000

IC13R2 0.000 0.000 0.000 0.000 0.000

IC13R3 0.000 0.000 0.000 0.000 0.000

IC14R1 0.000 0.000 0.000 0.000 0.000

IC14R2 0.000 0.000 0.000 0.000 0.000

IC14R3 0.000 0.000 0.000 0.000 0.000

SR12R1 0.011 0.023 0.034 0.042 0.057

SR12R2 0.011 0.023 0.035 0.042 0.058

SR12R3 0.010 0.021 0.033 0.040 0.055

SR13R1 -0.063 -0.033 -0.004 0.016 0.056

SR13R2 -0.064 -0.034 -0.004 0.016 0.057

SR13R3 -0.058 -0.031 -0.003 0.014 0.051

SR14R1 -0.072 -0.040 -0.008 0.012 0.056

SR14R2 -0.073 -0.041 -0.009 0.013 0.056

SR14R3 -0.066 -0.037 -0.008 0.011 0.051

Model Estimated Covariances

MARC18 MARC19 MARC20 IC12R1 IC12R2

________ ________ ________ ________ ________

MARC18 4.596

MARC19 3.755 5.047

MARC20 3.891 4.269 5.656

IC12R1 0.000 0.000 0.000 0.981

IC12R2 0.000 0.000 0.000 0.354 0.920

IC12R3 0.000 0.000 0.000 0.363 0.484

IC13R1 0.000 0.000 0.000 0.026 0.035

IC13R2 0.000 0.000 0.000 0.035 0.046

IC13R3 0.000 0.000 0.000 0.036 0.158

IC14R1 0.000 0.000 0.000 0.084 0.000

IC14R2 0.000 0.000 0.000 0.000 0.000

IC14R3 0.000 0.000 0.000 0.137 0.000

SR12R1 0.073 0.081 0.084 0.030 0.040

SR12R2 0.074 0.082 0.085 0.031 0.041

SR12R3 0.070 0.077 0.080 0.029 0.038

SR13R1 0.096 0.115 0.123 0.026 0.035

SR13R2 0.098 0.117 0.125 0.027 0.035

SR13R3 0.088 0.106 0.113 0.024 0.032

SR14R1 0.099 0.120 0.128 0.000 0.000

SR14R2 0.101 0.121 0.130 0.000 0.000

SR14R3 0.091 0.109 0.117 0.000 0.000

Model Estimated Covariances

IC12R3 IC13R1 IC13R2 IC13R3 IC14R1

________ ________ ________ ________ ________

IC12R3 1.104

IC13R1 0.036 0.542

IC13R2 0.048 0.306 0.857

IC13R3 0.148 0.216 0.419 0.748

IC14R1 0.000 0.000 0.000 0.000 0.515

IC14R2 0.000 0.000 0.000 0.000 0.271

IC14R3 0.000 0.000 0.000 0.000 0.278

SR12R1 0.041 0.026 0.035 0.036 0.000

SR12R2 0.042 0.027 0.035 0.036 0.000

SR12R3 0.039 0.025 0.033 0.034 0.000

SR13R1 0.036 -0.005 -0.007 -0.008 0.000

SR13R2 0.036 -0.006 -0.007 -0.008 0.000

SR13R3 0.033 -0.005 -0.007 -0.007 0.000

SR14R1 0.000 0.000 0.000 0.000 -0.032

SR14R2 0.000 0.000 0.000 0.000 -0.033

SR14R3 0.000 0.000 0.000 0.000 -0.029

Model Estimated Covariances

IC14R2 IC14R3 SR12R1 SR12R2 SR12R3

________ ________ ________ ________ ________

IC14R2 0.531

IC14R3 0.371 0.698

SR12R1 0.000 0.000 0.669

SR12R2 0.000 0.000 0.363 0.562

SR12R3 0.000 0.000 0.340 0.345 0.570

SR13R1 0.000 0.000 0.121 0.123 0.115

SR13R2 0.000 0.000 0.123 0.125 0.117

SR13R3 0.000 0.000 0.111 0.112 0.105

SR14R1 -0.043 -0.044 0.075 0.077 0.072

SR14R2 -0.043 -0.045 0.077 0.078 0.073

SR14R3 -0.039 -0.040 0.069 0.070 0.066

Model Estimated Covariances

SR13R1 SR13R2 SR13R3 SR14R1 SR14R2

________ ________ ________ ________ ________

SR13R1 0.285

SR13R2 0.157 0.352

SR13R3 0.096 0.208 0.331

SR14R1 0.081 0.082 0.074 0.258

SR14R2 0.055 0.084 0.075 0.129 0.324

SR14R3 0.074 0.075 0.068 0.117 0.196

Model Estimated Covariances

SR14R3

________

SR14R3 0.308

Residuals for Covariances

MARC13 MARC14 MARC15 MARC16 MARC17

________ ________ ________ ________ ________

MARC13 -0.976

MARC14 -0.028 -0.485

MARC15 -1.275 0.590 -0.116

MARC16 -0.499 1.311 3.572 -0.641

MARC17 4.082 2.432 2.566 2.285 -0.453

MARC18 1.324 1.606 1.739 1.692 2.910

MARC19 0.509 2.853 2.149 1.617 0.848

MARC20 0.928 -0.424 2.899 1.529 0.079

IC12R1 -0.413 -0.113 0.070 -0.384 -0.200

IC12R2 -0.530 0.274 0.289 0.151 0.435

IC12R3 -0.030 0.572 0.196 0.096 -0.041

IC13R1 -0.080 0.404 0.097 0.136 -0.003

IC13R2 -0.094 0.311 0.012 -0.445 -0.449

IC13R3 0.131 0.454 -0.261 -0.354 0.060

IC14R1 0.050 0.061 0.221 0.141 -0.278

IC14R2 -0.090 -0.235 0.302 0.208 -0.005

IC14R3 0.545 0.044 0.010 -0.335 -0.327

SR12R1 -0.209 0.005 -0.135 0.301 0.805

SR12R2 -0.625 -0.146 -0.231 0.031 0.367

SR12R3 -0.748 0.086 0.217 -0.380 -0.121

SR13R1 -0.096 0.307 -0.317 -0.074 -0.050

SR13R2 -0.471 -0.173 -0.626 -0.473 -0.115

SR13R3 -0.299 0.097 -0.567 -0.266 0.100

SR14R1 -0.119 -0.039 -0.207 -0.094 -0.403

SR14R2 0.240 0.056 -0.217 0.092 -0.277

SR14R3 0.042 -0.010 -0.081 0.151 -0.257

Residuals for Covariances

MARC18 MARC19 MARC20 IC12R1 IC12R2

________ ________ ________ ________ ________

MARC18 -0.810

MARC19 0.760 -0.944

MARC20 0.394 0.532 -1.687

IC12R1 0.034 0.142 -0.363 0.094

IC12R2 0.156 0.333 -0.321 0.157 0.197

IC12R3 0.257 -0.027 -0.387 0.100 0.128

IC13R1 0.141 -0.040 -0.175 0.098 0.142

IC13R2 -0.350 0.065 0.023 0.094 0.119

IC13R3 -0.237 0.333 0.356 0.140 0.140

IC14R1 0.051 0.024 -0.075 0.077 0.152

IC14R2 -0.029 0.159 0.083 0.081 0.157

IC14R3 0.126 0.056 0.229 0.092 0.117

SR12R1 -0.095 -0.211 -0.021 -0.080 -0.130

SR12R2 0.128 -0.007 0.076 -0.088 -0.065

SR12R3 -0.141 -0.199 -0.175 -0.085 -0.050

SR13R1 -0.197 -0.039 -0.140 -0.028 -0.012

SR13R2 -0.272 -0.068 -0.199 -0.033 -0.081

SR13R3 -0.215 -0.022 -0.130 -0.055 -0.094

SR14R1 -0.120 -0.009 -0.059 -0.100 -0.082

SR14R2 0.222 -0.091 0.032 -0.062 -0.093

SR14R3 0.265 -0.049 0.075 -0.064 -0.095

Residuals for Covariances

IC12R3 IC13R1 IC13R2 IC13R3 IC14R1

________ ________ ________ ________ ________

IC12R3 0.113

IC13R1 0.186 -0.054

IC13R2 0.194 -0.102 -0.121

IC13R3 0.126 -0.031 -0.061 0.019

IC14R1 0.179 0.091 0.187 0.119 0.011

IC14R2 0.101 0.159 0.193 0.198 -0.053

IC14R3 0.146 0.092 0.214 0.186 -0.059

SR12R1 -0.115 -0.068 -0.072 -0.095 -0.124

SR12R2 -0.116 -0.042 -0.081 -0.112 -0.152

SR12R3 -0.077 -0.041 -0.066 -0.110 -0.103

SR13R1 -0.011 0.004 0.007 0.006 -0.005

SR13R2 -0.063 -0.021 0.010 -0.025 -0.050

SR13R3 -0.106 -0.068 -0.015 -0.059 -0.031

SR14R1 -0.115 -0.046 -0.023 -0.038 -0.011

SR14R2 -0.117 -0.009 -0.038 0.018 0.033

SR14R3 -0.109 -0.014 -0.012 -0.020 0.025

Residuals for Covariances

IC14R2 IC14R3 SR12R1 SR12R2 SR12R3

________ ________ ________ ________ ________

IC14R2 -0.079

IC14R3 -0.120 -0.112

SR12R1 -0.065 -0.074 -0.043

SR12R2 -0.094 -0.102 -0.011 0.011

SR12R3 -0.030 -0.061 -0.042 -0.020 -0.037

SR13R1 -0.026 0.014 -0.017 -0.009 -0.025

SR13R2 -0.072 0.018 -0.036 0.000 -0.026

SR13R3 -0.087 -0.005 -0.022 0.027 0.013

SR14R1 0.021 -0.003 -0.048 -0.081 -0.036

SR14R2 0.004 0.043 -0.056 -0.055 -0.061

SR14R3 -0.004 0.017 -0.050 -0.053 -0.067

Residuals for Covariances

SR13R1 SR13R2 SR13R3 SR14R1 SR14R2

________ ________ ________ ________ ________

SR13R1 0.007

SR13R2 -0.007 -0.017

SR13R3 0.006 0.005 0.024

SR14R1 -0.009 -0.014 0.029 -0.037

SR14R2 -0.001 -0.003 0.024 -0.006 0.000

SR14R3 0.018 0.026 0.070 -0.004 -0.002

Residuals for Covariances

SR14R3

________

SR14R3 -0.008

UNIVARIATE DISTRIBUTION FIT

Variable Observed Estimated Residual (Obs.-Est.) Stand. Residual

MARD13

Category 1 0.975 0.945 0.030 2.583

Category 2 0.025 0.055 -0.030 -2.583

MARD14

Category 1 0.908 0.922 -0.015 -1.064

Category 2 0.092 0.078 0.015 1.064

MARD15

Category 1 0.860 0.879 -0.020 -1.174

Category 2 0.140 0.121 0.020 1.174

MARD16

Category 1 0.817 0.807 0.010 0.519

Category 2 0.183 0.193 -0.010 -0.519

MARD17

Category 1 0.698 0.707 -0.009 -0.383

Category 2 0.302 0.293 0.009 0.383

MARD18

Category 1 0.520 0.522 -0.002 -0.075

Category 2 0.480 0.478 0.002 0.075

MARD19

Category 1 0.527 0.497 0.030 1.187

Category 2 0.473 0.503 -0.030 -1.187

MARD20

Category 1 0.500 0.504 -0.004 -0.159

Category 2 0.500 0.496 0.004 0.159

BIVARIATE DISTRIBUTIONS FIT

Variable Variable Observed Estimated Residual (Obs.-Est.) Stand. Residual

MARD13 MARD14

Category 1 Category 1 0.900 0.891 0.009 0.585

Category 1 Category 2 0.074 0.054 0.020 1.745

Category 2 Category 1 0.011 0.032 -0.020 -2.249

Category 2 Category 2 0.014 0.024 -0.009 -1.213

MARD13 MARD15

Category 1 Category 1 0.858 0.851 0.007 0.362

Category 1 Category 2 0.124 0.094 0.030 2.045

Category 2 Category 1 0.006 0.028 -0.022 -2.618

Category 2 Category 2 0.012 0.027 -0.015 -1.797

MARD13 MARD16

Category 1 Category 1 0.812 0.782 0.031 1.456

Category 1 Category 2 0.166 0.163 0.003 0.138

Category 2 Category 1 0.006 0.025 -0.019 -2.361

Category 2 Category 2 0.016 0.030 -0.014 -1.656

MARD13 MARD17

Category 1 Category 1 0.688 0.685 0.004 0.157

Category 1 Category 2 0.291 0.260 0.031 1.380

Category 2 Category 1 0.007 0.023 -0.016 -2.077

Category 2 Category 2 0.014 0.033 -0.019 -2.082

MARD13 MARD18

Category 1 Category 1 0.512 0.502 0.010 0.387

Category 1 Category 2 0.456 0.442 0.014 0.533

Category 2 Category 1 0.004 0.020 -0.016 -2.276

Category 2 Category 2 0.028 0.036 -0.007 -0.767

MARD13 MARD19

Category 1 Category 1 0.525 0.478 0.047 1.859

Category 1 Category 2 0.452 0.467 -0.016 -0.614

Category 2 Category 1 0.003 0.019 -0.016 -2.276

Category 2 Category 2 0.020 0.036 -0.016 -1.661

MARD13 MARD20

Category 1 Category 1 0.489 0.485 0.004 0.176

Category 1 Category 2 0.493 0.460 0.033 1.306

Category 2 Category 1 0.000 0.019 -0.019 -2.757

Category 2 Category 2 0.017 0.036 -0.018 -1.928

MARD14 MARD15

Category 1 Category 1 0.827 0.839 -0.012 -0.621

Category 1 Category 2 0.081 0.084 -0.003 -0.214

Category 2 Category 1 0.030 0.041 -0.011 -1.071

Category 2 Category 2 0.063 0.037 0.026 2.640

MARD14 MARD16

Category 1 Category 1 0.785 0.773 0.011 0.520

Category 1 Category 2 0.135 0.149 -0.013 -0.731

Category 2 Category 1 0.037 0.033 0.004 0.383

Category 2 Category 2 0.043 0.044 -0.001 -0.128

MARD14 MARD17

Category 1 Category 1 0.673 0.680 -0.006 -0.263

Category 1 Category 2 0.259 0.243 0.017 0.765

Category 2 Category 1 0.027 0.028 -0.001 -0.079

Category 2 Category 2 0.040 0.050 -0.010 -0.880

MARD14 MARD18

Category 1 Category 1 0.500 0.501 -0.001 -0.035

Category 1 Category 2 0.417 0.421 -0.005 -0.183

Category 2 Category 1 0.021 0.021 0.000 -0.060

Category 2 Category 2 0.062 0.057 0.006 0.504

MARD14 MARD19

Category 1 Category 1 0.503 0.476 0.027 1.054

Category 1 Category 2 0.422 0.446 -0.024 -0.954

Category 2 Category 1 0.026 0.021 0.006 0.760

Category 2 Category 2 0.049 0.057 -0.008 -0.689

MARD14 MARD20

Category 1 Category 1 0.464 0.483 -0.019 -0.761

Category 1 Category 2 0.434 0.439 -0.005 -0.192

Category 2 Category 1 0.038 0.021 0.017 2.398

Category 2 Category 2 0.064 0.057 0.007 0.575

MARD15 MARD16

Category 1 Category 1 0.769 0.754 0.015 0.683

Category 1 Category 2 0.106 0.125 -0.019 -1.138

Category 2 Category 1 0.051 0.053 -0.001 -0.123

Category 2 Category 2 0.074 0.068 0.006 0.436

MARD15 MARD17

Category 1 Category 1 0.647 0.668 -0.021 -0.887

Category 1 Category 2 0.233 0.211 0.022 1.055

Category 2 Category 1 0.039 0.039 0.000 -0.040

Category 2 Category 2 0.081 0.082 0.000 -0.018

MARD15 MARD18

Category 1 Category 1 0.489 0.497 -0.007 -0.293

Category 1 Category 2 0.380 0.383 -0.002 -0.087

Category 2 Category 1 0.018 0.026 -0.007 -0.923

Category 2 Category 2 0.112 0.095 0.017 1.139

MARD15 MARD19

Category 1 Category 1 0.490 0.473 0.017 0.668

Category 1 Category 2 0.381 0.406 -0.026 -1.016

Category 2 Category 1 0.034 0.024 0.010 1.241

Category 2 Category 2 0.095 0.097 -0.001 -0.086

MARD15 MARD20

Category 1 Category 1 0.451 0.479 -0.028 -1.102

Category 1 Category 2 0.407 0.400 0.007 0.292

Category 2 Category 1 0.049 0.025 0.024 3.039

Category 2 Category 2 0.093 0.096 -0.003 -0.216

MARD16 MARD17

Category 1 Category 1 0.659 0.643 0.017 0.686

Category 1 Category 2 0.176 0.164 0.011 0.604

Category 2 Category 1 0.039 0.065 -0.025 -2.002

Category 2 Category 2 0.125 0.129 -0.003 -0.181

MARD16 MARD18

Category 1 Category 1 0.489 0.487 0.001 0.056

Category 1 Category 2 0.326 0.319 0.006 0.272

Category 2 Category 1 0.030 0.035 -0.005 -0.545

Category 2 Category 2 0.156 0.158 -0.003 -0.151

MARD16 MARD19

Category 1 Category 1 0.502 0.465 0.037 1.444

Category 1 Category 2 0.311 0.342 -0.031 -1.260

Category 2 Category 1 0.035 0.032 0.003 0.280

Category 2 Category 2 0.152 0.161 -0.009 -0.467

MARD16 MARD20

Category 1 Category 1 0.450 0.471 -0.021 -0.816

Category 1 Category 2 0.356 0.336 0.020 0.838

Category 2 Category 1 0.050 0.033 0.017 1.841

Category 2 Category 2 0.144 0.160 -0.016 -0.862

MARD17 MARD18

Category 1 Category 1 0.478 0.470 0.008 0.305

Category 1 Category 2 0.220 0.237 -0.017 -0.785

Category 2 Category 1 0.065 0.052 0.013 1.134

Category 2 Category 2 0.237 0.240 -0.004 -0.165

MARD17 MARD19

Category 1 Category 1 0.445 0.450 -0.005 -0.182

Category 1 Category 2 0.245 0.257 -0.012 -0.540

Category 2 Category 1 0.087 0.047 0.040 3.667

Category 2 Category 2 0.223 0.246 -0.023 -1.046

MARD17 MARD20

Category 1 Category 1 0.408 0.456 -0.047 -1.853

Category 1 Category 2 0.314 0.252 0.062 2.814

Category 2 Category 1 0.089 0.049 0.040 3.686

Category 2 Category 2 0.188 0.244 -0.056 -2.539

MARD18 MARD19

Category 1 Category 1 0.438 0.398 0.039 1.559

Category 1 Category 2 0.082 0.124 -0.042 -2.476

Category 2 Category 1 0.094 0.099 -0.005 -0.313

Category 2 Category 2 0.387 0.379 0.007 0.299

MARD18 MARD20

Category 1 Category 1 0.353 0.402 -0.049 -1.942

Category 1 Category 2 0.136 0.120 0.016 0.939

Category 2 Category 1 0.125 0.102 0.023 1.477

Category 2 Category 2 0.386 0.376 0.010 0.412

MARD19 MARD20

Category 1 Category 1 0.380 0.391 -0.011 -0.450

Category 1 Category 2 0.106 0.106 0.000 -0.014

Category 2 Category 1 0.115 0.113 0.002 0.145

Category 2 Category 2 0.399 0.390 0.009 0.364

TECHNICAL 4 OUTPUT

ESTIMATES DERIVED FROM THE MODEL

ESTIMATED MEANS FOR THE LATENT VARIABLES

SR12 SR13 SR14 IC12 IC13

________ ________ ________ ________ ________

-0.645 -0.753 -0.715 -0.746 -0.434

ESTIMATED MEANS FOR THE LATENT VARIABLES

IC14 DFIM1 DFIM2 DFIM3 SD

________ ________ ________ ________ ________

-0.342 0.101 -0.320 -0.373 0.847

ESTIMATED MEANS FOR THE LATENT VARIABLES

SC IDFIM SDFIM ID IC

________ ________ ________ ________ ________

0.351 0.101 -0.474 0.124 0.305

ESTIMATED MEANS FOR THE LATENT VARIABLES

GENDER

________

0.446

S.E. FOR ESTIMATED MEANS FOR THE LATENT VARIABLES

SR12 SR13 SR14 IC12 IC13

________ ________ ________ ________ ________

0.232 0.236 0.238 0.234 0.206

S.E. FOR ESTIMATED MEANS FOR THE LATENT VARIABLES

IC14 DFIM1 DFIM2 DFIM3 SD

________ ________ ________ ________ ________

0.203 0.032 0.065 0.074 0.084

S.E. FOR ESTIMATED MEANS FOR THE LATENT VARIABLES

SC IDFIM SDFIM ID IC

________ ________ ________ ________ ________

0.055 0.032 0.074 0.207 0.248

S.E. FOR ESTIMATED MEANS FOR THE LATENT VARIABLES

GENDER

________

0.025

EST./S.E. FOR ESTIMATED MEANS FOR THE LATENT VARIABLES

SR12 SR13 SR14 IC12 IC13

________ ________ ________ ________ ________

-2.774 -3.194 -3.008 -3.193 -2.103

EST./S.E. FOR ESTIMATED MEANS FOR THE LATENT VARIABLES

IC14 DFIM1 DFIM2 DFIM3 SD

________ ________ ________ ________ ________

-1.689 3.137 -4.936 -5.014 10.110

EST./S.E. FOR ESTIMATED MEANS FOR THE LATENT VARIABLES

SC IDFIM SDFIM ID IC

________ ________ ________ ________ ________

6.417 3.137 -6.376 0.600 1.229

EST./S.E. FOR ESTIMATED MEANS FOR THE LATENT VARIABLES

GENDER

________

17.576

TWO-TAILED P-VALUE FOR ESTIMATED MEANS FOR THE LATENT VARIABLES

SR12 SR13 SR14 IC12 IC13

________ ________ ________ ________ ________

0.006 0.001 0.003 0.001 0.036

TWO-TAILED P-VALUE FOR ESTIMATED MEANS FOR THE LATENT VARIABLES

IC14 DFIM1 DFIM2 DFIM3 SD

________ ________ ________ ________ ________

0.091 0.002 0.000 0.000 0.000

TWO-TAILED P-VALUE FOR ESTIMATED MEANS FOR THE LATENT VARIABLES

SC IDFIM SDFIM ID IC

________ ________ ________ ________ ________

0.000 0.002 0.000 0.549 0.219

TWO-TAILED P-VALUE FOR ESTIMATED MEANS FOR THE LATENT VARIABLES

GENDER

________

0.000

ESTIMATED COVARIANCE MATRIX FOR THE LATENT VARIABLES

SR12 SR13 SR14 IC12 IC13

________ ________ ________ ________ ________

SR12 0.358

SR13 0.121 0.155

SR14 0.075 0.081 0.127

IC12 0.030 0.026 0.000 0.265

IC13 0.026 -0.005 0.000 0.026 0.230

IC14 0.000 0.000 -0.032 0.000 0.000

DFIM1 0.328 0.095 0.075 -0.235 0.000

DFIM2 0.095 0.160 0.081 0.000 -0.235

DFIM3 0.075 0.081 0.159 0.000 0.000

SD 0.064 0.046 0.044 0.000 0.000

SC 0.012 0.030 0.032 0.000 0.000

IDFIM 0.251 0.095 0.075 0.000 0.000

SDFIM -0.175 -0.014 0.006 0.000 0.000

ID -0.025 -0.075 -0.081 0.000 0.000

IC 0.011 -0.063 -0.072 0.000 0.000

GENDER 0.056 0.047 0.046 0.000 0.000

ESTIMATED COVARIANCE MATRIX FOR THE LATENT VARIABLES

IC14 DFIM1 DFIM2 DFIM3 SD

________ ________ ________ ________ ________

IC14 0.203

DFIM1 0.000 0.563

DFIM2 0.000 0.095 0.395

DFIM3 -0.235 0.075 0.081 0.394

SD 0.000 0.064 0.046 0.044 0.354

SC 0.000 0.012 0.030 0.032 0.112

IDFIM 0.000 0.251 0.095 0.075 0.064

SDFIM 0.000 -0.175 -0.014 0.006 -0.020

ID 0.000 -0.025 -0.075 -0.081 -0.771

IC 0.000 0.011 -0.063 -0.072 -0.194

GENDER 0.000 0.056 0.047 0.046 0.032

ESTIMATED COVARIANCE MATRIX FOR THE LATENT VARIABLES

SC IDFIM SDFIM ID IC

________ ________ ________ ________ ________

SC 0.098

IDFIM 0.012 0.251

SDFIM 0.020 -0.175 0.182

ID 0.018 -0.025 -0.056 6.794

IC -0.023 0.011 -0.083 1.961 0.853

GENDER 0.066 0.056 -0.011 0.069 -0.087

ESTIMATED COVARIANCE MATRIX FOR THE LATENT VARIABLES

GENDER

________

GENDER 0.247

S.E. FOR ESTIMATED COVARIANCE MATRIX FOR THE LATENT VARIABLES

SR12 SR13 SR14 IC12 IC13

________ ________ ________ ________ ________

SR12 0.053

SR13 0.026 0.021

SR14 0.024 0.016 0.019

IC12 0.022 0.010 0.000 0.049

IC13 0.010 0.012 0.000 0.010 0.050

IC14 0.000 0.000 0.014 0.000 0.000

DFIM1 0.050 0.028 0.024 0.052 0.000

DFIM2 0.028 0.025 0.016 0.000 0.052

DFIM3 0.024 0.016 0.024 0.000 0.000

SD 0.039 0.024 0.024 0.000 0.000

SC 0.023 0.016 0.018 0.000 0.000

IDFIM 0.047 0.028 0.024 0.000 0.000

SDFIM 0.043 0.025 0.022 0.000 0.000

ID 0.167 0.100 0.102 0.000 0.000

IC 0.098 0.082 0.094 0.000 0.000

GENDER 0.018 0.011 0.012 0.000 0.000

S.E. FOR ESTIMATED COVARIANCE MATRIX FOR THE LATENT VARIABLES

IC14 DFIM1 DFIM2 DFIM3 SD

________ ________ ________ ________ ________

IC14 0.046

DFIM1 0.000 0.074

DFIM2 0.000 0.028 0.067

DFIM3 0.052 0.024 0.016 0.066

SD 0.000 0.039 0.024 0.024 0.079

SC 0.000 0.023 0.016 0.018 0.028

IDFIM 0.000 0.047 0.028 0.024 0.039

SDFIM 0.000 0.043 0.025 0.022 0.036

ID 0.000 0.167 0.100 0.102 0.264

IC 0.000 0.098 0.082 0.094 0.105

GENDER 0.000 0.018 0.011 0.012 0.026

S.E. FOR ESTIMATED COVARIANCE MATRIX FOR THE LATENT VARIABLES

SC IDFIM SDFIM ID IC

________ ________ ________ ________ ________

SC 0.017

IDFIM 0.023 0.047

SDFIM 0.029 0.043 0.052

ID 0.031 0.167 0.155 1.627

IC 0.023 0.098 0.137 0.501 0.356

GENDER 0.017 0.018 0.018 0.115 0.071

S.E. FOR ESTIMATED COVARIANCE MATRIX FOR THE LATENT VARIABLES

GENDER

________

GENDER 0.018

EST./S.E. FOR ESTIMATED COVARIANCE MATRIX FOR THE LATENT VARIABLES

SR12 SR13 SR14 IC12 IC13

________ ________ ________ ________ ________

SR12 6.744

SR13 4.716 7.414

SR14 3.101 4.946 6.738

IC12 1.399 2.586 0.000 5.402

IC13 2.586 -0.468 0.000 2.586 4.582

IC14 0.000 0.000 -2.284 0.000 0.000

DFIM1 6.513 3.422 3.101 -4.503 0.000

DFIM2 3.422 6.514 4.946 0.000 -4.503

DFIM3 3.101 4.946 6.690 0.000 0.000

SD 1.660 1.961 1.826 0.000 0.000

SC 0.510 1.851 1.772 0.000 0.000

IDFIM 5.372 3.422 3.101 0.000 0.000

SDFIM -4.034 -0.550 0.292 0.000 0.000

ID -0.148 -0.751 -0.794 0.000 0.000

IC 0.112 -0.766 -0.768 0.000 0.000

GENDER 3.106 4.221 3.959 0.000 0.000

EST./S.E. FOR ESTIMATED COVARIANCE MATRIX FOR THE LATENT VARIABLES

IC14 DFIM1 DFIM2 DFIM3 SD

________ ________ ________ ________ ________

IC14 4.395

DFIM1 0.000 7.598

DFIM2 0.000 3.422 5.934

DFIM3 -4.503 3.101 4.946 5.945

SD 0.000 1.660 1.961 1.826 4.486

SC 0.000 0.510 1.851 1.772 3.947

IDFIM 0.000 5.372 3.422 3.101 1.660

SDFIM 0.000 -4.034 -0.550 0.292 -0.564

ID 0.000 -0.148 -0.751 -0.794 -2.924

IC 0.000 0.112 -0.766 -0.768 -1.842

GENDER 0.000 3.106 4.221 3.959 1.221

EST./S.E. FOR ESTIMATED COVARIANCE MATRIX FOR THE LATENT VARIABLES

SC IDFIM SDFIM ID IC

________ ________ ________ ________ ________

SC 5.746

IDFIM 0.510 5.372

SDFIM 0.713 -4.034 3.520

ID 0.598 -0.148 -0.364 4.176

IC -0.996 0.112 -0.607 3.915 2.397

GENDER 3.954 3.106 -0.571 0.600 -1.227

EST./S.E. FOR ESTIMATED COVARIANCE MATRIX FOR THE LATENT VARIABLES

GENDER

________

GENDER 13.838

TWO-TAILED P-VALUE FOR ESTIMATED COVARIANCE MATRIX FOR THE LATENT VARIABLES

SR12 SR13 SR14 IC12 IC13

________ ________ ________ ________ ________

SR12 0.000

SR13 0.000 0.000

SR14 0.002 0.000 0.000

IC12 0.162 0.010 1.000 0.000

IC13 0.010 0.640 1.000 0.010 0.000

IC14 1.000 1.000 0.022 1.000 1.000

DFIM1 0.000 0.001 0.002 0.000 1.000

DFIM2 0.001 0.000 0.000 1.000 0.000

DFIM3 0.002 0.000 0.000 1.000 1.000

SD 0.097 0.050 0.068 1.000 1.000

SC 0.610 0.064 0.076 1.000 1.000

IDFIM 0.000 0.001 0.002 1.000 1.000

SDFIM 0.000 0.582 0.770 1.000 1.000

ID 0.882 0.453 0.427 1.000 1.000

IC 0.911 0.444 0.442 1.000 1.000

GENDER 0.002 0.000 0.000 1.000 1.000

TWO-TAILED P-VALUE FOR ESTIMATED COVARIANCE MATRIX FOR THE LATENT VARIABLES

IC14 DFIM1 DFIM2 DFIM3 SD

________ ________ ________ ________ ________

IC14 0.000

DFIM1 1.000 0.000

DFIM2 1.000 0.001 0.000

DFIM3 0.000 0.002 0.000 0.000

SD 1.000 0.097 0.050 0.068 0.000

SC 1.000 0.610 0.064 0.076 0.000

IDFIM 1.000 0.000 0.001 0.002 0.097

SDFIM 1.000 0.000 0.582 0.770 0.573

ID 1.000 0.882 0.453 0.427 0.003

IC 1.000 0.911 0.444 0.442 0.066

GENDER 1.000 0.002 0.000 0.000 0.222

TWO-TAILED P-VALUE FOR ESTIMATED COVARIANCE MATRIX FOR THE LATENT VARIABLES

SC IDFIM SDFIM ID IC

________ ________ ________ ________ ________

SC 0.000

IDFIM 0.610 0.000

SDFIM 0.476 0.000 0.000

ID 0.550 0.882 0.716 0.000

IC 0.319 0.911 0.544 0.000 0.017

GENDER 0.000 0.002 0.568 0.549 0.220

TWO-TAILED P-VALUE FOR ESTIMATED COVARIANCE MATRIX FOR THE LATENT VARIABLES

GENDER

________

GENDER 0.000

ESTIMATED CORRELATION MATRIX FOR THE LATENT VARIABLES

SR12 SR13 SR14 IC12 IC13

________ ________ ________ ________ ________

SR12 1.000

SR13 0.514 1.000

SR14 0.354 0.579 1.000

IC12 0.098 0.129 0.000 1.000

IC13 0.091 -0.029 0.000 0.106 1.000

IC14 0.000 0.000 -0.200 0.000 0.000

DFIM1 0.730 0.322 0.282 -0.608 0.000

DFIM2 0.252 0.648 0.362 0.000 -0.780

DFIM3 0.201 0.329 0.711 0.000 0.000

SD 0.181 0.199 0.208 0.000 0.000

SC 0.062 0.241 0.286 0.000 0.000

IDFIM 0.836 0.482 0.423 0.000 0.000

SDFIM -0.687 -0.082 0.042 0.000 0.000

ID -0.016 -0.073 -0.087 0.000 0.000

IC 0.020 -0.174 -0.220 0.000 0.000

GENDER 0.189 0.239 0.257 0.000 0.000

ESTIMATED CORRELATION MATRIX FOR THE LATENT VARIABLES

IC14 DFIM1 DFIM2 DFIM3 SD

________ ________ ________ ________ ________

IC14 1.000

DFIM1 0.000 1.000

DFIM2 0.000 0.201 1.000

DFIM3 -0.831 0.160 0.206 1.000

SD 0.000 0.144 0.124 0.118 1.000

SC 0.000 0.049 0.151 0.162 0.598

IDFIM 0.000 0.667 0.302 0.240 0.216

SDFIM 0.000 -0.548 -0.051 0.024 -0.080

ID 0.000 -0.013 -0.046 -0.050 -0.497

IC 0.000 0.016 -0.109 -0.125 -0.352

GENDER 0.000 0.151 0.150 0.146 0.107

ESTIMATED CORRELATION MATRIX FOR THE LATENT VARIABLES

SC IDFIM SDFIM ID IC

________ ________ ________ ________ ________

SC 1.000

IDFIM 0.074 1.000

SDFIM 0.153 -0.821 1.000

ID 0.022 -0.019 -0.051 1.000

IC -0.080 0.024 -0.212 0.815 1.000

GENDER 0.421 0.226 -0.050 0.053 -0.189

ESTIMATED CORRELATION MATRIX FOR THE LATENT VARIABLES

GENDER

________

GENDER 1.000

S.E. FOR ESTIMATED CORRELATION MATRIX FOR THE LATENT VARIABLES

SR12 SR13 SR14 IC12 IC13

________ ________ ________ ________ ________

SR12 0.000

SR13 0.077 0.000

SR14 0.101 0.095 0.000

IC12 0.068 0.051 0.000 0.000

IC13 0.036 0.062 0.000 0.044 0.000

IC14 0.000 0.000 0.085 0.000 0.000

DFIM1 0.044 0.078 0.080 0.064 0.000

DFIM2 0.064 0.048 0.064 0.000 0.035

DFIM3 0.060 0.061 0.047 0.000 0.000

SD 0.111 0.097 0.111 0.000 0.000

SC 0.119 0.131 0.167 0.000 0.000

IDFIM 0.040 0.103 0.117 0.000 0.000

SDFIM 0.055 0.147 0.142 0.000 0.000

ID 0.107 0.097 0.110 0.000 0.000

IC 0.177 0.230 0.290 0.000 0.000

GENDER 0.056 0.051 0.060 0.000 0.000

S.E. FOR ESTIMATED CORRELATION MATRIX FOR THE LATENT VARIABLES

IC14 DFIM1 DFIM2 DFIM3 SD

________ ________ ________ ________ ________

IC14 0.000

DFIM1 0.000 0.000

DFIM2 0.000 0.056 0.000

DFIM3 0.036 0.050 0.045 0.000

SD 0.000 0.087 0.061 0.063 0.000

SC 0.000 0.095 0.083 0.095 0.115

IDFIM 0.000 0.054 0.071 0.069 0.134

SDFIM 0.000 0.065 0.093 0.081 0.144

ID 0.000 0.086 0.061 0.063 0.089

IC 0.000 0.141 0.145 0.166 0.197

GENDER 0.000 0.045 0.034 0.036 0.086

S.E. FOR ESTIMATED CORRELATION MATRIX FOR THE LATENT VARIABLES

SC IDFIM SDFIM ID IC

________ ________ ________ ________ ________

SC 0.000

IDFIM 0.143 0.000

SDFIM 0.217 0.047 0.000

ID 0.037 0.128 0.139 0.000

IC 0.075 0.212 0.342 0.109 0.000

GENDER 0.092 0.066 0.086 0.087 0.149

S.E. FOR ESTIMATED CORRELATION MATRIX FOR THE LATENT VARIABLES

GENDER

________

GENDER 0.000

EST./S.E. FOR ESTIMATED CORRELATION MATRIX FOR THE LATENT VARIABLES

SR12 SR13 SR14 IC12 IC13

________ ________ ________ ________ ________

SR12 999.000

SR13 6.694 999.000

SR14 3.495 6.107 999.000

IC12 1.441 2.526 0.000 999.000

IC13 2.518 -0.470 0.000 2.414 999.000

IC14 0.000 0.000 -2.349 0.000 0.000

DFIM1 16.647 4.135 3.507 -9.449 0.000

DFIM2 3.969 13.504 5.636 0.000 -22.067

DFIM3 3.358 5.426 15.130 0.000 0.000

SD 1.635 2.038 1.875 0.000 0.000

SC 0.518 1.845 1.718 0.000 0.000

IDFIM 21.129 4.668 3.627 0.000 0.000

SDFIM -12.406 -0.557 0.298 0.000 0.000

ID -0.148 -0.752 -0.795 0.000 0.000

IC 0.113 -0.757 -0.759 0.000 0.000

GENDER 3.345 4.667 4.252 0.000 0.000

EST./S.E. FOR ESTIMATED CORRELATION MATRIX FOR THE LATENT VARIABLES

IC14 DFIM1 DFIM2 DFIM3 SD

________ ________ ________ ________ ________

IC14 999.000

DFIM1 0.000 999.000

DFIM2 0.000 3.615 999.000

DFIM3 -23.163 3.217 4.550 999.000

SD 0.000 1.663 2.030 1.888 999.000

SC 0.000 0.520 1.814 1.718 5.199

IDFIM 0.000 12.393 4.228 3.476 1.618

SDFIM 0.000 -8.469 -0.552 0.298 -0.554

ID 0.000 -0.148 -0.749 -0.791 -5.597

IC 0.000 0.113 -0.750 -0.754 -1.784

GENDER 0.000 3.358 4.381 4.056 1.245

EST./S.E. FOR ESTIMATED CORRELATION MATRIX FOR THE LATENT VARIABLES

SC IDFIM SDFIM ID IC

________ ________ ________ ________ ________

SC 999.000

IDFIM 0.518 999.000

SDFIM 0.702 -17.637 999.000

ID 0.605 -0.148 -0.366 999.000

IC -1.069 0.113 -0.619 7.480 999.000

GENDER 4.565 3.434 -0.579 0.608 -1.267

EST./S.E. FOR ESTIMATED CORRELATION MATRIX FOR THE LATENT VARIABLES

GENDER

________

GENDER 999.000

TWO-TAILED P-VALUE FOR ESTIMATED CORRELATION MATRIX FOR THE LATENT VARIABLES

SR12 SR13 SR14 IC12 IC13

________ ________ ________ ________ ________

SR12 0.000

SR13 0.000 0.000

SR14 0.000 0.000 0.000

IC12 0.150 0.012 1.000 0.000

IC13 0.012 0.639 1.000 0.016 0.000

IC14 1.000 1.000 0.019 1.000 1.000

DFIM1 0.000 0.000 0.000 0.000 1.000

DFIM2 0.000 0.000 0.000 1.000 0.000

DFIM3 0.001 0.000 0.000 1.000 1.000

SD 0.102 0.042 0.061 1.000 1.000

SC 0.604 0.065 0.086 1.000 1.000

IDFIM 0.000 0.000 0.000 1.000 1.000

SDFIM 0.000 0.578 0.766 1.000 1.000

ID 0.882 0.452 0.426 1.000 1.000

IC 0.910 0.449 0.448 1.000 1.000

GENDER 0.001 0.000 0.000 1.000 1.000

TWO-TAILED P-VALUE FOR ESTIMATED CORRELATION MATRIX FOR THE LATENT VARIABLES

IC14 DFIM1 DFIM2 DFIM3 SD

________ ________ ________ ________ ________

IC14 0.000

DFIM1 1.000 0.000

DFIM2 1.000 0.000 0.000

DFIM3 0.000 0.001 0.000 0.000

SD 1.000 0.096 0.042 0.059 0.000

SC 1.000 0.603 0.070 0.086 0.000

IDFIM 1.000 0.000 0.000 0.001 0.106

SDFIM 1.000 0.000 0.581 0.765 0.580

ID 1.000 0.882 0.454 0.429 0.000

IC 1.000 0.910 0.453 0.451 0.074

GENDER 1.000 0.001 0.000 0.000 0.213

TWO-TAILED P-VALUE FOR ESTIMATED CORRELATION MATRIX FOR THE LATENT VARIABLES

SC IDFIM SDFIM ID IC

________ ________ ________ ________ ________

SC 0.000

IDFIM 0.605 0.000

SDFIM 0.483 0.000 0.000

ID 0.545 0.882 0.714 0.000

IC 0.285 0.910 0.536 0.000 0.000

GENDER 0.000 0.001 0.563 0.543 0.205

TWO-TAILED P-VALUE FOR ESTIMATED CORRELATION MATRIX FOR THE LATENT VARIABLES

GENDER

________

GENDER 0.000

DIAGRAM INFORMATION

Use View Diagram under the Diagram menu in the Mplus Editor to view the diagram.

If running Mplus from the Mplus Diagrammer, the diagram opens automatically.

Diagram output

u:\windows\dcn r&r\r&r analyses\prediction model\latent difference growth model predicting mu two-part.dgm

Beginning Time: 15:44:48

Ending Time: 15:44:57

Elapsed Time: 00:00:09

MUTHEN & MUTHEN

3463 Stoner Ave.

Los Angeles, CA 90066

Tel: (310) 391-9971

Fax: (310) 391-8971

Web: www.StatModel.com

Support: Support@StatModel.com

Copyright (c) 1998-2018 Muthen & Muthen
