# Supplementary material for: Mind the gap: A review and recommendations for statistically evaluating Dual Systems models of adolescent risk behavior
Source: Dev Cogn Neurosci. 2019 Jul 25;39:100681. doi: 10.1016/j.dcn.2019.100681 (PMC6969358; doi:10.1016/j.dcn.2019.100681)
Supplement: Supplementary file 5 [file mmc5.docx]

Mplus VERSION 8.2

MUTHEN & MUTHEN

04/05/2019 4:01 PM

INPUT INSTRUCTIONS

Title: GMM Predicting Alcohol Two-Part Model

Data: file is predictR&R2.dat;

Variable:

NAMES ARE

subject gender alcd12-alcd20 alcc12-alcc20 c_imb;

!For the class variable (c_imb) 1 is the class with the larger imbalance

!and 0 is the class with the smaller imbalance

USEV

alcd12-alcd20 alcc12-alcc20 c_imb gender;

missing are .;

CATEGORICAL ARE alcd12-alcd20;

Define:

alcc12=log(alcc12+1);

alcc13=log(alcc13+1);

alcc14=log(alcc14+1);

alcc15=log(alcc15+1);

alcc16=log(alcc16+1);

alcc17=log(alcc17+1);

alcc18=log(alcc18+1);

alcc19=log(alcc19+1);

alcc20=log(alcc20+1);

Analysis: estimator is MLR;

model=nocovariances;

mconvergence=.01;

Model:

!Alchol Two-Part Growth Model With Random Effects

!Dichotomous Growth Model ages 12-20

id sd | alcd12@-1 alcd13@0 alcd14@1 alcd15* alcd16@3 alcd17@4

alcd18@5 alcd19@6 alcd20*;

sd BY alcd15*2.32557;

sd BY alcd20*6.22476;

!Continuous Growth Model ages 12-16

ic sc | alcc12@-1 alcc13@0 alcc14@1 alcc15@2 alcc16@3 alcc17@3

alcc18@3 alcc19@3 alcc20@3;

!Continuous Growth Model ages 16-20

ic sc2 | alcc12@0 alcc13@0 alcc14@0 alcc15@0 alcc16@0 alcc17@1

alcc18@2 alcc19* alcc20*;

sc2 BY alcc19*2.32512;

sc2 BY alcc20*2.39166;

!Covariances Between Growth Factors

id WITH sd*-0.72279;

id WITH ic*0.83417;

id WITH sc*0.25907;

id WITH sc2@0;

ic WITH sc*0.08127;

ic WITH sc2*-0.10838;

sc WITH sc2@0;

sd WITH ic@0;

sd WITH sc@0;

sd WITH sc2*0.16021;

!Continuous Alcohl Use Intercepts Constrained to 0 for Model

!Identification

[ alcc12@0 ];

[ alcc13@0 ];

[ alcc14@0 ];

[ alcc15@0 ];

[ alcc16@0 ];

[ alcc17@0 ];

[ alcc18@0 ];

[ alcc19@0 ];

[ alcc20@0 ];

[ id@0 ];

!Growth Factor Means Start Values

[ sd*0.97615 ];

[ ic*0.45873 ];

[ sc*0.41418 ];

[ sc2*1.03536 ];

!Dichotomous Alcohol Use Thresholds Start Values

[ alcd12$1*3.67237 ] (28);

[ alcd13$1*3.67237 ] (28);

[ alcd14$1*3.67237 ] (28);

[ alcd15$1*3.67237 ] (28);

[ alcd16$1*3.67237 ] (28);

[ alcd17$1*3.67237 ] (28);

[ alcd18$1*3.67237 ] (28);

[ alcd19$1*3.67237 ] (28);

[ alcd20$1*3.67237 ] (28);

!Variance of Continuous Alcohol Use Constrained to 0 Due

!to a Negative Residual Variance

alcc12@0;

!Start Values for Variances of Continous Alcohol Use and

!Alcohol Use Growth Factors

alcc13*0.43376;

alcc14*0.60803;

alcc15*0.93064;

alcc16*0.73305;

alcc17*1.55435;

alcc18*1.50926;

alcc19*0.81892;

alcc20*0.74486;

id*7.48873;

sd*0.30789;

ic*0.30434;

sc*0.04254;

sc2*0.35544;

!Class Membership Predicting Growth in Alcohol Use

id sd ic sc sc2 on c_imb;

!Controlling for Gender

id sd ic sc sc2 on gender;

Output: res stdyx tech4 sampstat svalues;

*** WARNING

Data set contains cases with missing on x-variables.

These cases were not included in the analysis.

Number of cases with missing on x-variables: 25

1 WARNING(S) FOUND IN THE INPUT INSTRUCTIONS

Revised Classes Predicting Alcohol 2-Part DCN R&R

SUMMARY OF ANALYSIS

Number of groups 1

Number of observations 362

Number of dependent variables 18

Number of independent variables 2

Number of continuous latent variables 5

Observed dependent variables

Continuous

ALCC12 ALCC13 ALCC14 ALCC15 ALCC16 ALCC17

ALCC18 ALCC19 ALCC20

Binary and ordered categorical (ordinal)

ALCD12 ALCD13 ALCD14 ALCD15 ALCD16 ALCD17

ALCD18 ALCD19 ALCD20

Observed independent variables

GENDER C_IMB

Continuous latent variables

SD SC2 ID IC SC

Estimator MLR

Information matrix OBSERVED

Optimization Specifications for the Quasi-Newton Algorithm for

Continuous Outcomes

Maximum number of iterations 100

Convergence criterion 0.100D-05

Optimization Specifications for the EM Algorithm

Maximum number of iterations 500

Convergence criteria

Loglikelihood change 0.100D-02

Relative loglikelihood change 0.100D-05

Derivative 0.100D-01

Optimization Specifications for the M step of the EM Algorithm for

Categorical Latent variables

Number of M step iterations 1

M step convergence criterion 0.100D-02

Basis for M step termination ITERATION

Optimization Specifications for the M step of the EM Algorithm for

Censored, Binary or Ordered Categorical (Ordinal), Unordered

Categorical (Nominal) and Count Outcomes

Number of M step iterations 1

M step convergence criterion 0.100D-02

Basis for M step termination ITERATION

Maximum value for logit thresholds 15

Minimum value for logit thresholds -15

Minimum expected cell size for chi-square 0.100D-01

Maximum number of iterations for H1 2000

Convergence criterion for H1 0.100D-03

Optimization algorithm EMA

Integration Specifications

Type STANDARD

Number of integration points 15

Dimensions of numerical integration 2

Adaptive quadrature ON

Link LOGIT

Cholesky OFF

Input data file(s)

predictR&R.dat

Input data format FREE

SUMMARY OF DATA

Number of missing data patterns 193

Number of y missing data patterns 106

Number of u missing data patterns 58

COVARIANCE COVERAGE OF DATA

Minimum covariance coverage value 0.100

PROPORTION OF DATA PRESENT

Covariance Coverage

ALCD12 ALCD13 ALCD14 ALCD15 ALCD16

________ ________ ________ ________ ________

ALCD12 0.972

ALCD13 0.914 0.942

ALCD14 0.939 0.923 0.964

ALCD15 0.884 0.876 0.890 0.906

ALCD16 0.851 0.843 0.856 0.823 0.876

ALCD17 0.787 0.771 0.785 0.751 0.743

ALCD18 0.757 0.751 0.765 0.738 0.710

ALCD19 0.804 0.793 0.815 0.776 0.760

ALCD20 0.613 0.608 0.616 0.597 0.583

ALCC12 0.039 0.030 0.036 0.033 0.028

ALCC13 0.105 0.116 0.116 0.110 0.099

ALCC14 0.202 0.196 0.204 0.199 0.174

ALCC15 0.307 0.296 0.312 0.315 0.282

ALCC16 0.356 0.351 0.359 0.354 0.367

ALCC17 0.395 0.398 0.398 0.398 0.384

ALCC18 0.478 0.483 0.489 0.475 0.459

ALCC19 0.586 0.580 0.599 0.572 0.561

ALCC20 0.461 0.461 0.464 0.453 0.450

GENDER 0.972 0.942 0.964 0.906 0.876

C_IMB 0.972 0.942 0.964 0.906 0.876

Covariance Coverage

ALCD17 ALCD18 ALCD19 ALCD20 ALCC12

________ ________ ________ ________ ________

ALCD17 0.804

ALCD18 0.652 0.782

ALCD19 0.699 0.680 0.823

ALCD20 0.500 0.489 0.547 0.630

ALCC12 0.033 0.028 0.030 0.019 0.039

ALCC13 0.075 0.088 0.088 0.077 0.022

ALCC14 0.149 0.152 0.169 0.146 0.033

ALCC15 0.229 0.243 0.260 0.224 0.028

ALCC16 0.290 0.293 0.323 0.262 0.028

ALCC17 0.409 0.331 0.356 0.262 0.019

ALCC18 0.423 0.497 0.428 0.318 0.028

ALCC19 0.508 0.483 0.599 0.409 0.025

ALCC20 0.378 0.367 0.417 0.475 0.017

GENDER 0.804 0.782 0.823 0.630 0.039

C_IMB 0.804 0.782 0.823 0.630 0.039

Covariance Coverage

ALCC13 ALCC14 ALCC15 ALCC16 ALCC17

________ ________ ________ ________ ________

ALCC13 0.116

ALCC14 0.075 0.204

ALCC15 0.086 0.152 0.315

ALCC16 0.072 0.127 0.229 0.367

ALCC17 0.052 0.113 0.193 0.238 0.409

ALCC18 0.075 0.124 0.199 0.240 0.285

ALCC19 0.072 0.144 0.229 0.282 0.315

ALCC20 0.069 0.124 0.199 0.229 0.235

GENDER 0.116 0.204 0.315 0.367 0.409

C_IMB 0.116 0.204 0.315 0.367 0.409

Covariance Coverage

ALCC18 ALCC19 ALCC20 GENDER C_IMB

________ ________ ________ ________ ________

ALCC18 0.497

ALCC19 0.387 0.599

ALCC20 0.296 0.359 0.475

GENDER 0.497 0.599 0.475 1.000

C_IMB 0.497 0.599 0.475 1.000 1.000

WARNING: THE COVARIANCE COVERAGE FALLS BELOW THE SPECIFIED LIMIT.

PROPORTION OF DATA PRESENT FOR U

Covariance Coverage

ALCD12 ALCD13 ALCD14 ALCD15 ALCD16

________ ________ ________ ________ ________

ALCD12 0.972

ALCD13 0.914 0.942

ALCD14 0.939 0.923 0.964

ALCD15 0.884 0.876 0.890 0.906

ALCD16 0.851 0.843 0.856 0.823 0.876

ALCD17 0.787 0.771 0.785 0.751 0.743

ALCD18 0.757 0.751 0.765 0.738 0.710

ALCD19 0.804 0.793 0.815 0.776 0.760

ALCD20 0.613 0.608 0.616 0.597 0.583

Covariance Coverage

ALCD17 ALCD18 ALCD19 ALCD20

________ ________ ________ ________

ALCD17 0.804

ALCD18 0.652 0.782

ALCD19 0.699 0.680 0.823

ALCD20 0.500 0.489 0.547 0.630

PROPORTION OF DATA PRESENT FOR Y

Covariance Coverage

ALCC12 ALCC13 ALCC14 ALCC15 ALCC16

________ ________ ________ ________ ________

ALCC12 0.039

ALCC13 0.022 0.116

ALCC14 0.033 0.075 0.204

ALCC15 0.028 0.086 0.152 0.315

ALCC16 0.028 0.072 0.127 0.229 0.367

ALCC17 0.019 0.052 0.113 0.193 0.238

ALCC18 0.028 0.075 0.124 0.199 0.240

ALCC19 0.025 0.072 0.144 0.229 0.282

ALCC20 0.017 0.069 0.124 0.199 0.229

GENDER 0.039 0.116 0.204 0.315 0.367

C_IMB 0.039 0.116 0.204 0.315 0.367

Covariance Coverage

ALCC17 ALCC18 ALCC19 ALCC20 GENDER

________ ________ ________ ________ ________

ALCC17 0.409

ALCC18 0.285 0.497

ALCC19 0.315 0.387 0.599

ALCC20 0.235 0.296 0.359 0.475

GENDER 0.409 0.497 0.599 0.475 1.000

C_IMB 0.409 0.497 0.599 0.475 1.000

Covariance Coverage

C_IMB

________

C_IMB 1.000

THE COVARIANCE COVERAGE FALLS BELOW THE SPECIFIED LIMIT. THE MISSING

DATA EM ALGORITHM WILL NOT BE INITIATED. CHECK YOUR DATA OR LOWER THE

COVARIANCE COVERAGE LIMIT.

UNIVARIATE PROPORTIONS AND COUNTS FOR CATEGORICAL VARIABLES

ALCD12

Category 1 0.960 338.000

Category 2 0.040 14.000

ALCD13

Category 1 0.877 299.000

Category 2 0.123 42.000

ALCD14

Category 1 0.788 275.000

Category 2 0.212 74.000

ALCD15

Category 1 0.652 214.000

Category 2 0.348 114.000

ALCD16

Category 1 0.580 184.000

Category 2 0.420 133.000

ALCD17

Category 1 0.491 143.000

Category 2 0.509 148.000

ALCD18

Category 1 0.364 103.000

Category 2 0.636 180.000

ALCD19

Category 1 0.272 81.000

Category 2 0.728 217.000

ALCD20

Category 1 0.246 56.000

Category 2 0.754 172.000

SAMPLE STATISTICS

UNIVARIATE SAMPLE STATISTICS

UNIVARIATE HIGHER-ORDER MOMENT DESCRIPTIVE STATISTICS

Variable/ Mean/ Skewness/ Minimum/ % with Percentiles

Sample Size Variance Kurtosis Maximum Min/Max 20%/60% 40%/80% Median

ALCC12 0.568 1.433 0.223 42.86% 0.223 0.223 0.405

14.000 0.218 0.940 1.792 7.14% 0.405 0.693

ALCC13 0.819 1.730 0.010 2.38% 0.223 0.405 0.405

42.000 0.667 2.354 3.453 2.38% 0.693 1.253

ALCC14 1.310 1.219 0.223 17.57% 0.405 0.693 1.099

74.000 1.109 1.248 4.575 1.35% 1.253 2.079

ALCC15 1.959 0.672 0.405 11.40% 0.693 1.386 1.748

114.000 1.534 -0.326 5.347 0.88% 2.197 3.045

ALCC16 2.209 0.493 0.405 12.78% 0.693 1.609 2.197

133.000 1.926 -0.548 5.707 0.75% 2.565 3.258

ALCC17 3.206 0.310 0.405 3.38% 1.792 2.565 3.045

148.000 2.616 -0.485 7.463 0.68% 3.584 4.691

ALCC18 4.438 -0.284 0.693 0.56% 2.944 4.190 4.654

180.000 2.866 -0.561 7.463 1.67% 5.017 5.666

ALCC19 4.516 -0.366 1.099 5.99% 3.219 4.111 4.691

217.000 2.691 -0.525 7.353 0.92% 5.056 6.033

ALCC20 4.594 -0.379 1.099 5.23% 3.611 4.290 4.673

172.000 2.437 -0.379 7.310 1.16% 5.056 6.064

GENDER 0.445 0.222 0.000 55.52% 0.000 0.000 0.000

362.000 0.247 -1.951 1.000 44.48% 1.000 1.000

C_IMB 0.108 2.530 0.000 89.23% 0.000 0.000 0.000

362.000 0.096 4.403 1.000 10.77% 0.000 0.000

THE MODEL ESTIMATION TERMINATED NORMALLY

MODEL FIT INFORMATION

Number of Free Parameters 38

Loglikelihood

H0 Value -2992.410

H0 Scaling Correction Factor 0.9983

for MLR

Information Criteria

Akaike (AIC) 6060.820

Bayesian (BIC) 6208.703

Sample-Size Adjusted BIC 6088.146

(n* = (n + 2) / 24)

MODEL RESULTS

Two-Tailed

Estimate S.E. Est./S.E. P-Value

ID |

ALCD12 1.000 0.000 999.000 999.000

ALCD13 1.000 0.000 999.000 999.000

ALCD14 1.000 0.000 999.000 999.000

ALCD15 1.000 0.000 999.000 999.000

ALCD16 1.000 0.000 999.000 999.000

ALCD17 1.000 0.000 999.000 999.000

ALCD18 1.000 0.000 999.000 999.000

ALCD19 1.000 0.000 999.000 999.000

ALCD20 1.000 0.000 999.000 999.000

SD |

ALCD12 -1.000 0.000 999.000 999.000

ALCD13 0.000 0.000 999.000 999.000

ALCD14 1.000 0.000 999.000 999.000

ALCD15 2.327 0.146 15.971 0.000

ALCD16 3.000 0.000 999.000 999.000

ALCD17 4.000 0.000 999.000 999.000

ALCD18 5.000 0.000 999.000 999.000

ALCD19 6.000 0.000 999.000 999.000

ALCD20 6.185 0.374 16.519 0.000

IC |

ALCC12 1.000 0.000 999.000 999.000

ALCC13 1.000 0.000 999.000 999.000

ALCC14 1.000 0.000 999.000 999.000

ALCC15 1.000 0.000 999.000 999.000

ALCC16 1.000 0.000 999.000 999.000

ALCC17 1.000 0.000 999.000 999.000

ALCC18 1.000 0.000 999.000 999.000

ALCC19 1.000 0.000 999.000 999.000

ALCC20 1.000 0.000 999.000 999.000

SC |

ALCC12 -1.000 0.000 999.000 999.000

ALCC13 0.000 0.000 999.000 999.000

ALCC14 1.000 0.000 999.000 999.000

ALCC15 2.000 0.000 999.000 999.000

ALCC16 3.000 0.000 999.000 999.000

ALCC17 3.000 0.000 999.000 999.000

ALCC18 3.000 0.000 999.000 999.000

ALCC19 3.000 0.000 999.000 999.000

ALCC20 3.000 0.000 999.000 999.000

SC2 |

ALCC12 0.000 0.000 999.000 999.000

ALCC13 0.000 0.000 999.000 999.000

ALCC14 0.000 0.000 999.000 999.000

ALCC15 0.000 0.000 999.000 999.000

ALCC16 0.000 0.000 999.000 999.000

ALCC17 1.000 0.000 999.000 999.000

ALCC18 2.000 0.000 999.000 999.000

ALCC19 2.312 0.098 23.680 0.000

ALCC20 2.375 0.109 21.770 0.000

ID ON

C_IMB -0.339 0.511 -0.664 0.507

GENDER -0.292 0.384 -0.762 0.446

SD ON

C_IMB 0.050 0.146 0.342 0.732

GENDER -0.003 0.091 -0.028 0.978

IC ON

C_IMB -0.169 0.338 -0.501 0.616

GENDER -0.067 0.139 -0.482 0.630

SC ON

C_IMB -0.111 0.133 -0.838 0.402

GENDER 0.032 0.059 0.543 0.587

SC2 ON

C_IMB 0.098 0.147 0.667 0.505

GENDER 0.244 0.108 2.268 0.023

ID WITH

SD -0.390 0.217 -1.798 0.072

IC 1.081 0.377 2.868 0.004

SC 0.170 0.116 1.462 0.144

SC2 0.000 0.000 999.000 999.000

IC WITH

SC 0.072 0.017 4.171 0.000

SC2 -0.090 0.059 -1.520 0.129

SD 0.000 0.000 999.000 999.000

SC WITH

SC2 0.000 0.000 999.000 999.000

SD 0.000 0.000 999.000 999.000

SD WITH

SC2 0.162 0.039 4.156 0.000

Intercepts

ALCC12 0.000 0.000 999.000 999.000

ALCC13 0.000 0.000 999.000 999.000

ALCC14 0.000 0.000 999.000 999.000

ALCC15 0.000 0.000 999.000 999.000

ALCC16 0.000 0.000 999.000 999.000

ALCC17 0.000 0.000 999.000 999.000

ALCC18 0.000 0.000 999.000 999.000

ALCC19 0.000 0.000 999.000 999.000

ALCC20 0.000 0.000 999.000 999.000

SD 0.973 0.084 11.630 0.000

SC2 0.914 0.078 11.732 0.000

ID 0.000 0.000 999.000 999.000

IC 0.329 0.216 1.523 0.128

SC 0.482 0.086 5.616 0.000

Thresholds

ALCD12$1 3.476 0.337 10.300 0.000

ALCD13$1 3.476 0.337 10.300 0.000

ALCD14$1 3.476 0.337 10.300 0.000

ALCD15$1 3.476 0.337 10.300 0.000

ALCD16$1 3.476 0.337 10.300 0.000

ALCD17$1 3.476 0.337 10.300 0.000

ALCD18$1 3.476 0.337 10.300 0.000

ALCD19$1 3.476 0.337 10.300 0.000

ALCD20$1 3.476 0.337 10.300 0.000

Residual Variances

ALCC12 0.000 0.000 999.000 999.000

ALCC13 0.447 0.110 4.071 0.000

ALCC14 0.580 0.138 4.210 0.000

ALCC15 0.935 0.160 5.828 0.000

ALCC16 0.784 0.147 5.324 0.000

ALCC17 1.537 0.181 8.482 0.000

ALCC18 1.476 0.201 7.349 0.000

ALCC19 0.816 0.219 3.728 0.000

ALCC20 0.790 0.169 4.691 0.000

SD 0.248 0.057 4.368 0.000

SC2 0.330 0.055 5.952 0.000

ID 5.970 1.166 5.122 0.000

IC 0.370 0.122 3.022 0.003

SC 0.033 0.012 2.756 0.006

STANDARDIZED MODEL RESULTS

STDYX Standardization

Two-Tailed

Estimate S.E. Est./S.E. P-Value

ID |

ALCD12 0.763 0.020 39.055 0.000

ALCD13 0.804 0.028 28.816 0.000

ALCD14 0.828 0.042 19.653 0.000

ALCD15 0.825 0.061 13.636 0.000

ALCD16 0.809 0.068 11.935 0.000

ALCD17 0.770 0.073 10.509 0.000

ALCD18 0.720 0.074 9.758 0.000

ALCD19 0.666 0.071 9.384 0.000

ALCD20 0.656 0.076 8.605 0.000

SD |

ALCD12 -0.155 0.013 -12.331 0.000

ALCD13 0.000 0.000 999.000 999.000

ALCD14 0.169 0.016 10.756 0.000

ALCD15 0.391 0.046 8.433 0.000

ALCD16 0.494 0.050 9.902 0.000

ALCD17 0.626 0.063 9.912 0.000

ALCD18 0.732 0.072 10.202 0.000

ALCD19 0.813 0.076 10.725 0.000

ALCD20 0.825 0.076 10.805 0.000

IC |

ALCC12 1.197 0.118 10.104 0.000

ALCC13 0.675 0.072 9.363 0.000

ALCC14 0.574 0.082 6.998 0.000

ALCC15 0.463 0.075 6.204 0.000

ALCC16 0.442 0.072 6.185 0.000

ALCC17 0.364 0.061 6.002 0.000

ALCC18 0.322 0.051 6.277 0.000

ALCC19 0.334 0.052 6.469 0.000

ALCC20 0.331 0.052 6.408 0.000

SC |

ALCC12 -0.364 0.140 -2.605 0.009

ALCC13 0.000 0.000 999.000 999.000

ALCC14 0.174 0.033 5.215 0.000

ALCC15 0.282 0.050 5.684 0.000

ALCC16 0.403 0.070 5.784 0.000

ALCC17 0.332 0.057 5.819 0.000

ALCC18 0.293 0.054 5.423 0.000

ALCC19 0.304 0.057 5.327 0.000

ALCC20 0.301 0.057 5.265 0.000

SC2 |

ALCC12 0.000 0.000 999.000 999.000

ALCC13 0.000 0.000 999.000 999.000

ALCC14 0.000 0.000 999.000 999.000

ALCC15 0.000 0.000 999.000 999.000

ALCC16 0.000 0.000 999.000 999.000

ALCC17 0.350 0.030 11.571 0.000

ALCC18 0.619 0.046 13.561 0.000

ALCC19 0.742 0.056 13.186 0.000

ALCC20 0.755 0.049 15.489 0.000

ID ON

C_IMB -0.043 0.064 -0.665 0.506

GENDER -0.059 0.077 -0.770 0.441

SD ON

C_IMB 0.031 0.091 0.343 0.731

GENDER -0.003 0.091 -0.028 0.978

IC ON

C_IMB -0.086 0.170 -0.507 0.612

GENDER -0.055 0.112 -0.489 0.625

SC ON

C_IMB -0.185 0.218 -0.852 0.394

GENDER 0.086 0.159 0.542 0.588

SC2 ON

C_IMB 0.052 0.078 0.666 0.506

GENDER 0.206 0.087 2.378 0.017

ID WITH

SD -0.320 0.133 -2.403 0.016

IC 0.728 0.179 4.069 0.000

SC 0.382 0.210 1.817 0.069

SC2 0.000 0.000 999.000 999.000

IC WITH

SC 0.654 0.160 4.080 0.000

SC2 -0.257 0.176 -1.459 0.144

SD 0.000 0.000 999.000 999.000

SC WITH

SC2 0.000 0.000 999.000 999.000

SD 0.000 0.000 999.000 999.000

SD WITH

SC2 0.566 0.131 4.311 0.000

Intercepts

ALCC12 0.000 0.000 999.000 999.000

ALCC13 0.000 0.000 999.000 999.000

ALCC14 0.000 0.000 999.000 999.000

ALCC15 0.000 0.000 999.000 999.000

ALCC16 0.000 0.000 999.000 999.000

ALCC17 0.000 0.000 999.000 999.000

ALCC18 0.000 0.000 999.000 999.000

ALCC19 0.000 0.000 999.000 999.000

ALCC20 0.000 0.000 999.000 999.000

SD 1.951 0.207 9.404 0.000

SC2 1.553 0.202 7.701 0.000

ID 0.000 0.000 999.000 999.000

IC 0.538 0.426 1.262 0.207

SC 2.595 0.804 3.226 0.001

Thresholds

ALCD12$1 1.082 0.089 12.094 0.000

ALCD13$1 1.140 0.091 12.501 0.000

ALCD14$1 1.174 0.096 12.263 0.000

ALCD15$1 1.170 0.105 11.184 0.000

ALCD16$1 1.147 0.109 10.510 0.000

ALCD17$1 1.092 0.112 9.767 0.000

ALCD18$1 1.021 0.110 9.272 0.000

ALCD19$1 0.944 0.105 8.968 0.000

ALCD20$1 0.930 0.112 8.307 0.000

Residual Variances

ALCC12 0.000 999.000 999.000 999.000

ALCC13 0.544 0.097 5.592 0.000

ALCC14 0.511 0.077 6.649 0.000

ALCC15 0.537 0.058 9.227 0.000

ALCC16 0.410 0.069 5.985 0.000

ALCC17 0.545 0.043 12.550 0.000

ALCC18 0.409 0.050 8.148 0.000

ALCC19 0.243 0.062 3.930 0.000

ALCC20 0.231 0.049 4.760 0.000

SD 0.999 0.006 177.781 0.000

SC2 0.953 0.037 25.537 0.000

ID 0.994 0.011 90.290 0.000

IC 0.989 0.035 28.161 0.000

SC 0.960 0.082 11.662 0.000

R-SQUARE

Observed Two-Tailed

Variable Estimate S.E. Est./S.E. P-Value

ALCD12 0.681 0.049 13.867 0.000

ALCD13 0.646 0.045 14.408 0.000

ALCD14 0.624 0.038 16.289 0.000

ALCD15 0.627 0.034 18.679 0.000

ALCD16 0.642 0.034 18.965 0.000

ALCD17 0.676 0.035 19.243 0.000

ALCD18 0.716 0.035 20.473 0.000

ALCD19 0.757 0.033 22.828 0.000

ALCD20 0.764 0.037 20.573 0.000

ALCC12 1.000 999.000 999.000 999.000

ALCC13 0.456 0.097 4.681 0.000

ALCC14 0.489 0.077 6.367 0.000

ALCC15 0.463 0.058 7.960 0.000

ALCC16 0.590 0.069 8.595 0.000

ALCC17 0.455 0.043 10.487 0.000

ALCC18 0.591 0.050 11.783 0.000

ALCC19 0.757 0.062 12.240 0.000

ALCC20 0.769 0.049 15.852 0.000

Latent Two-Tailed

Variable Estimate S.E. Est./S.E. P-Value

SD 0.001 0.006 0.172 0.863

SC2 0.047 0.037 1.248 0.212

ID 0.006 0.011 0.516 0.606

IC 0.011 0.035 0.312 0.755

SC 0.040 0.082 0.483 0.629

QUALITY OF NUMERICAL RESULTS

Condition Number for the Information Matrix 0.173E-04

(ratio of smallest to largest eigenvalue)

MODEL COMMAND WITH FINAL ESTIMATES USED AS STARTING VALUES

id sd | alcd12@-1 alcd13@0 alcd14@1 alcd15* alcd16@3 alcd17@4

alcd18@5 alcd19@6 alcd20*;

ic sc | alcc12@-1 alcc13@0 alcc14@1 alcc15@2 alcc16@3 alcc17@3

alcc18@3 alcc19@3 alcc20@3;

ic sc2 | alcc12@0 alcc13@0 alcc14@0 alcc15@0 alcc16@0 alcc17@1

alcc18@2 alcc19* alcc20*;

sd BY alcd15*2.32680;

sd BY alcd20*6.18476;

sc2 BY alcc19*2.31249;

sc2 BY alcc20*2.37505;

id ON c_imb*-0.33884;

id ON gender*-0.29234;

sd ON c_imb*0.05011;

sd ON gender*-0.00253;

ic ON c_imb*-0.16942;

ic ON gender*-0.06713;

sc ON c_imb*-0.11111;

sc ON gender*0.03231;

sc2 ON c_imb*0.09819;

sc2 ON gender*0.24419;

id WITH sd*-0.39026;

id WITH ic*1.08121;

id WITH sc*0.16968;

id WITH sc2@0;

ic WITH sc*0.07236;

ic WITH sc2*-0.08996;

ic WITH sd@0;

sc WITH sc2@0;

sc WITH sd@0;

sd WITH sc2*0.16210;

[ alcc12@0 ];

[ alcc13@0 ];

[ alcc14@0 ];

[ alcc15@0 ];

[ alcc16@0 ];

[ alcc17@0 ];

[ alcc18@0 ];

[ alcc19@0 ];

[ alcc20@0 ];

[ sd*0.97281 ];

[ sc2*0.91354 ];

[ id@0 ];

[ ic*0.32891 ];

[ sc*0.48194 ];

[ alcd12$1*3.47570 ] (38);

[ alcd13$1*3.47570 ] (38);

[ alcd14$1*3.47570 ] (38);

[ alcd15$1*3.47570 ] (38);

[ alcd16$1*3.47570 ] (38);

[ alcd17$1*3.47570 ] (38);

[ alcd18$1*3.47570 ] (38);

[ alcd19$1*3.47570 ] (38);

[ alcd20$1*3.47570 ] (38);

alcc12@0;

alcc13*0.44664;

alcc14*0.58034;

alcc15*0.93491;

alcc16*0.78427;

alcc17*1.53701;

alcc18*1.47626;

alcc19*0.81637;

alcc20*0.79048;

sd*0.24848;

sc2*0.33009;

id*5.97048;

ic*0.36982;

sc*0.03313;

RESIDUAL OUTPUT

ESTIMATED MODEL AND RESIDUALS (OBSERVED - ESTIMATED)

Model Estimated Means

ALCC12 ALCC13 ALCC14 ALCC15 ALCC16

________ ________ ________ ________ ________

-0.204 0.281 0.765 1.249 1.734

Model Estimated Means

ALCC17 ALCC18 ALCC19 ALCC20

________ ________ ________ ________

2.767 3.799 4.122 4.187

Residuals for Means

ALCC12 ALCC13 ALCC14 ALCC15 ALCC16

________ ________ ________ ________ ________

0.771 0.538 0.545 0.709 0.475

Residuals for Means

ALCC17 ALCC18 ALCC19 ALCC20

________ ________ ________ ________

0.440 0.639 0.394 0.408

Model Estimated Covariances

ALCC12 ALCC13 ALCC14 ALCC15 ALCC16

________ ________ ________ ________ ________

ALCC12 0.261

ALCC13 0.300 0.821

ALCC14 0.339 0.448 1.136

ALCC15 0.379 0.521 0.664 1.741

ALCC16 0.418 0.595 0.772 0.949 1.911

ALCC17 0.321 0.499 0.677 0.854 1.032

ALCC18 0.224 0.403 0.581 0.760 0.938

ALCC19 0.194 0.373 0.551 0.730 0.909

ALCC20 0.188 0.367 0.545 0.724 0.903

Model Estimated Covariances

ALCC17 ALCC18 ALCC19 ALCC20

________ ________ ________ ________

ALCC17 2.821

ALCC18 1.536 3.611

ALCC19 1.615 2.322 3.359

ALCC20 1.631 2.359 2.587 3.423

Residuals for Covariances

ALCC12 ALCC13 ALCC14 ALCC15 ALCC16

________ ________ ________ ________ ________

ALCC12 -0.043

ALCC13 0.286 -0.154

ALCC14 0.740 0.889 -0.027

ALCC15 0.967 0.201 0.768 -0.208

ALCC16 0.498 0.387 1.186 0.906 0.015

ALCC17 0.266 1.635 0.200 0.019 1.405

ALCC18 0.063 0.431 0.651 1.068 1.504

ALCC19 -0.576 -0.074 -0.814 0.166 0.741

ALCC20 -1.303 -0.917 -1.306 -0.191 -0.047

Residuals for Covariances

ALCC17 ALCC18 ALCC19 ALCC20

________ ________ ________ ________

ALCC17 -0.205

ALCC18 1.266 -0.745

ALCC19 0.700 0.793 -0.668

ALCC20 -2.175 -0.049 1.150 -0.985

UNIVARIATE DISTRIBUTION FIT

Variable Observed Estimated Residual (Obs.-Est.) Stand. Residual

ALCD12

Category 1 0.960 0.926 0.035 2.507

Category 2 0.040 0.074 -0.035 -2.507

ALCD13

Category 1 0.877 0.886 -0.009 -0.545

Category 2 0.123 0.114 0.009 0.545

ALCD14

Category 1 0.788 0.819 -0.031 -1.545

Category 2 0.212 0.181 0.031 1.545

ALCD15

Category 1 0.652 0.680 -0.028 -1.134

Category 2 0.348 0.320 0.028 1.134

ALCD16

Category 1 0.580 0.594 -0.014 -0.532

Category 2 0.420 0.406 0.014 0.532

ALCD17

Category 1 0.491 0.466 0.025 0.959

Category 2 0.509 0.534 -0.025 -0.959

ALCD18

Category 1 0.364 0.356 0.008 0.309

Category 2 0.636 0.644 -0.008 -0.309

ALCD19

Category 1 0.272 0.272 0.000 0.000

Category 2 0.728 0.728 0.000 0.000

ALCD20

Category 1 0.246 0.259 -0.013 -0.579

Category 2 0.754 0.741 0.013 0.579

BIVARIATE DISTRIBUTIONS FIT

Variable Variable Observed Estimated Residual (Obs.-Est.) Stand. Residual

ALCD12 ALCD13

Category 1 Category 1 0.876 0.850 0.026 1.397

Category 1 Category 2 0.091 0.076 0.015 1.071

Category 2 Category 1 0.009 0.036 -0.027 -2.753

Category 2 Category 2 0.024 0.038 -0.014 -1.402

ALCD12 ALCD14

Category 1 Category 1 0.782 0.790 -0.008 -0.376

Category 1 Category 2 0.179 0.135 0.044 2.457

Category 2 Category 1 0.003 0.029 -0.026 -2.942

Category 2 Category 2 0.035 0.046 -0.010 -0.935

ALCD12 ALCD15

Category 1 Category 1 0.647 0.659 -0.012 -0.496

Category 1 Category 2 0.316 0.266 0.049 2.117

Category 2 Category 1 0.006 0.021 -0.015 -1.958

Category 2 Category 2 0.031 0.053 -0.022 -1.870

ALCD12 ALCD16

Category 1 Category 1 0.581 0.576 0.005 0.196

Category 1 Category 2 0.386 0.350 0.037 1.468

Category 2 Category 1 0.000 0.018 -0.018 -2.583

Category 2 Category 2 0.032 0.056 -0.024 -1.964

ALCD12 ALCD17

Category 1 Category 1 0.481 0.451 0.029 1.122

Category 1 Category 2 0.477 0.474 0.003 0.110

Category 2 Category 1 0.018 0.015 0.003 0.416

Category 2 Category 2 0.025 0.059 -0.035 -2.807

ALCD12 ALCD18

Category 1 Category 1 0.369 0.343 0.025 1.008

Category 1 Category 2 0.595 0.582 0.013 0.489

Category 2 Category 1 0.000 0.013 -0.013 -2.160

Category 2 Category 2 0.036 0.062 -0.025 -1.988

ALCD12 ALCD19

Category 1 Category 1 0.265 0.261 0.004 0.175

Category 1 Category 2 0.698 0.665 0.032 1.310

Category 2 Category 1 0.007 0.011 -0.004 -0.790

Category 2 Category 2 0.031 0.063 -0.032 -2.517

ALCD12 ALCD20

Category 1 Category 1 0.243 0.248 -0.005 -0.206

Category 1 Category 2 0.725 0.678 0.047 1.933

Category 2 Category 1 0.005 0.011 -0.007 -1.190

Category 2 Category 2 0.027 0.063 -0.036 -2.835

ALCD13 ALCD14

Category 1 Category 1 0.743 0.771 -0.028 -1.270

Category 1 Category 2 0.132 0.115 0.016 0.975

Category 2 Category 1 0.045 0.049 -0.004 -0.329

Category 2 Category 2 0.081 0.065 0.015 1.185

ALCD13 ALCD15

Category 1 Category 1 0.634 0.648 -0.014 -0.557

Category 1 Category 2 0.240 0.238 0.002 0.083

Category 2 Category 1 0.028 0.032 -0.004 -0.409

Category 2 Category 2 0.098 0.082 0.016 1.105

ALCD13 ALCD16

Category 1 Category 1 0.551 0.568 -0.017 -0.662

Category 1 Category 2 0.331 0.318 0.013 0.542

Category 2 Category 1 0.033 0.026 0.007 0.797

Category 2 Category 2 0.085 0.088 -0.003 -0.181

ALCD13 ALCD17

Category 1 Category 1 0.455 0.447 0.009 0.331

Category 1 Category 2 0.448 0.439 0.009 0.331

Category 2 Category 1 0.029 0.020 0.009 1.228

Category 2 Category 2 0.068 0.094 -0.026 -1.709

ALCD13 ALCD18

Category 1 Category 1 0.338 0.341 -0.002 -0.094

Category 1 Category 2 0.544 0.545 -0.001 -0.048

Category 2 Category 1 0.018 0.016 0.003 0.426

Category 2 Category 2 0.099 0.098 0.001 0.052

ALCD13 ALCD19

Category 1 Category 1 0.247 0.259 -0.011 -0.497

Category 1 Category 2 0.641 0.627 0.014 0.551

Category 2 Category 1 0.021 0.013 0.008 1.332

Category 2 Category 2 0.091 0.101 -0.010 -0.662

ALCD13 ALCD20

Category 1 Category 1 0.227 0.246 -0.019 -0.842

Category 1 Category 2 0.645 0.640 0.006 0.232

Category 2 Category 1 0.014 0.013 0.001 0.175

Category 2 Category 2 0.114 0.101 0.012 0.768

ALCD14 ALCD15

Category 1 Category 1 0.596 0.625 -0.029 -1.127

Category 1 Category 2 0.180 0.194 -0.014 -0.679

Category 2 Category 1 0.053 0.055 -0.002 -0.207

Category 2 Category 2 0.171 0.126 0.045 2.601

ALCD14 ALCD16

Category 1 Category 1 0.526 0.552 -0.026 -0.999

Category 1 Category 2 0.271 0.267 0.004 0.158

Category 2 Category 1 0.055 0.042 0.013 1.190

Category 2 Category 2 0.148 0.139 0.010 0.542

ALCD14 ALCD17

Category 1 Category 1 0.447 0.437 0.010 0.377

Category 1 Category 2 0.363 0.382 -0.019 -0.751

Category 2 Category 1 0.046 0.029 0.017 1.915

Category 2 Category 2 0.144 0.152 -0.008 -0.398

ALCD14 ALCD18

Category 1 Category 1 0.325 0.335 -0.010 -0.419

Category 1 Category 2 0.477 0.484 -0.007 -0.280

Category 2 Category 1 0.036 0.021 0.015 2.028

Category 2 Category 2 0.162 0.160 0.003 0.131

ALCD14 ALCD19

Category 1 Category 1 0.234 0.256 -0.022 -0.954

Category 1 Category 2 0.559 0.563 -0.004 -0.158

Category 2 Category 1 0.031 0.016 0.014 2.190

Category 2 Category 2 0.176 0.165 0.012 0.591

ALCD14 ALCD20

Category 1 Category 1 0.211 0.244 -0.033 -1.454

Category 1 Category 2 0.552 0.576 -0.024 -0.927

Category 2 Category 1 0.036 0.015 0.020 3.167

Category 2 Category 2 0.202 0.165 0.036 1.863

ALCD15 ALCD16

Category 1 Category 1 0.507 0.505 0.002 0.080

Category 1 Category 2 0.151 0.176 -0.025 -1.231

Category 2 Category 1 0.064 0.090 -0.026 -1.720

Category 2 Category 2 0.279 0.230 0.048 2.184

ALCD15 ALCD17

Category 1 Category 1 0.423 0.411 0.012 0.448

Category 1 Category 2 0.272 0.269 0.003 0.130

Category 2 Category 1 0.048 0.055 -0.007 -0.605

Category 2 Category 2 0.257 0.265 -0.007 -0.318

ALCD15 ALCD18

Category 1 Category 1 0.296 0.321 -0.025 -1.031

Category 1 Category 2 0.375 0.359 0.015 0.614

Category 2 Category 1 0.060 0.035 0.025 2.583

Category 2 Category 2 0.270 0.285 -0.015 -0.637

ALCD15 ALCD19

Category 1 Category 1 0.224 0.248 -0.024 -1.047

Category 1 Category 2 0.441 0.432 0.009 0.346

Category 2 Category 1 0.039 0.024 0.015 1.907

Category 2 Category 2 0.295 0.296 -0.001 -0.022

ALCD15 ALCD20

Category 1 Category 1 0.199 0.237 -0.037 -1.678

Category 1 Category 2 0.426 0.444 -0.018 -0.680

Category 2 Category 1 0.042 0.022 0.019 2.475

Category 2 Category 2 0.333 0.297 0.036 1.498

ALCD16 ALCD17

Category 1 Category 1 0.413 0.388 0.025 0.958

Category 1 Category 2 0.197 0.206 -0.009 -0.426

Category 2 Category 1 0.071 0.078 -0.008 -0.534

Category 2 Category 2 0.320 0.328 -0.008 -0.323

ALCD16 ALCD18

Category 1 Category 1 0.280 0.309 -0.029 -1.183

Category 1 Category 2 0.307 0.285 0.022 0.931

Category 2 Category 1 0.074 0.047 0.027 2.387

Category 2 Category 2 0.339 0.359 -0.020 -0.794

ALCD16 ALCD19

Category 1 Category 1 0.207 0.241 -0.034 -1.516

Category 1 Category 2 0.367 0.353 0.014 0.576

Category 2 Category 1 0.055 0.030 0.024 2.669

Category 2 Category 2 0.371 0.375 -0.004 -0.176

ALCD16 ALCD20

Category 1 Category 1 0.171 0.231 -0.060 -2.711

Category 1 Category 2 0.379 0.364 0.016 0.618

Category 2 Category 1 0.057 0.028 0.029 3.277

Category 2 Category 2 0.393 0.378 0.016 0.622

ALCD17 ALCD18

Category 1 Category 1 0.280 0.282 -0.002 -0.096

Category 1 Category 2 0.212 0.184 0.028 1.352

Category 2 Category 1 0.072 0.074 -0.002 -0.160

Category 2 Category 2 0.436 0.460 -0.023 -0.881

ALCD17 ALCD19

Category 1 Category 1 0.213 0.227 -0.013 -0.612

Category 1 Category 2 0.277 0.239 0.037 1.664

Category 2 Category 1 0.059 0.045 0.014 1.321

Category 2 Category 2 0.451 0.489 -0.038 -1.456

ALCD17 ALCD20

Category 1 Category 1 0.188 0.218 -0.030 -1.378

Category 1 Category 2 0.287 0.249 0.039 1.707

Category 2 Category 1 0.055 0.041 0.014 1.343

Category 2 Category 2 0.470 0.493 -0.023 -0.872

ALCD18 ALCD19

Category 1 Category 1 0.228 0.206 0.021 1.004

Category 1 Category 2 0.142 0.150 -0.008 -0.406

Category 2 Category 1 0.061 0.066 -0.005 -0.350

Category 2 Category 2 0.569 0.578 -0.009 -0.354

ALCD18 ALCD20

Category 1 Category 1 0.203 0.199 0.004 0.198

Category 1 Category 2 0.147 0.157 -0.010 -0.526

Category 2 Category 1 0.045 0.060 -0.015 -1.166

Category 2 Category 2 0.605 0.584 0.020 0.788

ALCD19 ALCD20

Category 1 Category 1 0.146 0.176 -0.030 -1.494

Category 1 Category 2 0.106 0.095 0.011 0.689

Category 2 Category 1 0.091 0.083 0.008 0.577

Category 2 Category 2 0.657 0.646 0.011 0.435

TECHNICAL 4 OUTPUT

ESTIMATES DERIVED FROM THE MODEL

ESTIMATED MEANS FOR THE LATENT VARIABLES

SD SC2 ID IC SC

________ ________ ________ ________ ________

0.977 1.033 -0.167 0.281 0.484

ESTIMATED MEANS FOR THE LATENT VARIABLES

GENDER C_IMB

________ ________

0.445 0.108

S.E. FOR ESTIMATED MEANS FOR THE LATENT VARIABLES

SD SC2 ID IC SC

________ ________ ________ ________ ________

0.073 0.072 0.177 0.226 0.086

S.E. FOR ESTIMATED MEANS FOR THE LATENT VARIABLES

GENDER C_IMB

________ ________

0.026 0.016

EST./S.E. FOR ESTIMATED MEANS FOR THE LATENT VARIABLES

SD SC2 ID IC SC

________ ________ ________ ________ ________

13.455 14.354 -0.939 1.241 5.628

EST./S.E. FOR ESTIMATED MEANS FOR THE LATENT VARIABLES

GENDER C_IMB

________ ________

17.028 6.611

TWO-TAILED P-VALUE FOR ESTIMATED MEANS FOR THE LATENT VARIABLES

SD SC2 ID IC SC

________ ________ ________ ________ ________

0.000 0.000 0.348 0.215 0.000

TWO-TAILED P-VALUE FOR ESTIMATED MEANS FOR THE LATENT VARIABLES

GENDER C_IMB

________ ________

0.000 0.000

ESTIMATED COVARIANCE MATRIX FOR THE LATENT VARIABLES

SD SC2 ID IC SC

________ ________ ________ ________ ________

SD 0.249

SC2 0.163 0.346

ID -0.392 -0.022 6.005

IC -0.001 -0.096 1.092 0.374

SC -0.001 0.001 0.171 0.074 0.035

GENDER 0.000 0.061 -0.076 -0.018 0.007

C_IMB 0.005 0.012 -0.036 -0.017 -0.010

ESTIMATED COVARIANCE MATRIX FOR THE LATENT VARIABLES

GENDER C_IMB

________ ________

GENDER 0.247

C_IMB 0.010 0.096

S.E. FOR ESTIMATED COVARIANCE MATRIX FOR THE LATENT VARIABLES

SD SC2 ID IC SC

________ ________ ________ ________ ________

SD 0.057

SC2 0.040 0.059

ID 0.218 0.028 1.177

IC 0.004 0.059 0.382 0.125

SC 0.002 0.004 0.117 0.017 0.012

GENDER 0.023 0.027 0.095 0.035 0.015

C_IMB 0.014 0.014 0.049 0.033 0.013

S.E. FOR ESTIMATED COVARIANCE MATRIX FOR THE LATENT VARIABLES

GENDER C_IMB

________ ________

GENDER 0.018

C_IMB 0.008 0.007

EST./S.E. FOR ESTIMATED COVARIANCE MATRIX FOR THE LATENT VARIABLES

SD SC2 ID IC SC

________ ________ ________ ________ ________

SD 4.363

SC2 4.111 5.907

ID -1.797 -0.786 5.100

IC -0.210 -1.620 2.857 2.994

SC -0.320 0.159 1.465 4.233 2.827

GENDER -0.005 2.271 -0.797 -0.519 0.465

C_IMB 0.338 0.831 -0.722 -0.517 -0.811

EST./S.E. FOR ESTIMATED COVARIANCE MATRIX FOR THE LATENT VARIABLES

GENDER C_IMB

________ ________

GENDER 13.454

C_IMB 1.244 13.454

TWO-TAILED P-VALUE FOR ESTIMATED COVARIANCE MATRIX FOR THE LATENT VARIABLES

SD SC2 ID IC SC

________ ________ ________ ________ ________

SD 0.000

SC2 0.000 0.000

ID 0.072 0.432 0.000

IC 0.834 0.105 0.004 0.003

SC 0.749 0.873 0.143 0.000 0.005

GENDER 0.996 0.023 0.426 0.604 0.642

C_IMB 0.735 0.406 0.470 0.605 0.417

TWO-TAILED P-VALUE FOR ESTIMATED COVARIANCE MATRIX FOR THE LATENT VARIABLES

GENDER C_IMB

________ ________

GENDER 0.000

C_IMB 0.213 0.000

ESTIMATED CORRELATION MATRIX FOR THE LATENT VARIABLES

SD SC2 ID IC SC

________ ________ ________ ________ ________

SD 1.000

SC2 0.554 1.000

ID -0.321 -0.015 1.000

IC -0.003 -0.267 0.729 1.000

SC -0.006 0.006 0.376 0.648 1.000

GENDER 0.000 0.210 -0.062 -0.060 0.074

C_IMB 0.031 0.065 -0.047 -0.089 -0.180

ESTIMATED CORRELATION MATRIX FOR THE LATENT VARIABLES

GENDER C_IMB

________ ________

GENDER 1.000

C_IMB 0.066 1.000

S.E. FOR ESTIMATED CORRELATION MATRIX FOR THE LATENT VARIABLES

SD SC2 ID IC SC

________ ________ ________ ________ ________

SD 0.000

SC2 0.129 0.000

ID 0.133 0.019 0.000

IC 0.012 0.171 0.178 0.000

SC 0.018 0.038 0.208 0.161 0.000

GENDER 0.091 0.087 0.077 0.114 0.160

C_IMB 0.091 0.078 0.064 0.171 0.218

S.E. FOR ESTIMATED CORRELATION MATRIX FOR THE LATENT VARIABLES

GENDER C_IMB

________ ________

GENDER 0.000

C_IMB 0.052 0.000

EST./S.E. FOR ESTIMATED CORRELATION MATRIX FOR THE LATENT VARIABLES

SD SC2 ID IC SC

________ ________ ________ ________ ________

SD 999.000

SC2 4.284 999.000

ID -2.405 -0.803 999.000

IC -0.211 -1.566 4.103 999.000

SC -0.321 0.159 1.805 4.025 999.000

GENDER -0.005 2.418 -0.807 -0.528 0.464

C_IMB 0.339 0.832 -0.726 -0.524 -0.826

EST./S.E. FOR ESTIMATED CORRELATION MATRIX FOR THE LATENT VARIABLES

GENDER C_IMB

________ ________

GENDER 999.000

C_IMB 1.252 999.000

TWO-TAILED P-VALUE FOR ESTIMATED CORRELATION MATRIX FOR THE LATENT VARIABLES

SD SC2 ID IC SC

________ ________ ________ ________ ________

SD 0.000

SC2 0.000 0.000

ID 0.016 0.422 0.000

IC 0.833 0.117 0.000 0.000

SC 0.748 0.874 0.071 0.000 0.000

GENDER 0.996 0.016 0.420 0.597 0.643

C_IMB 0.734 0.406 0.468 0.601 0.409

TWO-TAILED P-VALUE FOR ESTIMATED CORRELATION MATRIX FOR THE LATENT VARIABLES

GENDER C_IMB

________ ________

GENDER 0.000

C_IMB 0.211 0.000

DIAGRAM INFORMATION

Use View Diagram under the Diagram menu in the Mplus Editor to view the diagram.

If running Mplus from the Mplus Diagrammer, the diagram opens automatically.

Diagram output

u:\windows\dcn r&r\r&r analyses\prediction model\revised gmm umsv predicting au two-part.dgm

Beginning Time: 14:33:28

Ending Time: 14:33:35

Elapsed Time: 00:00:07

MUTHEN & MUTHEN

3463 Stoner Ave.

Los Angeles, CA 90066

Tel: (310) 391-9971

Fax: (310) 391-8971

Web: www.StatModel.com

Support: Support@StatModel.com

Copyright (c) 1998-2018 Muthen & Muthen
