# Supplementary material for: Mind the gap: A review and recommendations for statistically evaluating Dual Systems models of adolescent risk behavior
Source: Dev Cogn Neurosci. 2019 Jul 25;39:100681. doi: 10.1016/j.dcn.2019.100681 (PMC6969358; doi:10.1016/j.dcn.2019.100681)
Supplement: Supplementary file 6 [file mmc6.docx]

Mplus VERSION 8.2

MUTHEN & MUTHEN

04/05/2019 4:03 PM

INPUT INSTRUCTIONS

Title: GMM Predicting Marijuana Two-Part Model

Data: file is predictR&R2.dat;

Variable:

NAMES ARE

subject gender mard12-mard20 marc12-marc20 c_imb;

!For the class variable 1 (c_imb) is the class with the larger imbalance

!and 0 is the class with the smaller imbalance

USEV

mard12-mard20 marc12-marc20 c_imb gender;

missing are .;

CATEGORICAL ARE mard13-mard20;

Define:

marc13=log(marc13+1);

marc14=log(marc14+1);

marc15=log(marc15+1);

marc16=log(marc16+1);

marc17=log(marc17+1);

marc18=log(marc18+1);

marc19=log(marc19+1);

marc20=log(marc20+1);

Analysis:

estimator is MLR;

model=nocovariances;

mconvergence=.01;

Model:

!Marijuana Two-Part Growth Model With Random Effects

!Dichotomous Growth Model ages 13-20

id sd | mard13@0 mard14@1 mard15@2 mard16@3 mard17@4 mard18*

mard19@6 mard20*;

sd BY mard18*5.74545;

sd BY mard20*5.98383;

!Continuous Growth Model ages 13-20

ic sc | marc13@0 marc14@1 marc15@2 marc16* marc17@4

marc18* marc19@6 marc20*;

sc BY marc16*2.42648;

sc BY marc18*5.38491;

sc BY marc20*6.28684;

!Covariances Between Growth Factors

id WITH sd*-0.87695;

id WITH ic*1.93801;

id WITH sc@0;

ic WITH sc@0;

sd WITH ic*-0.22456;

sd WITH sc*0.12190;

!Continuous Marijuana Use Intercepts Constrained to 0 for Model

!Identification

[ marc13@0 ];

[ marc14@0 ];

[ marc15@0 ];

[ marc16@0 ];

[ marc17@0 ];

[ marc18@0 ];

[ marc19@0 ];

[ marc20@0 ];

[ id@0 ];

!Growth Factor Means Start Values

[ sd*0.86006 ];

[ ic*0.31980 ];

[ sc*0.33860 ];

!Dichotomous Marijuana Use Thresholds Start Values

[ mard13$1*5.18694 ] (25);

[ mard14$1*5.18694 ] (25);

[ mard15$1*5.18694 ] (25);

[ mard16$1*5.18694 ] (25);

[ mard17$1*5.18694 ] (25);

[ mard18$1*5.18694 ] (25);

[ mard19$1*5.18694 ] (25);

[ mard20$1*5.18694 ] (25);

!Start Values for Variances of Continous Marijuana Use and

!Marijuana Use Growth Factors

marc13*0.43001;

marc14*0.33564;

marc15*0.94733;

marc16*0.98942;

marc17*1.45900;

marc18*1.11107;

marc19*0.94213;

marc20*1.31857;

sc*0.09352;

id*7.00211;

sd*0.38070;

ic*0.82317;

!class Membership Predicting Growth in Marijuana Use

id sd ic sc on c_imb;

!Controlling for Gender

id sd ic sc on gender;

Output: res stdyx tech4 sampstat mod(5) svalues;

*** WARNING in OUTPUT command

MODINDICES option is not available for ALGORITHM=INTEGRATION.

Request for MODINDICES is ignored.

*** WARNING

Data set contains cases with missing on x-variables.

These cases were not included in the analysis.

Number of cases with missing on x-variables: 25

*** WARNING

Data set contains cases with missing on all variables except

x-variables. These cases were not included in the analysis.

Number of cases with missing on all variables except x-variables: 3

3 WARNING(S) FOUND IN THE INPUT INSTRUCTIONS

Revised Classes Predicting Alcohol 2-Part DCN R&R

SUMMARY OF ANALYSIS

Number of groups 1

Number of observations 359

Number of dependent variables 16

Number of independent variables 2

Number of continuous latent variables 4

Observed dependent variables

Continuous

MARC13 MARC14 MARC15 MARC16 MARC17 MARC18

MARC19 MARC20

Binary and ordered categorical (ordinal)

MARD13 MARD14 MARD15 MARD16 MARD17 MARD18

MARD19 MARD20

Observed independent variables

GENDER C_IMB

Continuous latent variables

SD SC ID IC

Estimator MLR

Information matrix OBSERVED

Optimization Specifications for the Quasi-Newton Algorithm for

Continuous Outcomes

Maximum number of iterations 100

Convergence criterion 0.100D-05

Optimization Specifications for the EM Algorithm

Maximum number of iterations 500

Convergence criteria

Loglikelihood change 0.100D-02

Relative loglikelihood change 0.100D-05

Derivative 0.100D-01

Optimization Specifications for the M step of the EM Algorithm for

Categorical Latent variables

Number of M step iterations 1

M step convergence criterion 0.100D-02

Basis for M step termination ITERATION

Optimization Specifications for the M step of the EM Algorithm for

Censored, Binary or Ordered Categorical (Ordinal), Unordered

Categorical (Nominal) and Count Outcomes

Number of M step iterations 1

M step convergence criterion 0.100D-02

Basis for M step termination ITERATION

Maximum value for logit thresholds 15

Minimum value for logit thresholds -15

Minimum expected cell size for chi-square 0.100D-01

Maximum number of iterations for H1 2000

Convergence criterion for H1 0.100D-03

Optimization algorithm EMA

Integration Specifications

Type STANDARD

Number of integration points 15

Dimensions of numerical integration 2

Adaptive quadrature ON

Link LOGIT

Cholesky OFF

Input data file(s)

predictR&R.dat

Input data format FREE

SUMMARY OF DATA

Number of missing data patterns 158

Number of y missing data patterns 70

Number of u missing data patterns 55

COVARIANCE COVERAGE OF DATA

Minimum covariance coverage value 0.100

PROPORTION OF DATA PRESENT

Covariance Coverage

MARD13 MARD14 MARD15 MARD16 MARD17

________ ________ ________ ________ ________

MARD13 0.955

MARD14 0.936 0.975

MARD15 0.877 0.889 0.903

MARD16 0.852 0.864 0.822 0.880

MARD17 0.777 0.791 0.747 0.735 0.808

MARD18 0.766 0.780 0.741 0.721 0.660

MARD19 0.794 0.811 0.772 0.758 0.696

MARD20 0.607 0.618 0.593 0.579 0.501

MARC13 0.025 0.025 0.017 0.019 0.017

MARC14 0.081 0.086 0.078 0.064 0.050

MARC15 0.109 0.114 0.114 0.089 0.078

MARC16 0.145 0.145 0.136 0.148 0.111

MARC17 0.231 0.231 0.228 0.214 0.237

MARC18 0.368 0.370 0.359 0.343 0.295

MARC19 0.370 0.379 0.362 0.345 0.320

MARC20 0.312 0.309 0.298 0.290 0.253

GENDER 0.955 0.975 0.903 0.880 0.808

C_IMB 0.955 0.975 0.903 0.880 0.808

Covariance Coverage

MARD18 MARD19 MARD20 MARC13 MARC14

________ ________ ________ ________ ________

MARD18 0.794

MARD19 0.685 0.819

MARD20 0.493 0.543 0.632

MARC13 0.025 0.019 0.011 0.025

MARC14 0.061 0.056 0.058 0.014 0.086

MARC15 0.089 0.086 0.072 0.011 0.050

MARC16 0.125 0.131 0.100 0.014 0.031

MARC17 0.198 0.209 0.136 0.011 0.031

MARC18 0.376 0.323 0.251 0.022 0.045

MARC19 0.315 0.382 0.279 0.017 0.033

MARC20 0.253 0.276 0.318 0.011 0.033

GENDER 0.794 0.819 0.632 0.025 0.086

C_IMB 0.794 0.819 0.632 0.025 0.086

Covariance Coverage

MARC15 MARC16 MARC17 MARC18 MARC19

________ ________ ________ ________ ________

MARC15 0.114

MARC16 0.047 0.148

MARC17 0.053 0.086 0.237

MARC18 0.078 0.106 0.156 0.376

MARC19 0.058 0.103 0.148 0.259 0.382

MARC20 0.045 0.072 0.095 0.189 0.217

GENDER 0.114 0.148 0.237 0.376 0.382

C_IMB 0.114 0.148 0.237 0.376 0.382

Covariance Coverage

MARC20 GENDER C_IMB

________ ________ ________

MARC20 0.318

GENDER 0.318 1.000

C_IMB 0.318 1.000 1.000

WARNING: THE COVARIANCE COVERAGE FALLS BELOW THE SPECIFIED LIMIT.

PROPORTION OF DATA PRESENT FOR U

Covariance Coverage

MARD13 MARD14 MARD15 MARD16 MARD17

________ ________ ________ ________ ________

MARD13 0.955

MARD14 0.936 0.975

MARD15 0.877 0.889 0.903

MARD16 0.852 0.864 0.822 0.880

MARD17 0.777 0.791 0.747 0.735 0.808

MARD18 0.766 0.780 0.741 0.721 0.660

MARD19 0.794 0.811 0.772 0.758 0.696

MARD20 0.607 0.618 0.593 0.579 0.501

Covariance Coverage

MARD18 MARD19 MARD20

________ ________ ________

MARD18 0.794

MARD19 0.685 0.819

MARD20 0.493 0.543 0.632

PROPORTION OF DATA PRESENT FOR Y

Covariance Coverage

MARC13 MARC14 MARC15 MARC16 MARC17

________ ________ ________ ________ ________

MARC13 0.025

MARC14 0.014 0.086

MARC15 0.011 0.050 0.114

MARC16 0.014 0.031 0.047 0.148

MARC17 0.011 0.031 0.053 0.086 0.237

MARC18 0.022 0.045 0.078 0.106 0.156

MARC19 0.017 0.033 0.058 0.103 0.148

MARC20 0.011 0.033 0.045 0.072 0.095

GENDER 0.025 0.086 0.114 0.148 0.237

C_IMB 0.025 0.086 0.114 0.148 0.237

Covariance Coverage

MARC18 MARC19 MARC20 GENDER C_IMB

________ ________ ________ ________ ________

MARC18 0.376

MARC19 0.259 0.382

MARC20 0.189 0.217 0.318

GENDER 0.376 0.382 0.318 1.000

C_IMB 0.376 0.382 0.318 1.000 1.000

THE COVARIANCE COVERAGE FALLS BELOW THE SPECIFIED LIMIT. THE MISSING

DATA EM ALGORITHM WILL NOT BE INITIATED. CHECK YOUR DATA OR LOWER THE

COVARIANCE COVERAGE LIMIT.

UNIVARIATE PROPORTIONS AND COUNTS FOR CATEGORICAL VARIABLES

MARD13

Category 1 0.974 334.000

Category 2 0.026 9.000

MARD14

Category 1 0.911 319.000

Category 2 0.089 31.000

MARD15

Category 1 0.873 283.000

Category 2 0.127 41.000

MARD16

Category 1 0.832 263.000

Category 2 0.168 53.000

MARD17

Category 1 0.707 205.000

Category 2 0.293 85.000

MARD18

Category 1 0.526 150.000

Category 2 0.474 135.000

MARD19

Category 1 0.534 157.000

Category 2 0.466 137.000

MARD20

Category 1 0.498 113.000

Category 2 0.502 114.000

SAMPLE STATISTICS

UNIVARIATE SAMPLE STATISTICS

UNIVARIATE HIGHER-ORDER MOMENT DESCRIPTIVE STATISTICS

Variable/ Mean/ Skewness/ Minimum/ % with Percentiles

Sample Size Variance Kurtosis Maximum Min/Max 20%/60% 40%/80% Median

MARC13 1.533 -0.214 0.693 22.22% 0.693 1.386 1.609

9.000 0.298 -0.906 2.398 11.11% 1.609 1.946

MARC14 1.767 0.392 0.693 22.58% 0.693 1.099 1.386

31.000 0.854 -1.274 3.434 6.45% 2.079 2.773

MARC15 2.158 0.933 0.693 24.39% 0.693 1.386 1.609

41.000 2.129 -0.235 5.707 2.44% 2.197 3.434

MARC16 2.392 0.906 0.693 11.32% 1.386 1.946 2.197

53.000 1.787 0.173 5.903 1.89% 2.398 3.045

MARC17 2.796 0.470 0.693 9.41% 0.916 1.792 2.398

85.000 3.300 -1.244 5.889 8.24% 2.773 4.875

MARC18 3.178 0.013 0.693 1.48% 0.916 2.565 3.434

135.000 3.827 -1.590 5.889 13.33% 3.970 5.460

MARC19 3.351 -0.098 0.916 35.04% 0.916 2.565 3.434

137.000 4.061 -1.650 5.889 16.79% 4.875 5.460

MARC20 3.565 -0.234 0.916 28.95% 0.916 3.434 3.434

114.000 3.903 -1.542 5.889 19.30% 4.875 5.460

GENDER 0.448 0.207 0.000 55.15% 0.000 0.000 0.000

359.000 0.247 -1.957 1.000 44.85% 1.000 1.000

C_IMB 0.106 2.562 0.000 89.42% 0.000 0.000 0.000

359.000 0.095 4.566 1.000 10.58% 0.000 0.000

THE MODEL ESTIMATION TERMINATED NORMALLY

MODEL FIT INFORMATION

Number of Free Parameters 33

Loglikelihood

H0 Value -1997.752

H0 Scaling Correction Factor 0.9823

for MLR

Information Criteria

Akaike (AIC) 4061.504

Bayesian (BIC) 4189.653

Sample-Size Adjusted BIC 4084.961

(n* = (n + 2) / 24)

MODEL RESULTS

Two-Tailed

Estimate S.E. Est./S.E. P-Value

ID |

MARD13 1.000 0.000 999.000 999.000

MARD14 1.000 0.000 999.000 999.000

MARD15 1.000 0.000 999.000 999.000

MARD16 1.000 0.000 999.000 999.000

MARD17 1.000 0.000 999.000 999.000

MARD18 1.000 0.000 999.000 999.000

MARD19 1.000 0.000 999.000 999.000

MARD20 1.000 0.000 999.000 999.000

SD |

MARD13 0.000 0.000 999.000 999.000

MARD14 1.000 0.000 999.000 999.000

MARD15 2.000 0.000 999.000 999.000

MARD16 3.000 0.000 999.000 999.000

MARD17 4.000 0.000 999.000 999.000

MARD18 5.720 0.221 25.934 0.000

MARD19 6.000 0.000 999.000 999.000

MARD20 5.932 0.287 20.692 0.000

IC |

MARC13 1.000 0.000 999.000 999.000

MARC14 1.000 0.000 999.000 999.000

MARC15 1.000 0.000 999.000 999.000

MARC16 1.000 0.000 999.000 999.000

MARC17 1.000 0.000 999.000 999.000

MARC18 1.000 0.000 999.000 999.000

MARC19 1.000 0.000 999.000 999.000

MARC20 1.000 0.000 999.000 999.000

SC |

MARC13 0.000 0.000 999.000 999.000

MARC14 1.000 0.000 999.000 999.000

MARC15 2.000 0.000 999.000 999.000

MARC16 2.451 0.269 9.103 0.000

MARC17 4.000 0.000 999.000 999.000

MARC18 5.336 0.196 27.204 0.000

MARC19 6.000 0.000 999.000 999.000

MARC20 6.294 0.238 26.466 0.000

ID ON

C_IMB -1.621 1.016 -1.596 0.110

GENDER 0.316 0.489 0.647 0.517

SD ON

C_IMB 0.298 0.211 1.411 0.158

GENDER 0.090 0.111 0.812 0.417

IC ON

C_IMB -0.245 0.561 -0.437 0.662

GENDER -0.365 0.269 -1.357 0.175

SC ON

C_IMB -0.031 0.107 -0.293 0.769

GENDER 0.254 0.062 4.102 0.000

ID WITH

SD -0.850 0.269 -3.154 0.002

IC 1.975 0.518 3.814 0.000

SC 0.000 0.000 999.000 999.000

IC WITH

SC 0.000 0.000 999.000 999.000

SD -0.203 0.091 -2.244 0.025

SD WITH

SC 0.101 0.028 3.676 0.000

Intercepts

MARC13 0.000 0.000 999.000 999.000

MARC14 0.000 0.000 999.000 999.000

MARC15 0.000 0.000 999.000 999.000

MARC16 0.000 0.000 999.000 999.000

MARC17 0.000 0.000 999.000 999.000

MARC18 0.000 0.000 999.000 999.000

MARC19 0.000 0.000 999.000 999.000

MARC20 0.000 0.000 999.000 999.000

SD 0.795 0.102 7.806 0.000

SC 0.250 0.059 4.252 0.000

ID 0.000 0.000 999.000 999.000

IC 0.465 0.261 1.781 0.075

Thresholds

MARD13$1 5.187 0.505 10.275 0.000

MARD14$1 5.187 0.505 10.275 0.000

MARD15$1 5.187 0.505 10.275 0.000

MARD16$1 5.187 0.505 10.275 0.000

MARD17$1 5.187 0.505 10.275 0.000

MARD18$1 5.187 0.505 10.275 0.000

MARD19$1 5.187 0.505 10.275 0.000

MARD20$1 5.187 0.505 10.275 0.000

Residual Variances

MARC13 0.554 0.539 1.028 0.304

MARC14 0.268 0.162 1.658 0.097

MARC15 0.895 0.251 3.570 0.000

MARC16 0.962 0.295 3.262 0.001

MARC17 1.496 0.229 6.542 0.000

MARC18 1.160 0.246 4.717 0.000

MARC19 0.926 0.218 4.248 0.000

MARC20 1.276 0.312 4.087 0.000

SD 0.368 0.079 4.644 0.000

SC 0.076 0.015 4.972 0.000

ID 6.953 1.663 4.181 0.000

IC 0.900 0.345 2.607 0.009

STANDARDIZED MODEL RESULTS

STDYX Standardization

Two-Tailed

Estimate S.E. Est./S.E. P-Value

ID |

MARD13 0.829 0.031 26.658 0.000

MARD14 0.890 0.045 19.798 0.000

MARD15 0.923 0.064 14.490 0.000

MARD16 0.917 0.081 11.342 0.000

MARD17 0.874 0.091 9.562 0.000

MARD18 0.753 0.097 7.757 0.000

MARD19 0.732 0.091 8.018 0.000

MARD20 0.737 0.093 7.957 0.000

SD |

MARD13 0.000 0.000 999.000 999.000

MARD14 0.204 0.021 9.668 0.000

MARD15 0.423 0.045 9.496 0.000

MARD16 0.630 0.064 9.774 0.000

MARD17 0.801 0.076 10.470 0.000

MARD18 0.987 0.077 12.773 0.000

MARD19 1.006 0.079 12.786 0.000

MARD20 1.002 0.078 12.781 0.000

IC |

MARC13 0.793 0.118 6.722 0.000

MARC14 0.866 0.095 9.083 0.000

MARC15 0.667 0.099 6.735 0.000

MARC16 0.634 0.102 6.206 0.000

MARC17 0.503 0.089 5.619 0.000

MARC18 0.459 0.087 5.249 0.000

MARC19 0.438 0.086 5.116 0.000

MARC20 0.411 0.081 5.063 0.000

SC |

MARC13 0.000 0.000 999.000 999.000

MARC14 0.271 0.045 6.044 0.000

MARC15 0.417 0.056 7.397 0.000

MARC16 0.485 0.067 7.191 0.000

MARC17 0.628 0.056 11.214 0.000

MARC18 0.765 0.060 12.652 0.000

MARC19 0.821 0.058 14.125 0.000

MARC20 0.808 0.054 14.992 0.000

ID ON

C_IMB -0.186 0.113 -1.645 0.100

GENDER 0.059 0.089 0.656 0.512

SD ON

C_IMB 0.149 0.103 1.448 0.148

GENDER 0.073 0.089 0.819 0.413

IC ON

C_IMB -0.078 0.172 -0.452 0.651

GENDER -0.187 0.133 -1.412 0.158

SC ON

C_IMB -0.032 0.108 -0.295 0.768

GENDER 0.416 0.096 4.344 0.000

ID WITH

SD -0.531 0.084 -6.333 0.000

IC 0.789 0.106 7.461 0.000

SC 0.000 0.000 999.000 999.000

IC WITH

SC 0.000 0.000 999.000 999.000

SD -0.353 0.165 -2.144 0.032

SD WITH

SC 0.607 0.116 5.245 0.000

Intercepts

MARC13 0.000 0.000 999.000 999.000

MARC14 0.000 0.000 999.000 999.000

MARC15 0.000 0.000 999.000 999.000

MARC16 0.000 0.000 999.000 999.000

MARC17 0.000 0.000 999.000 999.000

MARC18 0.000 0.000 999.000 999.000

MARC19 0.000 0.000 999.000 999.000

MARC20 0.000 0.000 999.000 999.000

SD 1.292 0.187 6.914 0.000

SC 0.825 0.242 3.411 0.001

ID 0.000 0.000 999.000 999.000

IC 0.480 0.312 1.536 0.124

Thresholds

MARD13$1 1.600 0.107 14.890 0.000

MARD14$1 1.719 0.113 15.213 0.000

MARD15$1 1.783 0.124 14.389 0.000

MARD16$1 1.771 0.139 12.765 0.000

MARD17$1 1.687 0.151 11.178 0.000

MARD18$1 1.454 0.162 8.967 0.000

MARD19$1 1.413 0.153 9.255 0.000

MARD20$1 1.423 0.155 9.187 0.000

Residual Variances

MARC13 0.371 0.187 1.979 0.048

MARC14 0.214 0.147 1.454 0.146

MARC15 0.424 0.103 4.101 0.000

MARC16 0.410 0.090 4.583 0.000

MARC17 0.402 0.051 7.882 0.000

MARC18 0.259 0.056 4.638 0.000

MARC19 0.189 0.046 4.078 0.000

MARC20 0.229 0.052 4.389 0.000

SD 0.971 0.034 28.540 0.000

SC 0.828 0.079 10.445 0.000

ID 0.964 0.041 23.681 0.000

IC 0.957 0.056 17.111 0.000

R-SQUARE

Observed Two-Tailed

Variable Estimate S.E. Est./S.E. P-Value

MARD13 0.687 0.052 13.329 0.000

MARD14 0.639 0.053 12.150 0.000

MARD15 0.611 0.049 12.595 0.000

MARD16 0.617 0.042 14.556 0.000

MARD17 0.652 0.038 16.991 0.000

MARD18 0.741 0.036 20.627 0.000

MARD19 0.756 0.034 22.477 0.000

MARD20 0.752 0.036 21.029 0.000

MARC13 0.629 0.187 3.361 0.001

MARC14 0.786 0.147 5.354 0.000

MARC15 0.576 0.103 5.577 0.000

MARC16 0.590 0.090 6.583 0.000

MARC17 0.598 0.051 11.744 0.000

MARC18 0.741 0.056 13.248 0.000

MARC19 0.811 0.046 17.500 0.000

MARC20 0.771 0.052 14.765 0.000

Latent Two-Tailed

Variable Estimate S.E. Est./S.E. P-Value

SD 0.029 0.034 0.854 0.393

SC 0.172 0.079 2.172 0.030

ID 0.036 0.041 0.893 0.372

IC 0.043 0.056 0.773 0.440

QUALITY OF NUMERICAL RESULTS

Condition Number for the Information Matrix 0.308E-04

(ratio of smallest to largest eigenvalue)

MODEL COMMAND WITH FINAL ESTIMATES USED AS STARTING VALUES

id sd | mard13@0 mard14@1 mard15@2 mard16@3 mard17@4 mard18*

mard19@6 mard20*;

ic sc | marc13@0 marc14@1 marc15@2 marc16* marc17@4

marc18* marc19@6 marc20*;

sd BY mard18*5.72000;

sd BY mard20*5.93242;

sc BY marc16*2.45128;

sc BY marc18*5.33641;

sc BY marc20*6.29398;

id ON c_imb*-1.62101;

id ON gender*0.31630;

sd ON c_imb*0.29820;

sd ON gender*0.08979;

ic ON c_imb*-0.24532;

ic ON gender*-0.36510;

sc ON c_imb*-0.03142;

sc ON gender*0.25350;

id WITH sd*-0.84962;

id WITH ic*1.97504;

id WITH sc@0;

ic WITH sc@0;

ic WITH sd*-0.20314;

sd WITH sc*0.10145;

[ marc13@0 ];

[ marc14@0 ];

[ marc15@0 ];

[ marc16@0 ];

[ marc17@0 ];

[ marc18@0 ];

[ marc19@0 ];

[ marc20@0 ];

[ sd*0.79477 ];

[ sc*0.25019 ];

[ id@0 ];

[ ic*0.46513 ];

[ mard13$1*5.18679 ] (33);

[ mard14$1*5.18679 ] (33);

[ mard15$1*5.18679 ] (33);

[ mard16$1*5.18679 ] (33);

[ mard17$1*5.18679 ] (33);

[ mard18$1*5.18679 ] (33);

[ mard19$1*5.18679 ] (33);

[ mard20$1*5.18679 ] (33);

marc13*0.55381;

marc14*0.26807;

marc15*0.89509;

marc16*0.96167;

marc17*1.49573;

marc18*1.16022;

marc19*0.92630;

marc20*1.27620;

sd*0.36761;

sc*0.07605;

id*6.95314;

ic*0.90010;

RESIDUAL OUTPUT

ESTIMATED MODEL AND RESIDUALS (OBSERVED - ESTIMATED)

Model Estimated Means

MARC13 MARC14 MARC15 MARC16 MARC17

________ ________ ________ ________ ________

0.275 0.636 0.997 1.159 1.718

Model Estimated Means

MARC18 MARC19 MARC20

________ ________ ________

2.199 2.439 2.545

Residuals for Means

MARC13 MARC14 MARC15 MARC16 MARC17

________ ________ ________ ________ ________

1.258 1.131 1.162 1.233 1.078

Residuals for Means

MARC18 MARC19 MARC20

________ ________ ________

0.978 0.913 1.020

Model Estimated Covariances

MARC13 MARC14 MARC15 MARC16 MARC17

________ ________ ________ ________ ________

MARC13 1.495

MARC14 0.918 1.255

MARC15 0.895 1.056 2.112

MARC16 0.885 1.087 1.290 2.343

MARC17 0.850 1.195 1.539 1.695 3.724

MARC18 0.819 1.287 1.754 1.965 2.689

MARC19 0.804 1.333 1.861 2.100 2.918

MARC20 0.798 1.353 1.909 2.159 3.019

Model Estimated Covariances

MARC18 MARC19 MARC20

________ ________ ________

MARC18 4.474

MARC19 3.624 4.901

MARC20 3.762 4.130 5.570

Residuals for Covariances

MARC13 MARC14 MARC15 MARC16 MARC17

________ ________ ________ ________ ________

MARC13 -1.196

MARC14 -0.154 -0.402

MARC15 -1.117 0.844 0.017

MARC16 -0.413 1.478 3.487 -0.556

MARC17 3.988 2.397 3.255 2.413 -0.425

MARC18 1.299 1.910 2.185 1.984 2.726

MARC19 0.468 3.135 2.030 1.379 1.014

MARC20 0.901 -0.813 1.907 0.900 -0.265

Residuals for Covariances

MARC18 MARC19 MARC20

________ ________ ________

MARC18 -0.647

MARC19 0.962 -0.840

MARC20 0.447 -0.009 -1.666

UNIVARIATE DISTRIBUTION FIT

Variable Observed Estimated Residual (Obs.-Est.) Stand. Residual

MARD13

Category 1 0.974 0.947 0.027 2.267

Category 2 0.026 0.053 -0.027 -2.267

MARD14

Category 1 0.911 0.927 -0.015 -1.105

Category 2 0.089 0.073 0.015 1.105

MARD15

Category 1 0.873 0.887 -0.013 -0.804

Category 2 0.127 0.113 0.013 0.804

MARD16

Category 1 0.832 0.817 0.015 0.728

Category 2 0.168 0.183 -0.015 -0.728

MARD17

Category 1 0.707 0.718 -0.011 -0.469

Category 2 0.293 0.282 0.011 0.469

MARD18

Category 1 0.526 0.529 -0.003 -0.112

Category 2 0.474 0.471 0.003 0.112

MARD19

Category 1 0.534 0.502 0.032 1.218

Category 2 0.466 0.498 -0.032 -1.218

MARD20

Category 1 0.498 0.508 -0.011 -0.400

Category 2 0.502 0.492 0.011 0.400

BIVARIATE DISTRIBUTIONS FIT

Variable Variable Observed Estimated Residual (Obs.-Est.) Stand. Residual

MARD13 MARD14

Category 1 Category 1 0.902 0.896 0.006 0.346

Category 1 Category 2 0.071 0.051 0.021 1.788

Category 2 Category 1 0.012 0.030 -0.019 -2.043

Category 2 Category 2 0.015 0.023 -0.008 -0.989

MARD13 MARD15

Category 1 Category 1 0.870 0.859 0.011 0.576

Category 1 Category 2 0.111 0.088 0.023 1.571

Category 2 Category 1 0.006 0.028 -0.021 -2.459

Category 2 Category 2 0.013 0.025 -0.013 -1.533

MARD13 MARD16

Category 1 Category 1 0.824 0.792 0.031 1.451

Category 1 Category 2 0.154 0.154 -0.001 -0.045

Category 2 Category 1 0.007 0.025 -0.018 -2.238

Category 2 Category 2 0.016 0.028 -0.012 -1.348

MARD13 MARD17

Category 1 Category 1 0.695 0.695 0.000 0.003

Category 1 Category 2 0.283 0.252 0.031 1.375

Category 2 Category 1 0.007 0.023 -0.016 -1.981

Category 2 Category 2 0.014 0.030 -0.016 -1.764

MARD13 MARD18

Category 1 Category 1 0.516 0.509 0.007 0.275

Category 1 Category 2 0.451 0.438 0.013 0.500

Category 2 Category 1 0.004 0.020 -0.017 -2.228

Category 2 Category 2 0.029 0.033 -0.004 -0.405

MARD13 MARD19

Category 1 Category 1 0.530 0.482 0.048 1.813

Category 1 Category 2 0.446 0.465 -0.019 -0.733

Category 2 Category 1 0.004 0.020 -0.016 -2.220

Category 2 Category 2 0.021 0.033 -0.012 -1.285

MARD13 MARD20

Category 1 Category 1 0.486 0.488 -0.002 -0.083

Category 1 Category 2 0.495 0.459 0.037 1.403

Category 2 Category 1 0.000 0.020 -0.020 -2.702

Category 2 Category 2 0.018 0.033 -0.015 -1.565

MARD14 MARD15

Category 1 Category 1 0.840 0.848 -0.008 -0.410

Category 1 Category 2 0.072 0.079 -0.007 -0.467

Category 2 Category 1 0.031 0.039 -0.008 -0.749

Category 2 Category 2 0.056 0.034 0.022 2.295

MARD14 MARD16

Category 1 Category 1 0.794 0.785 0.009 0.397

Category 1 Category 2 0.132 0.142 -0.009 -0.512

Category 2 Category 1 0.039 0.032 0.006 0.664

Category 2 Category 2 0.035 0.041 -0.005 -0.515

MARD14 MARD17

Category 1 Category 1 0.683 0.691 -0.008 -0.316

Category 1 Category 2 0.254 0.236 0.018 0.790

Category 2 Category 1 0.025 0.027 -0.003 -0.299

Category 2 Category 2 0.039 0.046 -0.007 -0.670

MARD14 MARD18

Category 1 Category 1 0.504 0.508 -0.004 -0.162

Category 1 Category 2 0.418 0.419 -0.001 -0.036

Category 2 Category 1 0.021 0.021 0.000 -0.001

Category 2 Category 2 0.057 0.052 0.005 0.445

MARD14 MARD19

Category 1 Category 1 0.505 0.481 0.024 0.913

Category 1 Category 2 0.426 0.446 -0.019 -0.741

Category 2 Category 1 0.027 0.021 0.007 0.888

Category 2 Category 2 0.041 0.053 -0.011 -0.962

MARD14 MARD20

Category 1 Category 1 0.459 0.487 -0.028 -1.059

Category 1 Category 2 0.446 0.439 0.007 0.256

Category 2 Category 1 0.041 0.021 0.020 2.592

Category 2 Category 2 0.054 0.052 0.002 0.139

MARD15 MARD16

Category 1 Category 1 0.783 0.767 0.016 0.731

Category 1 Category 2 0.108 0.120 -0.012 -0.681

Category 2 Category 1 0.051 0.051 0.000 0.012

Category 2 Category 2 0.058 0.062 -0.005 -0.374

MARD15 MARD17

Category 1 Category 1 0.660 0.680 -0.019 -0.788

Category 1 Category 2 0.235 0.207 0.028 1.310

Category 2 Category 1 0.034 0.038 -0.005 -0.456

Category 2 Category 2 0.071 0.075 -0.004 -0.289

MARD15 MARD18

Category 1 Category 1 0.500 0.504 -0.004 -0.148

Category 1 Category 2 0.380 0.383 -0.003 -0.128

Category 2 Category 1 0.015 0.025 -0.010 -1.245

Category 2 Category 2 0.105 0.088 0.018 1.174

MARD15 MARD19

Category 1 Category 1 0.495 0.478 0.017 0.637

Category 1 Category 2 0.394 0.409 -0.016 -0.601

Category 2 Category 1 0.036 0.024 0.012 1.487

Category 2 Category 2 0.076 0.089 -0.013 -0.880

MARD15 MARD20

Category 1 Category 1 0.451 0.484 -0.033 -1.262

Category 1 Category 2 0.427 0.403 0.024 0.939

Category 2 Category 1 0.047 0.024 0.023 2.775

Category 2 Category 2 0.075 0.089 -0.014 -0.908

MARD16 MARD17

Category 1 Category 1 0.674 0.655 0.019 0.759

Category 1 Category 2 0.174 0.162 0.012 0.617

Category 2 Category 1 0.034 0.063 -0.029 -2.244

Category 2 Category 2 0.117 0.120 -0.002 -0.134

MARD16 MARD18

Category 1 Category 1 0.498 0.495 0.003 0.113

Category 1 Category 2 0.328 0.322 0.006 0.236

Category 2 Category 1 0.027 0.034 -0.007 -0.747

Category 2 Category 2 0.147 0.148 -0.002 -0.088

MARD16 MARD19

Category 1 Category 1 0.507 0.470 0.037 1.404

Category 1 Category 2 0.320 0.347 -0.027 -1.083

Category 2 Category 1 0.037 0.031 0.005 0.571

Category 2 Category 2 0.136 0.151 -0.015 -0.795

MARD16 MARD20

Category 1 Category 1 0.452 0.476 -0.024 -0.922

Category 1 Category 2 0.375 0.341 0.034 1.351

Category 2 Category 1 0.048 0.032 0.016 1.716

Category 2 Category 2 0.125 0.150 -0.025 -1.348

MARD17 MARD18

Category 1 Category 1 0.489 0.478 0.012 0.452

Category 1 Category 2 0.211 0.241 -0.030 -1.310

Category 2 Category 1 0.063 0.052 0.012 0.987

Category 2 Category 2 0.236 0.230 0.006 0.273

MARD17 MARD19

Category 1 Category 1 0.452 0.456 -0.004 -0.139

Category 1 Category 2 0.248 0.262 -0.014 -0.620

Category 2 Category 1 0.088 0.046 0.042 3.769

Category 2 Category 2 0.212 0.236 -0.024 -1.059

MARD17 MARD20

Category 1 Category 1 0.411 0.461 -0.050 -1.891

Category 1 Category 2 0.317 0.257 0.059 2.579

Category 2 Category 1 0.083 0.047 0.036 3.194

Category 2 Category 2 0.189 0.234 -0.046 -2.039

MARD18 MARD19

Category 1 Category 1 0.447 0.404 0.043 1.675

Category 1 Category 2 0.081 0.125 -0.044 -2.527

Category 2 Category 1 0.093 0.098 -0.005 -0.293

Category 2 Category 2 0.378 0.373 0.005 0.212

MARD18 MARD20

Category 1 Category 1 0.362 0.407 -0.045 -1.750

Category 1 Category 2 0.130 0.122 0.008 0.442

Category 2 Category 1 0.124 0.101 0.023 1.438

Category 2 Category 2 0.384 0.369 0.015 0.582

MARD19 MARD20

Category 1 Category 1 0.379 0.395 -0.016 -0.607

Category 1 Category 2 0.108 0.107 0.001 0.060

Category 2 Category 1 0.113 0.113 0.000 -0.022

Category 2 Category 2 0.400 0.385 0.015 0.587

TECHNICAL 4 OUTPUT

ESTIMATES DERIVED FROM THE MODEL

ESTIMATED MEANS FOR THE LATENT VARIABLES

SD SC ID IC GENDER

________ ________ ________ ________ ________

0.867 0.361 -0.030 0.275 0.448

ESTIMATED MEANS FOR THE LATENT VARIABLES

C_IMB

________

0.106

S.E. FOR ESTIMATED MEANS FOR THE LATENT VARIABLES

SD SC ID IC GENDER

________ ________ ________ ________ ________

0.088 0.055 0.253 0.246 0.026

S.E. FOR ESTIMATED MEANS FOR THE LATENT VARIABLES

C_IMB

________

0.016

EST./S.E. FOR ESTIMATED MEANS FOR THE LATENT VARIABLES

SD SC ID IC GENDER

________ ________ ________ ________ ________

9.833 6.555 -0.117 1.117 17.085

EST./S.E. FOR ESTIMATED MEANS FOR THE LATENT VARIABLES

C_IMB

________

6.519

TWO-TAILED P-VALUE FOR ESTIMATED MEANS FOR THE LATENT VARIABLES

SD SC ID IC GENDER

________ ________ ________ ________ ________

0.000 0.000 0.906 0.264 0.000

TWO-TAILED P-VALUE FOR ESTIMATED MEANS FOR THE LATENT VARIABLES

C_IMB

________

0.000

ESTIMATED COVARIANCE MATRIX FOR THE LATENT VARIABLES

SD SC ID IC GENDER

________ ________ ________ ________ ________

SD 0.379

SC 0.107 0.092

ID -0.889 0.020 7.215

IC -0.220 -0.023 1.990 0.941

GENDER 0.025 0.062 0.060 -0.093 0.247

C_IMB 0.029 0.000 -0.150 -0.027 0.011

ESTIMATED COVARIANCE MATRIX FOR THE LATENT VARIABLES

C_IMB

________

C_IMB 0.095

S.E. FOR ESTIMATED COVARIANCE MATRIX FOR THE LATENT VARIABLES

SD SC ID IC GENDER

________ ________ ________ ________ ________

SD 0.082

SC 0.029 0.016

ID 0.281 0.036 1.729

IC 0.091 0.021 0.530 0.368

GENDER 0.028 0.016 0.123 0.067 0.018

C_IMB 0.020 0.010 0.097 0.053 0.008

S.E. FOR ESTIMATED COVARIANCE MATRIX FOR THE LATENT VARIABLES

C_IMB

________

C_IMB 0.007

EST./S.E. FOR ESTIMATED COVARIANCE MATRIX FOR THE LATENT VARIABLES

SD SC ID IC GENDER

________ ________ ________ ________ ________

SD 4.614

SC 3.710 5.650

ID -3.169 0.561 4.174

IC -2.421 -1.076 3.753 2.555

GENDER 0.926 3.934 0.491 -1.387 13.398

C_IMB 1.452 -0.017 -1.540 -0.512 1.362

EST./S.E. FOR ESTIMATED COVARIANCE MATRIX FOR THE LATENT VARIABLES

C_IMB

________

C_IMB 13.398

TWO-TAILED P-VALUE FOR ESTIMATED COVARIANCE MATRIX FOR THE LATENT VARIABLES

SD SC ID IC GENDER

________ ________ ________ ________ ________

SD 0.000

SC 0.000 0.000

ID 0.002 0.575 0.000

IC 0.015 0.282 0.000 0.011

GENDER 0.354 0.000 0.624 0.166 0.000

C_IMB 0.146 0.986 0.124 0.609 0.173

TWO-TAILED P-VALUE FOR ESTIMATED COVARIANCE MATRIX FOR THE LATENT VARIABLES

C_IMB

________

C_IMB 0.000

ESTIMATED CORRELATION MATRIX FOR THE LATENT VARIABLES

SD SC ID IC GENDER

________ ________ ________ ________ ________

SD 1.000

SC 0.574 1.000

ID -0.538 0.025 1.000

IC -0.368 -0.077 0.764 1.000

GENDER 0.083 0.414 0.045 -0.193 1.000

C_IMB 0.154 -0.002 -0.181 -0.091 0.072

ESTIMATED CORRELATION MATRIX FOR THE LATENT VARIABLES

C_IMB

________

C_IMB 1.000

S.E. FOR ESTIMATED CORRELATION MATRIX FOR THE LATENT VARIABLES

SD SC ID IC GENDER

________ ________ ________ ________ ________

SD 0.000

SC 0.114 0.000

ID 0.083 0.043 0.000

IC 0.154 0.066 0.099 0.000

GENDER 0.089 0.096 0.091 0.131 0.000

C_IMB 0.103 0.110 0.114 0.171 0.053

S.E. FOR ESTIMATED CORRELATION MATRIX FOR THE LATENT VARIABLES

C_IMB

________

C_IMB 0.000

EST./S.E. FOR ESTIMATED CORRELATION MATRIX FOR THE LATENT VARIABLES

SD SC ID IC GENDER

________ ________ ________ ________ ________

SD 999.000

SC 5.040 999.000

ID -6.488 0.570 999.000

IC -2.382 -1.170 7.683 999.000

GENDER 0.939 4.331 0.496 -1.468 999.000

C_IMB 1.502 -0.017 -1.593 -0.533 1.373

EST./S.E. FOR ESTIMATED CORRELATION MATRIX FOR THE LATENT VARIABLES

C_IMB

________

C_IMB 999.000

TWO-TAILED P-VALUE FOR ESTIMATED CORRELATION MATRIX FOR THE LATENT VARIABLES

SD SC ID IC GENDER

________ ________ ________ ________ ________

SD 0.000

SC 0.000 0.000

ID 0.000 0.569 0.000

IC 0.017 0.242 0.000 0.000

GENDER 0.348 0.000 0.620 0.142 0.000

C_IMB 0.133 0.986 0.111 0.594 0.170

TWO-TAILED P-VALUE FOR ESTIMATED CORRELATION MATRIX FOR THE LATENT VARIABLES

C_IMB

________

C_IMB 0.000

DIAGRAM INFORMATION

Use View Diagram under the Diagram menu in the Mplus Editor to view the diagram.

If running Mplus from the Mplus Diagrammer, the diagram opens automatically.

Diagram output

u:\windows\dcn r&r\r&r analyses\prediction model\revised gmm umsv predicting mu two-part.dgm

Beginning Time: 14:34:51

Ending Time: 14:34:54

Elapsed Time: 00:00:03

MUTHEN & MUTHEN

3463 Stoner Ave.

Los Angeles, CA 90066

Tel: (310) 391-9971

Fax: (310) 391-8971

Web: www.StatModel.com

Support: Support@StatModel.com

Copyright (c) 1998-2018 Muthen & Muthen
