# Supplementary material for: Mind the gap: A review and recommendations for statistically evaluating Dual Systems models of adolescent risk behavior
Source: Dev Cogn Neurosci. 2019 Jul 25;39:100681. doi: 10.1016/j.dcn.2019.100681 (PMC6969358; doi:10.1016/j.dcn.2019.100681)
Supplement: Supplementary file 7 [file mmc7.docx]

Mplus VERSION 8.2

MUTHEN & MUTHEN

04/05/2019 3:54 PM

INPUT INSTRUCTIONS

Title: Growth Mixture Model with Unique Means and Shared Variances

Data: file is Dual Systems LDS R&R.dat;

Variable:

NAMES ARE

subject gender sr12r1 sr12r2 sr12r3 sr13r1 sr13r2 sr13r3

sr14r1 sr14r2 sr14r3 ic12r1 ic12r2 ic12r3 ic13r1 ic13r2

ic13r3 ic14r1 ic14r2 ic14r3;

!SR=sensitivity to reward, IC=inhibitory control

!12=age 12, 13=age 13, 14=age 14

!r1=response block 1, r2=response block 2, r3=response block 3

USEV

ic12-ic14 sr12-sr14;

missing are .;

classes=c(2);

Define:

!Creating Observed Variables for Growth Models

ic12= MEAN (ic12r1 ic12r2 ic12r3);

ic13= MEAN (ic13r1 ic13r2 ic13r3);

ic14= MEAN (ic14r1 ic14r2 ic14r3);

sr12= MEAN (sr12r1 sr12r2 sr12r3);

sr13= MEAN (sr13r1 sr13r2 sr13r3);

sr14= MEAN (sr14r1 sr14r2 sr14r3);

Analysis:

type=mixture;

model=nocovariances;

STARTS=100 20;

LRTSTARTS=0 0 100 20;

Processor=4;

Model:

!Growth Micture Model with Unique Means and Shared Variances

%Overall%

!Inhibitory Control Growth Curve

iic sic | ic12@0 ic13* ic14@1;

iic sic;

iic with sic;

!Residual for ic12 was estimated to be negative in the two

!class solution, and was constrained to be 0

ic12@0;

!Sensitivity to Reward Growth Curve

isr ssr | sr12@0 sr13* sr14@1;

isr ssr;

isr with ssr;

!Initial model estimated residual variance for sr12 to be negative,

!so it was set to 0

sr12@0;

sr13 sr14 (srres);

!Covariances between Inhibitory Control and Sensitivity to Reward

!Growth Factors

iic with isr;

iic with ssr;

sic with isr;

sic with ssr;

Output: res stdyx sampstat mod(3) svalues tech14;

*** WARNING

Data set contains cases with missing on all variables.

These cases were not included in the analysis.

Number of cases with missing on all variables: 25

1 WARNING(S) FOUND IN THE INPUT INSTRUCTIONS

SUMMARY OF ANALYSIS

Number of groups 1

Number of observations 362

Number of dependent variables 6

Number of independent variables 0

Number of continuous latent variables 4

Number of categorical latent variables 1

Observed dependent variables

Continuous

IC12 IC13 IC14 SR12 SR13 SR14

Continuous latent variables

IIC SIC ISR SSR

Categorical latent variables

C

Variables with special functions

ID variable SUBJECT

Estimator MLR

Information matrix OBSERVED

Optimization Specifications for the Quasi-Newton Algorithm for

Continuous Outcomes

Maximum number of iterations 100

Convergence criterion 0.100D-05

Optimization Specifications for the EM Algorithm

Maximum number of iterations 500

Convergence criteria

Loglikelihood change 0.100D-06

Relative loglikelihood change 0.100D-06

Derivative 0.100D-05

Optimization Specifications for the M step of the EM Algorithm for

Categorical Latent variables

Number of M step iterations 1

M step convergence criterion 0.100D-05

Basis for M step termination ITERATION

Optimization Specifications for the M step of the EM Algorithm for

Censored, Binary or Ordered Categorical (Ordinal), Unordered

Categorical (Nominal) and Count Outcomes

Number of M step iterations 1

M step convergence criterion 0.100D-05

Basis for M step termination ITERATION

Maximum value for logit thresholds 15

Minimum value for logit thresholds -15

Minimum expected cell size for chi-square 0.100D-01

Maximum number of iterations for H1 2000

Convergence criterion for H1 0.100D-03

Optimization algorithm EMA

Random Starts Specifications

Number of initial stage random starts 100

Number of final stage optimizations 20

Number of initial stage iterations 10

Initial stage convergence criterion 0.100D+01

Random starts scale 0.500D+01

Random seed for generating random starts 0

Input data file(s)

Dual Systems LDS R&R.dat

Input data format FREE

SUMMARY OF DATA

Number of missing data patterns 12

Number of y missing data patterns 12

Number of u missing data patterns 0

COVARIANCE COVERAGE OF DATA

Minimum covariance coverage value 0.100

PROPORTION OF DATA PRESENT FOR Y

Covariance Coverage

IC12 IC13 IC14 SR12 SR13

________ ________ ________ ________ ________

IC12 0.964

IC13 0.887 0.920

IC14 0.525 0.517 0.536

SR12 0.959 0.881 0.519 0.959

SR13 0.898 0.917 0.519 0.892 0.931

SR14 0.528 0.519 0.536 0.522 0.522

Covariance Coverage

SR14

________

SR14 0.539

SAMPLE STATISTICS

ESTIMATED SAMPLE STATISTICS

Means

IC12 IC13 IC14 SR12 SR13

________ ________ ________ ________ ________

-0.172 0.227 0.341 0.627 0.530

Means

SR14

________

0.571

Covariances

IC12 IC13 IC14 SR12 SR13

________ ________ ________ ________ ________

IC12 0.807

IC13 0.236 0.451

IC14 0.202 0.188 0.360

SR12 -0.051 -0.046 -0.076 0.411

SR13 -0.028 -0.027 -0.041 0.115 0.213

SR14 -0.030 -0.030 -0.025 0.073 0.080

Covariances

SR14

________

SR14 0.189

Correlations

IC12 IC13 IC14 SR12 SR13

________ ________ ________ ________ ________

IC12 1.000

IC13 0.391 1.000

IC14 0.374 0.467 1.000

SR12 -0.088 -0.106 -0.197 1.000

SR13 -0.067 -0.088 -0.147 0.388 1.000

SR14 -0.077 -0.103 -0.094 0.260 0.400

Correlations

SR14

________

SR14 1.000

MAXIMUM LOG-LIKELIHOOD VALUE FOR THE UNRESTRICTED (H1) MODEL IS -1537.314

UNIVARIATE SAMPLE STATISTICS

UNIVARIATE HIGHER-ORDER MOMENT DESCRIPTIVE STATISTICS

Variable/ Mean/ Skewness/ Minimum/ % with Percentiles

Sample Size Variance Kurtosis Maximum Min/Max 20%/60% 40%/80% Median

IC12 -0.173 -1.094 -3.309 0.29% -0.786 -0.157 0.033

349.000 0.808 1.319 1.776 0.29% 0.144 0.483

IC13 0.225 -0.683 -2.763 0.30% -0.248 0.113 0.264

333.000 0.452 1.538 2.041 0.30% 0.425 0.758

IC14 0.321 -0.768 -2.190 0.52% -0.071 0.211 0.335

194.000 0.362 2.048 1.955 0.52% 0.496 0.813

SR12 0.623 0.800 -1.253 0.29% 0.173 0.440 0.558

347.000 0.409 2.133 3.206 0.29% 0.677 1.025

SR13 0.528 1.217 -0.747 0.30% 0.173 0.358 0.445

337.000 0.212 2.388 2.521 0.30% 0.527 0.844

SR14 0.575 0.785 -0.303 0.51% 0.209 0.445 0.509

195.000 0.189 0.670 2.119 0.51% 0.597 0.880

RANDOM STARTS RESULTS RANKED FROM THE BEST TO THE WORST LOGLIKELIHOOD VALUES

2 perturbed starting value run(s) did not converge.

Final stage loglikelihood values at local maxima, seeds, and initial stage start numbers:

-1497.354 246261 38

-1497.354 136842 58

-1497.354 637345 19

-1497.354 347515 24

-1497.354 789985 67

-1497.354 259507 53

-1497.354 93468 3

-1497.354 432148 30

-1497.354 227563 63

-1497.354 749453 33

-1497.354 68985 17

-1497.354 963053 43

-1506.485 626891 32

-1506.485 285380 1

-1506.485 405079 68

-1506.551 957392 79

-1506.551 576596 99

-1506.551 268217 83

-1506.551 124999 96

-1506.551 569833 85

THE BEST LOGLIKELIHOOD VALUE HAS BEEN REPLICATED. RERUN WITH AT LEAST TWICE THE

RANDOM STARTS TO CHECK THAT THE BEST LOGLIKELIHOOD IS STILL OBTAINED AND REPLICATED.

THE MODEL ESTIMATION TERMINATED NORMALLY

MODEL FIT INFORMATION

Number of Free Parameters 24

Loglikelihood

H0 Value -1497.354

H0 Scaling Correction Factor 1.3448

for MLR

Information Criteria

Akaike (AIC) 3042.709

Bayesian (BIC) 3136.108

Sample-Size Adjusted BIC 3059.967

(n* = (n + 2) / 24)

FINAL CLASS COUNTS AND PROPORTIONS FOR THE LATENT CLASSES

BASED ON THE ESTIMATED MODEL

Latent

Classes

1 39.75048 0.10981

2 322.24952 0.89019

FINAL CLASS COUNTS AND PROPORTIONS FOR THE LATENT CLASSES

BASED ON ESTIMATED POSTERIOR PROBABILITIES

Latent

Classes

1 39.75048 0.10981

2 322.24952 0.89019

FINAL CLASS COUNTS AND PROPORTIONS FOR THE LATENT CLASSES

BASED ON THEIR MOST LIKELY LATENT CLASS MEMBERSHIP

Class Counts and Proportions

Latent

Classes

1 39 0.10773

2 323 0.89227

CLASSIFICATION QUALITY

Entropy 0.901

Average Latent Class Probabilities for Most Likely Latent Class Membership (Row)

by Latent Class (Column)

1 2

1 0.883 0.117

2 0.016 0.984

Classification Probabilities for the Most Likely Latent Class Membership (Column)

by Latent Class (Row)

1 2

1 0.866 0.134

2 0.014 0.986

Logits for the Classification Probabilities for the Most Likely Latent Class Membership (Column)

by Latent Class (Row)

1 2

1 1.868 0.000

2 -4.242 0.000

MODEL RESULTS

Two-Tailed

Estimate S.E. Est./S.E. P-Value

Latent Class 1

IIC |

IC12 1.000 0.000 999.000 999.000

IC13 1.000 0.000 999.000 999.000

IC14 1.000 0.000 999.000 999.000

SIC |

IC12 0.000 0.000 999.000 999.000

IC13 0.885 0.048 18.592 0.000

IC14 1.000 0.000 999.000 999.000

ISR |

SR12 1.000 0.000 999.000 999.000

SR13 1.000 0.000 999.000 999.000

SR14 1.000 0.000 999.000 999.000

SSR |

SR12 0.000 0.000 999.000 999.000

SR13 0.902 0.081 11.121 0.000

SR14 1.000 0.000 999.000 999.000

IIC WITH

SIC -0.263 0.035 -7.450 0.000

ISR 0.007 0.028 0.247 0.805

SSR 0.003 0.022 0.115 0.909

ISR WITH

SSR -0.330 0.045 -7.356 0.000

SIC -0.056 0.034 -1.678 0.093

SIC WITH

SSR 0.027 0.028 0.957 0.338

Means

IIC -2.065 0.152 -13.557 0.000

SIC 2.040 0.183 11.124 0.000

ISR 0.875 0.204 4.285 0.000

SSR -0.172 0.131 -1.318 0.187

Intercepts

IC12 0.000 0.000 999.000 999.000

IC13 0.000 0.000 999.000 999.000

IC14 0.000 0.000 999.000 999.000

SR12 0.000 0.000 999.000 999.000

SR13 0.000 0.000 999.000 999.000

SR14 0.000 0.000 999.000 999.000

Variances

IIC 0.365 0.045 8.135 0.000

SIC 0.334 0.049 6.751 0.000

ISR 0.403 0.042 9.486 0.000

SSR 0.336 0.057 5.858 0.000

Residual Variances

IC12 0.000 0.000 999.000 999.000

IC13 0.269 0.034 7.828 0.000

IC14 0.165 0.040 4.099 0.000

SR12 0.000 0.000 999.000 999.000

SR13 0.120 0.014 8.757 0.000

SR14 0.120 0.014 8.757 0.000

Latent Class 2

IIC |

IC12 1.000 0.000 999.000 999.000

IC13 1.000 0.000 999.000 999.000

IC14 1.000 0.000 999.000 999.000

SIC |

IC12 0.000 0.000 999.000 999.000

IC13 0.885 0.048 18.592 0.000

IC14 1.000 0.000 999.000 999.000

ISR |

SR12 1.000 0.000 999.000 999.000

SR13 1.000 0.000 999.000 999.000

SR14 1.000 0.000 999.000 999.000

SSR |

SR12 0.000 0.000 999.000 999.000

SR13 0.902 0.081 11.121 0.000

SR14 1.000 0.000 999.000 999.000

IIC WITH

SIC -0.263 0.035 -7.450 0.000

ISR 0.007 0.028 0.247 0.805

SSR 0.003 0.022 0.115 0.909

ISR WITH

SSR -0.330 0.045 -7.356 0.000

SIC -0.056 0.034 -1.678 0.093

SIC WITH

SSR 0.027 0.028 0.957 0.338

Means

IIC 0.061 0.046 1.339 0.181

SIC 0.289 0.048 6.029 0.000

ISR 0.596 0.036 16.424 0.000

SSR -0.077 0.038 -1.999 0.046

Intercepts

IC12 0.000 0.000 999.000 999.000

IC13 0.000 0.000 999.000 999.000

IC14 0.000 0.000 999.000 999.000

SR12 0.000 0.000 999.000 999.000

SR13 0.000 0.000 999.000 999.000

SR14 0.000 0.000 999.000 999.000

Variances

IIC 0.365 0.045 8.135 0.000

SIC 0.334 0.049 6.751 0.000

ISR 0.403 0.042 9.486 0.000

SSR 0.336 0.057 5.858 0.000

Residual Variances

IC12 0.000 0.000 999.000 999.000

IC13 0.269 0.034 7.828 0.000

IC14 0.165 0.040 4.099 0.000

SR12 0.000 0.000 999.000 999.000

SR13 0.120 0.014 8.757 0.000

SR14 0.120 0.014 8.757 0.000

Categorical Latent Variables

Means

C#1 -2.093 0.243 -8.617 0.000

STANDARDIZED MODEL RESULTS

STDYX Standardization

Two-Tailed

Estimate S.E. Est./S.E. P-Value

Latent Class 1

IIC |

IC12 1.000 0.000 999.000 999.000

IC13 0.922 0.062 14.887 0.000

IC14 1.039 0.086 12.103 0.000

SIC |

IC12 0.000 0.000 999.000 999.000

IC13 0.780 0.072 10.829 0.000

IC14 0.994 0.105 9.502 0.000

ISR |

SR12 1.000 0.000 999.000 999.000

SR13 1.414 0.080 17.574 0.000

SR14 1.421 0.086 16.582 0.000

SSR |

SR12 0.000 0.000 999.000 999.000

SR13 1.164 0.100 11.689 0.000

SR14 1.297 0.123 10.522 0.000

IIC WITH

SIC -0.753 0.047 -16.171 0.000

ISR 0.018 0.073 0.249 0.804

SSR 0.007 0.062 0.115 0.909

ISR WITH

SSR -0.896 0.024 -37.530 0.000

SIC -0.154 0.087 -1.756 0.079

SIC WITH

SSR 0.080 0.082 0.983 0.326

Means

IIC -3.418 0.261 -13.077 0.000

SIC 3.529 0.430 8.216 0.000

ISR 1.379 0.321 4.296 0.000

SSR -0.297 0.228 -1.302 0.193

Intercepts

IC12 0.000 0.000 999.000 999.000

IC13 0.000 0.000 999.000 999.000

IC14 0.000 0.000 999.000 999.000

SR12 0.000 0.000 999.000 999.000

SR13 0.000 0.000 999.000 999.000

SR14 0.000 0.000 999.000 999.000

Variances

IIC 1.000 0.000 999.000 999.000

SIC 1.000 0.000 999.000 999.000

ISR 1.000 0.000 999.000 999.000

SSR 1.000 0.000 999.000 999.000

Residual Variances

IC12 0.000 999.000 999.000 999.000

IC13 0.625 0.055 11.423 0.000

IC14 0.488 0.087 5.617 0.000

SR12 0.000 999.000 999.000 999.000

SR13 0.595 0.056 10.572 0.000

SR14 0.601 0.062 9.749 0.000

Latent Class 2

IIC |

IC12 1.000 0.000 999.000 999.000

IC13 0.922 0.062 14.887 0.000

IC14 1.039 0.086 12.103 0.000

SIC |

IC12 0.000 0.000 999.000 999.000

IC13 0.780 0.072 10.829 0.000

IC14 0.994 0.105 9.502 0.000

ISR |

SR12 1.000 0.000 999.000 999.000

SR13 1.414 0.080 17.574 0.000

SR14 1.421 0.086 16.582 0.000

SSR |

SR12 0.000 0.000 999.000 999.000

SR13 1.164 0.100 11.689 0.000

SR14 1.297 0.123 10.522 0.000

IIC WITH

SIC -0.753 0.047 -16.171 0.000

ISR 0.018 0.073 0.249 0.804

SSR 0.007 0.062 0.115 0.909

ISR WITH

SSR -0.896 0.024 -37.530 0.000

SIC -0.154 0.087 -1.756 0.079

SIC WITH

SSR 0.080 0.082 0.983 0.326

Means

IIC 0.102 0.079 1.278 0.201

SIC 0.501 0.086 5.834 0.000

ISR 0.939 0.064 14.669 0.000

SSR -0.132 0.066 -2.011 0.044

Intercepts

IC12 0.000 0.000 999.000 999.000

IC13 0.000 0.000 999.000 999.000

IC14 0.000 0.000 999.000 999.000

SR12 0.000 0.000 999.000 999.000

SR13 0.000 0.000 999.000 999.000

SR14 0.000 0.000 999.000 999.000

Variances

IIC 1.000 0.000 999.000 999.000

SIC 1.000 0.000 999.000 999.000

ISR 1.000 0.000 999.000 999.000

SSR 1.000 0.000 999.000 999.000

Residual Variances

IC12 0.000 999.000 999.000 999.000

IC13 0.625 0.055 11.423 0.000

IC14 0.488 0.087 5.617 0.000

SR12 0.000 999.000 999.000 999.000

SR13 0.595 0.056 10.572 0.000

SR14 0.601 0.062 9.749 0.000

R-SQUARE

Class 1

Observed Two-Tailed

Variable Estimate S.E. Est./S.E. P-Value

IC12 1.000 999.000 999.000 999.000

IC13 0.375 0.055 6.858 0.000

IC14 0.512 0.087 5.899 0.000

SR12 1.000 999.000 999.000 999.000

SR13 0.405 0.056 7.184 0.000

SR14 0.399 0.062 6.462 0.000

Class 2

Observed Two-Tailed

Variable Estimate S.E. Est./S.E. P-Value

IC12 1.000 999.000 999.000 999.000

IC13 0.375 0.055 6.858 0.000

IC14 0.512 0.087 5.899 0.000

SR12 1.000 999.000 999.000 999.000

SR13 0.405 0.056 7.184 0.000

SR14 0.399 0.062 6.462 0.000

QUALITY OF NUMERICAL RESULTS

Condition Number for the Information Matrix 0.254E-04

(ratio of smallest to largest eigenvalue)

MODEL COMMAND WITH FINAL ESTIMATES USED AS STARTING VALUES

%OVERALL%

iic sic | ic12@0 ic13* ic14@1;

isr ssr | sr12@0 sr13* sr14@1;

[ c#1*-2.09270 ];

%C#1%

sic BY ic13*0.88464 (1);

ssr BY sr13*0.90192 (2);

iic WITH sic*-0.26306 (11);

iic WITH isr*0.00698 (13);

iic WITH ssr*0.00250 (16);

isr WITH ssr*-0.32980 (18);

isr WITH sic*-0.05638 (14);

sic WITH ssr*0.02688 (17);

[ ic12@0 ];

[ ic13@0 ];

[ ic14@0 ];

[ sr12@0 ];

[ sr13@0 ];

[ sr14@0 ];

[ iic*-2.06549 ];

[ sic*2.03996 ];

[ isr*0.87541 ];

[ ssr*-0.17215 ];

ic12@0;

ic13*0.26852 (3);

ic14*0.16489 (4);

sr12@0;

sr13*0.12010 (srres);

sr14*0.12010 (srres);

iic*0.36520 (10);

sic*0.33406 (12);

isr*0.40314 (15);

ssr*0.33607 (19);

%C#2%

sic BY ic13*0.88464 (1);

ssr BY sr13*0.90192 (2);

iic WITH sic*-0.26306 (11);

iic WITH isr*0.00698 (13);

iic WITH ssr*0.00250 (16);

isr WITH ssr*-0.32980 (18);

isr WITH sic*-0.05638 (14);

sic WITH ssr*0.02688 (17);

[ ic12@0 ];

[ ic13@0 ];

[ ic14@0 ];

[ sr12@0 ];

[ sr13@0 ];

[ sr14@0 ];

[ iic*0.06134 ];

[ sic*0.28946 ];

[ isr*0.59621 ];

[ ssr*-0.07668 ];

ic12@0;

ic13*0.26852 (3);

ic14*0.16489 (4);

sr12@0;

sr13*0.12010 (srres);

sr14*0.12010 (srres);

iic*0.36520 (10);

sic*0.33406 (12);

isr*0.40314 (15);

ssr*0.33607 (19);

RESIDUAL OUTPUT

ESTIMATED MODEL AND RESIDUALS (OBSERVED - ESTIMATED) FOR CLASS 1

Model Estimated Means

IC12 IC13 IC14 SR12 SR13

________ ________ ________ ________ ________

-2.065 -0.261 -0.026 0.875 0.720

Model Estimated Means

SR14

________

0.703

Residuals for Means

IC12 IC13 IC14 SR12 SR13

________ ________ ________ ________ ________

-0.005 0.054 -0.030 -0.008 -0.037

Residuals for Means

SR14

________

-0.032

Model Estimated Covariances

IC12 IC13 IC14 SR12 SR13

________ ________ ________ ________ ________

IC12 0.365

IC13 0.132 0.430

IC14 0.102 0.165 0.338

SR12 0.007 -0.043 -0.049 0.403

SR13 0.009 -0.019 -0.023 0.106 0.202

SR14 0.009 -0.017 -0.020 0.073 0.079

Model Estimated Covariances

SR14

________

SR14 0.200

Residuals for Covariances

IC12 IC13 IC14 SR12 SR13

________ ________ ________ ________ ________

IC12 0.029

IC13 0.016 0.149

IC14 0.067 0.161 0.083

SR12 -0.044 -0.005 0.015 0.316

SR13 0.090 0.012 0.098 0.206 0.229

SR14 -0.027 0.109 0.023 -0.181 -0.088

Residuals for Covariances

SR14

________

SR14 -0.047

ESTIMATED MODEL AND RESIDUALS (OBSERVED - ESTIMATED) FOR CLASS 2

Model Estimated Means

IC12 IC13 IC14 SR12 SR13

________ ________ ________ ________ ________

0.061 0.317 0.351 0.596 0.527

Model Estimated Means

SR14

________

0.520

Residuals for Means

IC12 IC13 IC14 SR12 SR13

________ ________ ________ ________ ________

-0.001 -0.038 0.030 -0.004 -0.018

Residuals for Means

SR14

________

0.040

Model Estimated Covariances

IC12 IC13 IC14 SR12 SR13

________ ________ ________ ________ ________

IC12 0.365

IC13 0.132 0.430

IC14 0.102 0.165 0.338

SR12 0.007 -0.043 -0.049 0.403

SR13 0.009 -0.019 -0.023 0.106 0.202

SR14 0.009 -0.017 -0.020 0.073 0.079

Model Estimated Covariances

SR14

________

SR14 0.200

Residuals for Covariances

IC12 IC13 IC14 SR12 SR13

________ ________ ________ ________ ________

IC12 -0.003

IC13 0.003 -0.020

IC14 -0.040 -0.019 -0.012

SR12 0.005 0.013 -0.035 -0.041

SR13 0.000 -0.003 -0.010 -0.031 -0.020

SR14 -0.047 -0.020 0.001 -0.047 0.021

Residuals for Covariances

SR14

________

SR14 -0.006

MODEL MODIFICATION INDICES

NOTE: Modification indices for direct effects of observed dependent variables

regressed on covariates may not be included. To include these, request

MODINDICES (ALL).

Minimum M.I. value for printing the modification index 3.000

M.I. E.P.C. Std E.P.C. StdYX E.P.C.

CLASS 1

BY Statements

IIC BY IC13 3.924 -0.195 -0.118 -0.180

IIC BY IC14 5.652 0.275 0.166 0.286

SIC BY IC13 6.588 0.086 0.050 0.076

SIC BY IC14 4.320 -0.250 -0.145 -0.249

ISR BY IC12 7.187 -0.766 -0.487 -0.805

ISR BY IC13 3.113 0.269 0.171 0.261

ISR BY SR12 9.285 0.411 0.261 0.411

ISR BY SR13 302.990 8.238 5.231 11.647

SSR BY SR12 5.433 -0.355 -0.206 -0.324

SSR BY SR13 21.043 -0.745 -0.432 -0.962

ON/BY Statements

ISR ON IIC /

IIC BY ISR 999.000 0.000 0.000 0.000

ISR ON SIC /

SIC BY ISR 999.000 0.000 0.000 0.000

ISR ON ISR /

ISR BY ISR 56.051 1.022 1.022 1.022

ISR ON SSR /

SSR BY ISR 25.337 -0.717 -0.655 -0.655

SSR ON IIC /

IIC BY SSR 6.056 0.656 0.684 0.684

SSR ON SIC /

SIC BY SSR 11.879 -6.727 -6.707 -6.707

SSR ON ISR /

ISR BY SSR 22.359 0.781 0.856 0.856

SSR ON SSR /

SSR BY SSR 6.440 -0.366 -0.366 -0.366

WITH Statements

IC13 WITH IC12 10.158 -0.334 -0.334 999.000

SR12 WITH IC12 3.282 -0.131 -0.131 999.000

SR13 WITH IC12 999.000 0.000 0.000 999.000

SR13 WITH IC14 14.577 0.228 0.228 1.621

SR13 WITH SR12 11.919 0.167 0.167 999.000

SR14 WITH IC13 6.725 0.146 0.146 0.814

SIC WITH IIC 4.111 0.072 0.206 0.206

ISR WITH IIC 9.894 0.170 0.442 0.442

ISR WITH SIC 21.643 0.251 0.684 0.684

SSR WITH IIC 9.056 0.116 0.332 0.332

SSR WITH SIC 10.903 0.138 0.411 0.411

SSR WITH ISR 32.706 0.185 0.502 0.502

Means/Intercepts/Thresholds

[ IC12 ] 4.386 -4.906 -4.906 -8.118

[ IC13 ] 4.386 0.566 0.566 0.863

[ IC14 ] 4.387 -0.640 -0.640 -1.100

Variances/Residual Variances

IC12 11.406 1.461 1.461 4.000

IC13 3.700 0.183 0.183 0.427

SR12 3.788 0.187 0.187 0.463

SR13 999.000 0.000 0.000 0.000

SIC 8.539 0.280 0.838 0.838

ISR 140.911 0.854 2.119 2.119

SSR 5.677 0.098 0.293 0.293

CLASS 2

BY Statements

SIC BY IC13 6.588 -0.185 -0.107 -0.163

SIC BY IC14 4.320 0.250 0.145 0.249

ISR BY IC12 5.895 0.595 0.378 0.625

ISR BY SR12 7.408 -0.361 -0.229 -0.361

ISR BY SR13 15.171 -0.262 -0.166 -0.370

SSR BY SR12 5.433 0.355 0.206 0.324

SSR BY SR13 21.043 0.114 0.066 0.147

ON/BY Statements

ISR ON IIC /

IIC BY ISR 999.000 0.000 0.000 0.000

ISR ON SIC /

SIC BY ISR 999.000 0.000 0.000 0.000

ISR ON ISR /

ISR BY ISR 56.051 -1.022 -1.022 -1.022

ISR ON SSR /

SSR BY ISR 25.337 0.717 0.655 0.655

SSR ON IIC /

IIC BY SSR 6.055 -0.656 -0.684 -0.684

SSR ON SIC /

SIC BY SSR 11.879 6.727 6.707 6.707

SSR ON ISR /

ISR BY SSR 22.359 -0.781 -0.856 -0.856

SSR ON SSR /

SSR BY SSR 6.440 0.366 0.366 0.366

WITH Statements

SR13 WITH SR12 7.651 -0.119 -0.119 999.000

SIC WITH IIC 4.111 -0.013 -0.037 -0.037

ISR WITH IIC 9.894 0.020 0.053 0.053

ISR WITH SIC 21.643 0.001 0.002 0.002

SSR WITH IIC 9.056 -0.008 -0.023 -0.023

SSR WITH SIC 10.903 -0.015 -0.046 -0.046

SSR WITH ISR 32.706 -0.019 -0.050 -0.050

Means/Intercepts/Thresholds

[ IC12 ] 3.518 0.761 0.761 1.259

[ IC13 ] 3.518 -0.088 -0.088 -0.134

[ IC14 ] 3.518 0.099 0.099 0.171

Variances/Residual Variances

IC13 3.700 -0.023 -0.023 -0.054

SR12 4.155 -0.195 -0.195 -0.483

SIC 8.539 -0.006 -0.018 -0.018

ISR 140.910 -0.158 -0.391 -0.391

SSR 5.677 -0.016 -0.048 -0.048

Categorical Latent Variables

No modification indices above the minimum value.

TECHNICAL 14 OUTPUT

Random Starts Specifications for the k-1 Class Analysis Model

Number of initial stage random starts 100

Number of final stage optimizations 20

Random Starts Specification for the k-1 Class Model for Generated Data

Number of initial stage random starts 0

Number of final stage optimizations for the

initial stage random starts 0

Random Starts Specification for the k Class Model for Generated Data

Number of initial stage random starts 100

Number of final stage optimizations 20

Number of bootstrap draws requested Varies

PARAMETRIC BOOTSTRAPPED LIKELIHOOD RATIO TEST FOR 1 (H0) VERSUS 2 CLASSES

H0 Loglikelihood Value -1540.897

2 Times the Loglikelihood Difference 87.085

Difference in the Number of Parameters 5

Approximate P-Value 0.0000

Successful Bootstrap Draws 5

SAVEDATA INFORMATION

Save file

gmm_2classumsv.txt

Order of variables

IC12

IC13

IC14

SR12

SR13

SR14

CPROB1

CPROB2

C

SUBJECT

Save file format Free

Save file record length 10000

DIAGRAM INFORMATION

Mplus diagrams are currently not available for Mixture analysis.

No diagram output was produced.

Beginning Time: 15:54:12

Ending Time: 15:54:37

Elapsed Time: 00:00:25

MUTHEN & MUTHEN

3463 Stoner Ave.

Los Angeles, CA 90066

Tel: (310) 391-9971

Fax: (310) 391-8971

Web: www.StatModel.com

Support: Support@StatModel.com

Copyright (c) 1998-2018 Muthen & Muthen
